# Untargeted lipidomic analysis and network pharmacology for parthenolide treated papillary thyroid carcinoma cells

**DOI:** 10.1186/s12906-023-03944-7

**Published:** 2023-04-24

**Authors:** Le-Tian Huang, Tie-Jun Li, Ming-Lin Li, Han-Yong Luo, Yi-Bing Wang, Jia-He Wang

**Affiliations:** 1 Department of Oncology, Shengjing Hospital of China Medical University, Shenyang, China; 2 Department of Cardiology, Shengjing Hospital of China Medical University, Shenyang, China; 3 Department of Family Medicine, Shengjing Hospital of China Medical University, Shenyang, China; 4 Department of Urology, Shengjing Hospital of China Medical University, Shenyang, China

**Keywords:** Lipidomics, Network pharmacology, Parthenolide, Phosphatidylcholine, Thyroid cancer

## Abstract

**Background:**

With fast rising incidence, papillary thyroid carcinoma (PTC) is the most common head and neck cancer. Parthenolide, isolated from traditional Chinese medicine, inhibits various cancer cells, including PTC cells. The aim was to investigate the lipid profile and lipid changes of PTC cells when treated with parthenolide.

**Methods:**

Comprehensive lipidomic analysis of parthenolide treated PTC cells was conducted using a UHPLC/Q-TOF–MS platform, and the changed lipid profile and specific altered lipid species were explored. Network pharmacology and molecular docking were performed to show the associations among parthenolide, changed lipid species, and potential target genes.

**Results:**

With high stability and reproducibility, a total of 34 lipid classes and 1736 lipid species were identified. Lipid class analysis indicated that parthenolide treated PTC cells contained higher levels of fatty acid (FA), cholesterol ester (ChE), simple glc series 3 (CerG3) and lysophosphatidylglycerol (LPG), lower levels of zymosterol (ZyE) and Monogalactosyldiacylglycerol (MGDG) than controlled ones, but with no significant differences. Several specific lipid species were changed significantly in PTC cells treated by parthenolide, including the increasing of phosphatidylcholine (PC) (12:0e/16:0), PC (18:0/20:4), CerG3 (d18:1/24:1), lysophosphatidylethanolamine (LPE) (18:0), phosphatidylinositol (PI) (19:0/20:4), lysophosphatidylcholine (LPC) (28:0), ChE (22:6), and the decreasing of phosphatidylethanolamine (PE) (16:1/17:0), PC (34:1) and PC (16:0p/18:0). Four key targets (PLA2G4A, LCAT, LRAT, and PLA2G2A) were discovered when combining network pharmacology and lipidomics. Among them, PLA2G2A and PLA2G4A were able to bind with parthenolide confirmed by molecular docking.

**Conclusions:**

The changed lipid profile and several significantly altered lipid species of parthenolide treated PTC cells were observed. These altered lipid species, such as PC (34:1), and PC (16:0p/18:0), may be involved in the antitumor mechanisms of parthenolide. PLA2G2A and PLA2G4A may play key roles when parthenolide treated PTC cells.

**Supplementary Information:**

The online version contains supplementary material available at 10.1186/s12906-023-03944-7.

## Introduction

Thyroid carcinoma is the most frequent type of head and neck cancer, with an incidence rate among women that is three times greater than that among men, accounting for around 5% of cancer cases reported among women [1]. Papillary thyroid carcinoma (PTC) is the most common pathologic subtype, comprising 70–80% of all thyroid cancers [2], and has a relatively favourable prognosis, with a 90% 10-year survival rate [3]. However, with the global incidence of PTC rising rapidly, an increasing number of these patients are unable to be controlled due to distant metastases [1], making it imperative to explore deeper into its pathogenesis and to develop better treatments, with the aim of improving the overall prognosis of thyroid cancer.

Parthenolide is a sesquiterpene lactone, which is isolated and purified from traditional herbal medicine feverfew (*Tanacetum parthenium*). It has been widely utilized for its anti-inflammatory and antioxidant properties, with many studies demonstrating its effectiveness in treating headaches, fever, and rheumatoid arthritis [4, 5]. In recent years, numerous studies have explored its potential in inhibiting the growth of certain cancer cells, such as those found in breast, lung, and colorectal cancers [6–8]. The antitumor effects of parthenolide are thought to be due to its ability to inhibit signal transducer and activator of transcription 3 (STAT3) and nuclear factor κB (NF-κB), resulting in epithelial-mesenchymal transition (EMT) and other processes [9–11].

A recent study from our team has revealed that parthenolide can promote apoptosis of PTC cells in a concentration-dependent manner [5]. To investigate the pharmacological effects of parthenolide on PTC cells, metabolomics were utilized and it was observed that these metabolites were mainly involved in the lipid metabolism, tricarboxylic acid cycle, choline metabolism, and amino acid metabolism. These findings indicate that parthenolide can inhibit the growth and proliferation of PTC cells by enhancing oxidative stress response and metabolic imbalance, particularly in terms of amino acid and lipid changes [5, 12]. Furthermore, we demonstrated that parthenolide can lead to proteomic differences in PTC cells (BCPAP cells) [13].

Lipids play a pivotal role in cell membrane structure, cell differentiation, proliferation, and metabolism regulation. Additionally, aberrations in lipid biosynthesis and metabolism have been linked to cancer cell invasiveness and metastasis. Consequently, it is essential to further investigate the alterations in lipid metabolism that occur during malignancy and treatment [14–17].

In recent years, the development of lipidomics has been accelerated by advances in mass spectrometry. Several studies have proposed that certain aberrant lipid classes or species may be novel biomarkers for tumors; for example, glycerolipids have been used to detect early-stage breast cancer [18], and a combination of phosphatidylcholine (PC) (14:0/18:2), phosphatidylethanolamine (PE) (16:1e/18:2), and PE (15:1e/22:6) have been used to identify early stage cervical cancer [19]. After exposing endocrine disruptors to prostate cancer cells, some significant lipid changes were identified, with vital lipid-metabolism pathways being involved [20]. Thus, lipidomic analysis can not only identify potential biomarkers and therapeutic targets for cancer, but also help understand the pathogenesis of cancer and the mechanism of antitumor drugs.

The present study utilized network pharmacology and molecular docking to analyze the potential targets of parthenolide in PTC cells, and to further investigate the upstream molecular mechanisms and drug-binding affinity of parthenolide. Specifically, network pharmacology was used to identify the targets that parthenolide acted on, and the proteins that modulated the lipid metabolites identified from lipidomics [21, 22]. Additionally, molecular docking was employed to predict the binding strength between parthenolide and its targets at the spatial level [23]. The above methods will be used to initially explore the key targets of parthenolide in PTC cells.

In this research, an untargeted lipidomic analysis was performed using chemometric analysis tools in order to explore the lipid profile and changes of PTC cells treated with parthenolide. Additionally, network pharmacology and molecular docking were conducted to explore the potential targets of parthenolide against PTC. The aim of this study was to explore the lipid profile and lipid changes and to identify the potential targets of parthenolide against PTC.

## Materials and methods

### Reagents

Parthenolide was purchased from Absin (Shanghai, China), and human PTC cell line BCPAP was purchased from Shanghai Institutes for Biological Sciences, China. RPMI 1640 medium was purchased from Corning, USA. Fetal bovine serum was purchased from Shuangru Biology ScienceandTechnology Co.Ltd. HPLC-grade formic acid and HPLC-grade ammonium formates were purchased from Sigma. MS-grade acetonitrile, MS-grade methanol, and HPLC-grade 2-propanol were purchased from Thermo Fisher.

### Cell culture and treatment

BCPAP containing 10% fetal bovine serum, 100 U/mL streptomycin, and 100 U/ml penicillin, was maintained in a complete RPMI 1640 medium. BCPAP were cultured in an environment of 5% CO_2_, 37 °C. Sufficient cell samples were divided into 12 groups. According to the appropriate concentration (IC50) explored in the preliminary experiment [13], parthenolide was dissolved in 0.05% DMSO and diluted with PBS to 10 μM and was added to each treatment group (*n* = 6) for 24 h, and 6 control groups were added with the same amount of complete culture medium.

### Lipid extraction and sample preparation

The samples were homogenized with 200 µL water and 240 µL methanol. Then 800 µL of MTBE was added and the mixture was managed by ultrasound at 4 degree centigrade for 30 min at room temperature. Finally, the solution was centrifuged at 14,000 rpm for 15 min at 10 degree centigrade to obtain the supernatants and dried with nitrogen.

### LC–MS/MS method for lipid analysis

Reverse phase chromatography was used for liquid chromatography separation with a column (Waters, CSH C18, 1.7 µm, 2.1 mm × 100 mm). The lipid extracts were re-dissolved in 200 µL 90% isopropanol /acetonitrile, then centrifuged at 14,000 rpm at 10 degree centigrade for 15 min, at last 3 µL of each sample was injected onto the CSH C18 column. Solvent A contained acetonitrile–water (6:4, v/v) with 0.1 mm ammonium formate and 0.1% formic acid. Solvent B contained acetonitrile–isopropanol (1:9, v/v) with 0.1 mm ammonium formate and 0.1% formic acid. With a flow rate of 300 μL/min, 30% solvent B was maintained for 2 min. After that, solvent B increases to 100% in 23 min, and then it was balanced at 5% for 10 min. Mass spectra was performed on a Q-Exactive Plus in positive and negative mode, respectively. ESI parameters were adopted for all measurements as follows: heater temperature, 300 degree centigrade; sweep gas flow rate 1 arb; aux gas flow rate 15 arb; sheath gas flow rate 45 arb; spray voltage 3.0 kV and 2.5 kV for positive and negative electrospray ionization mode, respectively; S-Lens RF Level 50% and 60% for positive and negative, respectively; and the scan ranges 200–1800 m/z and 250–1800 m/z for positive and negative, respectively.

Based on MS/MS math, LipidSearch was used for the identification of lipid species. LipidSearch contains more than 1,500,000 fragment ions and more than 30 lipid classes in the database. Both mass tolerance for precursor and fragment were set to 5 ppm.

### Pharmacology network construction

In order to discover the related disease targets, the keyword ‘‘papillary thyroid carcinoma” was searched from OMIM Database (http://omim.org/), GeneCards Database (http://www.genecards.org/), and DrugBank (http://go.drugbank.com/). In addition, the targets of parthenolide were retrieved from SEA search server (http://sea.cbkslab.org/), STITCH (http://stitch.embl.de/), SwissTargetPrediction (http://swisstargetprediction.ch/), and PharmMapper server (http://www.lilab-ecust.cn/pharmmapper/). The intersection of the above targets was considered the potential targets of parthenolide against PTC. Afterward, the standard target names were obtained from UniProtKB (http://www.uniprot.org/). The lipid targets were generated from the differential lipid metabolites by using MetScape in Cytoscape 3.8.0 software. Then, a protein–protein interaction (PPI) network was presented by STRING 11.5 (http://cn.string-db.org/) to show the link between these predicted targets and lipid targets. Hub targets were obtained via CytoHubba in Cytoscape. As a result, the compound-targets-metabolites network was constructed containing relationships among parthenolide, relevant target genes, and lipid metabolites.

### Molecular docking

The three-dimensional structure of parthenolide was acquired from PubChem Compound (PubChem CID: 7,251,185). The structure was preprocessed by adding hydrogen atoms and extracting water molecules. The protein structures of the hub targets were obtained from PDB database (http://www.rcsb.org/). Four protein targets were studied: PLA2G4A (PDB ID: 1RLW), LCAT (PDB ID: 4X90), LRAT (PDB ID: 4DPZ), and PLA2G2A (PDB ID: 3U8I). Then, the molecular docking was performed using LibDock with the default docking parameters, and the results were sequenced according to the LibDockScore of each protein.

### Statistical analysis

The data extracted by LipidSearch were analyzed, including univariate statistical analysis, multivariate statistical analysis, as well as hierarchical clustering and correlation analysis. Student's t-test and multiple of variation analysis were used for univariate statistical analysis. The lipid profiles showing differences with lower than 0.67 fold decrease or more than 1.5 fold increase along with *p* value < 0.05 were supposed to be significantly different lipids among parthenolide treated cells. Multivariate statistical analysis included un-supervised principal component analysis (PCA), supervised partial least squares discrimination analysis (PLS-DA), as well as orthogonal partial least squares discrimination analysis (OPLS-DA). Discriminant lipids were determined by the variable importance in the projection (VIP) parameter (VIP > 1,) and *p* value (*P* value < 0.05). Lipid Pathway Enrichment Analysis (LIPEA) software was used to perform the pathway enrichment analysis of metabolites [24].

## Results

### Lipidomic analysis showed high stability and reproducibility

To evaluate the stability and repeatability of the experiment, each group of samples was equally mixed into the quality control sample (QC). The results of QC UHPLC-obitrap MS base peak were compared with overlapping spectra, which showed that the response intensity overlapped significantly, as well as the retention time of chromatographic peaks, indicating that this experiment was highly reproducible. The results of pearson correlation analysis showed that the coefficient between QC samples is above 0.9, indicating good reproducibility. All QC and experimental samples were extracted and PCA analysis was performed after Pareto-scaling. The QC samples were closely clustered, indicating that lipidomic analysis was highly reproducible (Additional file 1).

### Characterization of lipid composition

**Fig. 1:**
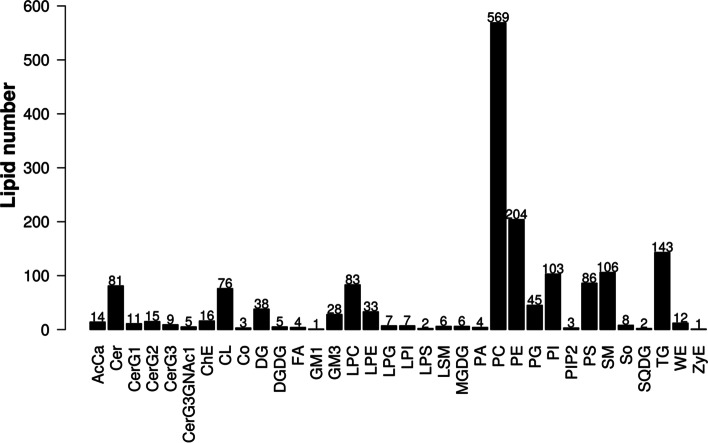
The lipid class and number identified in this study

The data obtained from positive- and negative-ion modes were analyzed qualitatively and quantitatively by using the LipidSearch software. In total, 34 lipid classes and 1736 lipid species were identified. The specific results are presented in Additional file 2. Figure 1 shows the lipid class identified in this study and the number of lipid class.

**Fig. 2:**
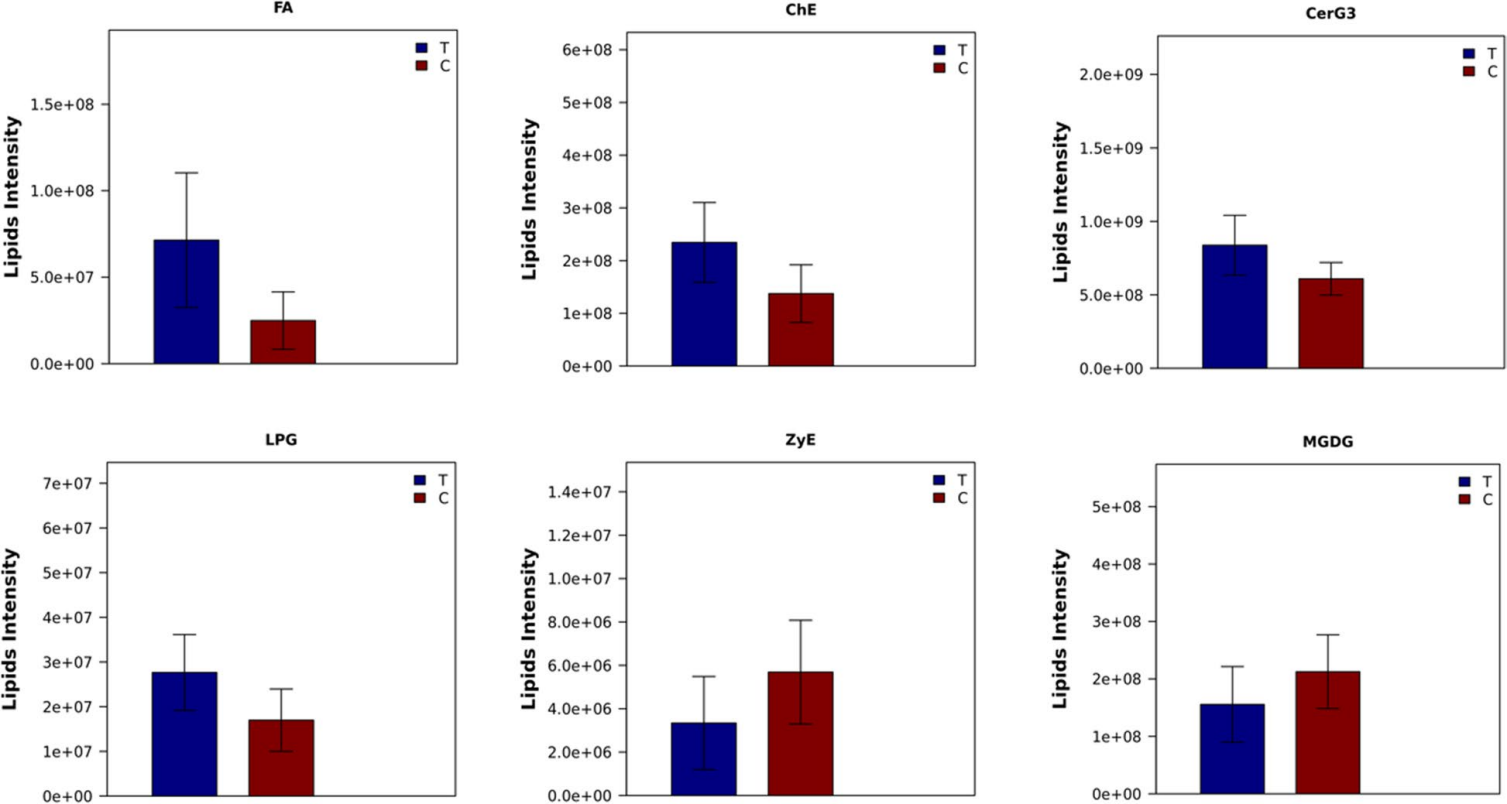
Parthenolide (T) altered the abundance of lipid class in papillary thyroid cells compared with control group (C), but with no significance. All *P* > 0.05. FA, fatty acid; ChE, cholesterol ester; CerG3, simple glc series 3; LPG, lysophosphatidylglycerol; ZyE, zymosterol; MGDG, monogalactosyldiacylglycerol

Lipid class analysis indicated that parthenolide treated BCPAP PTC cells contained higher levels of fatty acid (FA), cholesterol ester (ChE), simple glc series 3 (CerG3) and lysophosphatidylglycerol (LPG), lower levels of zymosterol (ZyE) and monogalactosyldiacylglycerol (MGDG) than controlled ones, but with no significant differences. The levels of other lipid classes were similar in both groups. (Fig. 2, Additional file 3).

### Parthenolide markedly altered the lipid profile

**Fig. 3:**
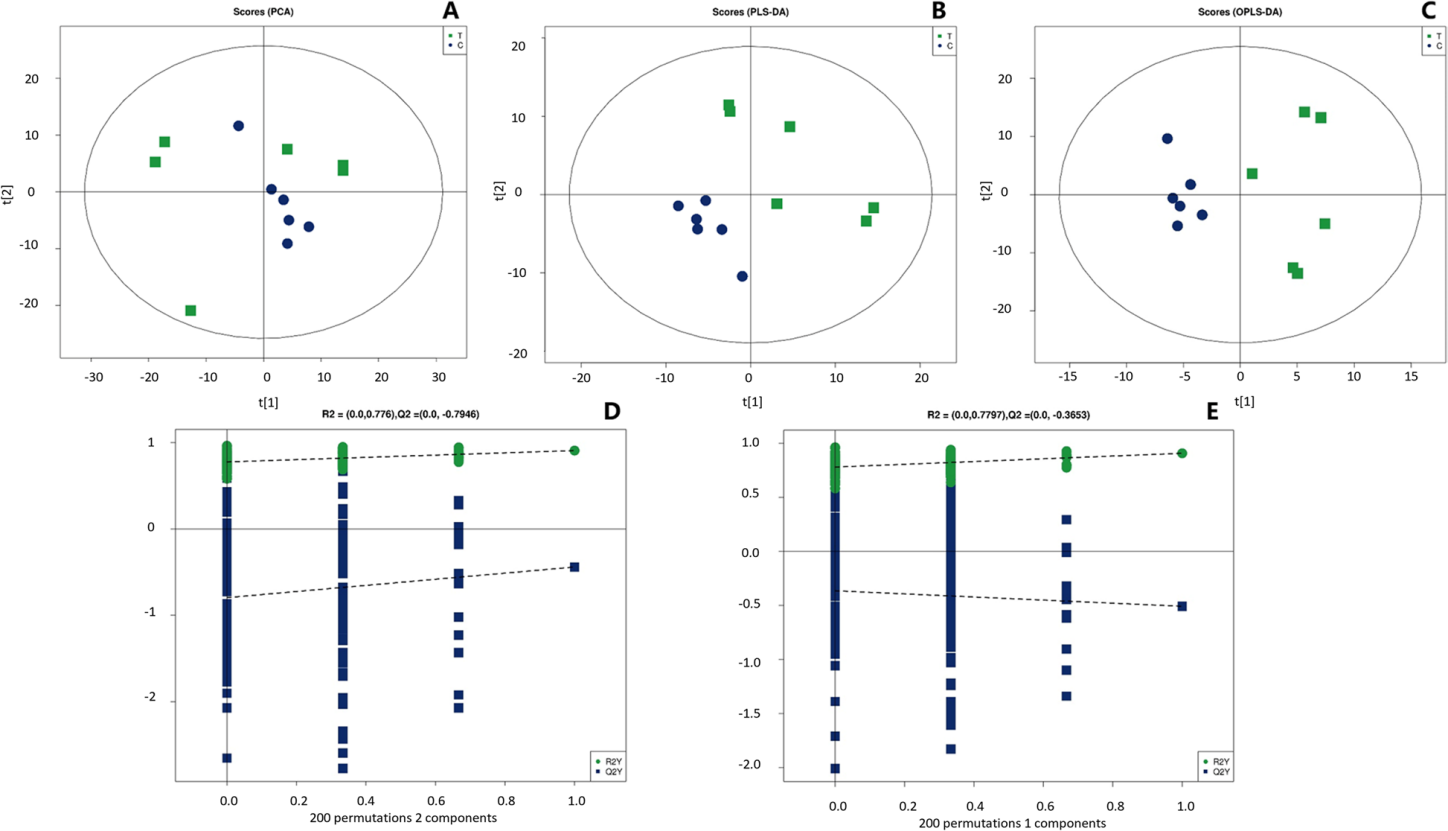
**A** Visible separation was not identified clearly between the two groups from the score plot of principal component analysis (PCA). **B** Partial least-squares determinant analysis (PLS-DA) showed a complete separation of detected ions in parthenolide treated groups from control groups [R2X (cum) = 0.384, R2Y (cum) = 0.907, Q2 (cum) = 0.44]. **C** Orthogonal projections to latent structures discriminant analysis (OPLS-DA) showed a complete separation of detected ions in parthenolide treated groups from control groups [R2X (cum) = 0.384, R2Y (cum) = 0.907, Q2 (cum) = 0.509]. **D** Permutation test of the PLS-DA model [Q2-intercept = -0.7946]. **E** Permutation test of the OPLS-DA model [Q2-intercept = -0.3653]

Visible separation was not identified clearly between the two groups from the score plot of PCA (Fig. 3A). PLS-DA and OPLS-DA model (Fig. 3B and C), however, showed a complete separation of detected ions in parthenolide treated groups from control groups, with a R2X (cum)-value of 0.384, a R2Y (cum)-value of 0.907, a Q2 (cum)-value of 0.44, and with a R2X (cum)-value of 0.384, a R2Y (cum)-value of 0.907, a Q2 (cum)-value of 0.509, respectively. Permutation tests were performed for avoiding overfitting (Fig. 3D and E).

**Fig. 4:**
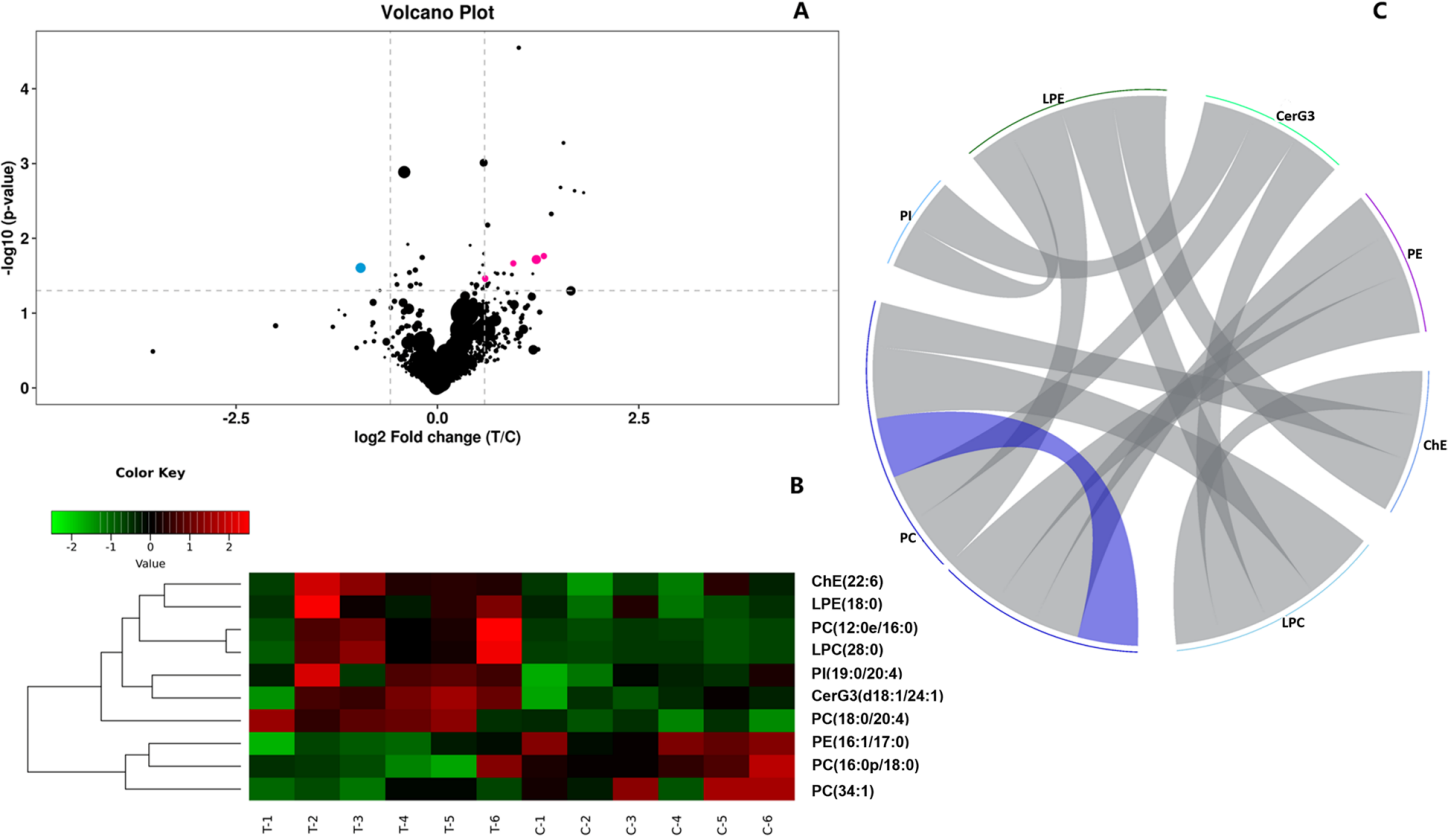
**A** The volcano plot of lipid species between the two groups based on the results of univariate analysis (fold change (FC) > 1.5 or FC < 0.67, *P*-value < 0. 05 and VIP > 1). **B** Hierarchical clustering of the 10 significantly changed lipid species. **C** The correlation analysis shows the correlation between the significant lipid species

The VIP obtained by the OPLS-DA model can be used to measure the impact strength of various lipid species on the discrimination of each group of samples. Univariate analysis can be used to visualize the significance of lipid species changes between the two groups (Fig. 4A), thus helping us to screen potential marker lipid species based on VIP and *P*-values (*P*-value < 0. 05 and VIP > 1). A total of 10 lipid species were selected and listed in Table 1, including PC (12:0e/16:0), PC (18:0/20:4), CerG3 (d18:1/24:1), lysophosphatidylethanolamine (LPE) (18:0), phosphatidylinositol (PI) (19:0/20:4), lysophosphatidylcholine (LPC) (28:0), ChE (22:6), and the decreasing of PE (16:1/17:0), PC (34:1) and PC (16:0p/18:0).

**Table 1:** Ten significant changed lipid species of parthenolide treated papillary thyroid carcinoma cells

LipidIon	Class	IonFormula	CalMz	RT-(min)	Fold Change	*P*-value	VIP
PE(16:1/17:0)-H	PE	C38 H73 O8 N1 P1	702.5079	10.2916	0.751478	0.0013	3.050149
PC(34:1) + H	PC	C42 H83 O8 N1 P1	760.5851	11.2935	0.516481	0.024864	2.369579
LPC(28:0) + H	LPC	C36 H75 O7 N1 P1	664.5276	9.12472	2.341388	0.019202	2.103261
PC(18:0/20:4) + HCOO	PC	C47 H85 O10 N1 P1	854.5917	10.46383	1.488194	0.000975	1.749522
CerG3(d18:1/24:1) + H	CerG3	C60 H112 O18 N1	1134.787	11.45426	1.507216	0.034294	1.248035
PC(12:0e/16:0) + HCOO	PC	C37 H75 O9 N1 P1	708.5185	9.155024	2.497935	0.017275	1.236085
ChE(22:6) + NH4	ChE	C49 H80 O2 N1	714.6184	15.11703	1.920327	0.021625	1.219279
PC(16:0p/18:0) + Na	PC	C42 H84 O7 N1 P1 Na1	768.5878	10.90136	0.793529	0.043174	1.087524
LPE(18:0)-H	LPE	C23 H47 O7 N1 P1	480.3096	3.931845	1.398238	0.042449	1.079679
PI(19:0/20:4)-H	PI	C48 H84 O13 N0 P1	899.5655	9.793264	1.509611	0.035162	1.041106

*CerG3* simple glc series 3, *ChE* Cholesterol ester, *LPC* Lysophosphatidylcholine, *LPE* Lysophosphatidylethanolamine, *PC* Phosphatidylcholine, *PE* Phosphatidylethanolamine, *PI* Phosphatidylinositol, *VIP* the variable importance in the projection

In order to assess the rationality of different lipid species, and to show the association between the samples and the expression patterns of lipid species in different samples more comprehensively, a hierarchical clustering (based on analysis of the Pearson correlation coefficients) was performed to demonstrate the 10 lipid species that have changed significantly. As shown in Fig. 4B, it revealed that these significantly changed lipid species formed a cluster, which means that they have similar expression patterns and may have close relation during the lipid metabolic process.

The correlation analysis was performed to obtain the correlation degree between the significant lipid species, and help measure the closeness of differences between lipid classes and species in the lipid metabolic process, further understand the relationship between lipid species in the process of biological changes. In this study, the correlation analysis of selected ten lipid species showed significant positive correlations between LPE (18:0) and PC (12:0e/16:0), ChE(22:6), and LPC (28:0), respectively, between LPC (28:0) and PC (12:0e/16:0), and between CerG3(d18:1/24:1) and PI (19:0/20:4) (*P* < 0.01, |r|> 0.5, Fig. 4C, Additional file 3). The significant negative correlations were found between PC (18:0/20:4) and PC (16:0p/18:0), as well as PC (18:0/20:4) and PE (16:1/17:0) (*P* < 0.01, |r|> 0.5, Fig. 4C, Additional file 4).

### Pathway enrichment analysis

LIPEA software was used to analyze the pathway enrichment of metabolites. The results demonstrated that glycerophospholipid was highly ranked (64%) and it was closely related to a group of significant lipid species identified in this study after PTC cells treated with parthenolide. Other pathways identified included ferroptosis (27%), glycosylphosphatidylinositol—anchor biosynthesis (18%), autophagy—other (18%), and autophagy—animal (18%) (Table 2).

**Table 2:** Lipid Pathway Enrichment Analysis (LIPEA)

Pathway name	Pathway lipids	Converted lipids (number)	Converted lipids (percentage)	*P*-value	Benjamin correction
Glycerophospholipid metabolism	26	7	63.64	0.0000	0.0000
Ferroptosis	11	3	27.27	0.0010	0.0043
Glycosylphosphatidylinositol—anchor biosynthesis	3	2	18.18	0.0011	0.0043
Autophagy—other	3	2	18.18	0.0011	0.0043
Autophagy—animal	4	2	18.18	0.0023	0.0068
Choline metabolism in cancer	5	2	18.18	0.0037	0.0093
Retrograde endocannabinoid signaling	8	2	18.18	0.0101	0.0217
Pathogenic Escherichia coli infection	1	1	9.09	0.0206	0.0386

### Network pharmacology analysis of parthenolide against PTC

Based on the OMIM Database, GeneCards Database, and DrugBank database, a total of 297 target genes were related to PTC. There were 238 target proteins of parthenolide based on the PharmMapper server, SEA search server, STITCH, and SwissTargetPrediction. When these selected genes were intersected, 14 potential targets of parthenolide against PTC were obtained. Besides, the 6 differential lipid metabolites regulated by parthenolide were introduced into MetScape in Cytoscape 3.8.0 software, and a total of 51 lipid metabolite targets were obtained.

**Fig. 5:**
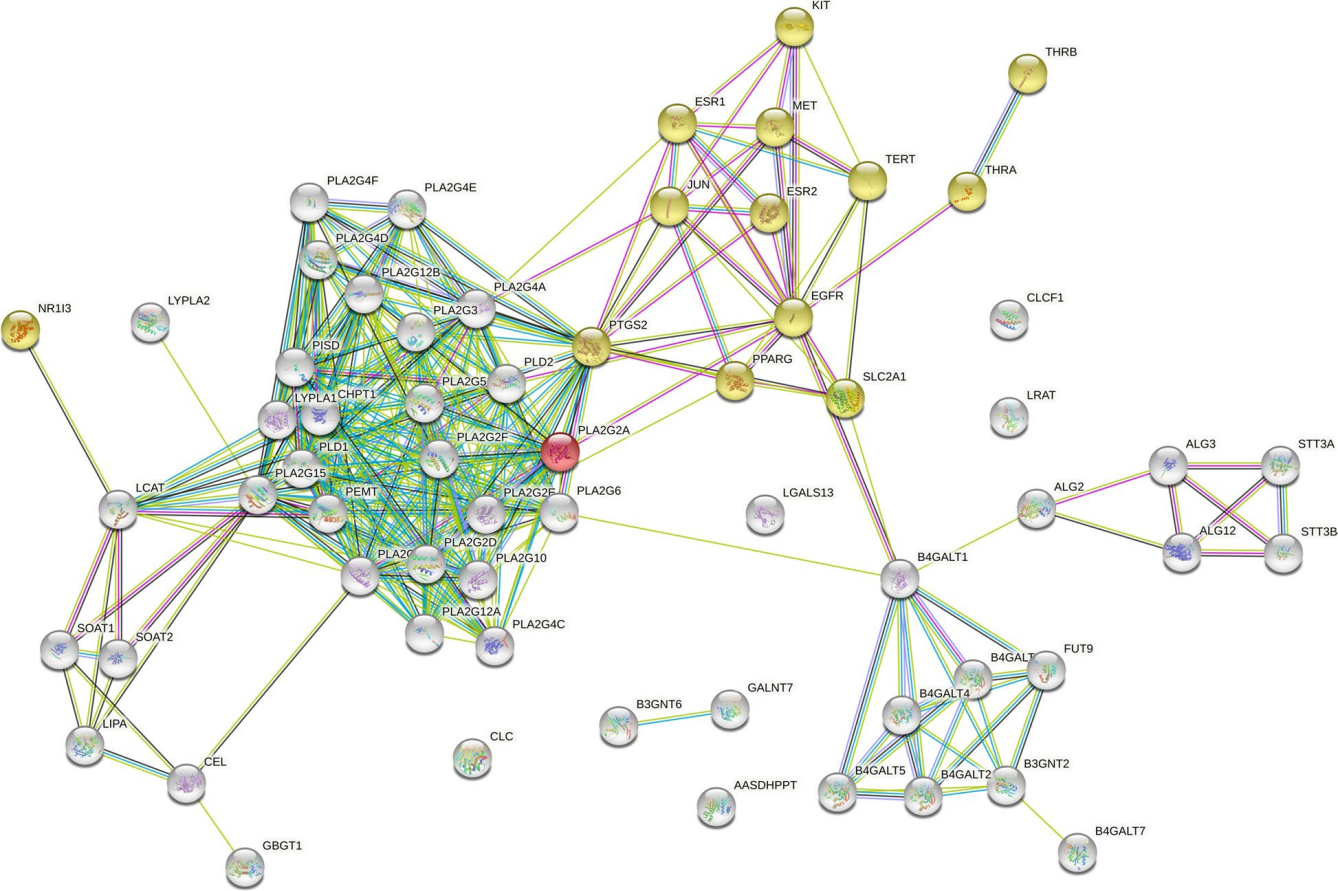
The protein–protein interaction networks by STRING. White nodes: lipid metabolite targets. Yellow nodes: potential parthenolide targets; Red node (PLA2G2A): both lipid metabolite and potential parthenolide targets

**Fig. 6:**
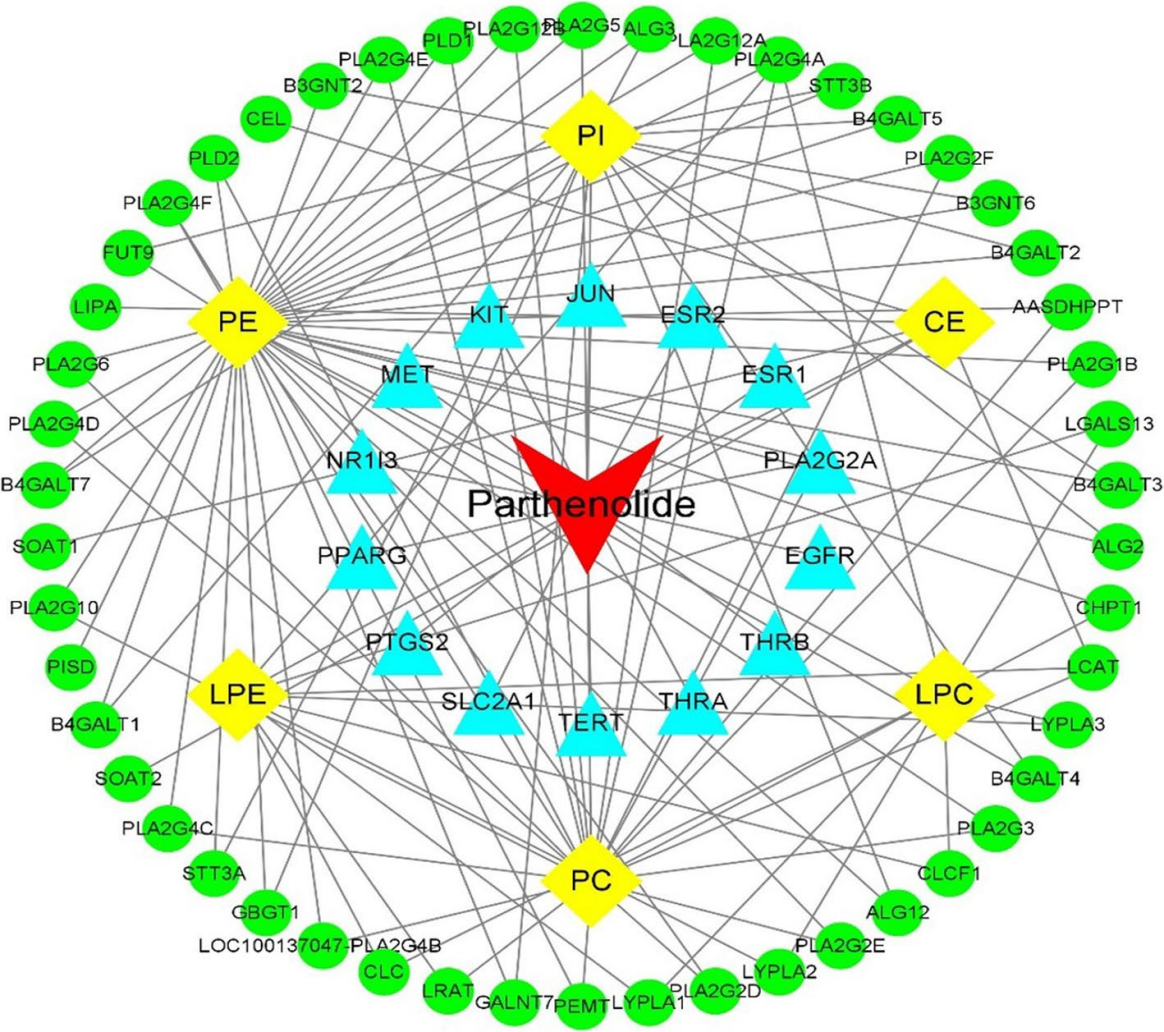
The compound-target-metabolite network of parthenolide treated papillary thyroid carcinoma cells. (Red: Compound, Blue: predicted-targets, Green: lipid metabolite targets, Yellow: lipid metabolites)

Subsequently, the 51 lipid metabolite targets and 14 potential targets of parthenolide against PTC were linked by using STRING 11.5 to establish the PPI network (Fig. 5). Finally, the compound-targets-metabolites network was constructed consisted of 72 nodes (parthenolide, 65 relevant target genes, and 6 lipid metabolites) and 120 edges (Fig. 6). PLA2G4A, LCAT, LRAT, and PLA2G2A were selected as the key targets in this network based on parameters including degree, betweenness, closeness, and stress in this network (Table 3).

**Table 3:** The top 8 nodes based on degree, betweenness, closeness and stress

Rank	Gene Name	Degree	Gene Name	Betweenness	Gene Name	Closeness	Gene Name	Stress
1	PLA2G4A	4	PLA2G4A	243.89251	PLA2G2A	34.83333	PLA2G4A	1842
2	LCAT	3	LCAT	138.16667	PLA2G4A	33.5	LCAT	614
3	PLA2G2A	3	LRAT	33.16667	PLA2G12A	30.66667	PLA2G2A	294
4	LRAT	2	PLA2G2A	4.10973	PLA2G2E	30.66667	LRAT	108
5	PLA2G2E	2	PLA2G12A	4.10973	PLA2G2D	30.66667	PLA2G2E	108
6	PLA2G2D	2	PLA2G2E	4.10973	PLA2G2F	30.66667	PLA2G2D	108
7	PLA2G2F	2	PLA2G2D	4.10973	LCAT	29.58333	PLA2G2F	108
8	PEMT	2	PLA2G2F	4.10973	LRAT	27.91667	PEMT	108

### Molecular docking analysis of parthenolide binding to predicted targets

**Fig. 7:**
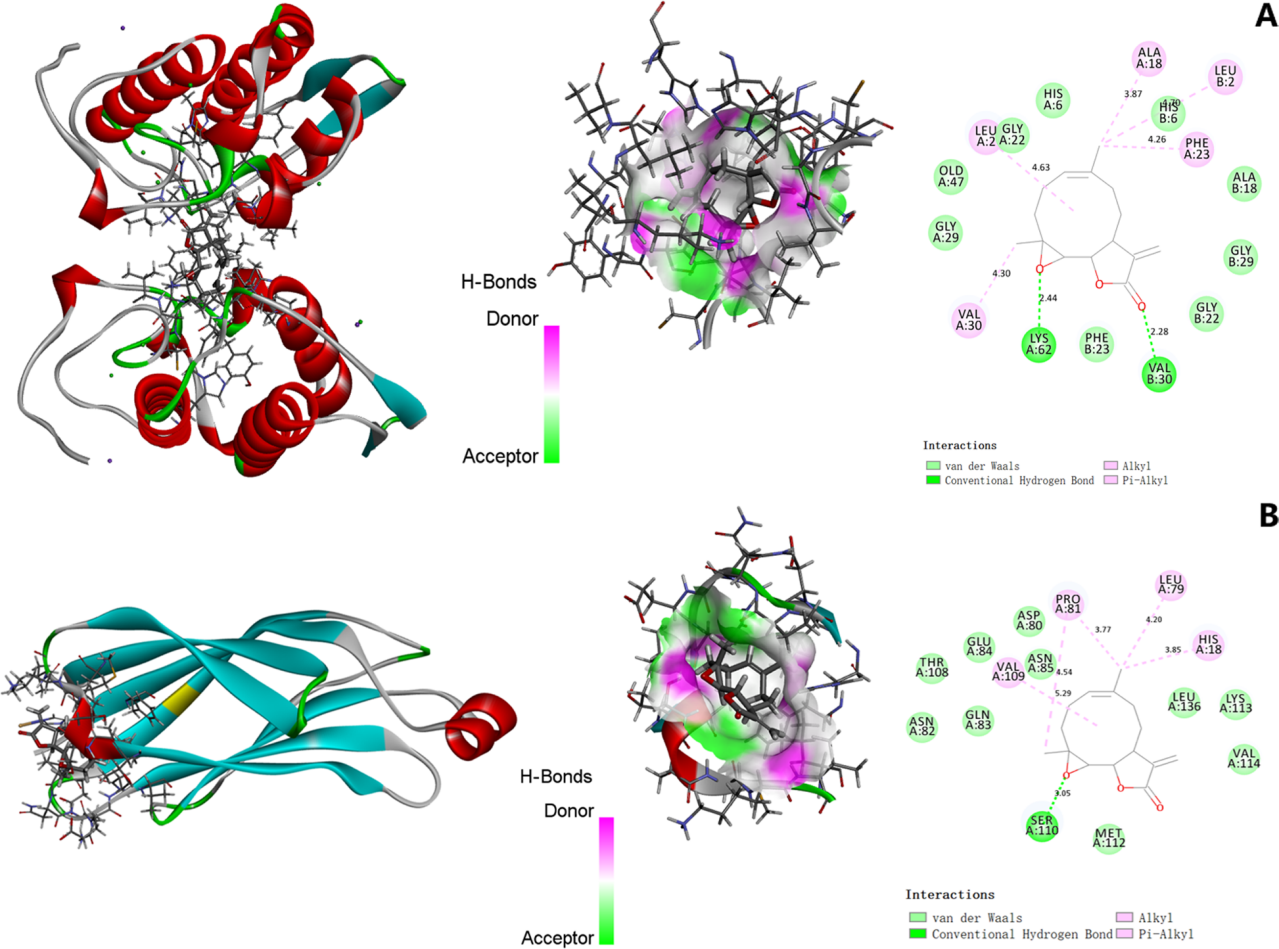
The binding mode of parthenolide and PLA2G2A (**A**) and PLA2G4A (**B**)

We performed molecular docking studies to further explore the interaction between parthenolide and predicted hub targets by using LibDock. The results proved that parthenolide could be docked into PLA2G2A and PLA2G4A, but not LCAT or LRAT. The docking analysis of PLA2G2A showed that parthenolide made hydrogen-bonding interactions with LYS A:62 and VAL B:30 residues at the active site. The van der Waals interactions included HIS A:6, GLY A:22, OLD A:47, GLY A:29, PHE B:23, GLY B:22, GLY B:29, ALA B:18, and HIS B:6 residues. The binding energy was 80.2222 between PLA2G2A and parthenolide (Fig. 7A). The docking analysis of PLA2G4A showed that parthenolide made hydrogen-bonding interactions with SER A:110 residue. The van der Waals interactions included ASP A:80, ASN A:85, GLU A:84, GLN A:83, ASN A:82, THR A:108, LEU A:136, LYS A:113, VAL A:114 and MET A:112 residues. The binding energy of parthenolide and PLA2G4A was 75.6353 (Fig. 7B). These docking analysis results presented the high affinities between parthenolide and the key targets, PLA2G2A and PLA2G4A.

## Discussion

For the first time, to our knowledge, this study investigated the lipid profile and lipid changes of PTC cells treated with parthenolide. We successfully identified 34 lipid classes and 1736 lipid species from PTC cells utilyzing LC–MS/MS. Several specific lipid species were found to have changed significantly in PTC cells treated by parthenolide, while different lipid classes were found in PTC cells treated by parthenolide, but without significance. The results of this lipidomics study were then further supplemented by network pharmacology to expand the mechanism of lipid metabolites of parthenolide against PTC. Through combining network pharmacology and lipidomics, four key targets (PLA2G4A, LCAT, LRAT, and PLA2G2A) were identified, and PLA2G2A and PLA2G4A were further confirmed to be able to bind with parthenolide through molecular docking.

A previous study demonstrated that parthenolide could promote apoptosis of PTC cells [5]. Metabolomics analysis revealed that the most important metabolic pathways affected by parthenolide treatment were amino acid metabolism and glycerophospholipid metabolism [5]. We have previously demonstrated that parthenolide led to proteomic differences in BCPAP cells [13]. Furthermore, previous studies have suggested that lipid metabolism is involved in the PTC progression [25, 26]. However, the precise lipid profile and lipid changes of PTC cells treated by parthenolide have not been fully elucidated. In this study, we further provide evidence that most of the top ten significant lipid species, such as PC, PE, LPC, PI and LPE, belong to glycerphospholipid, which may be involved in the mechanism of parthenolide in the treatment of PTC. This data was further supported by the results of an analysis of LIPEA, which showed a high level of glycerphospholipid metabolism and a significant correlation with the lipids identified in our study.

Former studies have shown that abnormal PC distribution may alter the microenvironment of the cellular lipid membrane, resulting in the variation of membrane fluidity and function [27, 28]. As a component of cell membrane, PC is increased in rapidly growing cancer cells. Abnormal distributions of PC have been observed in areas of cancer, including lung, colorectal, breast, oral, and gastric cancer [29, 30].

In recent years, the lipid composition of thyroid cancer patients has undergone considerable changes, which may play a key role in the pathogenesis of the disease. [31]. Ishikawa et al. conducted a tissue lipidomic study in seven PTC patients compared to non-cancerous tissues using imaging mass spectrometry [15]. They demonstrated that PC (16:0/18:1), PC (16:0/18:2) and sphingomyelin (SM) (d18:0/16:1) were elevated significantly in PTC tissues [15]. Wojakowska et al. also observed that multiple PC (32:0, 32:1, 34:1 and 36:3) and SM (34:1 and 36:1) concentrations in three PTC patients were significantly higher compared to the normal tissue [32]. Guo et al. highlighted that PC (34:1) in both tissue and serum could effectively distinguish between malignant thyroid cancer patients and healthy individuals [33]. Therefore, PC may serve as important lipid class associated with the pathogenesis of thyroid cancer. Besides, PE (16:1p, 18:0p, 36:1, 38:3, and 38:6) and LPE (16:0, 18:1, and 18:2) were found to be markedly high in the plasma of thyroid cancer patients when compared with healthy controls [34]. Benesch et al. demonstrated that thyroid cancer cell division can be stimulated by LPC [35]. Previously, no studies examining the association of thyroid cancer with PI, CerG3 or ChE had been conducted.

In our research, the significant difference of lipid class was not found from PTC cells treated by parthenolide, but several lipid species were changed significantly in PTC cells, such as increased levels of LPE (18:0), LPC (28:0), PC (12:0e/16:0) and PC (18:0/20:4), as well as decreased levels of PE (16:1/17:0), PC (34:1) and PC (16:0p/18:0). One of the mechanisms of parthenolide for treating thyroid cancer may be achieved by lowering the levels of PC (34:1) and PC (16:0), which have been found to be diagnostic markers for thyroid cancer [15, 32, 33].

The two key targets, PLA2G2A and PLA2G4A, which were discovered through network pharmacology and lipidomics and were subsequently confirmed via molecular docking, may play a crucial role in the treatment of parthenolide against PTC. As family members of phospholipase A2 (PLA2), PLA2G2A and PLA2G4A have a variety of biological functions, including involvement in cell signaling and inflammatory response [36, 37]. Furthermore, PLA2G2A and PLA2G4A have been implicated in the pathogenesis of various cancers, such as gastric cancer, colorectal cancer, and prostrate cancer [38–41]. Studies have also demonstrated that the expression of the PLA2G2A gene in mice can be suppressed by thyroid hormone [42, 43]. Nontheless, no studies have examined the association of the two key genes and the pathogenesis of PTC.

This study has some limitations. First, due to limited time and funds, the roles of PLA2G2A and PLA2G4A in the parthenolide-induced antitumor effect have not yet been validated through more experiments. Second, the sample size is relatively small, which may be related to the unstable results of PLA and insignificant difference in lipid classes between two groups. As we found altered lipid profile and changes of PTC cells treated with parthenolide, as well as the key targets from network pharmacology and molecular docking, it suggests that further research should use a larger sample size and more in vitro experiments.

## Conclusion

This study is the first of its kind to report the lipid profile and changes of PTC cells following treatment with parthenolide. We observed alteration in lipid species, such as PC (34:1), and PC (16:0p/18:0). To better elucidate the mechanisms by which this occurs, network pharmacology and molecular docking were employed to identify two key targets (PLA2G4A and PLA2G2A) that are able to bind parthenolide. This research provides novel insights into the underlying mechanisms of PTC, and further exploration is warranted.

## Supplementary Information

**Additional file 1.** A. UHPLC-Obitrap MS BPC of quality control samples shows high precision. B. Pearson correlation analysis of QC samples shows good reproducibility. C. Principal component analysis (PCA) analysis shows a good experimental repeatability.

**Additional file 2.** Lipid profiling of papillary thyroid carcinoma cell between two groups.

**Additional file 3.** Lipid class analysis between two groups.

**Additional file 4.** The correlation analysis between the significant lipid species.

## Data Availability

All data in this study are included in this article and its supplementary information files.

## References

[1] Sung H, Ferlay J, Siegel RL (2021). Global cancer statistics 2020: GLOBOCAN estimates of incidence and mortality worldwide for 36 cancers in 185 countries. CA Cancer J Clin.

[2] Wang X, Lu X, Geng Z, Yang G, Shi Y (2017). LncRNA PTCSC3/miR-574-5p governs cell proliferation and migration of papillary thyroid carcinoma via Wnt/β-Catenin signaling. J Cell Biochem.

[3] Sipos JA, Mazzaferri EL (2010). Thyroid cancer epidemiology and prognostic variables. Clin Oncol (R Coll Radiol).

[4] Gao W, Li L, Zhang X (2020). Nanomagnetic liposome-encapsulated parthenolide and indocyanine green for targeting and chemo-photothermal antitumor therapy. Nanomedicine (Lond).

[5] Yuan L, Wang Z, Zhang D, Wang J (2020). Metabonomic study of the intervention effects of Parthenolide on anti-thyroid cancer activity. J Chromatogr B Analyt Technol Biomed Life Sci.

[6] Talib WH, Al Kury LT (2018). Parthenolide inhibits tumor-promoting effects of nicotine in lung cancer by inducing P53 - dependent apoptosis and inhibiting VEGF expression. Biomed Pharmacother.

[7] Ge W, Hao X, Han F (2019). Synthesis and structure-activity relationship studies of parthenolide derivatives as potential anti-triple negative breast cancer agents. Eur J Med Chem.

[8] Li X, Kong L, Yang Q (2020). Parthenolide inhibits ubiquitin-specific peptidase 7 (USP7), Wnt signaling, and colorectal cancer cell growth. J Biol Chem.

[9] Liu M, Xiao C, Sun M, Tan M, Hu L, Yu Q (2018). Parthenolide Inhibits STAT3 Signaling by Covalently Targeting Janus Kinases. Molecules.

[10] Kim SL, Park YR, Lee ST, Kim SW (2017). Parthenolide suppresses hypoxia-inducible factor-1α signaling and hypoxia induced epithelial-mesenchymal transition in colorectal cancer. Int J Oncol.

[11] Gao W, Wei S, Li Z (2020). Nano magnetic liposomes-encapsulated parthenolide and glucose oxidase for ultra-efficient synergistic antitumor therapy. Nanotechnology.

[12] Carlisi D, Lauricella M, D’Anneo A (2022). Parthenolide and Its Soluble Analogues: Multitasking Compounds with Antitumor Properties. Biomedicines.

[13] Cui M, Wang Z, Huang LT, Wang JH (2022). Parthenolide leads to proteomic differences in thyroid cancer cells and promotes apoptosis. BMC Complement Med Ther.

[14] Gschwind A, Prenzel N, Ullrich A (2002). Lysophosphatidic acid-induced squamous cell carcinoma cell proliferation and motility involves epidermal growth factor receptor signal transactivation. Cancer Res.

[15] Ishikawa S, Tateya I, Hayasaka T (2012). Increased expression of phosphatidylcholine (16:0/18:1) and (16:0/18:2) in thyroid papillary cancer. PLoS ONE.

[16] Santos CR, Schulze A (2012). Lipid metabolism in cancer. FEBS J.

[17] Antal O, Péter M, Hackler L (2015). Lipidomic analysis reveals a radiosensitizing role of gamma-linolenic acid in glioma cells. Biochim Biophys Acta.

[18] Jiang N, Zhang Z, Chen X (2021). Plasma Lipidomics Profiling Reveals Biomarkers for Papillary Thyroid Cancer Diagnosis. Front Cell Dev Biol.

[19] Cheng F, Wen Z, Feng X, Wang X, Chen Y (2020). A serum lipidomic strategy revealed potential lipid biomarkers for early-stage cervical cancer. Life Sci.

[20] Bedia C, Dalmau N, Jaumot J, Tauler R (2015). Phenotypic malignant changes and untargeted lipidomic analysis of long-term exposed prostate cancer cells to endocrine disruptors. Environ Res.

[21] Li Y, Li Y, Lu W (2018). Integrated Network Pharmacology and Metabolomics Analysis of the Therapeutic Effects of Zi Dian Fang on Immune Thrombocytopenic Purpura. Front Pharmacol.

[22] Pang HQ, Yue SJ, Tang YP (2018). Integrated Metabolomics and Network Pharmacology Approach to Explain Possible Action Mechanisms of Xin-Sheng-Hua Granule for Treating Anemia. Front Pharmacol.

[23] Zeng Q, Li L, Jin Y (2019). A network pharmacology approach to reveal the underlying mechanisms of Paeonia lactiflora Pall. On the treatment of alzheimer’s disease. Evid Based Complement Alternat Med.

[24] Ingram LM, Finnerty MC, Mansoura M, Chou CW, Cummings BS (2021). Identification of lipidomic profiles associated with drug-resistant prostate cancer cells. Lipids Health Dis.

[25] Lu J, Zhang Y, Sun M (2021). Multi-omics analysis of fatty acid metabolism in thyroid carcinoma. Front Oncol.

[26] Wen S, Luo Y, Wu W (2021). Identification of lipid metabolism-related genes as prognostic indicators in papillary thyroid cancer. Acta Biochim Biophys Sin (Shanghai).

[27] Wang R, Zhao H, Zhang X, Zhao X, Song Z, Ouyang J (2019). Metabolic discrimination of breast cancer subtypes at the single-cell level by multiple microextraction coupled with mass spectrometry. Anal Chem.

[28] Guo S, Wang Y, Zhou D, Li Z (2014). Significantly increased monounsaturated lipids relative to polyunsaturated lipids in six types of cancer microenvironment are observed by mass spectrometry imaging. Sci Rep.

[29] Guo Y, Ren J, Li X (2017). Simultaneous quantification of serum multi-phospholipids as potential biomarkers for differentiating different pathophysiological states of lung, stomach, intestine, and pancreas. J Cancer.

[30] Uchiyama Y, Hayasaka T, Masaki N (2014). Imaging mass spectrometry distinguished the cancer and stromal regions of oral squamous cell carcinoma by visualizing phosphatidylcholine (16:0/16:1) and phosphatidylcholine (18:1/20:4). Anal Bioanal Chem.

[31] Jiang N, Zhang G, Pan L (2017). Potential plasma lipid biomarkers in early-stage breast cancer. Biotechnol Lett.

[32] Wojakowska A, Cole LM, Chekan M (2018). Discrimination of papillary thyroid cancer from non-cancerous thyroid tissue based on lipid profiling by mass spectrometry imaging. Endokrynol Pol.

[33] Guo S, Qiu L, Wang Y (2014). Tissue imaging and serum lipidomic profiling for screening potential biomarkers of thyroid tumors by matrix-assisted laser desorption/ionization-Fourier transform ion cyclotron resonance mass spectrometry. Anal Bioanal Chem.

[34] Lee GB, Lee JC, Moon MH (2019). Plasma lipid profile comparison of five different cancers by nanoflow ultrahigh performance liquid chromatography-tandem mass spectrometry. Anal Chim Acta.

[35] Benesch MG, Ko YM, Tang X (2015). Autotaxin is an inflammatory mediator and therapeutic target in thyroid cancer. Endocr Relat Cancer.

[36] Foldbjerg R, Dang DA, Autrup H (2011). Cytotoxicity and genotoxicity of silver nanoparticles in the human lung cancer cell line, A549. Arch Toxicol.

[37] Kasurinen S, Jalava PI, Happo MS (2017). Particulate emissions from the combustion of birch, beech, and spruce logs cause different cytotoxic responses in A549 cells. Environ Toxicol.

[38] Zhao R, Lv Y, Feng T (2022). ATF6α promotes prostate cancer progression by enhancing PLA2G4A-mediated arachidonic acid metabolism and protecting tumor cells against ferroptosis. Prostate.

[39] Zhan Y, Zheng L, Liu J (2021). PLA2G4A promotes right-sided colorectal cancer progression by inducing CD39+γδ Treg polarization. JCI Insight.

[40] Hatori S, Sakamaki K, Yokohori T (2021). Clinical Significance of PLA2G2A Expression in Gastric Cancer Patients who Receive Gastrectomy and Adjuvant S-1. Anticancer Res.

[41] Ozturk K, Onal MS, Efiloglu O, Nikerel E, Yildirim A, Telci D (2020). Association of 5'UTR polymorphism of secretory phospholipase A2 group IIA (PLA2G2A) gene with prostate cancer metastasis. Gene.

[42] Sharma P, Levesque T, Boilard E, Park EA (2014). Thyroid hormone status regulates the expression of secretory phospholipases. Biochem Biophys Res Commun.

[43] Sharma P, Thakran S, Deng X, Elam MB, Park EA (2013). Nuclear corepressors mediate the repression of phospholipase A2 group IIa gene transcription by thyroid hormone. J Biol Chem.

