# Supplementary material for: Untargeted lipidomic analysis and network pharmacology for parthenolide treated papillary thyroid carcinoma cells
Source: BMC Complement Med Ther. 2023 Apr 24;23:130. doi: 10.1186/s12906-023-03944-7 (PMC10123985; doi:10.1186/s12906-023-03944-7)

A. UHPLC-Obitrap MS BPC of quality control samples shows high precision. B. Pearson correlation analysis of QC samples shows good reproducibility. C. Principal component analysis (PCA) analysis shows a good experimental repeatability.


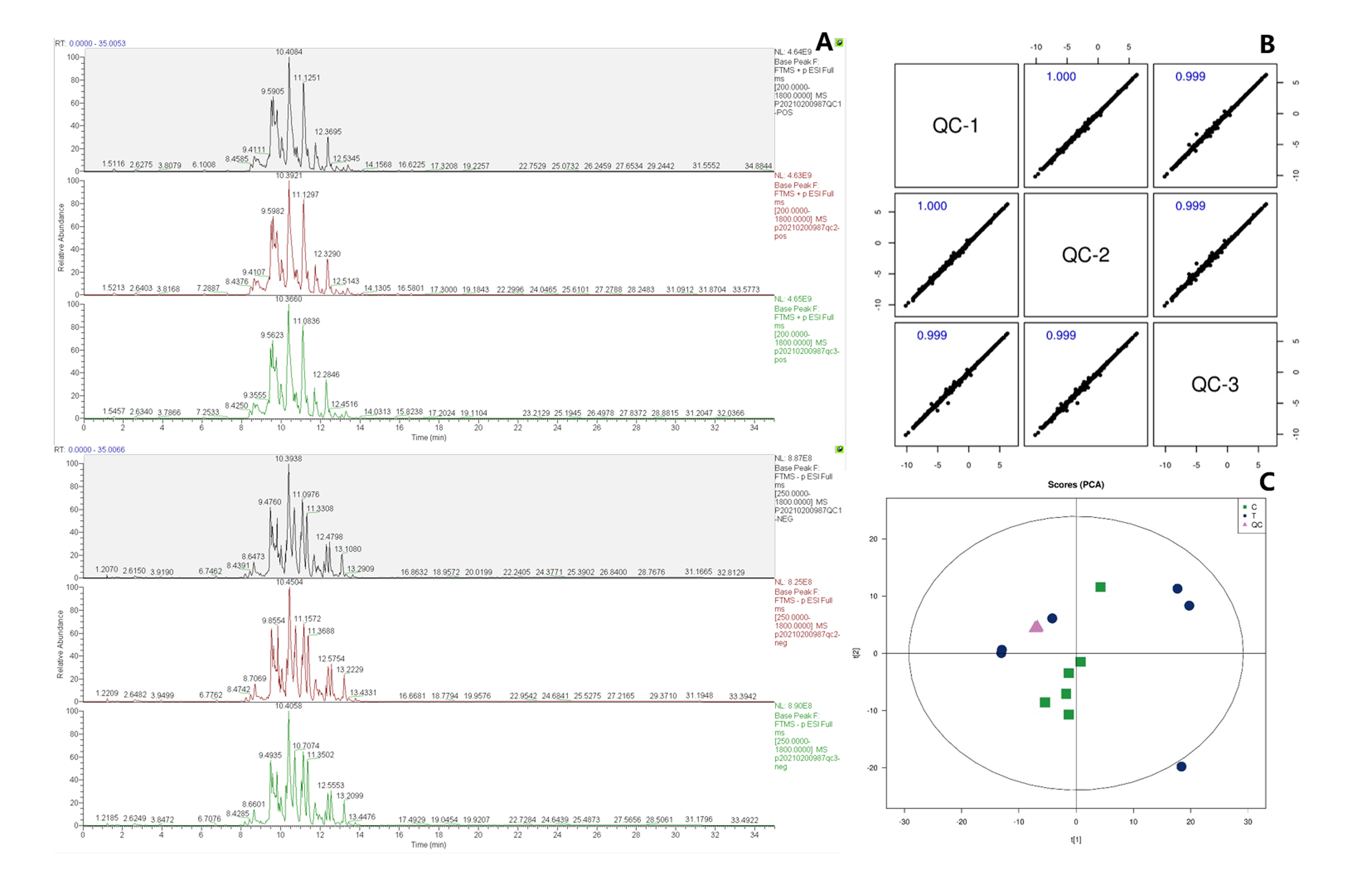

Supplement: Supplementary file 1 — Additional file 1. A. UHPLC-Obitrap MS BPC of quality control samples shows high precision. B. Pearson correlation analysis of QC samples shows good reproducibility. C. Principal component analysis (PCA) analysis shows a good experimental repeatability. [file 12906_2023_3944_MOESM1_ESM.docx]
