# Supplementary material for: Untargeted lipidomic analysis and network pharmacology for parthenolide treated papillary thyroid carcinoma cells
Source: BMC Complement Med Ther. 2023 Apr 24;23:130. doi: 10.1186/s12906-023-03944-7 (PMC10123985; doi:10.1186/s12906-023-03944-7)
Supplement: Supplementary file 2 — Additional file 2. Lipid profiling of papillary thyroid carcinoma cell between two groups. [file 12906_2023_3944_MOESM2_ESM.docx]

Additional file 2. Lipid profiling of papillary thyroid carcinoma cell between two groups.

**name DIFF LipidIon LipidGroup Class FattyAcid FA1 FA2 FA3 FA4 IonFormula CalMz RT-(min) QCRSD Fold Change P-value VIP C-1 C-2 C-3 C-4 C-5 C-6 T-1 T-2 T-3 T-4 T-5 T-6 QC-1 QC-2 QC-3**

1035POS Y CerG3(d18:1/24:1)+H CerG3(d42:2)+H CerG3 (d18:1/24:1) (d18:1) (24:1) C60 H112 O18 N1 1134.787395 11.45426343 0.029675113 1.507215828 0.034293551 1.248035478 50465382.49 92362327.87 79753755.77 95552473.77 114015950.1 98990071.82 58027050.58 135905379.8 132551475.9 154861963.3 169864325.3 149332362.4 153058323.4 153160176.8 161115568.2

1067NEG Y LPE(18:0)-H LPE(18:0)-H LPE (18:0) (18:0) C23 H47 O7 N1 P1 480.3095655 3.931845012 0.00388221 1.39823785 0.042449412 1.079678919 42193386.08 32445632.52 51242011.11 31211701.23 36670335.04 40535640.29 40535841.37 79783971.57 48460507.91 43304485.27 52562780.49 62957732.67 65307999.49 65309436.61 64870554

1168NEG Y PE(16:1/17:0)-H PE(33:1)-H PE (16:1/17:0) (16:1) (17:0) C38 H73 O8 N1 P1 702.5079305 10.29159545 0.244715961 0.751478432 0.001300086 3.050148552 190090402.3 150894634.3 155379874.2 188366288.6 179714145.6 189709144.6 105573969.2 134186458.3 129327133 125462333.8 147212595.3 150411873.2 81981512.59 130330489.1 130502394.3

1174NEG Y PC(12:0e/16:0)+HCOO PC(28:0e)+HCOO PC (12:0e/16:0) (12:0e) (16:0) C37 H75 O9 N1 P1 708.5184955 9.15502392 0.011770096 2.497934985 0.017274628 1.236084897 8590733.613 6846819.473 8318107.674 8038915.841 5989473.413 7507291.125 6958897.625 19967773.96 22192811.47 13250451.42 15454462.7 35310428.36 17336180.1 16984322.62 16990758.61

1488NEG Y PC(18:0/20:4)+HCOO PC(38:4)+HCOO PC (18:0/20:4) (18:0) (20:4) C47 H85 O10 N1 P1 854.5916605 10.4638329 0.012116754 1.488193715 0.000975384 1.749521577 138663222.2 124300633.8 136932174.8 104936329.3 136566041.9 103724901.7 212181160.9 173282646.1 187515357.6 194141161.2 206417118.9 135350372.6 172588112 169016994.8 169015030.4

1579NEG Y PI(19:0/20:4)-H PI(39:4)-H PI (19:0/20:4) (19:0) (20:4) C48 H84 O13 N0 P1 899.5655065 9.793263528 0.025019596 1.509611489 0.035162266 1.041105651 9910138.061 14539294.37 23487061 21209120.85 20270078.67 26330130.64 22219460.15 41713359.1 19530788.94 30628779.05 31624077.75 29014760.06 22002616.5 22038960.55 22988531.44

187POS Y LPC(28:0)+H LPC(28:0)+H LPC (28:0) (28:0) C36 H75 O7 N1 P1 664.5275685 9.124719663 0.009003326 2.34138841 0.019201846 2.103261038 106452359 96361953.57 108084504.7 108384879 85920807.71 98117538.07 78864955.01 250823180.8 313300703.6 166453030.1 188608610.5 414560756.9 243368525 247199399.3 243400031.2

279POS Y ChE(22:6)+NH4 ChE(22:6)+NH4 ChE (22:6) (22:6) C49 H80 O2 N1 714.6183555 15.11702945 0.005692952 1.920327123 0.021625068 1.219279286 55201625.89 19452176.44 54133450.2 29925339.82 93673692.3 64616888.64 54731067.27 154526728.5 129032575.3 89537833.77 91546735.26 89374851.75 127860773.8 129129344.9 127868022.8

407POS Y PC(34:1)+H PC(34:1)+H PC (34:1) (34:1) C42 H83 O8 N1 P1 760.5850835 11.29350012 0.053210358 0.516480553 0.024864161 2.369579257 379631149.2 299864315.3 589297793.1 200003021.6 632910451.4 641554114.1 168173444.7 212223667.1 144378484.6 331662868.2 330210641.8 230191771.6 308098526.5 338702139.6 310643847.1

430POS Y PC(16:0p/18:0)+Na PC(34:0p)+Na PC (16:0p/18:0) (16:0p) (18:0) C42 H84 O7 N1 P1 Na1 768.5877635 10.90135728 0.003168816 0.793529376 0.043173512 1.087524049 152583788.5 147065758.5 147987512 158578484.3 167049343.4 199322309.5 131171705.5 128918086 123966258.8 107300047.5 97882332.27 182538080.7 140865647.9 141639724.6 140864677

1001POS TG(25:1/18:1/18:1)+NH4 TG(61:3)+NH4 TG (25:1/18:1/18:1) (25:1) (18:1) (18:1) C64 H122 O6 N1 1000.926665 18.206 0.001933488 0.919372564 0.828148613 0.030627159 1356416.441 1565210.832 1778507.761 3698206.691 1227716.8 1145813.857 297481.9429 3763300.469 2514631.283 1084981.24 869332.8633 1373636.134 2608525.03 2608505.319 2599789.278

1002POS CerG2(d18:1/26:0)+H CerG2(d44:1)+H CerG2 (d18:1/26:0) (d18:1) (26:0) C56 H108 O13 N1 1002.78152 12.74234019 0.004257259 1.017882111 0.949536766 0.478122562 70246861.76 62380262.53 69873139.67 57802429.82 42491332.45 72943923.33 14861359.76 98983768.64 95238106.21 29348909.41 37774819.32 106249973.8 100392601.3 100662695.2 101232871.9

1003POS TG(18:1/22:4/22:4)+NH4 TG(62:9)+NH4 TG (18:1/22:4/22:4) (18:1) (22:4) (22:4) C65 H112 O6 N1 1002.848415 15.36259264 0.002988905 1.614122425 0.2239049 0.320791127 5008759.83 3039406.301 4924398.178 6908180.598 4278356.846 1517492.428 1119792.557 10339728.32 6825981.602 11587845.72 10059677.03 1512141.239 6695472.102 6695506.718 6730211.425

1004POS TG(27:1/16:0/18:1)+NH4 TG(61:2)+NH4 TG (27:1/16:0/18:1) (27:1) (16:0) (18:1) C64 H124 O6 N1 1002.942315 19.02325237 0.062531503 0.92533033 0.800553557 0.058839871 2126398.593 2261996.245 2070263.557 4067147.584 1713421.374 1470410.32 808865.9788 3790795.929 3586034.206 920354.2085 1332315.821 2247577.409 3553434.986 3558775.396 3955656.995

1005POS TG(18:1/22:1/22:6)+NH4 TG(62:8)+NH4 TG (18:1/22:1/22:6) (18:1) (22:1) (22:6) C65 H114 O6 N1 1004.864065 15.68985084 0.005871037 1.210743837 0.649036277 0.130024483 4573256.017 6587026.675 4160902.114 12376319.95 7837323.552 2572941.919 770152.4464 16413940.59 13354048.05 7060855.763 5601804.778 2937946.32 11179425.61 11177313.74 11292413.69

1006POS TG(18:1/22:1/22:6)+NH4 TG(62:8)+NH4 TG (18:1/22:1/22:6) (18:1) (22:1) (22:6) C65 H114 O6 N1 1004.864065 16.032 0.038775124 1.284698109 0.574897497 0.118109798 1391864.148 3135457.411 1689723.707 5090314.909 2894716.491 739238.0196 240212.1518 7113821.909 4706586.978 3066168.511 3361012.087 707277.0795 5018939.463 4688692.73 5017752.083

1007POS TG(26:0/17:0/18:1)+NH4 TG(61:1)+NH4 TG (26:0/17:0/18:1) (26:0) (17:0) (18:1) C64 H126 O6 N1 1004.957965 19.9341962 0.022117647 0.963376116 0.919727492 0.07450444 1502727.903 2037117.722 1584653.735 3208580.67 735804.7359 1342607.115 701028.7333 2579374.997 3622874.266 620733.3568 611381.4842 1894789.776 2775692.983 2887261.362 2783541.229

1008POS TG(16:0/22:6/24:1)+NH4 TG(62:7)+NH4 TG (16:0/22:6/24:1) (16:0) (22:6) (24:1) C65 H116 O6 N1 1006.879715 16.69022622 0.009673584 1.265167628 0.643328895 0.102784062 1924985.01 3469815.491 2098710.687 6274104.346 2364124.997 1210089.797 387461.4299 9347148.111 6464663.178 2410240.706 2129730.547 1201078.371 5647458.238 5742850.239 5647925.308

1009POS TG(16:0/22:6/24:0)+NH4 TG(62:6)+NH4 TG (16:0/22:6/24:0) (16:0) (22:6) (24:0) C65 H118 O6 N1 1008.895365 17.48485807 0.006755643 1.288727873 0.626547621 0.089273863 1297258.303 2557453.415 1475365.617 3933737.085 1141931.676 1057652.39 454946.4336 6255189.458 4836931.16 1146840.967 1039942.504 1039350.631 3819328.946 3868787.663 3830500.917

100POS PC(19:1)+H PC(19:1)+H PC (19:1) (19:1) C27 H53 O8 N1 P1 550.3503335 2.745993742 0.076260751 0.883275211 0.723269506 0.137580227 1729616.711 8272716.935 3266714.569 5051853.692 9222930.548 5116814.722 5185241.561 2134572.454 10894768.65 3725076.391 4174806.966 2733874.003 1308896.818 1308780.553 1489682.273

1010POS TG(16:0/22:5/24:0)+NH4 TG(62:5)+NH4 TG (16:0/22:5/24:0) (16:0) (22:5) (24:0) C65 H120 O6 N1 1010.911015 17.75402537 0.005615851 1.152249559 0.726313653 0.07190907 1974512.996 2762757.777 2267799.37 5322050.976 1747181.242 1574740.259 788638.1775 6568150.268 5211489.129 2032278.323 1839450.097 1591596.464 4481052.33 4529367.013 4492326.806

1011POS TG(26:1/18:1/18:2)+NH4 TG(62:4)+NH4 TG (26:1/18:1/18:2) (26:1) (18:1) (18:2) C65 H122 O6 N1 1012.926665 17.90194278 0.025489854 0.920012755 0.81835767 0.030241049 3138917.507 3847547.849 3294349.456 8725583.695 2753133.355 2489843.992 925925.2754 6860119.012 6533009.822 2838116.329 2588969.678 2563594.961 7541074.869 7526215.681 7206109.818

1012POS TG(26:1/18:1/18:1)+NH4 TG(62:3)+NH4 TG (26:1/18:1/18:1) (26:1) (18:1) (18:1) C65 H124 O6 N1 1014.942315 18.60293452 0.005816079 0.753728501 0.385565766 0.16249874 7309317.354 10405822.05 6418690.881 15299867.1 4897964.945 6073335.398 1682825.773 12094313.83 10377659.44 4416961.538 3863873.68 5556049.126 10865618.73 10975699.89 10975932.28

1013POS TG(26:0/18:1/18:1)+NH4 TG(62:2)+NH4 TG (26:0/18:1/18:1) (26:0) (18:1) (18:1) C65 H126 O6 N1 1016.957965 19.51157478 0.076308062 0.95045951 0.884764076 0.172502194 13643236.73 10771481.24 14656137.28 21522082.05 4945583.674 11994954 2292664.646 25611320.01 18882515.26 6979213.794 4977977.57 14948737.37 21069525.54 21059114.52 18397755.35

1014POS TG(26:0/18:0/18:1)+NH4 TG(62:1)+NH4 TG (26:0/18:0/18:1) (26:0) (18:0) (18:1) C65 H128 O6 N1 1018.973615 20.35163168 0.0040038 0.892103216 0.735402026 0.177920757 7813332.962 7682229.07 7807773.804 12004118.78 2875451.186 6226614.033 2495817.821 11113416.47 12946244.63 2603443.599 2251059.811 8207893.122 10718248.28 10792806.16 10718361.46

1015POS SM(d56:3+pO)+H SM(d56:3+pO)+H SM (d56:3+pO) (d56:3+pO) C61 H120 O7 N2 P1 1023.882767 14.309 0.01260504 0.895281126 0.719928744 0.087770994 2781922.777 1060897.079 3200362.527 2523581.141 3272690.042 4126701.096 1341218.359 2310116.615 2510735.181 1588432.436 1608100.661 5830874.802 4515521.947 4615584.05 4615542.945

1016POS CerG3(d18:1/16:0)+H CerG3(d34:1)+H CerG3 (d18:1/16:0) (d18:1) (16:0) C52 H98 O18 N1 1024.677845 9.260392042 0.001448584 1.407892394 0.087365947 1.391594383 88590555.34 99295243.11 119627887.1 152326606.5 174786642.1 143096664.5 71802643.62 212668475.3 156123369.1 215163574.1 230515246.6 208677830.7 207713484.1 207712401.3 207192222.8

1017POS TG(20:0/22:6/22:6)+NH4 TG(64:12)+NH4 TG (20:0/22:6/22:6) (20:0) (22:6) (22:6) C67 H110 O6 N1 1024.832765 14.65242636 0.010143676 1.152317544 0.814378047 0.085387843 1713981.716 7504185.555 2350170.229 11217761 7280566.869 1221014.434 225119.1281 18286304.68 9330548.186 870325.4676 6034448.35 1306596.54 10424633.48 10612532.52 10607270.74

1018POS TG(18:1/22:6/24:1)+NH4 TG(64:8)+NH4 TG (18:1/22:6/24:1) (18:1) (22:6) (24:1) C67 H118 O6 N1 1032.895365 16.65795883 0.007897545 1.202981971 0.717448052 0.084783859 1644500.492 3270085.131 1719097.163 7019472.124 2800929.081 1174038.484 242301.861 8805927.75 6150752.579 2682430.877 2261652.756 1063247.689 5412542.844 5487391.454 5487132.123

1019POS TG(26:1/16:0/22:6)+NH4 TG(64:7)+NH4 TG (26:1/16:0/22:6) (26:1) (16:0) (22:6) C67 H120 O6 N1 1034.911015 17.43532565 0.004451165 1.190749755 0.762589912 0.109142118 1980214.491 3390205.396 3354402.134 11300916.46 3412903.314 1965602.111 793484.2498 15308696.35 5923413.402 3209173.41 2874651.326 2140678.477 8789347.598 8865046.659 8845639.306

101POS LPC(20:1)+H LPC(20:1)+H LPC (20:1) (20:1) C28 H57 O7 N1 P1 550.3867185 3.68385557 0.006016723 1.479559117 0.068890714 0.303186262 8151949.787 6589135.736 10484768.83 4444745.007 12021635.82 7579860.917 7501981.728 17581442.34 15129855.36 8587823.5 10947244.86 13152631.2 14805094.93 14960074.87 14960846.71

1020POS TG(18:1/22:5/24:1)+NH4 TG(64:7)+NH4 TG (18:1/22:5/24:1) (18:1) (22:5) (24:1) C67 H120 O6 N1 1034.911015 16.89135453 0.021373905 1.075015629 0.862456737 0.041584115 2452066.59 2884256.313 1582643.043 5505557.383 2412841.875 1061727.191 416305.0821 6160833.072 5065484.535 2287457.463 1908819.84 1252872.816 4421801.615 4588778.567 4590492.263

1021POS TG(18:1/22:5/24:0)+NH4 TG(64:6)+NH4 TG (18:1/22:5/24:0) (18:1) (22:5) (24:0) C67 H122 O6 N1 1036.926665 17.65003625 0.102599537 0.980426004 0.965028047 0.030480683 1923187.199 6691942.205 2159773.864 6415539.116 2125314.133 1672210.947 611500.9124 7701829.447 6332816.429 2306316.267 2182985.787 1441700.238 6323723.152 7530360.279 6344323.049

1022POS TG(26:0/16:0/22:6)+NH4 TG(64:6)+NH4 TG (26:0/16:0/22:6) (26:0) (16:0) (22:6) C67 H122 O6 N1 1036.926665 18.23267925 0.005228946 1.200244051 0.733408806 0.089743298 1908132.853 4210272.898 1922530.966 5221512.855 1335892.672 1371146.902 465132.0307 8016275.612 6927523.603 1283902.481 1124011.226 1350439.393 4610814.966 4659160.794 4632082.62

1023POS TG(26:0/16:0/22:5)+NH4 TG(64:5)+NH4 TG (26:0/16:0/22:5) (26:0) (16:0) (22:5) C67 H124 O6 N1 1038.942315 18.52178172 0.005451969 0.712271619 0.492118544 0.119076961 2267148.237 6947854.86 2408338.204 10592620.34 3354013.613 1784081.769 679481.6795 7191690.426 6524143.983 1694836.418 1323546.421 2069819.567 4706347.712 4750972.015 4706430.08

1024POS TG(28:1/18:1/18:1)+NH4 TG(64:3)+NH4 TG (28:1/18:1/18:1) (28:1) (18:1) (18:1) C67 H128 O6 N1 1042.973615 19.354 0.010006458 1.020204693 0.96295386 0.054474669 2524156.528 1916283.876 1639390.038 3767399.436 1755677.803 1196621.424 327373.6172 5639609.873 3497208.708 1057379.182 832085.1979 1704483.086 2915111.331 2864478.953 2914289.598

1025POS TG(28:1/18:0/18:1)+NH4 TG(64:2)+NH4 TG (28:1/18:0/18:1) (28:1) (18:0) (18:1) C67 H130 O6 N1 1044.989265 20.2280188 0.037079326 0.859961148 0.574753898 0.092735684 2101622.316 2660587.99 2253759.46 2529720.394 1257158.782 1273606.409 556210.3893 2504819.295 2796292.082 973243.2786 904805.0853 2649912.279 2841789.636 2841232.073 3027993.544

1026POS TG(28:0/18:0/18:1)+NH4 TG(64:1)+NH4 TG (28:0/18:0/18:1) (28:0) (18:0) (18:1) C67 H132 O6 N1 1047.004915 21.11216975 0.022042372 0.984790344 0.969643233 0.076264898 1338828.701 1311531.502 1756628.312 1981317.379 666594.9063 1224417.151 264181.7474 2845224.665 2866415.619 377489.7039 365211.5505 1434869.087 2195916.926 2280474.269 2195227.514

1027POS TG(26:1/18:1/22:6)+NH4 TG(66:8)+NH4 TG (26:1/18:1/22:6) (26:1) (18:1) (22:6) C69 H122 O6 N1 1060.926665 17.3385628 0.011332469 1.113105521 0.833396639 0.048983939 1153763.278 2603032.803 1298276.348 4830938.474 1742571.676 731122.7797 154723.8192 5461192.111 4573250.369 1416598.616 1352642.745 799248.6106 3751632.418 3752153.009 3826020.039

1028POS TG(26:0/18:1/22:6)+NH4 TG(66:7)+NH4 TG (26:0/18:1/22:6) (26:0) (18:1) (22:6) C69 H124 O6 N1 1062.942315 18.20430425 0.010044441 1.176068437 0.763448863 0.072505663 1437933.636 4511072.39 1582449.987 5765470.715 1688985.733 1126222.444 338440.1543 7968125.079 6429984.808 1535640.896 1484018.156 1192764.227 4904253.722 4819921.705 4819898.888

1029POS TG(26:0/18:1/22:5)+NH4 TG(66:6)+NH4 TG (26:0/18:1/22:5) (26:0) (18:1) (22:5) C69 H126 O6 N1 1064.957965 18.4822505 0.007444862 1.141600069 0.785823885 0.056570459 1124432.46 3410409.443 1551949.134 4584058.642 1437188.781 859086.146 385165.1622 5629559.14 4971976.328 1347174.96 1405083.556 1064311.203 3493992.41 3539224.922 3539647.496

102POS LPC(20:0)+H LPC(20:0)+H LPC (20:0) (20:0) C28 H59 O7 N1 P1 552.4023685 5.268555135 0.008289541 1.071441301 0.692190958 0.049713176 2745397.417 2603592.656 3037674.864 1786169.639 1904611.068 2386380.186 2406773.371 3300485.445 3394491.88 1395845.403 1622179.888 3377364.384 3428649.744 3478456.827 3478250.002

1030POS TG(26:0/18:1/24:0)+NH4 TG(68:1)+NH4 TG (26:0/18:1/24:0) (26:0) (18:1) (24:0) C71 H140 O6 N1 1103.067515 22.333 0.006272393 1.005824598 0.989775551 0.072901705 994584.519 1439739.841 1124584.813 1959415.924 318939.3889 962792.9197 189004.261 2375983.977 2527612.364 233028.3433 204018.3504 1310017.708 1832326.422 1852115.841 1831956.576

1031POS CerG3(d18:1/22:0)+H CerG3(d40:1)+H CerG3 (d18:1/22:0) (d18:1) (22:0) C58 H110 O18 N1 1108.771745 11.49439885 0.007509018 1.378675815 0.054366933 0.862370302 37646117.95 41525150.99 55409211.25 59403075.53 60236772.39 59751758.71 35189939.29 84077291.64 63419216.14 81039220.53 84861396.82 84278658.19 80996426.99 80996196.15 82054336.56

1032POS CerG3(d18:1/23:0)+H CerG3(d41:1)+H CerG3 (d18:1/23:0) (d18:1) (23:0) C59 H112 O18 N1 1122.787395 11.78384231 0.00029847 1.47500062 0.047420267 0.598277431 13413005.88 15322031.64 19282097.48 24618932.98 24385130.01 21490726.74 14604957.86 30353642.65 23297970.74 37190436.24 38926227.94 30431927.07 30500362.13 30516132.72 30516137.66

1033POS GM3(d32:1)+H GM3(d32:1)+H GM3 (d32:1) (d32:1) C55 H101 O21 N2 1125.689139 7.105420473 0.008702956 0.863417096 0.585790504 0.079155084 2790209.499 1470484.844 5160620.706 4784927.983 2759767.203 3688240.507 2038344.938 2952563.102 2775864.904 2574448.901 1618126.297 5873885.049 3437995.373 3490152.472 3438135.878

1034POS CerG3(d18:2/24:1)+H CerG3(d42:3)+H CerG3 (d18:2/24:1) (d18:2) (24:1) C60 H110 O18 N1 1132.771745 10.913 0.011754751 1.501990792 0.043550364 0.46795727 7334965.154 10356637.58 10502532.13 12880422.77 17149582.92 15210797.26 8521923.563 20727241.78 15851495.52 22480068.9 22895336.85 19822533.76 18972968.57 19362272.41 18973710.45

1036POS CerG3(d18:1/24:0)+H CerG3(d42:1)+H CerG3 (d18:1/24:0) (d18:1) (24:0) C60 H114 O18 N1 1136.803045 12.04714589 0.00186698 1.362554153 0.10120724 1.811569596 153248599.6 193194119.2 242551394.3 305005810.9 290297340.3 245510561.5 133206654.8 400845969.2 288774572.1 373740405.9 396368616.2 355254372.6 382994935.7 384234956.3 382995318.9

1037POS GM3(d33:1)+H GM3(d33:1)+H GM3 (d33:1) (d33:1) C56 H103 O21 N2 1139.704789 7.677787768 0.013704615 0.9819523 0.937310027 0.023633268 2004978.55 1079545.65 3097412.374 2998895.907 2169665.823 2345478.006 1465678.857 2459838.586 1950470.926 2114930.974 1335414.582 4122461.508 2659885.729 2659901.555 2597251.158

1038POS CerG3(d18:1/25:0)+H CerG3(d43:1)+H CerG3 (d18:1/25:0) (d18:1) (25:0) C61 H116 O18 N1 1150.818695 12.30213565 0.048232969 1.28103181 0.200320871 0.354050831 9124476.116 10872931.76 10901012.82 18293168.42 14549786.02 13843998.64 6456855.829 22442630.33 15336667 16969796.64 20595175.38 17588206.65 19912309.61 19916428.41 18295761.53

1039POS GM3(d34:2)+H GM3(d34:2)+H GM3 (d34:2) (d34:2) C57 H103 O21 N2 1151.704789 7.282093932 0.00801145 0.839169862 0.53798796 0.08357441 2582613.991 892627.1572 4370537.207 2813637.174 1881259.897 3197825.235 2177607.324 2202602.603 1929564.809 1312279.393 1276324.399 4308896.893 2562940.329 2599183.62 2598478.636

103POS PE(21:3)+Na PE(21:3)+Na PE (21:3) (21:3) C26 H46 O8 N1 P1 Na1 554.2853285 2.155785993 0.010841878 0.836403005 0.558311816 0.169849364 2556577.011 8141916.352 4676166.123 6145037.427 8895075.9 6259718.772 7583915.862 2051346.976 10708223.83 3237276.612 3618235.801 3475655.891 1385408.814 1359599.705 1385500.7

1040POS GM3(d34:1)+H GM3(d34:1)+H GM3 (d34:1) (d34:1) C57 H105 O21 N2 1153.720439 8.209889223 0.002988164 1.058998252 0.793515009 0.203434933 97382455.59 62995393.29 168603075.4 169350775.4 117920076 135777781.4 69488399.12 148318240.2 119620378.1 134625077 99307694.73 225038197.1 146296592.6 147054401.4 147058371.7

1041POS CerG3(d18:1/26:1)+H CerG3(d44:2)+H CerG3 (d18:1/26:1) (d18:1) (26:1) C62 H116 O18 N1 1162.818695 11.99460958 0.009693979 1.114216479 0.534158664 0.230904508 16198838.58 20707289.9 23963060.01 32235452.7 25922217.73 31902520.85 12406916.2 34850927.77 26955480.64 27369278.93 28187504.59 38397893.98 35191543.17 35787110.83 35791098.01

1042POS CerG3(d18:1/26:0)+H CerG3(d44:1)+H CerG3 (d18:1/26:0) (d18:1) (26:0) C62 H118 O18 N1 1164.834345 12.55339099 0.005122961 1.086167024 0.668637369 0.165713048 23107040.42 23285348.66 29466178.11 41849800.2 30506574.45 33371218.15 13376525.86 45833679.84 35378134.6 26371462.13 29396613.06 46876483.56 46504596.93 46094723.42 46094032.74

1043POS GM3(d35:1)+H GM3(d35:1)+H GM3 (d35:1) (d35:1) C58 H107 O21 N2 1167.736089 8.717 0.014058998 1.204341746 0.35312754 0.113805627 3324254.649 1943124.322 6396542.158 6238101.552 4266229.86 5328904.26 3588649.686 5236391.999 5869892.562 5908294.528 4340911.874 8171833.175 5315722.605 5447023.564 5447577.198

1044POS GM3(d34:1+O)+H GM3(d34:1+O)+H GM3 (d34:1+O) (d34:1+O) C57 H105 O22 N2 1169.715354 7.971094218 0.008499464 1.396356085 0.19218189 0.238045427 3158999.399 2253697.946 5358375.895 6529072.242 6227389.825 4977881.893 2946302.516 6354066.084 3965750.534 9809668.458 7142355.983 9585569.175 6378111.726 6284694.963 6378152.551

1045POS CerG3GNAc1(d34:1)+H CerG3GNAc1(d34:1)+H CerG3GNAc1 (d34:1) (d34:1) C60 H111 O23 N2 1227.757219 9.011370485 0.007996349 1.123143415 0.570253859 0.206300925 31173257.23 23388352.24 40902982.31 44792877.87 35335999.7 50320024.51 21559919.79 48534267.03 39227524.02 35237684.77 37719639.62 71454217.78 47951962.33 48603809.81 47922601.97

1046POS GM3(d41:1)+H GM3(d41:1)+H GM3 (d41:1) (d41:1) C64 H119 O21 N2 1251.829989 10.92279289 0.008581764 1.222811537 0.262946438 0.324427042 18623951.06 12173296.39 29530670.33 34682782.96 23007646.01 27267869.75 20302422.31 32531890.66 23956082.11 30638597.66 27802685.71 42425983.23 27621058.09 27612531.38 28029269.79

1047POS GM3(d42:2)+H GM3(d42:2)+H GM3 (d42:2) (d42:2) C65 H119 O21 N2 1263.829989 10.58054757 0.008485816 1.081784352 0.596645848 0.301768806 67998789.01 56760420.99 103906052.3 114664444.1 97083995.89 114455032.9 77876113.02 105043850.2 81124283.48 98614467.23 94686241.9 142903359.2 93767998.66 92402166.07 92404201.49

1048POS GM3(d42:1)+H GM3(d42:1)+H GM3 (d42:1) (d42:1) C65 H121 O21 N2 1265.845639 11.20914069 0.008383535 1.09493024 0.598060949 0.548696874 223784520.1 161601360 364715930.5 405465455.6 275595650.9 354666599.7 236117005.9 389806620.1 265126864.3 317532391.3 282413598.6 464362261 323697684.7 323773351.7 319057749.5

1049POS GM3(d43:2)+H GM3(d43:2)+H GM3 (d43:2) (d43:2) C66 H121 O21 N2 1277.845639 10.87 0.006547131 1.110051644 0.573878179 0.138183752 5207605.202 5487374.16 9973708.13 13094132.47 8383947.447 8780921.432 7370694.045 11109520.74 5746145.581 10707477.48 8779728.041 12818798.85 9369273.381 9366944.718 9474727.012

104POS PE(21:2)+Na PE(21:2)+Na PE (21:2) (21:2) C26 H48 O8 N1 P1 Na1 556.3009785 2.354051613 0.035942791 0.931322505 0.829058877 0.077278809 1127060.431 2591374.169 2106386.016 2025903.201 2485768.508 2855528.431 4492331.514 689691.0218 3404267.339 883878.0808 1064115.683 1751742.18 531043.064 565511.9764 532415.2626

1050POS GM3(d43:1)+H GM3(d43:1)+H GM3 (d43:1) (d43:1) C66 H123 O21 N2 1279.861289 11.46739839 0.020920144 1.288407485 0.168111878 0.341345303 14125846.74 9513695.589 24152912.29 24407048.76 17756100.51 20928996.84 15594234.52 29786764.87 21271027.17 24928362.01 18744899.33 32539261.62 23364250.93 22528030.08 23364818.15

1051POS GM3(d44:2)+H GM3(d44:2)+H GM3 (d44:2) (d44:2) C67 H123 O21 N2 1291.861289 11.16513785 0.019571728 0.893914555 0.557254626 0.201333615 24036440.08 15265817.52 33065705.22 33736407.33 29329631.53 33777536.13 19487592.86 28254051.1 14596925.55 25590135.38 21205575.86 42126375.82 22625485.37 21862527.46 21889059.71

1052POS GM3(d44:1)+H GM3(d44:1)+H GM3 (d44:1) (d44:1) C67 H125 O21 N2 1293.876939 11.72001657 0.012240184 1.008613806 0.969857128 0.073951206 32041324.9 21211157.07 54942576.25 65788749.32 34008854.42 51761342.42 25007926.48 56003934.44 42850127.95 37579112.52 31040741.85 69509631.85 44941204.39 43998686.91 44004745.32

1053POS CerG3GNAc1(d40:1)+H CerG3GNAc1(d40:1)+H CerG3GNAc1 (d40:1) (d40:1) C66 H123 O23 N2 1311.851119 11.29998478 0.005066112 1.135878475 0.513242646 0.150084261 14146126.9 12191411.07 22324928.08 20798139.61 16675158.44 26410326.81 13289814.56 23828662.32 18200243.46 18466259.85 19083923.33 34969778.63 22839807.77 23039012.41 23043707.07

1054POS CerG3GNAc1(d42:2)+H CerG3GNAc1(d42:2)+H CerG3GNAc1 (d42:2) (d42:2) C68 H125 O23 N2 1337.866769 11.25612811 0.004052227 1.308206605 0.193821649 0.450891696 24312753.34 21612199.8 48886384.95 39169262.38 39115503.93 48638833.55 31247057.73 44858816.7 45132653.45 40723237.11 48964611.56 79148733.94 48716040.3 48374651.54 48715505.18

1055POS CerG3GNAc1(d42:1)+H CerG3GNAc1(d42:1)+H CerG3GNAc1 (d42:1) (d42:1) C68 H127 O23 N2 1339.882419 11.85309834 0.007296069 1.188543895 0.392633006 0.532711048 71924667.92 57880778.2 96430547.79 115499590.3 87978535.72 110460963.6 54179099.35 140986101.9 104862206.3 84539141.61 90265609.42 167189639.2 124174972 122610626.5 124171505.5

1056POS CerG3GNAc1(d44:1)+H CerG3GNAc1(d44:1)+H CerG3GNAc1 (d44:1) (d44:1) C70 H131 O23 N2 1367.913719 12.35131781 0.02540682 1.001498965 0.994986443 0.053999049 8265863.056 5792787.872 11152501.5 13776676.53 7915194.11 13941376.91 4285989.611 13684376.97 9433502.009 8259773.019 7756178.649 17515783.33 12337908.56 11801661.4 12335484.49

1057NEG FA(20:4)-H FA(20:4)-H FA (20:4) (20:4) O2 H31 C20 303.2329535 4.233731478 0.003751503 2.410198951 0.096970516 0.957388085 2422871.231 5538643.966 2407208.421 4299531.443 10978232.51 3690472.037 2576138.908 26491983.37 12010639.43 11273348.93 14598590.73 3757207.892 11708594.3 11708537.78 11784811.01

1058NEG FA(20:4)-H FA(20:4)-H FA (20:4) (20:4) O2 H31 C20 303.2329535 2.185671062 0.008530134 1.428163353 0.445931671 0.160429535 790667.1254 1351139.903 956324.2447 536265.372 1162540.606 712733.8569 105191.6113 2099478.808 1933133.912 413786.2375 353957.8521 2963161.942 1633825.344 1658079.81 1633817.143

1059NEG FA(22:6)-H FA(22:6)-H FA (22:6) (22:6) O2 H31 C22 327.2329535 3.863 0.003970813 3.153387855 0.050399572 2.17470354 5719236.577 24364298.22 5165746.739 13515580.69 46193472.89 9828309.962 3846529.223 115441774.6 51694536.56 79634825.37 38605603.94 41209664.27 40109771.04 40386999.05 40111233.15

105POS LPG(22:6)+H LPG(22:6)+H LPG (22:6) (22:6) C28 H46 O9 N0 P1 557.2873995 1.788151605 0.008323064 1.268822793 0.367152392 0.094179457 1029232.331 2521304.704 1524332.613 3763025.824 4593772.461 2593391.429 1276447.774 4146244.067 5157084.511 3387658.335 2901288.031 3464237.861 4582939.379 4649294.942 4582878.377

1060NEG FA(22:5)-H FA(22:5)-H FA (22:5) (22:5) O2 H33 C22 329.2486035 4.40727243 0.03577254 2.001509587 0.221690041 0.389672379 354318.6584 2107847.275 414563.6395 1240054.95 4713030.79 949974.9164 158082.1651 7582879.253 3811485.815 2845950.179 3989191.288 1186755.203 3854763.543 3622014.419 3628930.362

1061NEG LPE(16:1)-H LPE(16:1)-H LPE (16:1) (16:1) C21 H41 O7 N1 P1 450.2626155 2.144692605 0.011553036 1.044930203 0.856378868 0.132632683 5500324.386 3595600.025 6175938.079 7254261.96 6350572.19 2730246.717 7872194.896 4884802.995 4408452.82 5619836.441 10693425.1 7669191.004 7599862.387 7776030.013

1062NEG LPE(16:0)-H LPE(16:0)-H LPE (16:0) (16:0) C21 H43 O7 N1 P1 452.2782655 2.816812682 0.007163741 1.460484297 0.110674716 0.44038452 13612933.46 8710053.976 19619532.96 1560449.814 12661559.32 14293454.89 15680630.73 18462248.77 13847424.64 13060442.83 16418756.08 25433276.81 20250849.17 20001834.53 20001443.63

1063NEG LPE(17:1)-H LPE(17:1)-H LPE (17:1) (17:1) C22 H43 O7 N1 P1 464.2782655 2.444848013 0.070810092 1.275220537 0.235124798 0.141532185 1591956.104 875710.8599 1726268.802 1249469.494 1714743.296 1661845.865 931676.7078 1927271.911 1833031.04 1741816.274 1692747.335 3120894.757 2260384.146 1999253.202 2009235.826

1064NEG LPE(17:0)-H LPE(17:0)-H LPE (17:0) (17:0) C22 H45 O7 N1 P1 466.2939155 3.317753697 0.007169329 1.533425356 0.043004331 0.214310157 1551357.218 1311788.09 2443274.305 283333.2523 1295376.715 1477761.448 1854336.967 2376701.209 1857647.533 1693956.206 2539306.041 2501921.194 2275204.18 2247169.397 2247197.58

1065NEG LPE(18:2)-H LPE(18:2)-H LPE (18:2) (18:2) C23 H43 O7 N1 P1 476.2782655 2.295569165 0.006925188 1.143141589 0.510778091 0.202542441 3495025.091 2705209.696 4067095.762 4606882.677 2996410.484 2117126.623 5873659.94 3736048.053 2993763.881 4003481.171 5790304.159 4967761.78 5027844.647 5027813.953

1066NEG LPE(18:1)-H LPE(18:1)-H LPE (18:1) (18:1) C23 H45 O7 N1 P1 478.2939155 2.793226435 0.008162725 1.19789233 0.465885127 0.250842105 48717880.35 57059793.21 25689693.42 43850335.77 40976850.92 25965140.01 22053906.93 61823323.91 83748597.31 36720922.91 53623479.13 32230798.79 43728740.68 43727616.89 43112839.41

1068NEG LPE(20:5)-H LPE(20:5)-H LPE (20:5) (20:5) C25 H41 O7 N1 P1 498.2626155 1.906771772 0.009587373 1.056575419 0.77275516 0.128273396 3726265.731 2245568.906 3795076.268 3033076.565 3021095.2 1628310.785 3839727.972 3007649.009 2765198.306 3579058.078 5239456.312 4349735.803 4330987.113 4270535.667

1069NEG LPE(20:4)-H LPE(20:4)-H LPE (20:4) (20:4) C25 H43 O7 N1 P1 500.2782655 2.203611025 0.007780357 1.387508494 0.218916471 0.450964894 13534624.96 11285908.15 15307969.54 35203.00964 17515043.52 13245769.27 7110497.293 20795736.94 15428941.33 13348268.71 17592989.91 24131937.6 20095128.42 19826172.08 20096409.22

106POS LPC(20:0p)+Na LPC(20:0p)+Na LPC (20:0p) (20:0p) C28 H58 O6 N1 P1 Na1 558.3893985 5.93558962 0.002583128 1.403738643 0.361275142 0.349411003 11329634.45 19389103.14 12583021.23 23078466.48 27725350.31 19442905.25 1816653.661 53272364.4 41028767.08 16398281.7 17846440.75 29029882.8 40134317.36 40137270.55 40315615.83

1070NEG LPE(20:3)-H LPE(20:3)-H LPE (20:3) (20:3) C25 H45 O7 N1 P1 502.2939155 2.49885507 0.010821746 1.148547091 0.529886598 0.058482666 977642.968 837709.9087 1345941.672 959321.2079 1604359.064 1536821.353 560497.5789 2023207.05 1503760.634 845230.8396 1422914.502 1984904.269 1997737.869 1955098.665 1973673.939

1071NEG LPE(20:1)-H LPE(20:1)-H LPE (20:1) (20:1) C25 H49 O7 N1 P1 506.3252155 4.040867922 0.026299136 1.561400818 0.039292524 0.236580206 824755.136 756650.517 1033772.864 725447.4325 1290814.791 846468.658 918839.8072 2250979.186 1154523.175 1085281.411 1617255.038 1526333.601 1537144.403 1534932.852 1607059.954

1072NEG LPG(18:2)-H LPG(18:2)-H LPG (18:2) (18:2) C24 H44 O9 N0 P1 507.2728465 1.90457218 0.015209878 1.496811521 0.330290849 0.119122697 230718.5061 204664.7675 481078.2019 682819.1163 848006.3757 445477.2845 488364.5142 579098.152 623005.8137 599331.8539 1660475.175 793666.0547 816958.616 812073.042

1073NEG LPG(18:1)-H LPG(18:1)-H LPG (18:1) (18:1) C24 H46 O9 N0 P1 509.2884965 2.22642396 0.016234222 1.363644124 0.394766189 0.306819789 3186741.432 2040471.523 4520454.13 6775058.565 7066837.194 3576249.466 5163879.276 5069589.659 6004275.65 4821174.041 13966154.43 7435235.776 7230100.192 7229930.136

1074NEG LPC(14:0)+HCOO LPC(14:0)+HCOO LPC (14:0) (14:0) C23 H47 O9 N1 P1 512.2993955 1.97051791 0.013308831 1.167985893 0.664820238 0.225614481 4003788.648 2164861.186 4731257.354 4148898.283 3926307.783 2437402.031 4225785.742 5372424.944 2290026.93 2215841.465 10053716.4 5417581.658 5294593.768 5294582.269

1075NEG LPE(22:6)-H LPE(22:6)-H LPE (22:6) (22:6) C27 H43 O7 N1 P1 524.2782655 2.09984388 0.006942321 1.067737121 0.727589251 0.23433286 9778742.98 7844750.316 10406787.58 11077684.18 11221991.63 4778321.03 12738541.12 10198961.63 8362483.647 11819810.51 16588877.74 14166097.05 13996672.83 13997554.84

1076NEG LPE(22:5)-H LPE(22:5)-H LPE (22:5) (22:5) C27 H45 O7 N1 P1 526.2939155 2.26403865 0.014454319 1.122210392 0.718882952 0.162137139 3502642.965 2183827.59 3868112.004 1911786.814 2823538.544 4085849.983 2164249.266 3199879.068 3067985.426 1952514.525 2208453.373 8028384.819 4253901.352 4361382.981 4254070.007

1077NEG LPE(22:4)-H LPE(22:4)-H LPE (22:4) (22:4) C27 H47 O7 N1 P1 528.3095655 2.693465757 0.002196542 1.256688914 0.398794406 0.124041622 1346746.955 1077586.884 1577071.244 784900.7277 1581607.717 1704722.228 625590.8966 2015307.198 1995708.31 919948.85 1479747.648 3108488.961 2136147.35 2140595.705 2131216.275

1078NEG LPG(20:3)-H LPG(20:3)-H LPG (20:3) (20:3) C26 H46 O9 N0 P1 533.2884965 1.902333095 0.018483202 1.034485906 0.92066827 0.041038895 567200.4718 610897.2008 969570.9496 2456529.3 1199416.122 1359551.203 459907.669 1084487.74 1554324.418 739282.8477 1118403.237 2453787.58 1620355.234 1569822.816 1569311.794

1079NEG LSM(d20:2)+HCOO LSM(d20:2)+HCOO LSM (d20:2) (d20:2) C26 H52 O7 N2 P1 535.3517645 2.519657405 0.004892245 1.199508568 0.493709333 0.096165974 880999.7346 671529.3935 1013983.684 1032373.128 1015658.217 938554.3967 487366.6585 1568248.934 1183878.206 632827.0994 708613.9541 2080054.443 1305819.216 1294756.682 1294877.019

107POS LPC(20:4)+Na LPC(20:4)+Na LPC (20:4) (20:4) C28 H50 O7 N1 P1 Na1 566.3217135 2.106163628 0.04324619 1.27768373 0.275837306 0.075044319 1716595.762 1066244.396 1853358.463 568692.979 1885237.083 1305773.245 767127.2368 1877719.765 2388919.956 1449867.773 1729570.799 2514101.766 2125565.38 2293863.788 2292423.436

1080NEG LSM(d20:1)+HCOO LSM(d20:1)+HCOO LSM (d20:1) (d20:1) C26 H54 O7 N2 P1 537.3674145 3.28213373 0.006065264 1.18338844 0.476777169 0.114123861 1106038.671 950920.606 1318008.108 1031546.566 1147173.848 971190.9574 418618.5208 1858439.839 1853682.656 793182.1647 940138.5846 1857404.33 1668809.587 1651616.732 1651191.205

1081NEG LPC(16:1)+HCOO LPC(16:1)+HCOO LPC (16:1) (16:1) C25 H49 O9 N1 P1 538.3150455 2.05408393 0.002760792 1.395756244 0.405578539 0.4536114 17355639.12 8767290.933 21647520.51 21058.50382 20782340.09 20965386.51 9477771.899 19163875.91 23658333.42 12873805.22 12034090.38 47767070.48 25248418.72 25128262 25127876.29

1082NEG LPC(16:0)+HCOO LPC(16:0)+HCOO LPC (16:0) (16:0) C25 H51 O9 N1 P1 540.3306955 2.511602555 0.002421873 1.343979618 0.201146728 0.553096588 46654125.38 21556908.84 59668516.56 26837465.13 35840392.97 39680402.94 43892956.58 42689481.46 49831025.08 47797969.29 35718984.29 89504509.78 58740379.54 58478767.98 58514446.08

1083NEG LPC(17:1)+HCOO LPC(17:1)+HCOO LPC (17:1) (17:1) C26 H51 O9 N1 P1 552.3306955 2.302289402 0.002404137 1.296023566 0.32267185 0.194168515 4222432.551 2106575.177 3990069.72 2114439.174 3937912.601 3338158.411 2499931.887 4964899.842 4849017.131 2481900.478 2918614.331 7829726.386 5489717.187 5466871.739 5489682.053

1084NEG Cer(d18:2/14:0)+HCOO Cer(d32:2)+HCOO Cer (d18:2/14:0) (d18:2) (14:0) C33 H62 O5 N1 552.4633475 8.862298457 0.013988051 0.853386228 0.449549911 0.481192344 6661764.659 5101196.121 5692498.694 4533943.191 6874153.565 7149354.977 2667056.36 7140814.277 6052334.884 2726202.957 3482903.967 8663610.001 7585348.412 7405195.893 7403795.473

1085NEG LPC(17:0)+HCOO LPC(17:0)+HCOO LPC (17:0) (17:0) C26 H53 O9 N1 P1 554.3463455 3.100933832 0.047132396 1.196735163 0.311034419 0.049775099 1270596.57 996649.9421 1871875.931 797165.701 1726468.083 1773852.555 1316471.122 1920612.717 1928301.299 1060572.875 1589498.392 2280929.985 2120074.471 2120165.223 2298038.819

1086NEG Cer(d18:1/14:0)+HCOO Cer(d32:1)+HCOO Cer (d18:1/14:0) (d18:1) (14:0) C33 H64 O5 N1 554.4789975 9.79891572 0.009808496 0.7445486 0.072560984 2.026882471 99965306.47 92914720.61 60318206.61 58315785.52 84058672.08 61568900.76 52212484.34 52722220.98 78397208.21 38381434.17 47627905.24 71022879.25 72135454.22 70919242.82 70928171.52

1087NEG LPG(22:6)-H LPG(22:6)-H LPG (22:6) (22:6) C28 H44 O9 N0 P1 555.2728465 1.786406543 0.006872106 1.582784342 0.154189791 0.679091718 3601081.679 9370630.132 5151806.857 15202065.68 8514266.982 3836345.997 17333771.9 20155592.46 12738018.65 10893916.31 14510508.55 15628889.26 15445892.81 15442797.23

1088NEG LPG(22:5)-H LPG(22:5)-H LPG (22:5) (22:5) C28 H46 O9 N0 P1 557.2884965 1.883990822 0.040997702 1.596714104 0.215083139 0.177387549 90091.32883 666705.1568 212939.8134 1450034.33 1622057.808 1121339.412 72188.1511 1466980.671 1922604.013 1293428.176 1441481.809 2047420.108 1705726.353 1589276.645 1590894.316

1089NEG LPC(18:2)+HCOO LPC(18:2)+HCOO LPC (18:2) (18:2) C27 H51 O9 N1 P1 564.3306955 2.170859212 0.021543405 1.258469076 0.393440538 0.349141606 6121974.173 3737500.309 7362284.385 8782943.632 6546273.343 4006282.506 8942190.747 9902719.324 5791573.565 5928318.786 14586190.87 9603091.877 9249555.253 9603907.627

108POS Cer(d18:1/18:0)+H Cer(d36:1)+H Cer (d18:1/18:0) (d18:1) (18:0) C36 H72 O3 N1 566.5506705 11.3574301 0.00471143 0.960914673 0.791847963 0.219102929 44761823.4 26317060.11 41047269.33 28997286.1 39167924.29 42137654.91 30035602.65 40001160.69 34759040.96 25186019.7 28973138.73 54780344.41 45692147.69 45760554.24 45358949

1090NEG LPE(24:0)-H LPE(24:0)-H LPE (24:0) (24:0) C29 H59 O7 N1 P1 564.4034655 8.527980172 0.033990049 1.496098915 0.087214615 0.438097237 2659335.223 1405120.96 3254163.341 2646309.506 3599610.169 2755682.991 2063277.028 5613295.466 2368530.138 4070733.42 5767482.966 4533347.695 5178799.979 5182429.116 4881505.145

1091NEG LPC(18:1)+HCOO LPC(18:1)+HCOO LPC (18:1) (18:1) C27 H53 O9 N1 P1 566.3463455 2.632472585 0.030409762 1.273270714 0.399714564 0.900591564 98324204.83 68777976.56 119865876.9 54926974.26 154037187 112919404.7 50445042.38 144073403.5 161124276.7 85898380.7 92059726.35 241632112.9 169116237.9 169206119.8 160405321.1

1092NEG LPC(18:0)+HCOO LPC(18:0)+HCOO LPC (18:0) (18:0) C27 H55 O9 N1 P1 568.3619955 3.713827693 0.000800689 1.288094601 0.098443229 0.404159467 28181225.33 34215701.78 31110029.33 15817698.65 24484202.23 22452428.26 24399002.55 41524425.28 36487923.5 24301155.11 33936840.07 40629971.79 40216683.53 40263043.46 40278649.83

1093NEG Cer(d17:1/16:0)+HCOO Cer(d33:1)+HCOO Cer (d17:1/16:0) (d17:1) (16:0) C34 H66 O5 N1 568.4946475 10.21066437 0.006882326 1.029326308 0.768635956 0.4016975 31560035.23 25842173.39 24788708.5 22655679.74 33723416.13 28824310.8 23078464.9 34167872.92 29843098.08 21976955.32 30010300.53 33226689.47 30842412.91 31212125.97 31213900.2

1094NEG LPI(16:0)-H LPI(16:0)-H LPI (16:0) (16:0) C25 H48 O12 N0 P1 571.2888915 2.072530092 0.038657821 1.046551415 0.919973198 0.041320045 728141.7921 64048.31826 1964110.589 635780.773 874493.2033 1573760.329 408765.8934 230899.4852 1157871.817 502982.3226 1483947.869 930346.5974 886133.7999 862320.9288

1095NEG Cer(d18:2/16:0)+HCOO Cer(d34:2)+HCOO Cer (d18:2/16:0) (d18:2) (16:0) C35 H66 O5 N1 580.4946475 9.852658243 0.01263553 0.85784057 0.335114855 1.622242735 126921771.2 97361368.26 68491817.59 57645338.05 127138239.8 79414470.53 91742834.59 69438678.05 87069573.32 62651934.22 76318890.07 90572130.12 85143133.15 85164052.81 83303559.22

1096NEG Cer(d18:1/16:0)+HCOO Cer(d34:1)+HCOO Cer (d18:1/16:0) (d18:1) (16:0) C35 H68 O5 N1 582.5102975 10.62558913 0.005489954 1.097368513 0.586852415 1.261403485 527370744.4 399486454.4 444045080.8 330239543.7 501733723.5 470258567.2 287586766.3 641279064.8 558552842.2 310987534.6 425022040.9 709984959.6 572370687.8 566957555.3 566967194.3

1097NEG Cer(d18:0/16:0)+HCOO Cer(d34:0)+HCOO Cer (d18:0/16:0) (d18:0) (16:0) C35 H70 O5 N1 584.5259475 10.92186723 0.008496162 0.902508111 0.675171873 1.763664393 80686349.81 195285848.5 68448102.31 102248159.3 147709909.7 139677069.4 88415243.51 110148169.1 106231598 70614758.64 83041959.84 204039258.2 137958036.9 135946240.2 137975087.7

1098NEG LPC(20:5)+HCOO LPC(20:5)+HCOO LPC (20:5) (20:5) C29 H49 O9 N1 P1 586.3150455 1.835147117 0.006921864 1.197243551 0.432893504 0.126484451 1061739.78 568281.1997 1101146.131 960804.5736 787653.0918 412012.1405 994959.0612 1178803.041 968047.6521 1118184.048 1763836.304 1203214.439 1202655.275 1217406.601

1099NEG LPC(20:4)+HCOO LPC(20:4)+HCOO LPC (20:4) (20:4) C29 H51 O9 N1 P1 588.3306955 2.103805035 0.009242027 1.30159216 0.230204762 0.315743148 3306594.681 1983407.092 3874951.099 3890413.934 2805713.16 1879301.299 4356616.11 4711687.789 3411142.853 4046711.663 6368129.093 4933717.763 4855851.927 4855299.791

109POS LPC(22:6)+H LPC(22:6)+H LPC (22:6) (22:6) C30 H51 O7 N1 P1 568.3397685 2.047354017 0.008965591 1.147052111 0.618593587 0.184760309 13372058.34 9858494.68 15598770.34 5240808.245 12096427.2 10758407.25 6824404.246 15583054.85 20869964.43 6067020.594 7082451.967 20339527.48 17112784.07 17381371.17 17381240.72

10POS So(d18:1+hO)+H So(d18:1+hO)+H So (d18:1+hO) (d18:1+hO) C18 H38 O3 N1 316.2846205 1.944 0.008120723 0.986512313 0.940407086 0.017694076 850811.5573 481972.4453 1032073.212 524083.9344 867721.0072 1036283.543 783141.5381 788075.4829 599273.7672 532931.3726 792151.359 1232726.43 1242847.639 1225515.696 1225541.851

1100NEG LPC(20:3)+HCOO LPC(20:3)+HCOO LPC (20:3) (20:3) C29 H53 O9 N1 P1 590.3463455 2.34574364 0.02401379 1.216974746 0.432316732 0.10085315 1470866.876 860049.5781 1036454.342 735432.7037 1207730.663 1581620.932 678847.3961 1838327.549 1606646.432 902785.65 951439.7767 2409531.892 1645365.278 1649070.857 1716619.025

1101NEG LPC(20:1)+HCOO LPC(20:1)+HCOO LPC (20:1) (20:1) C29 H57 O9 N1 P1 594.3776455 3.821437315 0.033388406 1.521597106 0.073368091 0.292300361 1922824.09 1598456.523 2186561.046 1151989.644 2797085.638 1633140.493 1496799.717 4145980.501 3185142.783 1868031.047 2639933.879 3843030.787 3245877.879 3235775.306 3057157.068

1102NEG LPC(20:0)+HCOO LPC(20:0)+HCOO LPC (20:0) (20:0) C29 H59 O9 N1 P1 596.3932955 5.254542197 0.007242171 1.274337024 0.321181729 0.084253836 748911.4262 670201.0417 814417.3379 330426.4487 451344.6589 521548.4575 577861.1505 1011231.212 1008528.59 338181.3397 461631.9032 1109703.905 854112.4667 854179.8393 843476.6784

1103NEG Cer(d18:1/17:0)+HCOO Cer(d35:1)+HCOO Cer (d18:1/17:0) (d18:1) (17:0) C36 H70 O5 N1 596.5259475 11.02578603 0.007058096 1.222564965 0.169752697 0.435624537 30192235.93 25685904.46 24085220.49 20108775.22 30214494.6 25253967.25 19551906.48 43809929.59 34424536.14 23648559.49 35116089.97 33607464.04 38161317.26 37697678.28 38163313.28

1104NEG LPI(18:1)-H LPI(18:1)-H LPI (18:1) (18:1) C27 H50 O12 N0 P1 597.3045415 2.148922313 0.003364641 1.129027035 0.68063329 0.103227463 2350161.269 978144.8572 6334433.748 3227098.55 4140964.964 3883380.218 5561920.475 2440102.535 1787069.398 4599719.085 2798853.215 6425013.988 3701575.144 3723227.057 3701651.204

1105NEG LPI(18:0)-H LPI(18:0)-H LPI (18:0) (18:0) C27 H52 O12 N0 P1 599.3201915 2.854659287 0.000904754 1.600298845 0.269803291 0.371549953 4310859.357 2335429.146 11421559.33 4393821.19 7594331.571 7406817.947 23160644.19 5547230.727 3723525.619 10578061.19 11549437.53 5392805.988 6818378.187 6830002.712 6827803.748

1106NEG Cer(d18:1/18:0)+HCOO Cer(d36:1)+HCOO Cer (d18:1/18:0) (d18:1) (18:0) C37 H72 O5 N1 610.5415975 11.38819534 0.00623607 1.057180929 0.719340839 0.431189523 49382560.04 32690051.94 43350417.59 29481550.85 47513049.48 49974107.81 48945518.19 42053785.85 40281522.42 30554746.85 36514869.3 68473289.13 48918388.57 48387679.77 48909734.02

1107NEG LPC(22:6)+HCOO LPC(22:6)+HCOO LPC (22:6) (22:6) C31 H51 O9 N1 P1 612.3306955 2.03866981 0.007640715 1.19063879 0.557077928 0.2459612 3459834.509 2172140.03 3679258.127 2741505.48 2600429.273 1558005.571 3964231.355 5520092.815 1628635.898 2305717.036 5959272.753 4236859.473 4181332.525 4181225.221

1108NEG Cer(d18:0/18:0)+HCOO Cer(d36:0)+HCOO Cer (d18:0/18:0) (d18:0) (18:0) C37 H74 O5 N1 612.5572475 11.6293027 0.005746165 0.75486502 0.360990952 1.159551999 13940490.13 42840447.99 16562320.86 24666467.55 37113365.77 33501102.2 16333937.4 24067166.01 26947666.05 2403996.538 15579116.03 41956623.94 32362106.47 32044471.77 32039859.32

1109NEG LPC(22:5)+HCOO LPC(22:5)+HCOO LPC (22:5) (22:5) C31 H53 O9 N1 P1 614.3463455 2.16667411 0.013005605 1.096468475 0.766836942 0.168654926 2658876.981 1872548.393 2728373.159 2600937.706 2200906.813 952521.6959 2660549.573 3920271.689 1596886.678 1491533.868 5248490.128 3287202.815 3213751.453 3287295.817

110POS Cer(d18:0/18:0)+H Cer(d36:0)+H Cer (d18:0/18:0) (d18:0) (18:0) C36 H74 O3 N1 568.5663205 11.59733865 0.011460423 0.800711438 0.384207656 0.337637884 8689679.992 26704605.27 8839694.39 16119596.27 21539179.38 22149704.22 11241331.1 16193417.61 15001918.45 8610955.249 8915887.515 23344477.47 21289518.8 21291771.65 21716075.73

1110NEG LPI(20:4)-H LPI(20:4)-H LPI (20:4) (20:4) C29 H48 O12 N0 P1 619.2888915 1.786138175 0.007918096 1.665380833 0.131216249 0.532754283 2124119.413 1316324.032 5444927.901 8058164.713 5110187.212 6601141.197 5484085.298 3551179.341 11103156.66 6322148.438 11011706.71 5968166.022 6050354.34 5968090.585

1111NEG LPI(20:3)-H LPI(20:3)-H LPI (20:3) (20:3) C29 H50 O12 N0 P1 621.3045415 1.983 0.011094777 1.466100285 0.440657808 0.053311383 342205.6069 188363.427 521844.01 40092.72321 1032229.722 881899.8386 469856.1366 669986.8815 493732.2024 340237.223 502363.7909 1931852.677 790926.2635 806028.8119 790551.5521

1112NEG PE(30:4p)-H PE(30:4p)-H PE (30:4p) (30:4p) C35 H61 O7 N1 P1 638.4191155 7.195100258 0.040092655 2.270607871 0.029810382 0.219910662 198884.2579 200449.801 226809.256 324587.2001 220718.3598 384668.389 60231.7244 618014.9559 435401.2746 660274.7753 860725.3928 898683.9851 736579.8332 687719.0081 687667.2085

1113NEG Cer(d18:1/20:0)+HCOO Cer(d38:1)+HCOO Cer (d18:1/20:0) (d18:1) (20:0) C39 H76 O5 N1 638.5728975 12.02878794 0.002498194 1.087288591 0.614865802 0.143791436 42276049.55 30370130.68 39293614.34 31780966.62 49606199.13 43871771.85 22113050.71 54289041.63 47146728.83 31591062.99 41348547.19 61415043.88 53932518.93 53932301.7 53699380.98

1114NEG Cer(d18:1/24:1)-H Cer(d42:2)-H Cer (d18:1/24:1) (d18:1) (24:1) C42 H80 O3 N1 646.6143675 12.57752666 0.080682214 0.915639626 0.519283303 0.330147383 11951593.97 11111021.25 10835213.7 8636246.484 9655291.34 8111173.199 5265336.575 12522846.06 10048909.36 6955989.606 11381029.31 9039452.921 11576092.66 13240693.22 11521238.93

1115NEG Cer(d18:1/24:0)-H Cer(d42:1)-H Cer (d18:1/24:0) (d18:1) (24:0) C42 H82 O3 N1 648.6300175 13.19030872 0.010225645 1.13883875 0.483766894 0.322845242 20359731.56 16414924.46 16715128 18632539.47 18557319.56 19808700.79 8940853.349 27983701.41 28636375.07 13750198 18629925.85 27887353.71 26215416.88 26215959.6 25754098.7

1116NEG LPC(24:1)+HCOO LPC(24:1)+HCOO LPC (24:1) (24:1) C33 H65 O9 N1 P1 650.4402455 7.005818583 0.00865068 1.127182079 0.600134526 0.098073591 1049548.263 774807.4258 1129368.762 1111395.066 1194664.611 1208651.168 622502.5705 1615937.741 1494116.987 635136.1216 849413.4649 2073997.457 1550801.301 1528110.66 1527491.337

1117NEG Cer(d17:1/22:0)+HCOO Cer(d39:1)+HCOO Cer (d17:1/22:0) (d17:1) (22:0) C40 H78 O5 N1 652.5885475 12.33936862 0.008479975 1.080763732 0.660974568 0.145618277 21451419.32 13917276.15 17878248.17 14499611.32 22164538.16 19110934.28 10851024.35 23674178.63 20088332.82 12523813 20463986.96 30225717.47 25019145.62 25385036.67 25012548.24

1118NEG PE(16:1/14:0)-H PE(30:1)-H PE (16:1/14:0) (16:1) (14:0) C35 H67 O8 N1 P1 660.4609805 8.943744542 0.008442211 0.949755541 0.870663295 0.631212477 16102981.52 11606861.48 19888084.67 16945617.52 12871345.44 23120643.7 5914032.661 17930378.35 19589591.6 8300611.248 7331315.918 36418251 20799769.64 20804875.8 20499651.69

1119NEG PE(16:0/14:0)-H PE(30:0)-H PE (16:0/14:0) (16:0) (14:0) C35 H69 O8 N1 P1 662.4766305 8.538534758 0.021669759 0.980714571 0.955002715 0.270456696 2399690.665 1805352.628 1956273.357 2452923.839 1622101.892 2565376.207 1132617.83 1957902.916 3335124.608 551397.7668 716965.7302 4860823.096 2949067.923 2957700.018 2844156.315

111POS LPC(22:5)+H LPC(22:5)+H LPC (22:5) (22:5) C30 H53 O7 N1 P1 570.3554185 2.177219682 0.008214017 1.101803575 0.717874017 0.143633588 9800411.235 7362634.465 10809209.79 4017445.891 9935353.179 9100170.397 4709423.655 9976022.031 15034006.67 5487467.344 5024648.175 15988207.42 12598217.54 12779301.87 12779037.56

1120NEG PE(32:5p)-H PE(32:5p)-H PE (32:5p) (32:5p) C37 H63 O7 N1 P1 664.4347655 7.619936873 0.012311856 1.642650331 0.028791047 0.221843061 978939.0789 548255.8687 969427.2206 503480.6342 317017.1208 864358.3657 659861.5626 1305931.751 1140684.665 976658.5423 1141506.424 1644063.752 1393778.789 1394024.527 1364387.601

1121NEG Cer(d18:2/22:0)+HCOO Cer(d40:2)+HCOO Cer (d18:2/22:0) (d18:2) (22:0) C41 H78 O5 N1 664.5885475 12.0208905 0.000877725 1.102041558 0.608080546 0.252578588 70963319.3 64311183.91 67820309.36 58420782.32 97333490.41 75554002.79 28531290.24 112410627.7 75225418.04 64808034.89 87444769.07 110310116.1 102422233.7 102578022.1 102578178.8

1122NEG Cer(d42:1+O)-H Cer(d42:1+O)-H Cer (d42:1+O) (d42:1+O) C42 H82 O4 N1 664.6249325 12.89017999 0.014603111 1.902781024 0.076915043 0.551334552 1843109.613 1711432.372 1566013.17 2118526.251 4283245.625 2128816.656 954117.2941 4116598.013 3202520.756 5731876.201 7896975.235 4073049.668 4585624.742 4472144.961 4588712.998

1123NEG PE(12:0p/20:4)-H PE(32:4p)-H PE (12:0p/20:4) (12:0p) (20:4) C37 H65 O7 N1 P1 666.4504155 8.414316762 0.012795436 1.673669572 0.02968078 0.537729332 4082915.319 2748858.537 3744529.3 2902762.747 1656717.726 3286398.822 2083785.962 6409005.264 5951797.432 4088437.005 5260545.431 7039075.124 6176871.257 6042535.263 6041412.54

1124NEG Cer(d18:0/22:1)+HCOO Cer(d40:1)+HCOO Cer (d18:0/22:1) (d18:0) (22:1) C41 H80 O5 N1 666.6041975 12.24232488 0.110785124 1.061371611 0.780530239 0.21765386 5492474.356 6860757.207 5549197.357 7309423.392 7184588.056 7549188.782 3362943.507 8441242.253 7827703.687 4496405.75 5552912.43 12715949.12 10344924.09 8551446.891 8620383.497

1125NEG Cer(d18:1/22:0)+HCOO Cer(d40:1)+HCOO Cer (d18:1/22:0) (d18:1) (22:0) C41 H80 O5 N1 666.6041975 12.6259715 0.002940509 1.174992231 0.346022138 1.093242748 222514546.2 163077432 205801124.9 177281858.9 224276016.6 212718650.8 121314014.3 297191175.6 285738289.2 161884120 216879977.3 333644871.3 286174655.6 286014014.9 284646351.3

1126NEG Cer(d18:0/22:0)+HCOO Cer(d40:0)+HCOO Cer (d18:0/22:0) (d18:0) (22:0) C41 H82 O5 N1 668.6198475 12.84314919 0.002871804 1.12019454 0.646148067 0.513857012 25456387.53 23177836.25 22226255.37 28569928.31 24006469.3 32032776.47 16589115.85 32928454.74 33289676.06 15930959.28 18747194.02 56670856.7 37587451.43 37590286.01 37402223.15

1127NEG PE(32:1p)-H PE(32:1p)-H PE (32:1p) (32:1p) C37 H71 O7 N1 P1 672.4973655 10.31821201 0.010421278 0.973960278 0.862406009 1.092044749 102356579.7 78765098.38 108374993.2 126060903.1 120890076.3 117890187.5 57023787.67 124500545.5 104178915.9 90615775.2 98867352.85 162112685.8 131185300 131882971.8 129252287

1128NEG PE(32:1p)-H PE(32:1p)-H PE (32:1p) (32:1p) C37 H71 O7 N1 P1 672.4973655 10.60653357 0.015315732 0.943253574 0.745441574 0.326956683 11632748.23 12130305.22 18512132.31 21926577.07 19203728.55 18728907.54 6966225.531 21142583.21 16007627.65 13517722.78 15632835.11 23071642.5 21104975.99 21217386.59 21719039.58

1129NEG PE(17:1/14:0)-H PE(31:1)-H PE (17:1/14:0) (17:1) (14:0) C36 H69 O8 N1 P1 674.4766305 9.423250467 0.013278464 1.050708912 0.83525176 0.286484569 5780500.336 5123551.081 7244234.927 5721019.005 6891360.38 6269449.548 2454693.722 8315476.654 9581262.915 3603588.771 4167373.676 10785476.4 8422475.466 8422535.628 8230270.303

112POS LPC(20:1)+Na LPC(20:1)+Na LPC (20:1) (20:1) C28 H56 O7 N1 P1 Na1 572.3686635 3.68385557 0.032644027 2.224390303 0.1605221 0.187802132 635320.7867 1137196.231 999471.9213 583808.138 2874040.283 910160.1559 671302.2034 6753061.002 3297677.745 1568346.745 2177411.319 1414342.225 2830868.978 2997973.733 2996413.103

1130NEG PE(32:0p)-H PE(32:0p)-H PE (32:0p) (32:0p) C37 H73 O7 N1 P1 674.5130155 11.00455117 0.007156187 1.086155074 0.61686094 0.474067954 22995063.27 22129148.04 25582018.06 24280763.15 38785297.84 30268199 13893044.14 37509848.71 24118279.24 30837710.2 34177831.06 37636696.53 37584621.62 37301836.82 37050908.6

1131NEG Cer(d18:2/23:1)+HCOO Cer(d41:3)+HCOO Cer (d18:2/23:1) (d18:2) (23:1) C42 H78 O5 N1 676.5885475 11.75634791 0.009594335 1.116783167 0.518512545 0.187285159 4670017.026 4959287.884 5051490.233 4781912.215 8146015.399 4068273.834 2747490.668 8026291.672 6347673.591 5801799.152 6476245.299 5976836.196 6115638.394 6112209.894 6216047.108

1132NEG Cer(d43:2+pO)-H Cer(d43:2+pO)-H Cer (d43:2+pO) (d43:2+pO) C43 H82 O4 N1 676.6249325 12.59 0.014935244 1.782990605 0.088714094 0.892388273 5305163.478 6625585.791 4823045.876 5469299.086 15704963.09 7474099.731 2318984.925 16275801.23 11478643.56 18947669.52 20053106.06 11877414.18 13942816.17 14305242.82 13940118.36

1133NEG Cer(d18:1/26:0)-H Cer(d44:1)-H Cer (d18:1/26:0) (d18:1) (26:0) C44 H86 O3 N1 676.6613175 13.74248171 0.010712509 0.937239475 0.78988986 0.247739809 2766196.395 1785843.272 2799163.392 2158145.704 1814288.713 2446665.401 1055952.446 2671231.148 2882084.182 948647.3912 1388850.701 3959305.564 2837020.891 2889442.402 2835964.706

1134NEG LPC(26:1)+HCOO LPC(26:1)+HCOO LPC (26:1) (26:1) C35 H69 O9 N1 P1 678.4715455 8.288074513 0.010434354 0.909347973 0.744575076 0.392620869 5006145.87 3330532.06 4786307.135 4340017.056 3474545.266 5298539.651 2225998.247 5339011.671 5135596.804 1106188.731 1709885.754 8341051.354 5826253.225 5736097.393 5851238.528

1135NEG Cer(d18:2/23:0)+HCOO Cer(d41:2)+HCOO Cer (d18:2/23:0) (d18:2) (23:0) C42 H80 O5 N1 678.6041975 12.28947256 0.003977415 1.178738519 0.355284132 0.632000386 67465461.44 57877688.26 60268373.26 55810770.55 86665486.24 63193581.86 30426139.86 97968761.39 86194412.42 57345225.17 94020559.93 95263313.94 92495474.7 91863757.69 91858630.49

1136NEG PC(10:0e/16:0)+HCOO PC(26:0e)+HCOO PC (10:0e/16:0) (10:0e) (16:0) C35 H71 O9 N1 P1 680.4871955 8.07723989 0.010483301 1.952807755 0.079104224 0.378838798 201681.4266 1372833.16 1531225.837 2145538.067 1473748.429 1907779.19 455579.22 2676093.117 3782471.924 2117526.108 2674635.559 5151904.794 3196394.384 3138160.019 3139982.225

1137NEG LPC(26:0)+HCOO LPC(26:0)+HCOO LPC (26:0) (26:0) C35 H71 O9 N1 P1 680.4871955 9.428526537 0.005812932 0.802168112 0.410927368 0.644432894 9305393.766 6789359.74 8613130.245 9317692.275 6338358.719 10146110.13 4136480.698 8487371.896 8614906.55 2535361.444 2668548.717 14074878.05 9221257.069 9222267.704 9129229.671

1138NEG Cer(d18:1/23:0)+HCOO Cer(d41:1)+HCOO Cer (d18:1/23:0) (d18:1) (23:0) C42 H82 O5 N1 680.6198475 12.672115 0.012069999 1.101949692 0.622590499 0.39836353 21131284.34 18696255.46 21932052.27 17998000.04 20990273.45 21974448.34 9269556.288 30622801.23 29982879.85 16252258.93 15979859.31 33126460.35 28510519.39 27923318.71 27922034.58

1139NEG Cer(d17:1/24:0)+HCOO Cer(d41:1)+HCOO Cer (d17:1/24:0) (d17:1) (24:0) C42 H82 O5 N1 680.6198475 12.93877743 0.007661299 1.22379729 0.256548499 1.149058926 105722613.7 82496613.95 92492621.85 93451379.39 112307319.6 98401953.48 47858825.72 158143087.6 133923357.2 87423138.95 133678042.4 154738931 145844606.6 145846755.8 143918867.8

113POS LPC(22:4)+H LPC(22:4)+H LPC (22:4) (22:4) C30 H55 O7 N1 P1 572.3710685 2.588993633 0.055166095 1.250133089 0.416060291 0.078435088 2789511.237 1974516.77 2993720.42 1013021.179 3383473.611 2629241.014 1317575.02 3147096.614 4412736.876 1953314.735 2250546.414 5400053.144 3765431.999 3619346.888 4033343.267

1140NEG Cer(d16:0+pO/24:0)+HCOO Cer(d40:0+pO)+HCOO Cer (d16:0+pO/24:0) (d16:0+pO) (24:0) C41 H82 O6 N1 684.6147625 12.29476449 0.004314838 1.290593721 0.225300381 0.341535401 6622052.641 5041092.616 5457289.236 5106831.594 7093895.842 5670733.175 2388799.805 10902983.41 9841432.356 5583829.099 7938917.041 8504358.385 8451132.904 8390277.59 8386467.562

1141NEG PE(16:1/16:1)-H PE(32:2)-H PE (16:1/16:1) (16:1) (16:1) C37 H69 O8 N1 P1 686.4766305 9.051734175 0.011147072 0.989983637 0.973738877 0.935077584 67191004.59 33437673.81 73279145.66 62940981.59 63432257.32 93018941.06 24456890.31 75614301.01 67095499.58 38443816.93 39044009.72 144706051 92521984.73 92582115.82 90777306.15

1142NEG PE(33:1p)-H PE(33:1p)-H PE (33:1p) (33:1p) C38 H73 O7 N1 P1 686.5130155 10.71086912 0.008248867 1.028375352 0.821564517 0.510414162 49899065.9 44808580.89 55314878.46 59908061.53 65286242.69 53931662.9 28118040.53 68007267.28 63678602.09 55379167.56 56591758.16 66713361.01 72204761.98 71413861.74 71050317.16

1143NEG PA(35:1)-H PA(35:1)-H PA (35:1) (35:1) C38 H72 O8 N0 P1 687.4970315 10.18709871 0.030522774 0.248487575 0.147754315 0.931215546 45364612.31 5596225.016 15241404.8 12662715.95 5204962.192 914688.7083 108709.1711 9708318.22 5037989.319 5408467.163 389882.868 464252.6763 11215467.86 11821286.18 11219767.87

1144NEG PE(16:0/16:1)-H PE(32:1)-H PE (16:0/16:1) (16:0) (16:1) C37 H71 O8 N1 P1 688.4922805 9.85799649 0.003839744 0.988982016 0.950804706 1.794762806 686170687.6 401502741.4 693751557.1 599776668.2 655712522.8 734253817.6 260443319.8 723310474.7 641775153.3 528187453.2 585001908.5 990899016.1 813437418.7 808074745.1 808028404.4

1145NEG PE(16:0/16:1)-H PE(32:1)-H PE (16:0/16:1) (16:0) (16:1) C37 H71 O8 N1 P1 688.4922805 8.69011491 0.03490472 0.99008279 0.973273165 0.322041135 5392937.535 3843556.868 5396502.668 6006634.276 4093992.496 6362820.727 2076035.894 5751912.826 5961483.597 2628598.68 2888727.658 11481295.93 6787664.305 6392462.668 6393946.9

1146NEG Cer(d18:1/24:3)+HCOO Cer(d42:4)+HCOO Cer (d18:1/24:3) (d18:1) (24:3) C43 H78 O5 N1 688.5885475 11.56830221 0.012595231 1.062996018 0.734527854 0.179515973 8023752.406 8593473.142 7490452.934 6682553.528 13970283.33 8766748.08 3982674.626 11162015.37 11469167.33 8166919.221 11655097.83 10463393.51 11391489.98 11394508.79 11146262.58

1147NEG PE(16:0/16:0)-H PE(32:0)-H PE (16:0/16:0) (16:0) (16:0) C37 H73 O8 N1 P1 690.5079305 9.518556623 0.013269743 0.894770051 0.591019158 0.628822678 13687098.52 12228991.6 13580380.61 13357956.33 10896893.68 12379959.62 4775499.031 13901758.21 15696420.79 6670597.628 7719264.102 19356449.82 14129403.3 14459093.35 14459316.66

1148NEG PE(16:0/16:0)-H PE(32:0)-H PE (16:0/16:0) (16:0) (16:0) C37 H73 O8 N1 P1 690.5079305 10.61123671 0.004405388 0.96873519 0.848058119 0.515743372 21068385.45 25686617.37 23867494.87 25667620.72 37361970.58 25057425.48 9525334.428 33370154.72 22561281.76 28755654.17 29475968.47 30059098.05 32054332.49 31809239.91 31812767.02

1149NEG Cer(d18:1/24:2)+HCOO Cer(d42:3)+HCOO Cer (d18:1/24:2) (d18:1) (24:2) C43 H80 O5 N1 690.6041975 12.05701881 0.003155722 1.131922182 0.522906882 0.470849589 103691488 106785707.2 94195439.99 98847100.99 173944000.9 114326963 46206635.86 177770707.4 152394213.7 103932571.2 145230196.5 157518913.9 160288643.8 161168102.8 161167827.1

114POS LPC(22:1)+H LPC(22:1)+H LPC (22:1) (22:1) C30 H61 O7 N1 P1 578.4180185 5.328997297 0.024747991 1.495936546 0.176106125 0.086934602 664496.8052 720023.0454 1269550.889 541284.1533 986559.2504 753543.2734 539708.3003 1305200.642 1471090.235 1040101.041 715878.8799 2311152.022 1312571.866 1312719.718 1369727.518

1150NEG SM(d30:1)+HCOO SM(d30:1)+HCOO SM (d30:1) (d30:1) C36 H72 O8 N2 P1 691.5031795 7.229161618 0.012094935 1.138839391 0.593650163 0.118423755 2267295.516 1513436.064 2287202.546 2293533.283 2423891.388 2469043.745 719837.4933 2915827.093 3181895.381 1801436.078 1954307.988 4521331.691 3475233.103 3403761.26 3403109.235

1151NEG PE(14:0p/20:5)-H PE(34:5p)-H PE (14:0p/20:5) (14:0p) (20:5) C39 H67 O7 N1 P1 692.4660655 8.758010358 0.015126965 1.269739108 0.163135629 0.297406563 4244763.846 2818304.69 4410816.504 3081459.981 3773124.734 4118948.232 1985334.269 5275493.815 5291226.342 4388241.929 5117953.551 6444114.591 6106267.457 6267847.901 6106623.745

1152NEG Cer(d18:1/24:1)+HCOO Cer(d42:2)+HCOO Cer (d18:1/24:1) (d18:1) (24:1) C43 H82 O5 N1 692.6198475 12.57752666 0.033339262 1.041961606 0.783936982 1.076138832 421621168.9 239479374.8 368136205.6 271561661.4 487988457.3 314911062.5 202110154.3 433339764.4 404464865.2 335930382.1 422833561.7 393293746.8 471732615.9 445953359.6 445072163.7

1153NEG Cer(d18:1/24:1)+HCOO Cer(d42:2)+HCOO Cer (d18:1/24:1) (d18:1) (24:1) C43 H82 O5 N1 692.6198475 12.24998339 0.017544896 1.102625624 0.624889519 0.109065665 9461834.366 12605444.16 10100625.45 11298141.65 13442932.6 12142697.93 4648246.434 17569843.05 14491757.46 8020914.626 12447044.84 18960341.12 14776011.77 14336718.13 14335096

1154NEG PG(16:0/14:0)-H PG(30:0)-H PG (16:0/14:0) (16:0) (14:0) C36 H70 O10 N0 P1 693.4712115 8.706242108 0.008028819 0.964894436 0.909273452 0.320663033 6748109.991 3163155.181 7017487.162 3768820.444 2264645.772 4942921.938 1404534.81 5041282.951 7183367.694 2427413.181 2548296.36 8320619.803 7035648.91 6939527.576 6937907.825

1155NEG PC(12:0/14:0)+HCOO PC(26:0)+HCOO PC (12:0/14:0) (12:0) (14:0) C35 H69 O10 N1 P1 694.4664605 7.368506618 0.005020623 0.938560826 0.895255033 0.339442886 4164927.284 1468423.614 4530672.067 3021204.573 657583.2323 3588441.691 1327926.244 2231440.971 4326303.826 399214.0519 365649.7587 7709755.856 3991338.713 3956886.387 3956774.266

1156NEG PE(34:4p)-H PE(34:4p)-H PE (34:4p) (34:4p) C39 H69 O7 N1 P1 694.4817155 9.425564368 0.014469452 1.208985842 0.285959208 0.405228765 14732255.77 13163389.83 15893691.02 12289787.63 16622721.05 13517970.66 6652057.748 24465193.03 20764471.1 14935767.47 16197448.57 21223598.86 21187562.54 21722352.81 21186234.49

1157NEG Cer(d18:0/24:1)+HCOO Cer(d42:1)+HCOO Cer (d18:0/24:1) (d18:0) (24:1) C43 H84 O5 N1 694.6354975 12.77428856 0.010769153 1.522924536 0.437609396 0.992832536 44710416.51 49947800.45 30771585.65 47672723.31 40340702.7 46010939.01 19172012.15 66525949.74 54576625.94 24148225.25 30849867.9 199856437 55658811.06 55660938.46 54628083.6

1158NEG Cer(d18:1/24:0)+HCOO Cer(d42:1)+HCOO Cer (d18:1/24:0) (d18:1) (24:0) C43 H84 O5 N1 694.6354975 13.19030872 0.002130667 1.149823843 0.425842391 1.945307715 772995828 657691330.6 692309727.7 739164588 745823579.6 738097895.5 358212350.7 1097528902 1068101923 577628885.7 772171950 1123585786 1013201197 1009468378 1009474092

1159NEG PE(34:3p)-H PE(34:3p)-H PE (34:3p) (34:3p) C39 H71 O7 N1 P1 696.4973655 10.1307977 0.044979649 0.899319863 0.42511763 0.605279222 17451804.06 13092057.04 18364325.1 16794664.71 17726736.86 21887245.39 8094864.673 16474379.19 18440461.15 13941965.36 17101661.93 20660187.69 17767134.71 19183852.81 17759072.75

115POS LPG(22:6)+Na LPG(22:6)+Na LPG (22:6) (22:6) C28 H45 O9 N0 P1 Na1 579.2693445 1.788151605 0.026340839 1.31850813 0.288064642 0.062696896 447010.8431 961953.7973 521222.2289 1383105.723 1508230.221 831406.5827 452758.6026 1500038.492 1909447.363 1197373.003 1031239.124 1362576.785 1525783.272 1596830.682 1598303.593

1160NEG PE(34:3p)-H PE(34:3p)-H PE (34:3p) (34:3p) C39 H71 O7 N1 P1 696.4973655 9.846405698 0.008003681 0.976440603 0.901120872 0.464531379 25594970.74 14325509.71 28188318.75 20885754.61 24215280.37 27158540.45 10784229.98 23435951.54 26657590.84 17567025.44 20414068.9 38202513.63 31500013.65 31499714.96 31938567.2

1161NEG Cer(d17:1+hO/24:0)+HCOO Cer(d41:1+hO)+HCOO Cer (d17:1+hO/24:0) (d17:1+hO) (24:0) C42 H82 O6 N1 696.6147625 12.20883428 0.014543518 1.167149113 0.478951515 0.074394233 8452836.851 7761746.243 8021835.979 6978570.276 17264147.77 9016638.706 5750727.474 13000618.83 13294229.97 8760216.449 10127327.38 16173023.63 11417278.47 11136514.89 11426182.46

1162NEG Cer(d18:0/24:0)+HCOO Cer(d42:0)+HCOO Cer (d18:0/24:0) (d18:0) (24:0) C43 H86 O5 N1 696.6511475 13.38769348 0.007969328 1.235082361 0.447404536 0.776264632 46710352.04 53670990.75 39615602.19 73560279.85 54399782.52 66517413.53 29019656.95 92465796.69 86939357.94 37960828.56 40870666.46 125847150.8 80895269.37 80908888.77 79790541.79

1163NEG PE(34:2p)-H PE(34:2p)-H PE (34:2p) (34:2p) C39 H73 O7 N1 P1 698.5130155 10.87486877 0.015793529 0.880523677 0.41262748 0.641627627 12061923 11363957.59 14519181.17 13844305.44 15548661.76 16155477.2 8350734.508 8114133.674 15343880.74 11457808.64 10858492.55 19392958.94 17213423.08 17694556.61 17691521.56

1164NEG PE(34:2p)-H PE(34:2p)-H PE (34:2p) (34:2p) C39 H73 O7 N1 P1 698.5130155 10.47323345 0.008748283 1.008705762 0.928477385 1.119495836 118393661.3 121603197.9 120838179 128757877.9 149785573.8 117595146 85632980.87 151488278.2 123207331.3 120944473.4 121627439.4 160663165 153080844.5 153082819.4 150773921.6

1165NEG Cer(d17:0+pO/24:0)+HCOO Cer(d41:0+pO)+HCOO Cer (d17:0+pO/24:0) (d17:0+pO) (24:0) C42 H84 O6 N1 698.6304125 12.63828003 0.013311552 1.259573724 0.223091975 0.401430922 7729760.227 6918124.109 7636369.914 8097634.075 8535269.267 8107781.713 3140795.46 13325057.28 11660102.27 8354157.483 9826237.149 12925028.27 11792060.18 11523499.27 11525097.51

1166NEG PE(17:1/16:1)-H PE(33:2)-H PE (17:1/16:1) (17:1) (16:1) C38 H71 O8 N1 P1 700.4922805 9.539300073 0.009285391 0.798743709 0.202933174 1.003312954 29275774.04 21283519.9 39932964.47 27034901.79 29290327.47 29648686.08 11777717.78 28533462.66 24750459.79 19286770.05 19483328.58 37119507.21 31936455.3 31937289.45 31425978.42

1167NEG PE(34:1p)-H PE(34:1p)-H PE (34:1p) (34:1p) C39 H75 O7 N1 P1 700.5286655 11.06117928 0.003852043 1.082929613 0.614018031 3.157636139 1347095727 1287729448 1440075663 1865040577 2064970046 1584223988 702897152.8 2221254296 1679039601 1751513282 1945633948 2084020459 2045951106 2032355631 2046000668

1169NEG PE(34:0p)-H PE(34:0p)-H PE (34:0p) (34:0p) C39 H77 O7 N1 P1 702.5443155 11.67571747 0.007046948 1.062760209 0.752550172 0.03392301 28488653 19830563.81 33403973.3 30411285.53 37878605.25 37885525.36 13763805.79 42748478.59 31151187.69 27703264.6 31961979.28 52362446.11 43785593.18 43255937.83 43255011.81

116POS LPC(22:0)+H LPC(22:0)+H LPC (22:0) (22:0) C30 H63 O7 N1 P1 580.4336685 6.952457065 0.000909741 1.065466963 0.682002199 0.028386403 1667745.595 1116726.421 1652318.57 1344035.054 1487231.004 1594587.219 1243576.403 2049460.312 1874560.965 1065371.666 1030010.901 2179873.994 2175795.335 2175803.966 2179229.891

1170NEG Cer(d18:2/25:1)+HCOO Cer(d43:3)+HCOO Cer (d18:2/25:1) (d18:2) (25:1) C44 H82 O5 N1 704.6198475 12.34170293 0.003316085 1.047206896 0.814081336 0.152441436 11705016.45 12026430.33 9612965.946 8879251.743 15552766.06 11279555.53 4295422.552 16296955.71 15481222.8 7520094.314 13031944.02 15690265.39 15850981.99 15851199.67 15760222.23

1171NEG SM(d31:1)+HCOO SM(d31:1)+HCOO SM (d31:1) (d31:1) C37 H74 O8 N2 P1 705.5188295 7.871571987 0.011506406 1.124462018 0.644087369 0.186566491 4318448.446 3033006.824 4442480.96 4250585.709 4150187.815 4578555.732 1303568.991 5502629.339 5772438.161 3068687.56 3511408.429 8697863.627 5939311.837 5939195.344 6058412.57

1172NEG Cer(d18:1/25:1)+HCOO Cer(d43:2)+HCOO Cer (d18:1/25:1) (d18:1) (25:1) C44 H84 O5 N1 706.6354975 12.83424398 0.022325209 1.114814304 0.59204309 0.166183333 32335095.34 28399037.8 30485677.69 38992387.97 44681966.79 27596066.26 13391853.37 46667795.43 54309678.47 24973309.89 37107569.9 49288799.88 50777925.55 48864991.73 48862549.59

1173NEG PE(14:0/20:5)-H PE(34:5)-H PE (14:0/20:5) (14:0) (20:5) C39 H67 O8 N1 P1 708.4609805 8.250237088 0.01289436 1.110621521 0.740357731 0.159428662 2036396.633 1257212.951 2952559.131 1416045.666 965868.1867 1703727.846 949452.8878 2055346.438 2265235.319 1064063.347 1117055.596 4023577.411 2628416.519 2570529.543 2629188.962

1175NEG Cer(d17:0+pO/25:2)+HCOO Cer(d42:2+pO)+HCOO Cer (d17:0+pO/25:2) (d17:0+pO) (25:2) C43 H82 O6 N1 708.6147625 12.324 0.008354945 1.534505027 0.093332909 0.610402356 5551236.465 3178623.434 4935471.809 3954999.997 8671436.063 5968313.466 3142233.178 7669438.915 6451757.601 10329151.73 12556892.8 9353782.594 8206133.711 8078024.086 8102971.491

1176NEG Cer(d18:1/25:0)+HCOO Cer(d43:1)+HCOO Cer (d18:1/25:0) (d18:1) (25:0) C44 H86 O5 N1 708.6511475 13.34468541 0.010024515 1.069725544 0.709445732 0.425181465 58727521.58 47627213.47 55651545.26 54047814.38 52276430.76 51248322.91 22432280.69 81177255.59 68841451.02 37266708.62 54493214.34 77650747.01 74428070.76 74468327.78 73163465.97

1177NEG PE(14:0/20:4)-H PE(34:4)-H PE (14:0/20:4) (14:0) (20:4) C39 H69 O8 N1 P1 710.4766305 8.971124615 0.007234439 1.047284519 0.846930559 0.332304037 11377120.49 6736109.079 13076381.73 7531735.505 6743395.951 10998924.17 4729752.503 11578089.57 11870649.96 6293832.702 7063239.535 17597959.99 12853501.13 13017673.28 12858425.9

1178NEG Cer(d18:1+hO/24:0)+HCOO Cer(d42:1+hO)+HCOO Cer (d18:1+hO/24:0) (d18:1+hO) (24:0) C43 H84 O6 N1 710.6304125 12.25744428 0.020131264 1.238570605 0.306713527 0.423919292 14405866.91 12213449.63 13272472.78 12326276.82 15293166.95 13388757.48 5476409.529 23964785.08 21671024.49 11550848.47 15460585.4 22076697.29 18602864.87 17887922.04 18399193.2

1179NEG PE(16:1/18:2)-H PE(34:3)-H PE (16:1/18:2) (16:1) (18:2) C39 H71 O8 N1 P1 712.4922805 9.252547683 0.007508008 1.004799688 0.984829659 0.581409317 41281132.17 26118616.73 45596881.25 38710185.06 38994022.25 51439841.65 17192822.36 45583889.87 37533053.3 27829002.31 32272171.16 82891939.92 56092255.12 55365669.79 55372552.66

117POS DG(16:0/16:1)+NH4 DG(32:1)+NH4 DG (16:0/16:1) (16:0) (16:1) C35 H70 O5 N1 584.5248505 11.21905427 0.001953518 0.796980052 0.259199083 0.687035518 81450861.16 82499444.97 78970819.8 95156741.66 74730109.53 78155451.72 23831386.04 104558822.8 74768985.54 46525187.71 41036211.86 100567465.3 97974273.82 98305333.36 98307710.46

1180NEG PE(16:0/18:3)-H PE(34:3)-H PE (16:0/18:3) (16:0) (18:3) C39 H71 O8 N1 P1 712.4922805 9.722983138 0.080958586 0.787340725 0.579716119 0.680468444 22522000.4 5338830.08 13400631.98 6754125.287 5675469.42 22979456.27 3468914.186 6248259.258 20689869.02 4315056.295 5221209.971 20422508.9 14743709.25 14614656.38 16835455.02

1181NEG PE(35:2p)-H PE(35:2p)-H PE (35:2p) (35:2p) C40 H75 O7 N1 P1 712.5286655 10.75423805 0.04707489 0.964217331 0.830651576 0.659037044 29558390.62 30552597.43 33438654.11 41880368.63 46340210.03 33113268.44 19648175.5 45903277.93 40021607.5 34186355.06 19355780.55 48079187.93 35630538.51 38709101.55 38696273.76

1182NEG Cer(d18:0+pO/24:0)+HCOO Cer(d42:0+pO)+HCOO Cer (d18:0+pO/24:0) (d18:0+pO) (24:0) C43 H86 O6 N1 712.6460625 12.85668899 0.012424337 1.460968439 0.122795743 0.653053845 9692443.074 9394708.032 8351556.555 9659379.599 11807013.7 8654801.682 4091402.931 21931344.9 19020253.63 10615661.15 13410128.02 15024410.49 15561823.86 15899049.12 15561670.74

1183NEG PA(37:2)-H PA(37:2)-H PA (37:2) (37:2) C40 H74 O8 N0 P1 713.5126815 10.24313021 0.031907635 0.406612349 0.152704365 0.691631496 19941353.76 5920715.712 7531947.165 22990867.06 9076528.998 233034.9456 48035.45726 13424802.47 5315981.987 7602405.944 173101.6859 147846.1429 9885033.557 10492691.41 9998589.873

1184NEG PE(16:1/18:1)-H PE(34:2)-H PE (16:1/18:1) (16:1) (18:1) C39 H73 O8 N1 P1 714.5079305 9.944415735 0.024974343 0.946140585 0.73602082 3.750537499 882567349.8 707920822.3 904912952.6 970462699.9 1210643417 1051331570 418467427.1 1077508543 891166309.1 791522027 852040494.4 1388635966 1222624891 1171659277 1170792205

1185NEG PE(16:1/18:1)-H PE(34:2)-H PE (16:1/18:1) (16:1) (18:1) C39 H73 O8 N1 P1 714.5079305 8.797912313 0.064350006 0.840766577 0.493126497 0.323516983 5966462.431 2287160.723 7384954.33 5951070.874 4255376.763 7067950.821 2026247.559 3713129.045 6045907.672 3465576.377 3870284.841 8550984.622 7727502.766 8618596.729 7721387.752

1186NEG PE(16:0/18:1)-H PE(34:1)-H PE (16:0/18:1) (16:0) (18:1) C39 H75 O8 N1 P1 716.5235805 10.67090069 0.003422277 0.991196775 0.934819409 4.18432766 1780311951 1472116492 1905458007 1750407749 1627660329 1773691644 1073219505 2051630387 1813516021 1462803131 1599552479 2218166515 2098222834 2085818210 2085850758

1187NEG PE(16:0/18:1)-H PE(34:1)-H PE (16:0/18:1) (16:0) (18:1) C39 H75 O8 N1 P1 716.5235805 9.620312008 0.02249458 1.017337673 0.911276078 0.522541155 35004298.99 28001068.36 34534880.13 30068785.82 28211400.05 37616247.32 18921550.84 33411563.17 36008624.16 25642130.5 30577595.68 52228958.31 37572484.33 37570159.24 39054426.55

1188NEG PG(16:1/16:1)-H PG(32:2)-H PG (16:1/16:1) (16:1) (16:1) C38 H70 O10 N0 P1 717.4712115 7.91171378 0.002430088 1.22659391 0.545918736 0.214446005 5831172.184 3401385.264 5829233.172 6314703.859 6274585.762 6969242.282 1860761.281 7811697.473 6547176.651 4769411.294 5103943.136 16372086.94 9214003.981 9173539.859 9210241.034

1189NEG SM(d14:1/18:1)+HCOO SM(d32:2)+HCOO SM (d14:1/18:1) (d14:1) (18:1) C38 H74 O8 N2 P1 717.5188295 7.420288975 0.019412783 1.181789691 0.507245813 0.202677854 4802298.875 3888148.859 4869514.206 4457003.125 5812148.018 5770331.49 1779528.86 7073762.492 7589826.598 3904826.474 4232126.172 10400247.87 7790912.505 7791555.847 7532166.509

118POS DG(16:0/16:0)+NH4 DG(32:0)+NH4 DG (16:0/16:0) (16:0) (16:0) C35 H72 O5 N1 586.5405005 11.83483391 0.006687431 0.857826874 0.541315028 0.471125689 54217480.85 31640942.62 51102598.09 35558814.74 29527589.78 45526210.93 14400990.4 49017572.09 47355670.31 19873689.1 18888814.51 62838582.6 51784726.73 51189789.92 51189214.7

1190NEG PE(14:0p/22:6)-H PE(36:6p)-H PE (14:0p/22:6) (14:0p) (22:6) C41 H69 O7 N1 P1 718.4817155 9.142722445 0.002476844 1.588289193 0.118058907 0.902628195 12220107.65 10088793.46 13996401.75 12411439.12 11863356.77 14329856.64 4768606.865 29712727.68 26035831.22 15932873.17 12341093.6 30187540.07 19092707.47 19011043.52 19011022.41

1191NEG PE(34:0)-H PE(34:0)-H PE (34:0) (34:0) C39 H77 O8 N1 P1 718.5392305 10.36278407 0.012625578 1.011662031 0.934654367 0.335284514 16620183.6 15536829.05 15286101.23 15842720.01 21767999.34 18357190.67 7592569.2 20698574.65 16761212.31 16807533.98 20613252.9 22143863.45 18774688.76 18772257.71 18365908.57

1192NEG PE(18:0/16:0)-H PE(34:0)-H PE (18:0/16:0) (18:0) (16:0) C39 H77 O8 N1 P1 718.5392305 11.30777625 0.006162215 1.083785028 0.640840914 0.392706675 22930661.23 28870164.3 25865840.76 33648891.01 34314902.71 25094909.56 10781590.98 44873301.26 30227496.19 31028719.71 35855746.36 32262744.96 38911558.67 38435024.23 38682995.66

1193NEG Cer(d18:2/26:1)+HCOO Cer(d44:3)+HCOO Cer (d18:2/26:1) (d18:2) (26:1) C45 H84 O5 N1 718.6354975 12.61822733 0.004545507 0.931372222 0.750488172 0.875550465 25540468.66 25619640.56 23892166.82 23153255.91 25113871.48 26398961.92 11110007.44 30396351.28 35973080.76 10742331.69 13608537.45 37613218.06 30160078.77 30162207.59 29924311.72

1194NEG PG(18:1/14:0)-H PG(32:1)-H PG (18:1/14:0) (18:1) (14:0) C38 H72 O10 N0 P1 719.4868615 8.8106959 0.006199792 0.960815785 0.902867431 0.660624622 23551391.18 12245312.09 26257945.75 23366072.84 12113976.57 24095231.72 5730348.565 26067737.93 23490000.29 7801685.658 10891828.95 42882355.44 27527583.32 27236096.11 27232111.16

1195NEG SM(d18:1/14:0)+HCOO SM(d32:1)+HCOO SM (d18:1/14:0) (d18:1) (14:0) C38 H76 O8 N2 P1 719.5344795 8.467825137 0.004557894 1.044109974 0.84035275 1.675609702 279994756.6 192707240 277991097.3 230074903 239967819.3 258756099.6 97229357.38 334937345.3 335523540.8 164130740.7 191850961.7 421080319.7 333458616.4 330694858.9 331014068.4

1196NEG PC(16:1/12:0)+HCOO PC(28:1)+HCOO PC (16:1/12:0) (16:1) (12:0) C37 H71 O10 N1 P1 720.4821105 7.5428087 0.017335155 0.94290727 0.901731461 0.55209457 10437693.15 4705066.855 10884883.07 8198733.196 2739671.839 12579548.59 3007519.096 6465856.179 10925983.64 1560893.857 1725667.274 23030983.27 11126930 11339600.59 10954050.47

1197NEG PE(16:0p/20:5)-H PE(36:5p)-H PE (16:0p/20:5) (16:0p) (20:5) C41 H71 O7 N1 P1 720.4973655 9.778573002 0.003590608 1.112455773 0.62858584 0.946797015 711453975.4 552571622.2 773632537.2 669650011.3 849017626.1 850773700.7 217659229.2 906353363.6 916182484.5 512366315.1 1052947292 1297194567 1093486770 1086679564 1093444808

1198NEG Cer(d18:1/26:1)+HCOO Cer(d44:2)+HCOO Cer (d18:1/26:1) (d18:1) (26:1) C45 H86 O5 N1 720.6511475 13.12980595 0.022624681 0.893998636 0.605178984 1.667694159 88320038.83 75606293.6 85285626.11 81041601.51 73186771.8 90249787.29 31885069.16 78498441.61 106785808.1 41281990.37 51292546.54 131614437.2 119858145.3 124682392.9 124679675.4

1199NEG SM(d16:0/16:0)+HCOO SM(d32:0)+HCOO SM (d16:0/16:0) (d16:0) (16:0) C38 H78 O8 N2 P1 721.5501295 8.876863332 0.007103909 0.989255658 0.959104194 0.546856863 17592915.37 14431424.95 17492747.72 16187153.71 16339446.73 15121838.01 6777128.139 21263800.15 21508890.41 9492718.969 10720445.08 26358564.05 21248142.55 20990661.37 20987027.53

119POS LPC(22:6)+Na LPC(22:6)+Na LPC (22:6) (22:6) C30 H50 O7 N1 P1 Na1 590.3217135 2.047354017 0.041275545 1.387396973 0.330993383 0.096734063 1654160.88 1124488.528 1692446.588 530432.2579 1238602.254 1174876.322 789933.9041 2293952.272 3064577.509 666962.3059 847742.4979 2624389.544 1851590.816 1989201.407 1991932.854

11POS So(d18:0+pO)+H So(d18:0+pO)+H So (d18:0+pO) (d18:0+pO) C18 H40 O3 N1 318.3002705 2.417540088 0.008162267 0.990925363 0.958186834 0.030132225 2829917.631 1119367.092 3134834.755 1543619.952 2705182.327 2729407.669 2279215.681 2454938.028 1709428.736 2175860.621 2035745.236 3279530.595 3291897.778 3246040.701 3245549.658

1200NEG PC(14:0/14:0)+HCOO PC(28:0)+HCOO PC (14:0/14:0) (14:0) (14:0) C37 H73 O10 N1 P1 722.4977605 8.521710202 0.004510108 0.971849797 0.927310827 1.937473958 151432743.4 91438695.56 150079365.2 128333849.2 74548024.92 153737687.3 60098654.77 135018319.1 178843285.7 43150169.84 53604783.08 257754595.3 163019782.8 161753275.5 161752550.5

1201NEG PE(36:4p)-H PE(36:4p)-H PE (36:4p) (36:4p) C41 H73 O7 N1 P1 722.5130155 10.30025137 0.009442148 1.218974088 0.164600524 5.601548834 1252088138 1033972099 1346689088 1382236276 1720954515 1354508648 788836128 1857508330 1739550850 1709732136 1954238981 1812180977 1751971355 1780889593 1780988986

1202NEG Cer(d18:1/26:0)+HCOO Cer(d44:1)+HCOO Cer (d18:1/26:0) (d18:1) (26:0) C45 H88 O5 N1 722.6667975 13.74248171 0.004075078 0.95784966 0.85582933 1.637459631 134417356 90242479.44 125506788.6 99963929.8 82616928.06 115624110.4 46832077.78 133196395.1 141341140.3 53553801.45 63752862.26 182366232.1 142179024.9 141654279.6 141026339.5

1203NEG Cer(d18:0/26:1)+HCOO Cer(d44:1)+HCOO Cer (d18:0/26:1) (d18:0) (26:1) C45 H88 O5 N1 722.6667975 13.33458394 0.007096793 0.879665852 0.751441345 0.954058873 16495811.42 29712036.31 15070089.06 28982998.14 13831195.06 34487337.24 8264186.966 23254148.86 29455194.47 4154831.167 4355886.416 52419377.3 28673264.51 29024001.93 28667092.64

1204NEG PE(36:3p)-H PE(36:3p)-H PE (36:3p) (36:3p) C41 H75 O7 N1 P1 724.5286655 10.90477919 0.012638609 1.10502314 0.520463401 0.291870276 75365932 68026399.28 81688625.81 91964821.28 108786704.9 94188652.62 48782238.08 127431354.5 99326080.16 80940334.1 89940593.57 128214788.2 121606742 118986389.2 118979573

1205NEG PE(36:3p)-H PE(36:3p)-H PE (36:3p) (36:3p) C41 H75 O7 N1 P1 724.5286655 10.63523548 0.004607998 1.015541391 0.912430142 0.992403282 110276592.8 90366204.4 115881114.4 125516792.7 131521742.7 119597676.9 78664421.68 152222554 130460711.3 85341073.64 93938335.11 163305700.4 144454885 143307829.5 143308273.3

1206NEG Cer(d18:0/26:0)+HCOO Cer(d44:0)+HCOO Cer (d18:0/26:0) (d18:0) (26:0) C45 H90 O5 N1 724.6824475 13.96640055 0.003724131 0.906580936 0.828453991 1.114076477 25174972.85 39813894.14 23735858.93 36704003.61 14691679.25 48135888.4 12477006.43 29132536.11 35732644.49 4770684.462 5014269.582 83542429.04 39391991.06 39392119.22 39646696.49

1207NEG PE(17:1/18:2)-H PE(35:3)-H PE (17:1/18:2) (17:1) (18:2) C40 H73 O8 N1 P1 726.5079305 9.800230037 0.004365852 1.10808128 0.634148842 0.386726701 30970562.97 22867841.76 33348357.17 40133483.11 31002143.92 30260485.73 10569938.72 47110968.38 45863350.12 22804476.04 31486181.42 51130238.42 41271042.14 40958253.57 41268024.11

1208NEG PE(36:2p)-H PE(36:2p)-H PE (36:2p) (36:2p) C41 H77 O7 N1 P1 726.5443155 11.0884704 0.002422926 1.110534483 0.465031828 1.61062315 288754788.3 298920899.3 310615065.1 389903529.2 431618158.9 355337323.5 170702292.7 474848773.4 392297501.9 396591887.2 416167524.9 453917391.4 447721941.9 449592532.3 447700288.5

1209NEG Cer(d42:1+hO+O)+HCOO Cer(d42:1+hO+O)+HCOO Cer (d42:1+hO+O) (d42:1+hO+O) C43 H84 O7 N1 726.6253275 11.84188084 0.006396359 1.309280695 0.299875658 0.303449976 5449321.786 5687621.152 4641881.805 5963859.105 7589730.331 4399264.82 2145408.232 12604509.8 10495995.52 5327903.061 7011936.003 6578483.506 7936052.901 7848362.868 7935867.702

120POS PC(23:0e)+H PC(23:0e)+H PC (23:0e) (23:0e) C31 H65 O7 N1 P1 594.4493185 5.995460417 0.009071508 1.509127548 0.200054297 0.114348242 601007.4129 1272127.631 706541.8587 2208699.831 1145126.572 1299134.32 235394.3691 2564735.709 2293158.605 1425065.206 1635507.397 2761111.403 2612928.65 2582005.16 2566882.746

1210NEG PE(17:1/18:1)-H PE(35:2)-H PE (17:1/18:1) (17:1) (18:1) C40 H75 O8 N1 P1 728.5235805 10.34851522 0.003525082 1.062889181 0.600008322 1.020562285 209760204.8 209513353 235395739.4 233477142.5 268108358.9 221539217.3 133708898.3 318725507.1 249825672.3 232729861.4 255384403.3 274068010.9 276051624.9 276047937.3 274367751.8

1211NEG PE(36:1p)-H PE(36:1p)-H PE (36:1p) (36:1p) C41 H79 O7 N1 P1 728.5599655 11.70950984 0.002033303 1.133364797 0.495504398 1.561939845 473877766.3 464911543.1 566069616.8 673888310.6 747883005.9 589860638.6 239627968.3 947768963.6 694405201.5 590685135 654719721 858259984 833149209.8 830219053.8 833150363.8

1212NEG Cer(d18:0+pO/24:0+O)+HCOO Cer(d42:0+pO+O)+HCOO Cer (d18:0+pO/24:0+O) (d18:0+pO) (24:0+O) C43 H86 O7 N1 728.6409775 12.46069332 0.003852769 1.340177402 0.282556764 0.510989253 12926718.23 14804600 11698607.67 14592582.69 14902873.52 9754242.548 4560482.17 30547931.95 25683044.63 11994074.89 14992024.05 17667097.25 20380386.73 20516044.99 20379109.91

1213NEG PE(17:0/18:1)-H PE(35:1)-H PE (17:0/18:1) (17:0) (18:1) C40 H77 O8 N1 P1 730.5392305 11.0079934 0.019517825 1.111826766 0.484696682 0.585104364 236820445.7 246615982.8 251833486.7 212128856.4 197546435.4 200868341.3 134029453.1 386908346.2 240655120.6 254206479.3 223908021.2 256604104.5 322056319.9 311353039.6 311465616.3

1214NEG PE(35:1)-H PE(35:1)-H PE (35:1) (35:1) C40 H77 O8 N1 P1 730.5392305 10.04255781 0.021030469 1.011784712 0.942300893 0.329933936 10206041.26 8110553.594 9731368.54 8287605.781 9903188.309 8787959.074 5705282.386 9769651.637 9900517.218 7453032.47 7317426.159 15529280.67 10705931.12 10310800.95 10341411.42

1215NEG PE(36:0p)-H PE(36:0p)-H PE (36:0p) (36:0p) C41 H81 O7 N1 P1 730.5756155 12.26966511 0.02140165 1.171978397 0.331704515 0.291088014 5595099.473 5027690.018 6571543.608 6935596.356 7496474.71 7184387.542 3209243.784 10005216.28 7294070.128 6807290.358 8435200.66 9734388.246 9038411.71 8706688.435 9036807.131

1216NEG SM(d16:1/17:1)+HCOO SM(d33:2)+HCOO SM (d16:1/17:1) (d16:1) (17:1) C39 H76 O8 N2 P1 731.5344795 8.070026828 0.030466385 1.480856006 0.20903341 0.238858198 1469078.39 911065.043 1670779.55 1115261.444 1415561.243 1298998.888 585770.0864 2656499.394 2490818.382 1119601.473 1288424.697 3529133.878 2250772.159 2250030.334 2371277.893

1217NEG PS(16:0/16:1)-H PS(32:1)-H PS (16:0/16:1) (16:0) (16:1) C38 H71 O10 N1 P1 732.4821105 8.733664198 0.019293711 0.768707666 0.545196016 0.68710964 14357068.99 6794202.448 17843032.44 22037659.25 9395066.39 43647719.16 13482545.42 10777472.61 11419590.15 6710152.037 8855049.681 36445323.9 17013934.01 16451087.66 17012807.86

1218NEG SM(d17:1/16:0)+HCOO SM(d33:1)+HCOO SM (d17:1/16:0) (d17:1) (16:0) C39 H78 O8 N2 P1 733.5501295 9.014801993 0.008108319 1.113863455 0.556825649 0.831029314 177804555.1 135295142.2 177202371 152793733.9 180923434.3 167569327.6 71824383.56 241372085.9 238922289 140107607.9 154424628.4 257843269.1 223492132.3 220264905.1 223271104.3

1219NEG PE(14:0/22:6)-H PE(36:6)-H PE (14:0/22:6) (14:0) (22:6) C41 H69 O8 N1 P1 734.4766305 8.684378997 0.00547559 1.06949526 0.730410786 0.25199515 7652422.474 7514227.07 10334993.07 7704378.363 6224671.179 6746507.318 4032296.497 10665932.5 11103902.83 5108776.313 6528281.869 11947105.93 11132726.75 11027641.03 11133057.76

121POS Cer(d18:1/20:0)+H Cer(d38:1)+H Cer (d18:1/20:0) (d18:1) (20:0) C38 H76 O3 N1 594.5819705 12.00654286 0.010953168 1.096875668 0.532128445 0.060334359 14384659.2 9925584.606 13009037.27 11699636.78 16283427.41 15190686.58 9543928.571 18239676.84 15510241.87 10572524.76 13750239.46 20674236.54 20321892.58 20565216.99 20120388.2

1220NEG PE(16:1/20:5)-H PE(36:6)-H PE (16:1/20:5) (16:1) (20:5) C41 H69 O8 N1 P1 734.4766305 8.393198242 0.00626986 1.068438937 0.771368556 0.231261388 20716964.79 11491515.67 22125388.42 15125226.65 14265925.75 22039397.05 7863971.557 19410158.78 18637675.9 14771515.39 17771085.32 34548415.73 25335203.33 25335105.93 25061014.4

1221NEG PC(17:1/12:0)+HCOO PC(29:1)+HCOO PC (17:1/12:0) (17:1) (12:0) C38 H73 O10 N1 P1 734.4977605 8.147644967 0.034267475 1.110489216 0.796936451 0.320806255 5147571.553 2814315.282 5510005.903 3596683.65 1274414.624 5251326.54 1556500.893 3120496.577 6923834.642 1666501.899 2292313.631 10641587.56 5274551 5599571.745 5601438.3

1222NEG CerG1(d36:5+pO)-H CerG1(d36:5+pO)-H CerG1 (d36:5+pO) (d36:5+pO) C42 H72 O9 N1 734.5212575 10.008 0.008930356 1.044522659 0.761611304 0.386243461 33007633.13 48101372.5 37153047.79 47433764.82 39792595.22 33457133.25 17458703.31 48525387.54 41930555.67 43493741.66 50332295.5 47843354.11 47621967.19 48366654.01 48365846.73

1223NEG PE(16:0/20:5)-H PE(36:5)-H PE (16:0/20:5) (16:0) (20:5) C41 H71 O8 N1 P1 736.4922805 9.075918467 0.001851745 1.13554327 0.495913291 0.634327007 59537951.87 42619585.4 71952669.42 64011036.48 74196151.79 79862846.77 26056143.46 90632099.46 67618936.83 70125320.32 83627714.68 107277419.5 100323847.2 100552781.1 100692557.6

1224NEG PC(16:0e/14:0)+HCOO PC(30:0e)+HCOO PC (16:0e/14:0) (16:0e) (14:0) C39 H79 O9 N1 P1 736.5497955 10.08184335 0.012466086 0.925690408 0.777554032 1.068620734 35751618.66 31822967.17 34361148.91 36372462.33 40860892.91 46834432.06 14316949.73 41123484.91 47292539.69 16372259.86 17850171.8 72253886.61 44578288.19 44585136.96 43626005.86

1225NEG PE(18:1/18:3)-H PE(36:4)-H PE (18:1/18:3) (18:1) (18:3) C41 H73 O8 N1 P1 738.5079305 9.476304857 0.044182249 0.867002865 0.596814334 0.319468002 19175693.77 14640631 41582899.72 28783398.5 14206485.44 17930458.85 12302132.26 20255357.96 23809380.01 7125570.698 28903418.54 25793595.89 29231387.65 31327198.61 28896316.78

1226NEG PE(16:0/20:4)-H PE(36:4)-H PE (16:0/20:4) (16:0) (20:4) C41 H73 O8 N1 P1 738.5079305 9.877971202 0.007902843 1.09346119 0.541964206 0.555091191 637810816.8 352686584.8 676298640 398095225.6 473751542.6 546199995.4 327976568.8 630237099.3 581022252.2 508705591.2 602926023.4 722288348.5 714560786.2 704930404.1 704807233.6

1227NEG PE(18:1/18:2)-H PE(36:3)-H PE (18:1/18:2) (18:1) (18:2) C41 H75 O8 N1 P1 740.5235805 10.11334296 0.0129451 1.136957968 0.36531071 1.64910275 442511166.9 427814156.2 453186893.2 445178277.6 586529683.2 471854526 265035893.7 716109963 538791282.5 487989148.1 554657490.5 651681330.8 667031949.2 666937045 652140824.1

1228NEG PE(16:0/20:3)-H PE(36:3)-H PE (16:0/20:3) (16:0) (20:3) C41 H75 O8 N1 P1 740.5235805 10.51169333 0.009169357 1.151670159 0.442471025 0.432751599 47077508.63 22468533.83 61204994.66 55482977.09 55770945.35 62892742.38 40829393.16 35386261.92 65044512.73 58374260.16 66154828.13 85352328.74 77216223.22 76002643.71 76002835.02

1229NEG PE(18:1/18:1)-H PE(36:2)-H PE (18:1/18:1) (18:1) (18:1) C41 H77 O8 N1 P1 742.5392305 9.723710478 0.113322983 0.838822245 0.429355781 0.603411301 13635024.02 14539146.68 32142709.5 14281231.37 21504143.1 30560165.78 12936307.42 21935669.73 16781301.18 13508726.34 14221242.48 26864008.79 20679725.47 25102890.31 20814662.3

122POS DG(16:0/18:1)+H DG(34:1)+H DG (16:0/18:1) (16:0) (18:1) C37 H71 O5 595.5296015 11.85623429 0.026515381 0.974819537 0.925034263 0.119062748 23046895.15 27606926.63 17229746.09 29060121.51 24724841.85 19602548.85 5274215.483 45753641.88 31008882.64 15676318.09 14484514.89 25516235.91 30955202.09 32423291.96 31002856.47

1230NEG PE(18:1/18:1)-H PE(36:2)-H PE (18:1/18:1) (18:1) (18:1) C41 H77 O8 N1 P1 742.5392305 10.73154854 0.001573678 1.118555459 0.375656596 5.839835071 2695067097 2645714058 2860623134 3315409570 3562538231 2941752632 1670842659 3942276418 3268396623 3531172033 3950548885 3794368439 3909086518 3898456832 3898444942

1231NEG PG(16:1/18:2)-H PG(34:3)-H PG (16:1/18:2) (16:1) (18:2) C40 H72 O10 N0 P1 743.4868615 8.140034912 0.004169808 1.492624699 0.235095163 0.515114884 6815180.154 5595233.117 9157784.315 8417581.977 8883184.737 8167588.697 3518462.382 13525426.35 11449493.92 8325478.518 8347892.243 25041167.35 13519517.66 13422702.63 13421987.83

1232NEG PE(16:1p/22:6)-H PE(38:7p)-H PE (16:1p/22:6) (16:1p) (22:6) C43 H71 O7 N1 P1 744.4973655 9.251865027 0.013591278 1.182278372 0.457312309 0.256171715 7645662.776 6820204.162 9808607.924 6686508.516 8995693.016 15721678.49 3400171.178 14383363.46 11128370.47 10170337.79 11772081.37 14972990.48 13030798.17 13025277.02 12723772.5

1233NEG PE(36:1)-H PE(36:1)-H PE (36:1) (36:1) C41 H79 O8 N1 P1 744.5548805 10.42932863 0.002083369 1.052279745 0.666844216 0.2882954 68618115.51 44421193.93 65070634.46 63621016 64886293.85 68980672.69 40199001.43 73488642.95 80892642.1 59804929.21 60605713.15 80243161.55 74703254.64 74704883.36 74973959.68

1234NEG PE(18:0/18:1)-H PE(36:1)-H PE (18:0/18:1) (18:0) (18:1) C41 H79 O8 N1 P1 744.5548805 11.36151526 0.001576824 1.14804033 0.273618528 4.109565489 2260809170 1987334707 2569705533 2335891138 2219272929 2019840677 1414086308 3354647920 2626778046 2342573438 2618243614 3019207371 3063719569 3056019807 3064914517

1235NEG PG(16:1/18:1)-H PG(34:2)-H PG (16:1/18:1) (16:1) (18:1) C40 H74 O10 N0 P1 745.5025115 8.890819415 0.040733977 1.32277585 0.278868882 0.994300413 37148638.23 32078640.67 49658879.06 44593936.22 47377612.5 42261952.48 12251676.25 78361616.8 55295664.24 47401932.4 47269646.58 94240036.08 63750858.74 63760841.01 59361028.08

1236NEG PG(16:1/18:1)-H PG(34:2)-H PG (16:1/18:1) (16:1) (18:1) C40 H74 O10 N0 P1 745.5025115 9.275493955 0.081267168 1.447019781 0.319210135 0.350110973 5115781.651 5364433.201 4105269.094 7954086.832 7704850.866 8225211.112 2733278.593 11881699.98 10189003.46 7229870.439 3410061.662 20222405.43 8245807.751 8161609.836 9412996.451

1237NEG SM(d16:1/18:1)+HCOO SM(d34:2)+HCOO SM (d16:1/18:1) (d16:1) (18:1) C40 H78 O8 N2 P1 745.5501295 8.619331637 0.004344208 1.082244958 0.715913027 1.437922339 216150410.6 153999551.7 206874463.2 155627983.2 185228568 194123526.1 80789691.92 256540270.2 268731566.4 130032472.8 141293821.7 326073443.3 261282033.5 259332268.3 259319511.2

1238NEG PS(17:1/16:0)-H PS(33:1)-H PS (17:1/16:0) (17:1) (16:0) C39 H73 O10 N1 P1 746.4977605 9.243701548 0.003781391 0.914242791 0.702755238 0.342142686 7672908.903 6566689.293 13752896.73 12458614.93 8257423.252 17389603.24 6587658.108 12992394.8 9928088.652 6310979.759 7832812.043 16777811.27 12684457.92 12684521.32 12601592.77

1239NEG PS(33:1)-H PS(33:1)-H PS (33:1) (33:1) C39 H73 O10 N1 P1 746.4977605 7.746 0.058555118 0.9355349 0.873292338 0.443290592 7047153.024 6194060.06 7840457.97 9830633.261 5585706.388 9795718.46 1359958.93 7733177.862 8030803.099 1056292.576 4479256.697 20649910.13 11085087.99 10046797.75 10017830.65

123POS Cer(d18:0/20:0)+H Cer(d38:0)+H Cer (d18:0/20:0) (d18:0) (20:0) C38 H78 O3 N1 596.5976205 12.224 0.040899223 0.838561958 0.269526011 0.204649348 6410875.755 7524738.468 6061338.461 7216730.632 7060412.452 9863087.644 6493602.01 5631570.271 6375068.945 4277362.995 4274589.923 9959568.79 9396619.247 9390550.179 8743518.925

1240NEG PS(33:1)-H PS(33:1)-H PS (33:1) (33:1) C39 H73 O10 N1 P1 746.4977605 8.327 0.005887231 1.129671104 0.763321441 0.436225161 13149217.89 6570078.053 13288920.06 7709183.059 7100558.248 20162651.98 4156857.332 10669714.96 15848247.4 6765003.033 7072642.741 32283264.5 15711393.66 15873475.59 15871911.01

1241NEG PE(16:0p/22:6)-H PE(38:6p)-H PE (16:0p/22:6) (16:0p) (22:6) C43 H73 O7 N1 P1 746.5130155 9.928089947 0.011177715 1.321234437 0.177576588 3.428640837 358141929.1 314999281.2 433669358 342370919.3 514264450 474054910 165835408.3 510383644.9 554976661.5 538308480.6 700319917.5 750685947.4 647433031.3 647463397.2 660064442.6

1242NEG PG(16:0/18:1)-H PG(34:1)-H PG (16:0/18:1) (16:0) (18:1) C40 H76 O10 N0 P1 747.5181615 9.655604855 0.014127076 1.2104418 0.235925998 1.520909005 158222271.7 90807918.92 186318302.6 133047734.2 129674101.5 148333997.2 86397261.15 211824461.1 171750258.4 155694517.2 178979890.7 219876787.5 204467571.4 199549148.3 199540454.4

1243NEG SM(d18:1/16:0)+HCOO SM(d34:1)+HCOO SM (d18:1/16:0) (d18:1) (16:0) C40 H80 O8 N2 P1 747.5657795 9.514950268 0.002096683 1.05819847 0.693847967 3.100848219 2764235032 2271920101 2743736942 2395771098 2822903082 2447156734 1376666949 3285161550 3406725379 2346421179 2264868543 3664796841 3203681331 3203777541 3192109260

1244NEG PC(16:1/14:0)+HCOO PC(30:1)+HCOO PC (16:1/14:0) (16:1) (14:0) C39 H75 O10 N1 P1 748.5134105 8.649751515 0.002999468 0.986193404 0.960692824 2.733730287 423304136.6 252410299.7 389428687.5 356485641.7 272785042.7 470987427.8 151818701.3 333709278.4 471532885.7 200828485.8 223836725.5 753778338.8 474294017.9 471462343.6 472735252.4

1245NEG PE(18:0p/20:5)-H PE(38:5p)-H PE (18:0p/20:5) (18:0p) (20:5) C43 H75 O7 N1 P1 748.5286655 10.32142776 0.005777168 1.094186021 0.523907309 0.869360565 827262083.5 615121815.6 884990236.3 749802511.8 919145671 985576377.2 549588405.6 910725922 876426736 838836664.2 922074479.3 1353471705 1032380454 1042741078 1042818860

1246NEG PE(18:0p/20:5)-H PE(38:5p)-H PE (18:0p/20:5) (18:0p) (20:5) C43 H75 O7 N1 P1 748.5286655 10.58661377 0.005131322 1.141923564 0.286230381 1.694879556 355599416.7 301787222.5 367973320.7 335043799.6 385878534.9 374693856.1 207613727.5 467801317.7 420876543.2 390328297.8 425570981.2 509801776.6 478222762.7 474023984 473971487.6

1247NEG SM(d18:0/16:0)+HCOO SM(d34:0)+HCOO SM (d18:0/16:0) (d18:0) (16:0) C40 H82 O8 N2 P1 749.5814295 9.859811907 0.003486391 1.032958675 0.83704389 1.499228596 260330592 205744564.3 234790056 237288463.6 255686454.6 217943550.5 124437038.3 290146079.8 325080389.5 182636800.8 196062533.2 339951358.7 285712818.1 285740618 284004961.1

1248NEG PC(16:0/14:0)+HCOO PC(30:0)+HCOO PC (16:0/14:0) (16:0) (14:0) C39 H77 O10 N1 P1 750.5290605 9.518406185 0.001407946 0.903163706 0.544855791 4.989193415 786674207.4 665926109.9 748508459 735064493.8 670222104.2 774074912 361682808.2 740648171.2 888166295.7 461708857.2 465082104.9 1038993542 852341129.3 850265559.5 850266351.9

1249NEG PE(38:4p)-H PE(38:4p)-H PE (38:4p) (38:4p) C43 H77 O7 N1 P1 750.5443155 10.85158129 0.004687363 1.2731819 0.159823722 4.356967538 854443411.2 818300913.5 888662278.1 885790481.6 1094612557 951600910.2 560053819.1 1417410925 1102185284 1040752498 1146360692 1727347668 1250509836 1250662413 1240461191

124POS PC(24:4p)+H PC(24:4p)+H PC (24:4p) (24:4p) C32 H57 O7 N1 P1 598.3867185 2.825509565 0.068166291 1.392023033 0.402427423 0.061489018 1007960.935 1392756.971 1194532.987 649558.7002 1955760.925 968623.1846 1124083.153 2509785.664 3736688.517 493631.7497 741184.5957 1374309.084 2268022.71 2555172.817 2283586.287

1250NEG PE(17:0/20:4)-H PE(37:4)-H PE (17:0/20:4) (17:0) (20:4) C42 H75 O8 N1 P1 752.5235805 10.16999438 0.049659599 1.08807065 0.672256001 0.242392664 47845259.01 31143705.62 73742138.2 29552549.2 49262065.27 50997058.71 29023838.99 70419108.57 46737375.91 35089433.3 62164104.04 63992641.15 49310831.56 53763863.9 49474020.77

1251NEG PE(38:3p)-H PE(38:3p)-H PE (38:3p) (38:3p) C43 H79 O7 N1 P1 752.5599655 11.3423099 0.00393971 1.069441162 0.77027396 0.29536306 94322141.6 49412844.6 59633341.53 70073547.12 75544350.85 109977127.7 29423590.49 137810351.7 85021612.13 70095201.3 63472766.32 105010780.1 87526859.15 87517898.13 88120923.02

1252NEG PS(34:4)-H PS(34:4)-H PS (34:4) (34:4) C40 H69 O10 N1 P1 754.4664605 9.05094623 0.025852972 0.848229256 0.430885119 0.414373573 6162031.459 4324417.732 8763081.659 10236100.19 7568942.823 11375206.01 5438526.097 5388928.02 7197994.099 6232358.467 4997355.321 11824394.13 9190429.118 9190492.582 8784976.67

1253NEG PE(17:0/20:3)-H PE(37:3)-H PE (17:0/20:3) (17:0) (20:3) C42 H77 O8 N1 P1 754.5392305 10.56937432 0.045112491 0.884181236 0.721595377 0.360850618 17374710.87 11314741.69 41319219.38 38778837.99 16280315.08 13592501.88 11053562.32 22464278.11 43489291.24 13494839.19 14812357.47 17286530.85 20975315.51 19377517.42 20974338.18

1254NEG PE(38:2p)-H PE(38:2p)-H PE (38:2p) (38:2p) C43 H81 O7 N1 P1 754.5756155 11.7718443 0.040391407 1.085786431 0.787220024 0.127475311 19532672.8 13724689.17 21270139.85 38122136.21 42246390.48 38334316.91 17462551.62 51990747.72 22059758.68 17724085.56 21481593.5 57372421.37 37298339.03 34653013.16 34990227.66

1255NEG PS(34:3)-H PS(34:3)-H PS (34:3) (34:3) C40 H71 O10 N1 P1 756.4821105 9.872664198 0.002242507 0.86273672 0.041334303 0.553524403 14906698.52 10716643.7 11572146.91 12549376.78 13437569.24 10731631.09 11076149.57 9879115.809 10410271.13 11204086.65 9937338.828 11261417.09 10855521.26 10830399.31 10879075.72

1256NEG PC(12:0e/20:4)+HCOO PC(32:4e)+HCOO PC (12:0e/20:4) (12:0e) (20:4) C41 H75 O9 N1 P1 756.5184955 8.249207938 0.002928656 1.771765236 0.029391139 0.46335773 1857274.584 1760060.506 2287928.058 2351181.944 1689884.613 1464539.156 1121404.965 4202248.15 4064823.641 2645323.875 3193472.06 4990108.071 3840228.626 3859451.2 3839663.228

1257NEG PE(19:1/18:1)-H PE(37:2)-H PE (19:1/18:1) (19:1) (18:1) C42 H79 O8 N1 P1 756.5548805 11.03262073 0.000834538 1.127251456 0.438427218 0.741624581 66353151.47 69389217.02 75743233.67 81851125.34 95669029.51 67629855.19 37542000.79 120689202.6 93908095.62 81360089.27 93298285.49 87945484.96 103529566.5 103529938.1 103679472.5

1258NEG PE(37:2)-H PE(37:2)-H PE (37:2) (37:2) C42 H79 O8 N1 P1 756.5548805 10.08872623 0.024642023 0.973044368 0.898313483 0.248645304 6111756.874 4055485.482 3721723.696 5475100.578 6768878.04 5207381.783 2750039.184 2439556.6 7158547.946 5077572.559 4690933.701 8378878.156 7713035.79 7391673.405 7394225.228

1259NEG PE(38:1p)-H PE(38:1p)-H PE (38:1p) (38:1p) C43 H83 O7 N1 P1 756.5912655 12.28578975 0.006054046 1.122075191 0.535298792 0.247989981 48686070.36 40425020.01 55454682.4 62781505.62 63709331.18 62921259.02 28979058.5 81795824.18 60929968.23 48047908.82 58076588.64 96918932.39 78377484.5 77434591.46 77884921.9

125POS DG(17:1/16:0)+NH4 DG(33:1)+NH4 DG (17:1/16:0) (17:1) (16:0) C36 H72 O5 N1 598.5405005 11.54313763 0.007283333 1.002565694 0.991318782 0.101331056 21513274.97 24732094.95 18605054.71 24700479.91 23193900.16 18801421.83 6196502.12 39816500.05 29801596.53 16491609.61 14474310.37 25103215.25 32834160.86 33249649.02 32833239.56

1260NEG PS(16:1/18:1)-H PS(34:2)-H PS (16:1/18:1) (16:1) (18:1) C40 H73 O10 N1 P1 758.4977605 8.894927632 0.156503992 1.134176943 0.764323016 0.174781152 14060363.79 11312428.78 37135420.06 41079816.42 21366539.86 29876131.91 13958268.96 16620371.22 18207650.55 20047513.95 28110511.5 78661094.7 30405004.28 22832285.22 30368222.26

1261NEG PS(16:1/18:1)-H PS(34:2)-H PS (16:1/18:1) (16:1) (18:1) C40 H73 O10 N1 P1 758.4977605 9.32 0.068269947 0.783203377 0.563818116 0.354444447 4914159.673 2671709.027 6312682.015 4119381.205 4491237.907 17081154.83 4043241.369 6137046.651 4448341.583 3057201.879 3356342.252 9965102.233 7354447.553 6550769.939 6545953.448

1262NEG PE(19:0/18:1)-H PE(37:1)-H PE (19:0/18:1) (19:0) (18:1) C42 H81 O8 N1 P1 758.5705305 11.58574639 0.012864275 1.107393661 0.591657835 0.288196122 23642907.36 28643551.82 26311988.53 38110608.09 28679272.38 20223470.28 12675652.63 48956045.9 32066105.08 27319649.44 32232283.9 30147718.89 36777597.61 35976334.08 35964062.09

1263NEG PG(17:1/18:1)-H PG(35:2)-H PG (17:1/18:1) (17:1) (18:1) C41 H76 O10 N0 P1 759.5181615 9.318998078 0.002268763 1.475663907 0.124952716 0.603545493 5500702.902 6205557.144 6554693.148 7544800.856 7242482.599 5881281.642 1903016.232 15526568.67 8868845.451 9446969.234 10245930.33 11455555.15 11593052.75 11547645.92 11547585.3

1264NEG SM(d17:1/18:1)+HCOO SM(d35:2)+HCOO SM (d17:1/18:1) (d17:1) (18:1) C41 H80 O8 N2 P1 759.5657795 9.16599648 0.004618423 1.30993836 0.19163603 0.41005849 8320442.52 6412516.266 7849837.485 5588580.024 7880099.466 6849466.088 3354924.469 12560712.62 12972252.67 7061579.591 8735941.251 11512178.81 10840030.61 10754018.84 10840971.95

1265NEG PE(20:5/18:2)-H PE(38:7)-H PE (20:5/18:2) (20:5) (18:2) C43 H71 O8 N1 P1 760.4922805 8.794016575 0.012042455 1.002260363 0.990154976 0.505467712 51603752.83 36967965.73 55141989.49 47420620.26 42930637.3 50138946.52 18583884.69 50731960.06 51718305.56 39319180.13 45385613.31 79107372.48 66474801.95 66485694.42 65103200.11

1266NEG PS(16:0/18:1)-H PS(34:1)-H PS (16:0/18:1) (16:0) (18:1) C40 H75 O10 N1 P1 760.5134105 8.363392122 0.00968316 1.061990995 0.84625826 0.259440816 8425868.472 4552354.277 8533487.984 7264933.361 4297487.472 10178973.31 1183949.188 7109828.551 9765370.152 5528981.878 6283876.39 16062401.74 9750153.807 9919006.698 9911815.649

1267NEG PS(16:0/18:1)-H PS(34:1)-H PS (16:0/18:1) (16:0) (18:1) C40 H75 O10 N1 P1 760.5134105 10.59735098 0.059907694 1.155007768 0.509506291 0.298772985 8113494.922 9117231.043 14348337.57 16221509.52 10373539.14 19788918.87 9819351.215 15423862.85 12652304.19 24269509.9 9740995.992 18141882.38 15700644.8 17397607.07 15717826.46

1268NEG PS(16:0/18:1)-H PS(34:1)-H PS (16:0/18:1) (16:0) (18:1) C40 H75 O10 N1 P1 760.5134105 9.645899415 0.011212061 1.019423303 0.918726018 0.335564389 168529165.3 161850798.3 289463049.5 299333144.9 202317224.5 391017143.3 185458176.3 349452678.6 222151977.7 190293758.9 261398725.7 333133158.9 247904381.1 252727429.5 247860369

1269NEG PC(16:0p/16:1)+HCOO PC(32:1p)+HCOO PC (16:0p/16:1) (16:0p) (16:1) C41 H79 O9 N1 P1 760.5497955 10.05952601 0.003466968 0.988693911 0.959925059 0.749559037 40707854.14 24139055.48 38465947.01 43927023.18 71402768.44 50122260.22 18216608.28 31216686.72 36668428.67 54954140.23 63832308.4 60838056.21 68358498.98 68270506.14 67912235.88

126POS LPI(18:0)+H LPI(18:0)+H LPI (18:0) (18:0) C27 H54 O12 N0 P1 601.3347445 2.86 0.009423504 1.564747579 0.311955699 0.088369508 975020.5571 574265.7584 2843162.396 1085018.919 1732336.927 1796220.3 5639374.277 1275078.019 760898.9675 2560783.201 2582875.141 1273145.99 1591891.446 1578532 1562208.741

1270NEG SM(d16:1+hO/18:1)+HCOO SM(d34:2+hO)+HCOO SM (d16:1+hO/18:1) (d16:1+hO) (18:1) C40 H78 O9 N2 P1 761.5450445 8.277038033 0.006375272 1.081179357 0.785617097 0.263653048 3352055.793 3330279.312 4334220.328 3426435.917 3076057.561 3927512.166 1130204.172 5373505.59 6425394.273 1604998.116 2155648.437 6497828.52 5155300.291 5154943.186 5212255.573

1271NEG SM(d17:0/18:1)+HCOO SM(d35:1)+HCOO SM (d17:0/18:1) (d17:0) (18:1) C41 H82 O8 N2 P1 761.5814295 9.976284105 0.006725677 1.203922998 0.215329752 0.84560631 97355595.07 82883882.76 92875953.81 69816726.89 89358918.84 78656720.52 51173806.98 131759552.4 130276189.2 88139863.46 98119281.67 115673110.8 108842519.9 107589424 108862376.2

1272NEG PE(16:0/22:6)-H PE(38:6)-H PE (16:0/22:6) (16:0) (22:6) C43 H73 O8 N1 P1 762.5079305 9.511221173 0.00615456 1.25948447 0.141582169 2.421304087 214216137.5 172108166.3 235014522.3 177287665.1 238953815.7 216379343.6 115029813.5 305638422.6 237358122.6 290565222 315262087.3 315489038.1 311862487.3 308569497.4 311901948.3

1273NEG PS(34:0)-H PS(34:0)-H PS (34:0) (34:0) C40 H77 O10 N1 P1 762.5290605 6.982 0.009963674 1.093592795 0.615071697 0.050800744 286016.6489 279971.1164 356784.9657 358534.6575 228971.0033 212067.4347 354004.357 422913.7 155457.0753 256741.5106 380504.1797 349407.1416 349097.0587 355308.2367

1274NEG PC(15:0/16:1)+HCOO PC(31:1)+HCOO PC (15:0/16:1) (15:0) (16:1) C40 H77 O10 N1 P1 762.5290605 9.155750277 0.004710092 1.126684188 0.61057538 1.200872003 137516569.5 87974611.56 128414142 107974683.8 98587193.56 133357038.7 53739809.43 148989909.8 176411001 81264524.56 94916932.58 226398622.3 160826551.9 159523071.8 159520147

1275NEG PC(16:0e/16:1)+HCOO PC(32:1e)+HCOO PC (16:0e/16:1) (16:0e) (16:1) C41 H81 O9 N1 P1 762.5654455 10.18766025 0.017293661 1.008590481 0.979458381 1.321030559 88204614.73 57525157.97 79758549.8 84275506.31 91697873.28 119456521.2 30556219.58 91319726.92 112644247.7 39542466.25 45549505.26 205780995.5 103257486.7 106412954.4 106413907

1276NEG PC(16:0p/16:0)+HCOO PC(32:0p)+HCOO PC (16:0p/16:0) (16:0p) (16:0) C41 H81 O9 N1 P1 762.5654455 10.77023492 0.011155396 1.140423183 0.498436096 0.491664706 54048660.99 60090517.35 45385908.27 62496454.37 107083301.4 69041331.49 28724156.81 86549443.28 88161936.7 77360705.01 75525005.73 97733879.54 87071559.72 85408372.66 85412802.71

1277NEG SM(d18:1+hO/16:0)+HCOO SM(d34:1+hO)+HCOO SM (d18:1+hO/16:0) (d18:1+hO) (16:0) C40 H80 O9 N2 P1 763.5606945 8.045 0.013771166 1.150663027 0.584454035 0.231183947 4362093.44 3406968.71 4564477.651 4298611.085 3926867.56 3816186.944 1347620.677 6593245.818 6518698.478 2586931.708 3218703.347 7782447.591 5875875.189 5736823.283 5875867.071

1278NEG PE(18:0/20:5)-H PE(38:5)-H PE (18:0/20:5) (18:0) (20:5) C43 H75 O8 N1 P1 764.5235805 10.19051833 0.003298155 1.082980873 0.545226889 0.369520417 471802038.8 336449434.3 525359486 363389914.3 314174492.2 383820980.4 284298464.4 499847189.6 462976036 365956726.7 414409508.3 566247309.3 537505144.4 537520656.7 534448184.3

1279NEG PE(18:0/20:5)-H PE(38:5)-H PE (18:0/20:5) (18:0) (20:5) C43 H75 O8 N1 P1 764.5235805 9.933053363 0.009118256 1.193352214 0.236371546 4.407919709 963262360.4 828980848.4 959895465.7 849536394.2 1248457466 893963324.4 520178607.6 1269694678 1108081217 1319544079 1446703328 1190527602 1281221945 1259162723 1276419964

127POS LPC(24:1)+H LPC(24:1)+H LPC (24:1) (24:1) C32 H65 O7 N1 P1 606.4493185 6.986476998 0.020618609 1.042618654 0.832191156 0.092140004 5106708.095 3781094.574 5381848.468 5204495.191 5628477.761 5329218.245 3379871.45 7003686.121 7231190.891 2961103.914 3175145.551 7977808.557 7333568.003 7600387.317 7337005.932

1280NEG PE(16:0/22:4)-H PE(38:4)-H PE (16:0/22:4) (16:0) (22:4) C43 H77 O8 N1 P1 766.5392305 10.30339033 0.018590278 1.048340683 0.722392662 0.470463288 94026216.39 80221153.04 100055361.8 102592753.8 92914665.1 93397999.42 50889872.93 124377668.5 114505327.1 87714418.61 85407010.81 127539718.4 133772047.3 129565331.5 129545139.5

1281NEG PE(18:0/20:4)-H PE(38:4)-H PE (18:0/20:4) (18:0) (20:4) C43 H77 O8 N1 P1 766.5392305 10.6945671 0.006375929 1.10175964 0.310181077 0.091970298 1262643404 1024911190 1340484545 1031071885 987986320.5 1146955140 922727455.7 1461679918 1446341626 1096601636 1157093637 1400968546 1449021000 1456582485 1438213904

1282NEG PG(16:0/20:5)-H PG(36:5)-H PG (16:0/20:5) (16:0) (20:5) C42 H72 O10 N0 P1 767.4868615 8.227790488 0.018709647 1.079534486 0.810441308 0.121636525 1588529.478 890623.3636 2429575.352 1782979.103 988341.872 1252202.845 409154.6482 2308610.086 1640138.569 1016472.751 1026168.593 3242129.438 2272152.038 2199455.334 2272456.698

1283NEG PG(16:1/20:4)-H PG(36:5)-H PG (16:1/20:4) (16:1) (20:4) C42 H72 O10 N0 P1 767.4868615 7.954151097 0.025114848 1.348993899 0.300266594 0.182914085 1575045.844 1140645.066 1697773.555 1588133.41 1495251.046 1473388.45 543289.67 2336149.378 2206285.678 1463810.132 1572558.615 3978702.013 2540340.278 2652179.624 2539783.262

1284NEG PS(36:3p)-H PS(36:3p)-H PS (36:3p) (36:3p) C42 H75 O9 N1 P1 768.5184955 11.085 0.004611337 0.987282017 0.892223997 1.004881097 58964122.45 60265042.21 60778551.09 76197508.39 75283502.83 68098179.58 49461453.31 66323833.84 55364742.8 79988070 80779691.37 62587175.62 63305771.19 63298471.7 63809030.07

1285NEG PE(18:0/20:3)-H PE(38:3)-H PE (18:0/20:3) (18:0) (20:3) C43 H79 O8 N1 P1 768.5548805 10.99621584 0.004778367 1.086149428 0.58924236 0.676618168 270527095.5 187932465.3 257789862.8 219241238.8 203500194.1 189274128.4 135474200.8 311362400 331047619.2 191855148.7 203278349.7 269676534.7 294360088.4 291940243.4 291934212.9

1286NEG PG(16:0/20:4)-H PG(36:4)-H PG (16:0/20:4) (16:0) (20:4) C42 H74 O10 N0 P1 769.5025115 8.842101115 0.015435871 1.142422789 0.582543658 0.299655963 8974473.451 6109064.974 10681567.15 7849577.546 5412811.68 8316483.52 2265456.035 11880119.91 11788511.75 6515877.883 7300078.709 14336795.48 11866787.5 11872232.04 11555008.12

1287NEG PG(18:2/18:2)-H PG(36:4)-H PG (18:2/18:2) (18:2) (18:2) C42 H74 O10 N0 P1 769.5025115 8.362991632 0.015499329 1.622578342 0.100624708 0.496939196 3882239.433 3444940.277 4115903.852 3603552.038 3943827.972 4142130.729 2027790.447 7394301.087 7400376.276 4349496.706 4947285.688 11415196.3 7199582.396 7202126.418 7009270.033

1288NEG PS(35:3)-H PS(35:3)-H PS (35:3) (35:3) C41 H73 O10 N1 P1 770.4977605 8.317 0.019300344 1.215667515 0.519864602 0.295047618 4957148.966 3641249.214 5095282.35 3820963.436 3851284.045 4805006.957 2224993.548 6470380.567 7176723.677 2861008.681 2409755.563 10672293.43 6879789.668 6874855.776 7109776.944

1289NEG PE(20:1/18:1)-H PE(38:2)-H PE (20:1/18:1) (20:1) (18:1) C43 H81 O8 N1 P1 770.5705305 11.34605429 0.003564754 1.271500758 0.161278866 1.798467197 148107424.7 145968831.9 169887537.2 102081631.3 207195220.4 148633508.1 87053795.06 259173382.6 201464327.4 189210444.4 213318625.8 221943109.7 236199347.8 236208477.3 234748527.3

128POS PC(23:0)+H PC(23:0)+H PC (23:0) (23:0) C31 H63 O8 N1 P1 608.4285835 5.302656687 0.017039092 0.428133235 0.090737215 0.109089639 1233678.809 743656.8559 2128943.454 1633067.099 276507.5461 542939.7692 8596.808481 758291.1703 903063.3145 237564.4152 42949.79655 857571.9882 1073466.452 1073550.677 1105505.249

1290NEG PE(38:2)-H PE(38:2)-H PE (38:2) (38:2) C43 H81 O8 N1 P1 770.5705305 10.50213152 0.0568625 1.079233412 0.622801532 0.221133374 30547230.63 24691177.23 27316899.32 28918730.01 40280131.29 30679807.76 16312420.07 39526217.46 32766893.65 29859970.52 31272385.43 47150955.45 40318386.27 40347671.91 36487026.21

1291NEG PG(18:1/18:2)-H PG(36:3)-H PG (18:1/18:2) (18:1) (18:2) C42 H76 O10 N0 P1 771.5181615 9.066245775 0.005089396 1.43066536 0.161417716 0.990137431 21327460.39 16234457.48 25257531.84 21257080.7 23564490.25 23587509.23 10925366.29 47768902.57 35896685.9 22820534.03 22818847.26 47513775.88 39156829.44 38811999.86 39155474.03

1292NEG PC(18:2/14:1)+HCOO PC(32:3)+HCOO PC (18:2/14:1) (18:2) (14:1) C41 H75 O10 N1 P1 772.5134105 8.449533712 0.022591245 1.02272093 0.93757908 0.615711737 23546333.75 16889202.01 23526329.81 17075796.65 16737395.84 25763262.13 8110643.696 19133618.01 28160919.57 11177685.61 14748806.22 45013552.57 28787334.62 27675362.45 28787230.34

1293NEG PC(18:2/14:1)+HCOO PC(32:3)+HCOO PC (18:2/14:1) (18:2) (14:1) C41 H75 O10 N1 P1 772.5134105 8.148935415 0.008390841 1.10246489 0.77205352 0.451956956 16630074.5 7780740.393 10525014.88 10147758.58 6859352.283 13058073.81 4580287.432 11667257.88 15403436.22 6369147.391 6620585.583 27020621.73 16900660.69 16657645.27 16657143.99

1294NEG PE(18:1p/22:6)-H PE(40:7p)-H PE (18:1p/22:6) (18:1p) (22:6) C45 H75 O7 N1 P1 772.5286655 10.09017554 0.004785867 1.168436376 0.303884048 1.98126811 248624244.5 260403687.8 265890934.6 282281498.6 385271642.4 318666576 144693259.8 400550541.8 356042873.2 358123041.6 402594768.7 395773899 400395654 398180361.4 396600012.9

1295NEG PE(38:1)-H PE(38:1)-H PE (38:1) (38:1) C43 H83 O8 N1 P1 772.5861805 11.1576683 0.015379031 1.067357649 0.720089188 0.245029559 32070718.36 28363461.95 29321887.59 27711484.65 30033828.73 26628393.36 10414988.54 42403793.53 44355329.48 23001169.22 29491840.48 36191625.63 42488658.31 41365870.38 42486668.9

1296NEG PE(18:0/20:1)-H PE(38:1)-H PE (18:0/20:1) (18:0) (20:1) C43 H83 O8 N1 P1 772.5861805 11.95660602 0.004447768 1.192576688 0.308122669 0.83821686 46217897.22 38531916.27 52884324.2 58182541.06 46096913.83 43810169.57 26112227.44 86988036.18 56174875.96 50223090.38 57358916.46 63890351.67 69851291.41 70389642.32 69849012.09

1297NEG PE(38:1)-H PE(38:1)-H PE (38:1) (38:1) C43 H83 O8 N1 P1 772.5861805 9.329298557 0.030764818 1.102187551 0.68875529 0.168253625 4900815.663 3506371.504 5319169.693 4094428.415 4618745.194 4047177.189 1335930.518 8361338.328 6486359.896 2960925.021 3850894.375 6197871.313 6668483.816 6658536.542 7024899.868

1298NEG PG(18:1/18:1)-H PG(36:2)-H PG (18:1/18:1) (18:1) (18:1) C42 H78 O10 N0 P1 773.5338115 9.780623453 0.013373232 0.997940516 0.993503131 0.966399273 109740271.9 97800057.06 156071465.7 176899589.1 97368043.04 107723959.3 30910579.45 180156980.3 162150958.8 74312610.98 95445437.02 201091261.5 190051349.7 189986649.9 194454323.7

1299NEG SM(d18:1/18:1)+HCOO SM(d36:2)+HCOO SM (d18:1/18:1) (d18:1) (18:1) C42 H82 O8 N2 P1 773.5814295 9.639828685 0.007649934 1.11863673 0.486661008 0.100338262 15486447.73 14949550.6 16013849.07 12679508.84 18445450.96 12516308.71 6958191.883 21340797.95 22785474.89 15522284.74 15929095.55 18243386.3 19788958.97 20052869.18 20054128.15

129POS LPC(24:0)+H LPC(24:0)+H LPC (24:0) (24:0) C32 H67 O7 N1 P1 608.4649685 8.273526105 0.013039158 1.118571488 0.643504518 0.186658387 15142819.17 9125667.576 16868713.94 16487765.49 14366692.22 18045890.64 8940425.136 23120078.55 20878845.67 9348203.224 9680967.893 28744914.77 24687100.91 24685507.55 24132944.27

12POS WE(21:2)+NH4 WE(21:2)+NH4 WE (21:2) (21:2) H42 C21 O2 N1 340.3210055 2.265897102 0.005372926 0.888278601 0.786142631 0.071466932 2190792.426 16541245.18 3460822.764 24458717.72 11137883.17 5946070.579 253577.1732 15831956.04 8906002.699 8891279.88 6911964.375 15820128.92 14579415.63 14435266.97 14456347.9

1300NEG PS(17:0/18:1)-H PS(35:1)-H PS (17:0/18:1) (17:0) (18:1) C41 H77 O10 N1 P1 774.5290605 10.07767804 0.012328755 1.119354951 0.43760552 0.813018583 81341310.5 93243605.74 111443497.6 119831634 128052156.9 149826723.9 69234115.01 165569580.9 126639626.6 118492016.8 136284989.3 149126226.1 129960797.9 127232384.5 127216809.4

1301NEG PS(35:1)-H PS(35:1)-H PS (35:1) (35:1) C41 H77 O10 N1 P1 774.5290605 9.357498352 0.016893372 1.065932364 0.788053206 0.868339777 101489359 53800434.2 91422825.15 51306272.4 49941602.42 91022218.41 48274069.79 77464538.97 92193672.06 51344652.8 56886258.56 141762687.2 95632087.53 92909435.36 95729432.09

1302NEG PC(16:1/16:1)+HCOO PC(32:2)+HCOO PC (16:1/16:1) (16:1) (16:1) C41 H77 O10 N1 P1 774.5290605 8.824870032 0.021539092 1.026134709 0.92087043 2.329894864 529571338.2 299589061.9 512630559.3 416079337.6 443703588.1 638637934.2 192782817.4 448739859.6 632457838 276556712.1 411245554.9 952657146 662833197.9 638543680.2 663110967.1

1303NEG PE(18:0p/22:6)-H PE(40:6p)-H PE (18:0p/22:6) (18:0p) (22:6) C45 H77 O7 N1 P1 774.5443155 10.82101896 0.000770442 1.042915612 0.787065328 1.655877267 568771754.9 568470000.3 631793816.7 651227771.6 666287233.8 651626015.7 296129834.7 857969893.1 777326133.8 535982087.6 538422594.1 892772187.5 802290893.9 801504523.7 801072772.2

1304NEG PE(18:1p/22:5)-H PE(40:6p)-H PE (18:1p/22:5) (18:1p) (22:5) C45 H77 O7 N1 P1 774.5443155 10.34588122 0.015289716 1.017730926 0.928219985 0.575778961 82776765.11 44438985.44 83085671.54 56619333.96 68718264.27 100819699.1 58637223.22 62809632.8 66232964.77 61177318.85 65512902.46 129827494.4 86135401.91 88438276.74 86138273.46

1305NEG PG(18:0/18:1)-H PG(36:1)-H PG (18:0/18:1) (18:0) (18:1) C42 H80 O10 N0 P1 775.5494615 10.41530005 0.026643419 1.00356802 0.983197569 0.99943874 100372811.5 69789196.69 106418320.3 93939605.07 70470330.04 110116156.3 54131838.89 108704048.5 110258613.6 72003449.96 67858129.78 140116698.1 133228273.3 127260787.2 127266400.5

1306NEG SM(d36:1)+HCOO SM(d36:1)+HCOO SM (d36:1) (d36:1) C42 H84 O8 N2 P1 775.5970795 10.413 0.002807488 1.053419378 0.665089758 0.276581358 62011623.32 47074815.28 61430563.73 38885886.97 49573315.52 50341406.44 35578396.26 63725157.89 67735733.2 46525584.26 50487749.79 61788544.31 61435900.37 61735902.96 61735330.39

1307NEG PC(16:0/16:1)+HCOO PC(32:1)+HCOO PC (16:0/16:1) (16:0) (16:1) C41 H79 O10 N1 P1 776.5447105 9.611998008 0.008797108 1.031846103 0.833460068 5.294242357 2775621243 2214814738 2479105443 2352677651 2411759551 2949891514 1610408404 2727883020 3464527451 2078135481 1995370181 3791092697 2973568744 2973482916 2928447122

1308NEG PE(18:0p/22:5)-H PE(40:5p)-H PE (18:0p/22:5) (18:0p) (22:5) C45 H79 O7 N1 P1 776.5599655 11.29937682 0.012768096 1.041429643 0.788791719 0.48361766 69968781.67 62662413.12 82058934.48 68682165.3 81801291.91 94989995.28 41947734.86 102449865.8 87820345.62 61652168.07 74887079.68 110470800.6 105520096.1 104368929.7 107057564.5

1309NEG PE(18:0p/22:5)-H PE(40:5p)-H PE (18:0p/22:5) (18:0p) (22:5) C45 H79 O7 N1 P1 776.5599655 11.03914899 0.009456523 1.080499241 0.765049901 1.450183998 354293608.8 224000715.4 364570888.4 276638356 249455149 387649261.8 214020655.8 316718358.8 303706723 194279592.8 275102331.8 702235850.2 380967682.6 380967507.6 374761542.2

130POS LPC(24:0)+H LPC(24:0)+H LPC (24:0) (24:0) C32 H67 O7 N1 P1 608.4649685 6.80868224 0.021526916 1.322017212 0.337438925 0.07460209 790885.2846 1179795.93 807297.5872 1919397.943 990276.511 1347146.122 222555.677 1593180.554 2130270.404 1371559.345 1362721.693 2619838.186 2130042.619 2211047.743 2131182.779

1310NEG PC(16:0/16:0)+HCOO PC(32:0)+HCOO PC (16:0/16:0) (16:0) (16:0) C41 H81 O10 N1 P1 778.5603605 10.36486861 0.00153269 0.977984061 0.868118047 3.483653519 1139665509 1040045074 1112596666 1154209289 1485208771 1243926488 518427399.8 1259976041 1154219307 1212203075 1342177781 1530669479 1354557849 1350963543 1354554748

1311NEG PE(40:4p)-H PE(40:4p)-H PE (40:4p) (40:4p) C45 H81 O7 N1 P1 778.5756155 11.50671753 0.040351644 1.265177139 0.269201148 1.400991444 94781536.87 87992079.33 106318237.9 94562341.74 95943645.44 120918741.7 49291199.76 186218916 148993667 72257331.18 135350384.3 167648354.3 139175289.3 139159158.9 149125768.8

1312NEG PI(16:0/14:1)-H PI(30:1)-H PI (16:0/14:1) (16:0) (14:1) C39 H72 O13 N0 P1 779.4716065 7.485348255 0.002630721 0.450688183 0.106050041 0.38411173 2868538.104 650906.3443 4369241.037 2385255.721 395787.6679 4220469.813 1771594.223 1105101.663 1028162.232 251442.0597 281500.3164 2273036.092 2314681.548 2311993.509 2323634.116

1313NEG PS(36:5)-H PS(36:5)-H PS (36:5) (36:5) C42 H71 O10 N1 P1 780.4821105 9.239124807 0.013925062 0.793744571 0.0418697 0.460736292 4953062.951 4213326.08 4812446.694 5249069.695 4645896.383 5859365.85 3308325.898 4474534.819 3121185.362 3591188.405 3561973.068 5543332.864 5537116.921 5405027.706 5406344.16

1314NEG PE(40:3p)-H PE(40:3p)-H PE (40:3p) (40:3p) C45 H83 O7 N1 P1 780.5912655 11.9617481 0.019181785 1.008227383 0.968290609 0.568103204 28041468.02 16642510.47 19957614.48 19185941.95 20928337.85 23687525.53 10165710.2 28157281.98 27190710.17 13735510.13 15396773.33 34854165.47 28358524.76 27438008.26 27435521.42

1315NEG PS(18:2/18:2)-H PS(36:4)-H PS (18:2/18:2) (18:2) (18:2) C42 H73 O10 N1 P1 782.4977605 9.953011518 0.006132349 0.877299237 0.01801557 0.930625326 33024353.43 38201618.56 35721566.2 39870884.33 37493923.39 38819534.28 33314253.41 31363315.33 27604579.35 33193812.89 33190833.8 37086633.51 33138025.1 33415978.89 33019433.61

1316NEG PS(16:0/20:4)-H PS(36:4)-H PS (16:0/20:4) (16:0) (20:4) C42 H73 O10 N1 P1 782.4977605 8.78127085 0.007788676 1.226226766 0.392044855 0.184935902 3741098.733 4187599.705 6576384.423 4300309.873 2874687.393 9217433.443 4332265.371 8254609.239 5998253.682 4285438.974 5419297.674 9597493.189 6047570.303 5955168.295 6014029.029

1317NEG PE(40:2p)-H PE(40:2p)-H PE (40:2p) (40:2p) C45 H85 O7 N1 P1 782.6069155 12.26 0.015508824 1.046261456 0.80331798 0.151889039 16307635.72 11960999.48 18960839.91 20219251.03 22376274.57 22404349.76 11101751.57 24421294.19 16706245.24 15043416.93 18824705.07 31323830.63 26371985.85 25674189.83 26380784.88

1318NEG PE(20:5/20:4)-H PE(40:9)-H PE (20:5/20:4) (20:5) (20:4) C45 H71 O8 N1 P1 784.4922805 8.391212828 0.040970896 1.051169003 0.866027218 0.18063164 2783971.275 1544986.911 3558720.525 1379930.012 979355.9576 2454002.468 982629.0413 3408623.716 3434315.253 1289169.473 1212657.078 3023468.417 3313064.314 3309983.219 3552199.956

1319NEG PS(36:3)-H PS(36:3)-H PS (36:3) (36:3) C42 H75 O10 N1 P1 784.5134105 10.66205569 0.017250834 0.932473026 0.169871711 0.856820454 67638395.43 56058729.34 65212202.61 59928050.46 51964639.25 63584976.15 59110344.49 56705932.77 53018384.6 53007267.09 56515689.34 61423423.8 63508211.3 63513635.39 61631979.81

131POS DG(16:1/18:1)+NH4 DG(34:2)+NH4 DG (16:1/18:1) (16:1) (18:1) C37 H72 O5 N1 610.5405005 11.31972819 0.05689115 0.873536718 0.493855218 0.506461466 119571691.3 147505843.1 98555450.68 119921241.1 172074547 81203140.61 42214843.14 174891355 104546802.2 117361296.3 95924875.43 110457632.8 183717610.6 184023588.1 166330419.8

1320NEG PS(18:1/18:2)-H PS(36:3)-H PS (18:1/18:2) (18:1) (18:2) C42 H75 O10 N1 P1 784.5134105 8.918903373 0.00542749 0.735703042 0.158105143 1.109874042 22936913.51 20496188.89 25929516.41 20837671.53 14937294.35 30660673.11 13941619.94 21080331.67 19808549.05 9391732.298 7203681.356 28481277.09 23173118.75 22975635.35 22941628.89

1321NEG PE(18:1/21:1)-H PE(39:2)-H PE (18:1/21:1) (18:1) (21:1) C44 H83 O8 N1 P1 784.5861805 11.6630545 0.008643352 1.298535565 0.197863619 0.65341257 9665429.323 10233887.42 11788930.38 14223780.21 14767348.83 9088213.8 5626609.532 22380881.88 13387203.55 16013974.27 18848207.69 14338819.92 17456105.8 17719892.8 17720248.53

1322NEG PE(40:1p)-H PE(40:1p)-H PE (40:1p) (40:1p) C45 H87 O7 N1 P1 784.6225655 12.81351757 0.005456494 1.268777935 0.222256206 0.648288553 14702021.48 12378259.33 16945284.09 20465122.57 22289875.85 19723942.9 9468857.994 30822606.63 19642741.09 20459996.04 23267026.32 31469339.42 27689135.47 27428405.35 27689409.88

1323NEG PE(18:2/22:6)-H PE(40:8)-H PE (18:2/22:6) (18:2) (22:6) C45 H73 O8 N1 P1 786.5079305 9.009259765 0.004820683 0.973994569 0.875065171 0.448187561 26924641.09 15801071.68 25612223.27 23179916.12 18895242.74 23531430.7 10209068.99 28769314.18 27885097.1 16147308.92 19207132.54 28243318.75 35806027.37 35508810.57 35508617.34

1324NEG PS(18:1/18:1)-H PS(36:2)-H PS (18:1/18:1) (18:1) (18:1) C42 H77 O10 N1 P1 786.5290605 9.819739255 0.073315386 0.929521508 0.815950719 0.908418927 111302076 113609517.4 243573848.1 213822033.4 168855031.6 491728498 115821498.6 277950409.5 197487334.5 146919035.4 188099559.3 321968233.7 267777812.2 266433859.3 302503746.8

1325NEG PS(18:1/18:1)-H PS(36:2)-H PS (18:1/18:1) (18:1) (18:1) C42 H77 O10 N1 P1 786.5290605 10.59425175 0.057407691 0.928738914 0.733337157 0.518693471 16037709.38 11829557.9 25218531.2 37343056.29 32459050.77 32401746.57 28018533.04 32239469.72 9620770.861 23172179.07 22202768.73 28969821.36 26876144.52 24273628.33 26840627.78

1326NEG PC(16:0p/18:2)+HCOO PC(34:2p)+HCOO PC (16:0p/18:2) (16:0p) (18:2) C43 H81 O9 N1 P1 786.5654455 10.166 0.00596085 1.19327489 0.348929198 0.344430609 19321212.14 18385253.62 16780478.06 18827963.68 31662454.01 20153015.28 8941169.201 30069813.07 31721324.45 22792363.31 25903320.36 29886946.23 28374037.28 28083376.14 28082809.14

1327NEG PE(17:1/22:0)-H PE(39:1)-H PE (17:1/22:0) (17:1) (22:0) C44 H85 O8 N1 P1 786.6018305 12.175 0.064157239 1.333037798 0.145994987 0.392700229 2917185.109 2966228.031 3961585.218 4271063.55 4476293.723 2710127.923 1861733.409 6776879.283 4269374.781 4871790.15 5839849.268 4777388.879 5283191.003 4852832.256 5511915.947

1328NEG PE(18:1/22:6)-H PE(40:7)-H PE (18:1/22:6) (18:1) (22:6) C45 H75 O8 N1 P1 788.5235805 9.704468833 0.004842685 1.098116374 0.375148663 1.686087502 361989573.3 336089076.7 366389749.3 382219042.6 383660798.4 264497143.8 261813587.4 467105603.3 397087733 406205474.5 448420136.6 319751483.1 425182120.8 421435061.7 424759582.3

1329NEG PS(18:0/18:1)-H PS(36:1)-H PS (18:0/18:1) (18:0) (18:1) C42 H79 O10 N1 P1 788.5447105 11.24663037 0.001363539 1.141334466 0.536131471 0.608351399 65012489.2 70839501.23 115799168.5 158828206 125746177.9 144993510.6 79834818.82 193075384.1 128209298.2 68422736.11 152043815.6 155912731.9 138172201.9 137825161.2 137872874

132POS DG(16:0/18:1)+NH4 DG(34:1)+NH4 DG (16:0/18:1) (16:0) (18:1) C37 H74 O5 N1 612.5561505 18.809 0.041347631 1.312730399 0.134124113 0.111123878 1550146.499 969084.8745 1654797.355 1473623.307 1171220.572 1564422.981 926573.0903 1911455.041 2054389.208 1321778.217 2511187.843 2279623.567 2290147.937 2153554.055 2119130.211

1330NEG PS(18:0/18:1)-H PS(36:1)-H PS (18:0/18:1) (18:0) (18:1) C42 H79 O10 N1 P1 788.5447105 12.708 0.035777815 1.800338745 0.169762649 1.326484512 14302960.02 17383327.72 22902785.36 24094875.47 21045891.68 22433973.7 18880250.27 88191578.4 22419709.1 43463141.58 23844649.33 23136918.86 19102814.63 17979431.14 17953006.1

1331NEG PS(18:0/18:1)-H PS(36:1)-H PS (18:0/18:1) (18:0) (18:1) C42 H79 O10 N1 P1 788.5447105 10.43532879 0.006306669 1.133653922 0.392953663 2.57747341 365891262.8 514270726.6 626839642.9 711042593 532636294.9 844255569.1 511879454.6 853962145.4 567506242.5 697906693.4 838107088.4 606051773.7 606959477.9 613637937.7 613638500.1

1332NEG PC(17:1/16:1)+HCOO PC(33:2)+HCOO PC (17:1/16:1) (17:1) (16:1) C42 H79 O10 N1 P1 788.5447105 9.263902023 0.027748525 0.98365395 0.929661958 1.109373882 224920392.1 166018935.2 216865914.7 198333590.2 226849086.9 217838466.3 78821410.92 225223132.4 187869310.2 168761547.9 217194172.4 352510740.8 227523334.4 216810628.2 227627645

1333NEG PC(17:1/16:1)+HCOO PC(33:2)+HCOO PC (17:1/16:1) (17:1) (16:1) C42 H79 O10 N1 P1 788.5447105 9.562235597 0.277573976 0.958791099 0.754463612 0.274506285 7184512.821 10667575.66 6083965.222 8415331.749 6931367.098 9278088.443 4173888.084 7449520.051 8165270.475 8212674.089 9284228.517 9274120.87 14342508.42 22285486.34 14008884.37

1334NEG PC(16:0p/18:1)+HCOO PC(34:1p)+HCOO PC (16:0p/18:1) (16:0p) (18:1) C43 H83 O9 N1 P1 788.5810955 10.81256157 0.006574301 1.189763724 0.388262888 0.918446574 146130434.5 163887857.8 133268897.9 174774335.2 268681286.3 167760687.9 62943013.97 256503469.7 259049370.3 207458964.8 201847431.4 266807760.8 232898083.5 232896295.2 235559299.3

1335NEG PE(18:1/22:5)-H PE(40:6)-H PE (18:1/22:5) (18:1) (22:5) C45 H77 O8 N1 P1 790.5392305 9.950950982 0.01724762 1.070823036 0.550029591 0.667265918 81026895.72 68454016.93 47588618.31 68967177.14 72266544.76 63735530.35 46975719.09 77649488.56 59656074.15 83825693.3 77431251.33 84974164.17 84088715.37 81461455.33 81830908.06

1336NEG PE(40:6)-H PE(40:6)-H PE (40:6) (40:6) C45 H77 O8 N1 P1 790.5392305 9.129 0.032103196 0.994271077 0.986989762 0.214829437 1778871.59 1815222.748 1694964.701 1251524.059 2038852.186 2622745.237 457091.7392 2217111.742 2565892.243 903043.7728 635044.8842 4359819.714 1915523.489 2024371.891 1916142.098

1337NEG PE(18:0/22:6)-H PE(40:6)-H PE (18:0/22:6) (18:0) (22:6) C45 H77 O8 N1 P1 790.5392305 10.46690512 0.005545028 0.775106649 0.24935866 3.268039434 527816850.2 313416760.5 525824641 401048153.7 242421000.3 344332152 323272028.2 50598746.33 399887485.6 311755073.9 312961301.7 426792665.5 457395120.2 457396996.9 453017122.4

1338NEG PC(17:1/16:0)+HCOO PC(33:1)+HCOO PC (17:1/16:0) (17:1) (16:0) C42 H81 O10 N1 P1 790.5603605 10.04550793 0.004449177 1.079762854 0.61555907 1.758326194 751937933.5 607817877.5 733122219.4 635486895.1 721974668 739198535.5 385648411.6 872872471.2 1010354231 563393745.2 673468454.8 1017970333 910626700.9 903722632.3 910815200.4

1339NEG PC(16:0e/18:1)+HCOO PC(34:1e)+HCOO PC (16:0e/18:1) (16:0e) (18:1) C43 H85 O9 N1 P1 790.5967455 10.92181147 0.010124769 1.008842623 0.976826985 2.018480961 241366381.1 193448717.8 234092090.3 245368777.3 302077981.1 346351643.4 86855401.14 282851446.8 317856842.5 142829129.2 159271470.5 586859717.6 329401937.6 323684223 329453107.6

133POS DG(16:0/18:1)+NH4 DG(34:1)+NH4 DG (16:0/18:1) (16:0) (18:1) C37 H74 O5 N1 612.5561505 20.555 0.193239312 1.055158182 0.841271902 0.018108186 2047207.085 912947.6333 1920485.452 1979135.138 1608875.976 2105977.388 634011.1313 1472620.296 1656458.778 1587408.756 1969118.697 3838288.307 1792215.5 2495575.261 1834775.434

1340NEG PG(16:1/22:6)-H PG(38:7)-H PG (16:1/22:6) (16:1) (22:6) C44 H72 O10 N0 P1 791.4868615 6.937183013 0.017186098 1.119321027 0.795681007 0.037020813 1970570.476 996613.1751 1842096.413 2203938.487 3562058.496 4284100.341 1219997.904 1724738.021 1446141.326 1824254.221 2796786.735 7620495.352 3495749.376 3395311.834 3392189.562

1341NEG PE(18:0/22:5)-H PE(40:5)-H PE (18:0/22:5) (18:0) (22:5) C45 H79 O8 N1 P1 792.5548805 10.69255819 0.00072354 1.026367399 0.859081258 0.750605385 169863341.6 114077559.8 195086349.3 141079500.6 87821026.27 141634997.1 142720196.5 152493837 163580567.4 108271866.7 111073841 193823226.9 173397918.9 173180778.3 173180815.2

1342NEG PC(18:0e/16:0)+HCOO PC(34:0e)+HCOO PC (18:0e/16:0) (18:0e) (16:0) C43 H87 O9 N1 P1 792.6123955 11.55651917 0.005312789 0.913270744 0.824506844 1.116953353 43518857.63 29524458.1 40339722.86 45141112.86 59507276.73 73003446.01 13876691.75 36829640.85 50117931.6 17995804.16 20337014.76 126636552.9 59507183.13 58962777.75 58963093.57

1343NEG PG(16:0/22:6)-H PG(38:6)-H PG (16:0/22:6) (16:0) (22:6) C44 H74 O10 N0 P1 793.5025115 7.868411552 0.018505375 1.181139753 0.682086254 0.115043669 4101992.4 1583051.602 4465183.253 3307701.461 4856148.424 7242000.705 3200092.217 3120620.423 3839431.812 3394650.942 3299846.522 13330657.57 6937679.14 6939346.4 6718473.487

1344NEG PG(18:2/20:4)-H PG(38:6)-H PG (18:2/20:4) (18:2) (20:4) C44 H74 O10 N0 P1 793.5025115 8.226853803 0.003676041 1.407233256 0.28054515 0.290876787 2483954.778 2412343.485 3251147.897 3619522.649 3193677.667 2675420.097 841884.9316 6304396.729 4203815.459 2986208.108 2737965.601 7743788.556 4928718.749 4917517.197 4893377.286

1345NEG PE(18:0/22:4)-H PE(40:4)-H PE (18:0/22:4) (18:0) (22:4) C45 H81 O8 N1 P1 794.5705305 11.17083479 0.006404429 1.076985545 0.581825515 0.332389001 135106240.6 118014819.2 150118174.7 119160330 114974051.1 127232153.5 79283203.36 186180046.3 157318986.4 105413908.9 129718482.3 165554733.9 173663461 171477229.9 172859990.5

1346NEG PG(18:1/20:4)-H PG(38:5)-H PG (18:1/20:4) (18:1) (20:4) C44 H76 O10 N0 P1 795.5181615 8.910120203 0.00330295 1.182776135 0.548437182 0.334413474 8231911.893 7740503.546 8924883.954 10191301.88 7481724.475 8096533.882 2042660.238 16421642.32 13271719.53 5749931.888 6525398.82 15916199.59 12371532.22 12312800.99 12391275.76

1347NEG PG(18:1/20:4)-H PG(38:5)-H PG (18:1/20:4) (18:1) (20:4) C44 H76 O10 N0 P1 795.5181615 8.234636947 0.02384524 1.577417675 0.386059209 0.337752788 3270041.133 1178003.594 4977165.279 4153781.513 7101111 8219200.798 1993865.45 3957099.249 2443120.779 8757291.038 7533217.157 20901678.18 8467767.663 8467024.933 8122432.676

1348NEG PS(37:4)-H PS(37:4)-H PS (37:4) (37:4) C43 H75 O10 N1 P1 796.5134105 10.34527492 0.017040963 1.027257523 0.790352868 0.244273218 5257509.685 6455686.274 6468072.562 7472259.341 5975846.148 5540320.087 7627407.223 6605590.137 4859237.382 7694768.61 6637238.248 4758606.276 5604029.734 5603968.708 5771049.353

1349NEG PC(14:0/20:5)+HCOO PC(34:5)+HCOO PC (14:0/20:5) (14:0) (20:5) C43 H75 O10 N1 P1 796.5134105 7.972994913 0.003316899 1.114836051 0.667502975 0.26167404 9785873.795 5388110.929 9277664.78 5487990.418 6320087.158 8240145.395 3287475.065 7674635.149 10991281.27 5616260.001 6686500.055 15353910.55 11055961.58 10992485.72 10992890.22

134POS DG(16:0/18:1)+NH4 DG(34:1)+NH4 DG (16:0/18:1) (16:0) (18:1) C37 H74 O5 N1 612.5561505 11.85623429 0.00613377 0.989777269 0.968328579 0.499234699 444494805.7 560612565 396664317.3 575715741.1 550811522.2 395997812.2 115016784.9 921590682.2 635596586 337704862.3 303926319.4 580567230.9 695420024.6 702925283.6 702794723

1350NEG PE(18:0/22:3)-H PE(40:3)-H PE (18:0/22:3) (18:0) (22:3) C45 H83 O8 N1 P1 796.5861805 11.62881725 0.077547631 1.233844018 0.51055675 0.5959254 30249070.21 28899584.15 53895264.28 30691813.27 60261811.8 25940285.06 15133209.96 93315234.1 69196606.05 32122477.81 34626302.55 39313583.97 67090546.89 67160670.02 58496110.66

1351NEG PG(18:0/20:4)-H PG(38:4)-H PG (18:0/20:4) (18:0) (20:4) C44 H78 O10 N0 P1 797.5338115 9.715423593 0.023317634 1.033217408 0.90426049 0.320513837 19078608.98 22159650.27 20449642.99 29607103.93 15740413.89 13524494.48 7502658.987 36067424.75 34876645.89 13240300.46 13536966.36 19340605.95 25124840.37 26157042.29 25131849.08

1352NEG PG(18:1/20:3)-H PG(38:4)-H PG (18:1/20:3) (18:1) (20:3) C44 H78 O10 N0 P1 797.5338115 8.589 0.022538396 1.146767677 0.806503936 0.183177691 2930036.585 1292415.088 3912683.884 3713234.543 3735929.491 6393058.241 939238.6594 2573385.87 2969334.154 2096411.232 2382272.568 14242281.1 5391705.346 5393123.509 5605693.361

1353NEG PC(14:0/20:4)+HCOO PC(34:4)+HCOO PC (14:0/20:4) (14:0) (20:4) C43 H77 O10 N1 P1 798.5290605 8.691259473 0.004399934 0.912454415 0.742149364 0.561872677 38306217.51 25358404.11 68084587.73 21052647.8 26975375.12 28841008.16 14378332.8 31223492.03 42016458.64 23113827.59 27120876.5 52501647.03 39674459.49 39372685.28 39374588.54

1354NEG PE(18:1/22:1)-H PE(40:2)-H PE (18:1/22:1) (18:1) (22:1) C45 H85 O8 N1 P1 798.6018305 11.97378597 0.003742484 1.406270713 0.160093317 1.660526697 39145854.76 39362456.95 44411735.94 60803484.17 70041154.72 40323373.26 21538300.31 101006003.8 54533903.71 76763503.97 95907707.24 63818006.52 78785909.08 78278191.84 78276617.23

1355NEG PC(16:1/18:2)+HCOO PC(34:3)+HCOO PC (16:1/18:2) (16:1) (18:2) C43 H79 O10 N1 P1 800.5447105 8.984850002 0.010050269 1.051807168 0.81735663 0.715083571 132255676.1 98340619.82 122658330.4 111185006.7 148009269.6 159548825.9 44869155.09 140416306.2 159464318.9 104604000.1 121562640.2 241076324 171372971.9 168415518.8 168432567.7

1356NEG PS(19:1/18:1)-H PS(37:2)-H PS (19:1/18:1) (19:1) (18:1) C43 H79 O10 N1 P1 800.5447105 9.832541517 0.044218099 2.252216066 0.060255536 1.821439667 6967592.707 15306530.34 9717381.918 16055491.24 30613801.84 20296877.69 7212890.955 43391532.47 27880164.41 48557053.66 71560532.88 24271892.79 37067524.04 40092417.08 40024779.12

1357NEG PC(16:0/18:3)+HCOO PC(34:3)+HCOO PC (16:0/18:3) (16:0) (18:3) C43 H79 O10 N1 P1 800.5447105 9.403782978 0.008034745 0.992376989 0.973043381 1.129029663 134435461.4 78120812.73 126685665.6 76773083.76 101718340.5 115033881.5 51281372.02 108444669.7 111032547.8 78424844.29 79451519.2 199308700.6 134399458.8 134419258.5 132547554.8

1358NEG PE(20:1p/22:6)-H PE(42:7p)-H PE (20:1p/22:6) (20:1p) (22:6) C47 H79 O7 N1 P1 800.5599655 10.81474328 0.034625189 1.028979657 0.837919354 0.236623323 13440395.57 11047199.9 13097132.2 12089765.05 12837626.03 12546277.1 6748511.838 18128675.79 15166072.94 10599904.73 11070759.98 15519637.11 12785444.86 11998746.95 12109618.14

1359NEG PE(18:1/22:0)-H PE(40:1)-H PE (18:1/22:0) (18:1) (22:0) C45 H87 O8 N1 P1 800.6174805 12.50435027 0.003143561 1.431782191 0.080075755 1.581171009 36920145.07 32440369.62 38016442.26 52431542.82 51198229.31 36903024.16 22596640.4 82773188.88 50583112.07 67861157.4 70147566.67 60991104.18 67642841.18 67275827.17 67275914.83

135POS DG(16:0/18:1)+NH4 DG(34:1)+NH4 DG (16:0/18:1) (16:0) (18:1) C37 H74 O5 N1 612.5561505 15.82 0.034920699 0.878286828 0.580022782 0.054563531 2684853.859 1949003.906 2494538.289 2534664.596 1816216.194 5774338.758 2066573.193 2747240.584 2664997.319 2822842.811 2715416.755 2136552.661 2500446.215 2650440.558 2492534.413

1360NEG PA(44:7)-H PA(44:7)-H PA (44:7) (44:7) C47 H78 O8 N0 P1 801.5439815 8.992878927 0.006260049 1.073350773 0.713466035 0.434946093 70369125.59 55141802.5 63008087.27 65618016.29 78256227.15 81133737.8 30309690.27 83671927.68 82730918.44 56656204.19 66828133.58 123662647.4 88208779.74 87257116.6 87261337.81

1361NEG MGDG(16:0/18:1)+HCOO MGDG(34:1)+HCOO MGDG (16:0/18:1) (16:0) (18:1) C44 H81 O12 801.5733535 10.99198035 0.002372446 0.732078787 0.401681155 1.597236304 61869605.22 32536961.3 55176247.89 39280982.92 18470763.1 57873094.98 23177111.41 30882419.28 45815394.5 4861281.996 7867106.748 81549584.73 46386326.52 46196209.84 46196263.67

1362NEG PC(16:1/18:1)+HCOO PC(34:2)+HCOO PC (16:1/18:1) (16:1) (18:1) C43 H81 O10 N1 P1 802.5603605 9.702063103 0.119494231 1.01272539 0.954425838 3.675178448 1119717880 1952444332 1183772620 2057631322 2471615788 1765954289 1077158171 2454735588 2868404196 1069711439 1218346684 1997047478 1865070212 2274769812 1857122690

1363NEG PS(19:0/18:1)-H PS(37:1)-H PS (19:0/18:1) (19:0) (18:1) C43 H81 O10 N1 P1 802.5603605 10.567 0.028851827 1.933940921 0.077088041 2.15017017 22936226.11 16194390.05 32596431.93 38280100.29 28120376.75 46250095.6 24508879.77 71018054.58 31628985.53 110423750.4 82487220.6 36508534.73 46231145.56 46261073.55 43973071.72

1365NEG PE(20:0p/22:6)-H PE(42:6p)-H PE (20:0p/22:6) (20:0p) (22:6) C47 H81 O7 N1 P1 802.5756155 11.4810807 0.001824418 1.155653496 0.444099893 0.571252699 51587053.11 56512435.45 61170302.75 57948097.18 55697206.63 58987111.75 22248805.05 89193967.89 82535755.22 52223931.91 57050988.22 91867032.51 85220449.9 85032139.09 84912652.04

1366NEG PA(44:6)-H PA(44:6)-H PA (44:6) (44:6) C47 H80 O8 N0 P1 803.5596315 9.69077451 0.018381969 1.033385156 0.862158377 0.150512018 3071770.684 2337512.6 2728836.612 2282632.506 2401711.237 2897547.115 1246597.932 2873520.041 3035813.881 2063237.432 2347585.008 4678071.469 2767808.022 2857345.34 2858330.247

1367NEG MGDG(38:5)-H MGDG(38:5)-H MGDG (38:5) (38:5) C47 H79 O10 803.5678735 10.973 0.042461286 0.498954706 0.290785848 0.734546103 8773069.658 11123761.16 84343650.03 96628409.6 12408054.72 14404145.97 12533426.37 20319298.77 10350811.69 29212888.42 28233691.56 12952434.97 13504647.04 13548255.33 12556168.14

1368NEG MGDG(16:0/18:0)+HCOO MGDG(34:0)+HCOO MGDG (16:0/18:0) (16:0) (18:0) C44 H83 O12 803.5890035 11.62 0.009444694 0.839301466 0.589235257 1.302006076 45960113.63 28454156.37 47624760.49 35403673.54 21581917.18 45591732.06 18695959.36 43993219.95 48293903.55 6804199.17 6484912.22 64248640.24 45350241.24 45350719.96 44612629.71

1369NEG SM(d20:0/18:1)+HCOO SM(d38:1)+HCOO SM (d20:0/18:1) (d20:0) (18:1) C44 H88 O8 N2 P1 803.6283795 11.1599779 0.009627611 1.010049824 0.936402154 0.448180271 42809530.39 28554662.45 39000520.34 26366501.18 37208709.8 35575206.77 22944142.26 39857627.19 38139066.08 29104008.29 34726446.56 46849430.83 43887459.22 43160052.83 43167321.11

136POS DG(18:0/16:0)+NH4 DG(34:0)+NH4 DG (18:0/16:0) (18:0) (16:0) C37 H76 O5 N1 614.5718005 12.42432181 0.017745857 0.97466049 0.932522534 0.385445007 51622557.57 23152124.04 45462703.43 30197734.03 18019789.94 36402129.76 11922438.18 50366312.63 52618626.28 15637020.29 15761884.9 53359779.56 52308200.09 53933895.23 52310664.74

1370NEG PS(38:7)-H PS(38:7)-H PS (38:7) (38:7) C44 H71 O10 N1 P1 804.4821105 9.067465245 0.016391986 1.107434976 0.474912343 0.341122109 6429641.571 5364465.146 6798014.684 7278737.648 8009987.593 7577918.657 3913638.881 8058512.34 7717909.259 9273299.814 10403937.4 6545589.06 8837052.547 8588751.466 8837558.194

1371NEG PC(16:0/18:1)+HCOO PC(34:1)+HCOO PC (16:0/18:1) (16:0) (18:1) C43 H83 O10 N1 P1 804.5760105 10.805 0.110910114 1.487651232 0.171372937 6.453480012 125180471.5 138367792 1337123774 1022157184 1056577058 1382685519 527391469.1 1209393701 1486817438 1392335379 1339414676 1575274437 1138542761 937725477.2 1149594152

1372NEG PC(16:0/18:1)+HCOO PC(34:1)+HCOO PC (16:0/18:1) (16:0) (18:1) C43 H83 O10 N1 P1 804.5760105 10.42812217 0.003888985 1.051177492 0.637257603 4.260772883 5570477961 4679516323 5344564130 5586201820 5578105560 5571651829 3462192592 6547816668 6706819271 4907410808 5412439550 6948433548 6269073072 6227003836 6269201910

1373NEG PC(16:0/18:1)+HCOO PC(34:1)+HCOO PC (16:0/18:1) (16:0) (18:1) C43 H83 O10 N1 P1 804.5760105 12.02095958 0.058885747 1.106773467 0.686817316 0.206455004 17887931.11 15355527.55 17373448.5 26107365.65 11038299.27 18083805.09 9739581.092 22243460.11 38138626.3 16583897.42 16091555.82 14350841.07 20578460.29 22639415.05 20388051.81

1374NEG PE(20:0p/22:5)-H PE(42:5p)-H PE (20:0p/22:5) (20:0p) (22:5) C47 H83 O7 N1 P1 804.5912655 11.65047056 0.011094129 1.195494152 0.462447676 1.018135724 95222611.08 69181754.8 63885442.28 83091649.63 56153143.41 75965879.81 44678967.38 113731398.9 118230297.5 51617736.38 52826511.54 149117319.8 83357690.04 81540788.46 82274190.82

1375NEG PI(18:1/14:1)-H PI(32:2)-H PI (18:1/14:1) (18:1) (14:1) C41 H74 O13 N0 P1 805.4872565 7.628390337 0.009202494 0.579088129 0.237283097 0.672622003 7837543.929 3424273.003 11919503.61 9886193.849 2902049.382 14722776.45 3571012.128 3988746.55 3648646.909 1531249.439 1544893.591 15070783.86 7719470.72 7719208.188 7843036.447

1376NEG PS(16:0/22:6)-H PS(38:6)-H PS (16:0/22:6) (16:0) (22:6) C44 H73 O10 N1 P1 806.4977605 8.512213375 0.011876914 1.080193088 0.661992059 0.160321861 17516324.43 12233313.91 29262142.75 17215601.47 12438024.9 29195650.14 18891148.26 21987871.35 16034032.71 20491054.73 23446326.22 26462266.45 22888981.34 22420806.07 22887911.23

1377NEG PC(18:0/16:0)+HCOO PC(34:0)+HCOO PC (18:0/16:0) (18:0) (16:0) C43 H85 O10 N1 P1 806.5916605 11.09966015 0.002434173 0.922444447 0.581938396 2.50868231 372989262.8 400231925.1 360219077.3 418326340.6 407027167.5 348808981.9 142066473.5 446109353.1 476841621 302853739.6 325317909.5 435446250.7 437894066.2 439750474.5 439740444.7

1378NEG PE(42:4p)-H PE(42:4p)-H PE (42:4p) (42:4p) C47 H85 O7 N1 P1 806.6069155 11.96276589 0.011001092 1.046963611 0.853690692 0.511056548 17347463.82 11408504.74 19381799.36 15266118.89 16303054.33 20428623.77 8240369.374 20339534.73 23929735.54 11285473.93 8883702.893 32159476.16 19061889.59 19123592.12 18735181.07

1379NEG PI(16:0/16:1)-H PI(32:1)-H PI (16:0/16:1) (16:0) (16:1) C41 H76 O13 N0 P1 807.5029065 8.547284877 0.002708629 0.801047525 0.522743153 1.202257572 68098026.75 30377033.19 115614306.2 60068012.3 28692288.44 117555965 49362433.04 53970926.83 44117314.84 32160948.46 35030801.58 122122466.1 69335792.48 69334750.7 69010495.51

137POS Cer(d18:2/22:0)+H Cer(d40:2)+H Cer (d18:2/22:0) (d18:2) (22:0) C40 H78 O3 N1 620.5976205 11.97202299 0.023436372 1.025972385 0.87854101 0.027605005 16815822.64 15356961.43 15911359.59 14324477.44 23781770.04 17657474.9 9279831.303 25012886.44 21345620.66 11994093.77 16873743.17 22038867.46 26056333.5 26066144.16 25017492.8

1380NEG PS(20:3/18:2)-H PS(38:5)-H PS (20:3/18:2) (20:3) (18:2) C44 H75 O10 N1 P1 808.5134105 10.13368477 0.003110375 1.044854167 0.45206295 0.454066356 22392770.7 25132769.41 29756794.86 29375918.09 29749820.3 29375300.53 26644042.41 29654900.98 25529455.6 30097062.3 31629805.84 29664181.93 29918574.09 29757400.14 29918006.05

1381NEG PS(18:0/20:5)-H PS(38:5)-H PS (18:0/20:5) (18:0) (20:5) C44 H75 O10 N1 P1 808.5134105 9.108044808 0.016530504 1.239475911 0.318993963 0.319509443 10942270.26 8432683.73 17817973.23 10654895.16 7251938.259 23316630.18 12277466.29 19910916.2 17582982.29 12033115.53 14451390.74 20939356.4 17746889.56 17181218.63 17569131.62

1382NEG PC(16:0p/20:5)+HCOO PC(36:5p)+HCOO PC (16:0p/20:5) (16:0p) (20:5) C45 H79 O9 N1 P1 808.5497955 9.425469972 0.006476969 1.442016938 0.179013287 0.685914766 8007279.888 10536148.77 8530247.332 7936051.674 21863686.48 10103985.14 3477457.376 17497393.8 16922455.46 19590391.62 21608181.65 17486664.35 18572860 18465204.27 18705472.08

1383NEG PI(16:0/16:0)-H PI(32:0)-H PI (16:0/16:0) (16:0) (16:0) C41 H78 O13 N0 P1 809.5185565 9.371783603 0.002782604 0.859500316 0.69874492 0.641620191 36881166.63 8520010.463 59093434.74 17085873.77 7022243.037 46158885.8 27535121.08 22575662.39 24224137.94 10728175.83 16317404.48 48827161.13 28387786.16 28387413.93 28251002.68

1384NEG PE(20:4/22:6)-H PE(42:10)-H PE (20:4/22:6) (20:4) (22:6) C47 H73 O8 N1 P1 810.5079305 8.769346433 0.007181933 1.002785386 0.989971467 0.330812182 6641504.436 6592405.481 8499951.203 5663089.32 4675979.741 6562240.086 3052794.755 8110257.033 8539697.067 3741991.133 4393223.257 10904820.9 8847076.878 8849059.029 8738470.537

1385NEG PS(18:0/20:4)-H PS(38:4)-H PS (18:0/20:4) (18:0) (20:4) C44 H77 O10 N1 P1 810.5290605 9.685365885 0.010663215 1.141474772 0.476424775 0.319358015 36632857.82 44376089.94 61744636.39 53846348.69 34801689.76 70725566 43995426 90270383.22 64516030.52 41311674.99 44048544.47 60728504.49 55183330.52 55180404.89 54168940.22

1386NEG PS(18:0/20:4)-H PS(38:4)-H PS (18:0/20:4) (18:0) (20:4) C44 H77 O10 N1 P1 810.5290605 10.74512542 0.002486913 0.973501812 0.764382463 1.212374573 109330770.7 112412767 122796967.2 166655679.8 125305320.1 119996778.9 112913246 120534444.9 113643780.1 150269315.4 133772199.8 105319464.1 118226272.7 118734765.1 118223308.9

1387NEG PC(16:0p/20:4)+HCOO PC(36:4p)+HCOO PC (16:0p/20:4) (16:0p) (20:4) C45 H81 O9 N1 P1 810.5654455 10.01619404 0.024191348 1.484673723 0.143231163 1.469317735 30033116.34 39325612.46 24627031.56 31285233.85 73499349.69 38827459.06 15785677.56 65760971.1 60091754.18 73405995.96 85108076.23 52602739.72 61776212.93 59226327.48 61782299.51

1388NEG PS(18:0/20:3)-H PS(38:3)-H PS (18:0/20:3) (18:0) (20:3) C44 H79 O10 N1 P1 812.5447105 11.36844664 0.011528409 1.051328457 0.302394371 0.888501878 172201416.5 162860553.2 188257911.6 179433945.9 159059875.9 151965593.2 153192682.8 186993069.5 173704776.3 182079927.6 194016121.9 175828445.4 181118165.3 181150788.1 184775456.5

1389NEG PS(18:0/20:3)-H PS(38:3)-H PS (18:0/20:3) (18:0) (20:3) C44 H79 O10 N1 P1 812.5447105 9.8860696 0.005663664 0.781705534 0.087721185 2.531280965 179211059 193050945.1 155078310.2 179650518.2 140484059.1 183394022 190074733.8 135144573.2 156693308.8 88097432.14 75860888.77 159964998.2 153050676 151358793.2 151922398.1

138POS LPI(20:4)+H LPI(20:4)+H LPI (20:4) (20:4) C29 H50 O12 N0 P1 621.3034445 1.786530322 0.001280556 1.497521387 0.191892717 0.105472177 482980.7129 245182.5521 1376145.6 1088636.758 2141123.901 1265977.105 1530849.261 1132211.708 755049.3781 2426308.886 1614170.541 2425121.206 1457553.515 1457572.906 1454332.785

1390NEG PC(16:0e/20:4)+HCOO PC(36:4e)+HCOO PC (16:0e/20:4) (16:0e) (20:4) C45 H83 O9 N1 P1 812.5810955 10.17555558 0.002964881 1.128232189 0.649215817 0.512222816 35407484.67 23376769.91 33851209.81 25938976.58 37198097.36 40226969.38 11993826.21 39893051.45 46919013.47 23907472.58 27132497.24 71287092.6 44007356.16 44007152.73 43781649.15

1391NEG PE(18:1/23:1)-H PE(41:2)-H PE (18:1/23:1) (18:1) (23:1) C46 H87 O8 N1 P1 812.6174805 12.20487599 0.01458865 1.450040071 0.125840691 0.594711669 4757614.457 4884103.735 5373495.046 6609823.235 7908202.027 4534908.464 2448640.647 12285439.24 6574117.258 9271298.599 10995194.1 7825488.385 8771305.124 8548978.184 8557978.043

1392NEG PE(42:1p)-H PE(42:1p)-H PE (42:1p) (42:1p) C47 H91 O7 N1 P1 812.6538655 13.27973642 0.00596792 1.294482357 0.216517978 0.413070332 4375943.056 4916203.311 5317823.16 7016297.118 8345748.522 6492500.102 2583682.092 11623475.72 7455499.463 8015860.471 8326611.581 9197542.346 9557896.677 9553584.954 9457375.368

1393NEG PS(40:8p)-H PS(40:8p)-H PS (40:8p) (40:8p) C46 H73 O9 N1 P1 814.5028455 10.017 0.006551324 0.946576797 0.398487651 0.779383076 34370427.05 36956847.09 35991156.22 42069692.68 40589172.87 40021463.09 28334968.41 35016900.1 35614129.04 38974321.99 42912868.05 36858301.12 37046678.63 36627689.53 37046281.6

1394NEG PS(20:1/18:1)-H PS(38:2)-H PS (20:1/18:1) (20:1) (18:1) C44 H81 O10 N1 P1 814.5603605 10.59694244 0.010959542 1.162344298 0.592532455 0.411386005 12300878.43 15181565.39 22720717.94 29762770.28 27056549.32 56197655.55 17401392.05 35367677.54 19156372.27 33721111.1 35412670.76 48658771.67 24494049.16 24966162.32 24502391.09

1395NEG PC(17:1/18:2)+HCOO PC(35:3)+HCOO PC (17:1/18:2) (17:1) (18:2) C44 H81 O10 N1 P1 814.5603605 9.43645749 0.01641002 1.152031968 0.51658848 0.442539188 33077018.86 22991762.24 29464353.28 27629404.48 35254087.31 28828338.27 14047301.13 50101858.36 42948878.51 22524037.91 23950590.71 50619198.57 43954789.71 45042911.93 45354573.42

1396NEG PC(18:1p/18:1)+HCOO PC(36:2p)+HCOO PC (18:1p/18:1) (18:1p) (18:1) C45 H85 O9 N1 P1 814.5967455 10.83579289 0.004111465 1.196602037 0.430458125 0.373324771 33525736.69 48257999.12 32062806.34 43475923.48 73757969.72 47912493.85 14483061.87 73127029.76 66690219.78 51429269.48 51830680.28 76283246.35 59680493.48 59255175.17 59677813.6

1397NEG PE(20:1/22:6)-H PE(42:7)-H PE (20:1/22:6) (20:1) (22:6) C47 H79 O8 N1 P1 816.5548805 10.437 0.009952004 1.065456978 0.669353472 0.126540522 9496025.322 7389727.341 5991727.104 7355865.561 4175324.414 3623630.72 7927602.854 7049866.28 5879130.921 6377695.949 6715935.073 6571548.825 7169213.613 7049292.598 7175011.793

1398NEG PC(17:1/18:1)+HCOO PC(35:2)+HCOO PC (17:1/18:1) (17:1) (18:1) C44 H83 O10 N1 P1 816.5760105 10.10869448 0.007249801 1.077561677 0.639408548 0.275371996 483120213.8 441738771.6 459155197.8 549585978.2 619460099.6 521006788.9 220263535.1 642056299.8 695122060.1 467052835.8 547637906.8 740364208.3 654197413.3 654283397.3 646059673.4

1399NEG PS(20:0/18:1)-H PS(38:1)-H PS (20:0/18:1) (20:0) (18:1) C44 H83 O10 N1 P1 816.5760105 11.1739761 0.221279685 1.539975417 0.006648012 0.922873098 15252681.02 16070532.15 23825865.32 29559227.52 21691628.78 31164221.51 38874953.17 33844323.61 34095946.46 44099796.21 34624620.07 26305779.38 52116794.86 34395090.68 52086994.1

139POS PC(24:0)+H PC(24:0)+H PC (24:0) (24:0) C32 H65 O8 N1 P1 622.4442335 6.017142253 0.035346806 1.004229422 0.9923552 0.095173306 2000621.15 1055377.055 1652947.111 1235029.189 608919.3076 1546897.407 218336.5477 2258503.66 1725331.492 491252.5362 36192.79798 3404431.617 3281741.162 3486644.748 3495477.465

13POS WE(21:1)+NH4 WE(21:1)+NH4 WE (21:1) (21:1) H44 C21 O2 N1 342.3366555 2.829885778 0.002871262 1.320915119 0.493272662 0.138654363 3518667.517 10332528.59 4067230.787 10741169.23 6569888.042 5486892.305 893025.878 13028284.1 8474067.756 6311174.629 4829162.739 20247162.16 11442906.11 11386162.36 11386210.91

1400NEG PC(18:0p/18:1)+HCOO PC(36:1p)+HCOO PC (18:0p/18:1) (18:0p) (18:1) C45 H87 O9 N1 P1 816.6123955 10.9504209 0.020893785 1.069382939 0.787280752 0.875556952 56208032.17 50193128.63 54954350.12 65114081.51 73140816 62691638.25 26512556.81 76131525.02 106099819.6 32783757.63 40904871.36 105007097.2 75611930.63 72915638.14 75627254.39

1401NEG PC(18:0p/18:1)+HCOO PC(36:1p)+HCOO PC (18:0p/18:1) (18:0p) (18:1) C45 H87 O9 N1 P1 816.6123955 11.48769013 0.010671539 1.206568042 0.42572968 0.424005097 40775964.7 49885048.89 38758108.36 49885550.69 91143314.73 57618633.22 16712406.48 71723294.86 86293106.67 61326285.1 65084878.22 94694728.65 79609815.07 78156866.12 78155628.31

1402NEG PG(18:2/22:6)-H PG(40:8)-H PG (18:2/22:6) (18:2) (22:6) C46 H74 O10 N0 P1 817.5025115 7.203320145 0.007568905 1.293428712 0.399044139 0.338440331 3040413.117 2037570.669 2778455.947 3406266.261 8156837.495 4971762.283 1753179.026 4259209.674 3568137.758 6422623.957 7936889.321 7608375.475 6446437.943 6362644.046 6447141.643

1403NEG PC(17:0/18:1)+HCOO PC(35:1)+HCOO PC (17:0/18:1) (17:0) (18:1) C44 H85 O10 N1 P1 818.5916605 10.80748141 0.008128661 1.04579908 0.773393809 1.812268016 583795106.8 553513309.2 550329184.5 551414984.2 550252973.9 622470283.8 305035052.8 786292650.3 851656772.8 475359211.9 459829067.5 689859280.4 698293996.5 688514003.8 698305227.3

1404NEG PC(18:0e/18:1)+HCOO PC(36:1e)+HCOO PC (18:0e/18:1) (18:0e) (18:1) C45 H89 O9 N1 P1 818.6280455 11.59971679 0.003749279 1.011418073 0.969483689 1.158075758 60310080.75 52374421.33 55216499.33 69210753.84 69767402.21 71880775.19 19343518.06 80925843.47 97551506.51 27575742.99 30818897.69 126869132.6 83582472.9 83041409.47 83042658.38

1405NEG PG(18:1/22:6)-H PG(40:7)-H PG (18:1/22:6) (18:1) (22:6) C46 H76 O10 N0 P1 819.5181615 7.988779872 0.004968501 1.336452703 0.384157114 0.863146675 19501581.95 14747187.88 20718733.93 33791278.6 69270184.45 43363953.77 14292816 37945307.32 30424746.35 48605214.59 59817598.89 78066429.89 55279117.45 54846207.49 54774227.91

1406NEG PE(22:1/20:4)-H PE(42:5)-H PE (22:1/20:4) (22:1) (20:4) C47 H83 O8 N1 P1 820.5861805 11.21226213 0.014018733 0.961333739 0.851835302 0.174034431 20107623.52 19330140.99 27370005.23 34015751.98 14853398.78 15206145.42 11824639.07 29480947.83 25981163.83 27993945.4 13768615.74 16772995.21 25619181.57 25005646.81 25626360.87

1407NEG PC(20:0e/16:0)+HCOO PC(36:0e)+HCOO PC (20:0e/16:0) (20:0e) (16:0) C45 H91 O9 N1 P1 820.6436955 12.15502955 0.007795537 1.012509057 0.96569084 0.42724298 9804859.063 8183371.604 9344224.919 8492655.188 13462104.95 13406750.83 4820530.522 12380872.83 12730253.22 5122036.197 5909575.967 22514940.23 13020177.37 12843538.07 13016834.55

1408NEG PI(17:1/16:0)-H PI(33:1)-H PI (17:1/16:0) (17:1) (16:0) C42 H78 O13 N0 P1 821.5185565 9.021999188 0.007271238 0.907626882 0.713849435 0.328603478 16789702.51 8440010.649 29552979.77 15058659.64 9300771.484 26110593.72 14650421.27 17969710.48 10731251 12206035.59 12963825.81 27008951.89 18454052.93 18724254.61 18597480

1409NEG PG(18:1/22:5)-H PG(40:6)-H PG (18:1/22:5) (18:1) (22:5) C46 H78 O10 N0 P1 821.5338115 8.712730653 0.008202818 0.956220063 0.910435234 0.271512 5122890.158 3738729.825 4309782.963 6694456.807 17996950.74 8755509.005 2862460.905 9767405.545 7385339.34 3829672.908 4784689.113 15947804.59 9957954.351 9958154.24 10100208.45

140POS PC(25:0e)+H PC(25:0e)+H PC (25:0e) (25:0e) C33 H69 O7 N1 P1 622.4806185 7.263857895 0.017524511 1.332643125 0.185780868 0.4837948 15572968.99 23769892.07 24871140.35 32543961.42 35027055.31 33351590.4 11728342.46 47389409.89 50072159.41 32483056.71 36097861.66 42297335.89 46925195.75 48380211.19 46956993.79

1410NEG PC(14:0/22:6)+HCOO PC(36:6)+HCOO PC (14:0/22:6) (14:0) (22:6) C45 H77 O10 N1 P1 822.5290605 8.395 0.087858062 0.956551809 0.903449299 1.292449219 70660103.52 42628827.21 39227082.04 38356509.63 56084102.23 41994033.06 13655711.6 56680939.28 77510358.24 10010025.46 15277444.27 103261795.5 76993340.72 77052295.9 65867850.88

1411NEG PE(22:0/20:4)-H PE(42:4)-H PE (22:0/20:4) (22:0) (20:4) C47 H85 O8 N1 P1 822.6018305 11.95643845 0.006584962 1.221349399 0.195687826 0.486079908 10909883.56 7635828.623 12109327.87 10784110.07 10389726.54 12055061.43 6517518.66 17612499.92 12317092.82 12274237.91 13314571.66 15988688.43 15273383.47 15448214.38 15273315.66

1412NEG Cer(d50:1+pO)+HCOO Cer(d50:1+pO)+HCOO Cer (d50:1+pO) (d50:1+pO) C51 H100 O6 N1 822.7556125 14.6129584 0.003888328 1.150173885 0.482215175 0.063163522 855051.8123 619321.2101 944475.0784 879558.1886 940873.8031 809226.3032 421368.36 1257410.796 1060269.468 731847.1542 780650.6853 1555113.749 1217313.05 1224995.76 1226007.123

1413NEG PC(16:0/20:5)+HCOO PC(36:5)+HCOO PC (16:0/20:5) (16:0) (20:5) C45 H79 O10 N1 P1 824.5447105 8.80329419 0.004045519 1.467698077 0.101589113 1.85767669 147726138.4 91532268.45 141180170.7 74907828.31 100235275.1 114570192.3 96306224.48 134136506.9 270755352.5 110679745 200895651 170807136.1 153993530.5 152917154.4 153993830.6

1414NEG PE(24:1/18:2)-H PE(42:3)-H PE (24:1/18:2) (24:1) (18:2) C47 H87 O8 N1 P1 824.6174805 11.99169934 0.069682331 1.07748361 0.643347262 0.410734921 12704033.7 12975146.31 15091433.39 16329774.56 18111341.29 11732356.46 7105505.526 16911049.22 11422689.05 18621165.11 19591751.27 20028667.16 18624427.95 21069596.84 21068333.09

1415NEG PC(16:0/20:4)+HCOO PC(36:4)+HCOO PC (16:0/20:4) (16:0) (20:4) C45 H81 O10 N1 P1 826.5603605 9.639965723 0.003828143 1.16225292 0.233098634 0.575642781 286322469.5 193786581.3 258144446.5 155160956.4 205979409.5 200510479 170723451.7 262277318.1 310076833.3 226475970.8 257843334.5 283420708.6 292853831.2 292848161.5 290913525.2

1416NEG PC(18:1/18:3)+HCOO PC(36:4)+HCOO PC (18:1/18:3) (18:1) (18:3) C45 H81 O10 N1 P1 826.5603605 9.24472408 0.015655692 1.113388777 0.680801656 0.999148535 104085007 73228878.26 93994646.69 110077885.7 113093040.5 92421966.89 36685741.34 149311273.6 185406152.8 75014988.64 50668752.11 156362551.4 111293656.6 108335240 108323504.1

1417NEG PE(18:1/24:1)-H PE(42:2)-H PE (18:1/24:1) (18:1) (24:1) C47 H89 O8 N1 P1 826.6331305 12.47488842 0.009908235 1.423268765 0.160285042 1.418935988 27758312.29 31997508.93 29442465.97 48844611.27 59005992.88 32062061.35 16417213.44 80098856.18 40095760.22 65683718.04 66948584.78 56842330.11 63107644.16 62036207.99 62037531.11

1418NEG PS(40:9)-H PS(40:9)-H PS (40:9) (40:9) C46 H71 O10 N1 P1 828.4821105 8.817 0.008606956 0.968699508 0.753248377 0.198101056 6326423.987 5548112.934 7026561.224 5616659.59 4935553.809 4803986.466 3810772.454 4942569.12 5833396.873 6038632.087 6924497.218 5635159.96 6627199.845 6625283.265 6527962.02

1419NEG PC(18:1/18:2)+HCOO PC(36:3)+HCOO PC (18:1/18:2) (18:1) (18:2) C45 H83 O10 N1 P1 828.5760105 9.861406418 0.040117157 0.880932131 0.416413031 3.579609064 639638421.2 461689677.8 727544477.4 759891076.6 650058839.3 575892226.6 247801326 649908400.7 720285505.6 464857120.2 519312163.6 758340251.1 810452541.2 757676127.9 755641904.2

141POS Cer(d18:1/22:0)+H Cer(d40:1)+H Cer (d18:1/22:0) (d18:1) (22:0) C40 H80 O3 N1 622.6132705 12.57261409 0.017473932 1.123254729 0.521087686 0.399205588 123666104.7 80287428.45 105285209.7 95202234.53 116967369.6 112327678.1 59940273.29 156834031.1 148802296.2 77082666.96 101449238.2 167738481.5 166994489.2 167033495.2 172120196.5

1420NEG PC(16:0/20:3)+HCOO PC(36:3)+HCOO PC (16:0/20:3) (16:0) (20:3) C45 H83 O10 N1 P1 828.5760105 10.2488632 0.002237148 1.167762522 0.417852179 0.843351262 118175733.5 78278463.48 101486083.7 97509950.75 112980932.5 110188851 53917970.91 154030413.4 150122961.7 88441052.79 96274014.47 179614855.7 138520486 137982287.2 138516154.2

1421NEG PE(18:1/24:0)-H PE(42:1)-H PE (18:1/24:0) (18:1) (24:0) C47 H91 O8 N1 P1 828.6487805 13.03808978 0.009464531 1.490821085 0.083666028 2.382747499 64688107.01 60672723.71 80716970.94 99852257.38 90437561.51 72721036.66 36682057.1 167923211.2 91519282.43 128431781.1 146647372.8 128123556.1 133258433.8 133267192.9 131090115.3

1422NEG PI(14:0/20:4)-H PI(34:4)-H PI (14:0/20:4) (14:0) (20:4) C43 H74 O13 N0 P1 829.4872565 7.558 0.010998367 1.003756223 0.989468243 0.057815009 761934.0921 387575.3457 1115412.81 591446.7343 378670.7001 1136311.427 450262.0528 740413.7115 593535.5912 559187.6658 600737.5205 1443634.336 890801.2183 873861.1872 890641.9154

1423NEG MGDG(18:0/18:1)+HCOO MGDG(36:1)+HCOO MGDG (18:0/18:1) (18:0) (18:1) C46 H85 O12 829.6046535 11.64997005 0.013345691 0.708767545 0.348649314 0.90946215 17028151.79 11003902.54 17010864.85 17460812.55 5268324.606 16563844.55 5649687.245 12474544.14 14790778.62 1053418.813 1654536.725 24151583.89 14918518.7 14586498.11 14571782.04

1424NEG SM(d22:1/18:1)+HCOO SM(d40:2)+HCOO SM (d22:1/18:1) (d22:1) (18:1) C46 H90 O8 N2 P1 829.6440295 11.11809959 0.039769548 1.067182273 0.780755823 0.221141236 48776499.52 35430430.75 43427307.19 29350355.14 83124129.4 38909262.72 25276510.35 74046580.67 62864093.79 32778564.49 44458334.51 58338963.44 72887075.72 67926923.94 68263715.01

1425NEG PS(40:8)-H PS(40:8)-H PS (40:8) (40:8) C46 H73 O10 N1 P1 830.4977605 9.343696083 0.016536201 1.173966527 0.377246454 0.689275028 15697253.78 15844984.31 16862489.49 14833754.84 19934947.95 15676523.28 12020190.8 16925174.66 13747318.87 27665282.37 29463631.62 16224938.45 18584060.89 18062528.44 18595620.59

1426NEG PS(39:1)-H PS(39:1)-H PS (39:1) (39:1) C45 H85 O10 N1 P1 830.5916605 10.49380274 0.002159202 1.105119917 0.45020795 2.19402438 2911843916 2536739130 2872156899 2876460238 3720412520 3019048403 1681774174 3845544533 3959771775 2958262487 3331114138 4045694327 3766119402 3752072708 3752066732

1427NEG PI(16:1/18:2)-H PI(34:3)-H PI (16:1/18:2) (16:1) (18:2) C43 H76 O13 N0 P1 831.5029065 7.889320982 0.090412138 0.859706745 0.674065714 0.26235772 3636632.495 1869628.86 5705210.52 3868696.242 2136816.256 5887380.34 1570772.898 4968716.08 2124144.071 1618874.506 1812326.898 7768143.734 4752338.758 4728440.331 5523322.204

1428NEG SM(d22:0/18:1)+HCOO SM(d40:1)+HCOO SM (d22:0/18:1) (d22:0) (18:1) C46 H92 O8 N2 P1 831.6596795 11.79932179 0.000863912 1.074040426 0.693274122 1.597499284 476347492.6 332203852.3 457940556.4 337707468 389061496.2 388106792.5 199509647.2 541349358.1 524817582.6 301675863.8 353947244.8 636385437.9 506998650 507365043.2 507871623.9

1429NEG PS(40:7)-H PS(40:7)-H PS (40:7) (40:7) C46 H75 O10 N1 P1 832.5134105 10.17062119 0.003063923 1.043529042 0.617876862 0.26473594 27477094.99 22548583.55 27050393.64 19993585.02 17110238.53 21239987.4 26709170.45 22688502.88 22505103.27 22220289.65 26174829.57 21016685.09 24133689.34 24117492.68 24253078.58

142POS PC(26:5p)+H PC(26:5p)+H PC (26:5p) (26:5p) C34 H59 O7 N1 P1 624.4023685 3.155363842 0.004288507 1.16846074 0.761710587 0.071131691 804444.9031 601489.8206 1052092.613 253321.9001 288436.8944 426997.1422 256538.5855 994103.4736 1750399.004 8927.030884 32864.72791 961228.8962 930791.0149 938307.5905 936977.7251

1430NEG PS(18:1/22:6)-H PS(40:7)-H PS (18:1/22:6) (18:1) (22:6) C46 H75 O10 N1 P1 832.5134105 8.590320442 0.041706859 1.008945463 0.965655723 0.071749464 11085327.68 9055092.168 24911971.36 12494796.49 11750411.74 21013451.63 10273223.76 14106654.05 13641794.15 14393886.44 16692668.28 22010698.56 14986132.36 16124149.31 16122809.46

1431NEG PC(18:0/18:1)+HCOO PC(36:1)+HCOO PC (18:0/18:1) (18:0) (18:1) C45 H87 O10 N1 P1 832.6073105 11.14819823 0.011358927 1.090518401 0.532045656 1.89949737 3082591984 2937693916 2903344813 3003251257 2903723121 2811450642 1709736906 4077171484 4270365730 2482695039 2940684072 3758333186 3888191772 3812758437 3812611607

1432NEG PI(16:1/18:1)-H PI(34:2)-H PI (16:1/18:1) (16:1) (18:1) C43 H78 O13 N0 P1 833.5185565 9.029 0.039217584 1.114013064 0.693001078 0.079893356 4094385.825 2469755.158 6173718.368 4079802.88 2522734.853 5949488.831 2780509.726 4997042.243 2889939.083 3801175.621 4399055.609 9305541.005 4626457.461 4623115.258 4946197.983

1433NEG PI(16:1/18:1)-H PI(34:2)-H PI (16:1/18:1) (16:1) (18:1) C43 H78 O13 N0 P1 833.5185565 8.635413665 0.003383979 0.866684709 0.584568566 1.656622418 165564099.1 111373011.4 271807242.2 244304148.9 150610620.2 340320423.4 112108140.9 199831014 148050196.6 143400558.1 152122877.8 357292651.5 222518163.1 224014590.2 223087255.3

1434NEG SM(d22:0/18:0)+HCOO SM(d40:0)+HCOO SM (d22:0/18:0) (d22:0) (18:0) C46 H94 O8 N2 P1 833.6753295 12.02138324 0.027268719 1.026264891 0.891192786 0.499940735 23298942.74 17053919.7 21751303.74 19059569.41 21193933.29 18579240.24 9336716.446 25287211.12 29045854.25 14388853.78 14973691.03 31080977.2 25818012.07 27050133.33 25805152.5

1435NEG PS(18:0/22:6)-H PS(40:6)-H PS (18:0/22:6) (18:0) (22:6) C46 H77 O10 N1 P1 834.5290605 9.83099194 0.004849734 1.086456945 0.749904714 0.211695858 53871117.26 24422532.26 86953065.31 47290343.98 20202917.47 77907265.6 54149655.21 84875499.39 36731669 46680722.32 40091224.97 74976082.35 57908762.49 58252783.38 57695790.86

1436NEG PS(40:6)-H PS(40:6)-H PS (40:6) (40:6) C46 H77 O10 N1 P1 834.5290605 10.71011854 0.006774521 1.013069745 0.874560193 0.438231722 48903980.88 43711200.52 54981319.95 41587250.55 35299328.62 47556250.58 56876548.2 47197687.69 44028461.77 44960222.91 39856844.05 42675051.27 45038577.41 44515015.82 44513397.95

1437NEG PS(18:0/22:6)-H PS(40:6)-H PS (18:0/22:6) (18:0) (22:6) C46 H77 O10 N1 P1 834.5290605 9.440261065 0.01217551 0.957134835 0.849291498 0.838708226 151685273.1 76788728.03 220239461.5 117430536.7 59979849.78 184853875 163161160.6 131747152.3 112596204.4 95705205.76 89265464.1 183739842.8 141263666.4 144273908.6 141283542.3

1438NEG PC(16:0p/22:6)+HCOO PC(38:6p)+HCOO PC (16:0p/22:6) (16:0p) (22:6) C47 H81 O9 N1 P1 834.5654455 9.758540192 0.003638075 1.260608619 0.317597285 0.490396488 14923112.1 22123147.19 14198081.79 17025391.16 35207423.83 17544318.81 6798640.458 29206856.65 32453968.22 25821722.52 29782398.56 28497127.91 29394256.8 29194855.97 29360173.26

1439NEG PC(20:0/16:0)+HCOO PC(36:0)+HCOO PC (20:0/16:0) (20:0) (16:0) C45 H89 O10 N1 P1 834.6229605 11.7484321 0.15523945 0.575300035 0.071898648 1.474593108 23869783.78 46694702.08 50923683.3 25952925.17 19285180.69 56421929.17 8573162.577 26094709.4 33039973.21 13689942.58 13871530.08 33107851.77 43962255.23 33938811.24 33937982.11

143POS DG(17:1/18:1)+NH4 DG(35:2)+NH4 DG (17:1/18:1) (17:1) (18:1) C38 H74 O5 N1 624.5561505 11.58112775 0.010949154 0.965709869 0.887813753 0.093589264 21286341.18 24655594.61 21505509.92 30551941.27 26110859.83 16481221.42 4958795.702 41062847.87 29478303.83 17327465.49 15182342.44 27760813.03 32960073.52 33589053.34 32959940.68

1440NEG PI(16:0/18:1)-H PI(34:1)-H PI (16:0/18:1) (16:0) (18:1) C43 H80 O13 N0 P1 835.5342065 9.458008575 0.007317657 0.897548999 0.658639521 1.971429587 421649392 203530982 638029107.1 390358817.6 238468645 640264638.7 388583676.1 399972299.8 283786535 301969975.4 264236055.9 634316208.8 448813933 447815416 442722577.2

1441NEG PS(18:0/22:5)-H PS(40:5)-H PS (18:0/22:5) (18:0) (22:5) C46 H79 O10 N1 P1 836.5447105 9.766191272 0.035889356 0.644274458 0.241554323 1.691864306 104975057.9 31919381.47 74144837.07 73813502.07 17100413.95 148440682.6 33245679.85 36780389.75 57093434.26 46879158.99 29192056.87 86986550.15 53255213.46 49670718.98 52305250.75

1442NEG PS(20:2/20:3)-H PS(40:5)-H PS (20:2/20:3) (20:2) (20:3) C46 H79 O10 N1 P1 836.5447105 10.98321849 0.013341307 1.01556357 0.850774408 0.239323568 19052031.48 16310570.42 21655389.51 17628885.2 16005755.03 18147298.38 18255077.47 24170968.89 16909436.89 16119994.15 17996628.06 17041139.94 19048072 18614413.21 18614767.84

1443NEG PC(18:1p/20:4)+HCOO PC(38:5p)+HCOO PC (18:1p/20:4) (18:1p) (20:4) C47 H83 O9 N1 P1 836.5810955 10.04635147 0.02281648 1.11991374 0.637067988 0.285492892 22740181.48 30938472.62 31344119.77 21607971.05 55983165.95 58008681.94 11529391.17 42622339.96 51170067.64 42961460.66 45264169.31 53530844.36 51503520.2 49510391.26 49530528.38

1444NEG PI(18:0/16:0)-H PI(34:0)-H PI (18:0/16:0) (18:0) (16:0) C43 H82 O13 N0 P1 837.5498565 10.18218391 0.014815967 0.873218605 0.659487875 0.753446488 76988824.16 32459558.32 131602900.3 49807579 20526032.12 90593561.75 65373790.24 65973635.25 45113190.55 39727657.39 42649805.43 92176987.58 63076587.52 64789614.52 64647939.09

1445NEG PS(18:0/22:4)-H PS(40:4)-H PS (18:0/22:4) (18:0) (22:4) C46 H81 O10 N1 P1 838.5603605 10.23635581 0.018380302 1.012142216 0.95076807 0.410678369 81868737.53 55339590.07 133130809.7 74629161.42 45875082.75 118642872.8 72528887.63 104417628.5 86304602.49 63168701.4 74752873.2 114499853.1 82943445.42 85699837.15 85578496.93

1446NEG PC(17:1/20:4)+HCOO PC(37:5)+HCOO PC (17:1/20:4) (17:1) (20:4) C46 H81 O10 N1 P1 838.5603605 9.27290818 0.009072356 1.234938532 0.259246179 0.401681982 19676870.44 15612693.45 18029295.54 12047356.17 17615095.91 15125974.26 7823869.626 22736673.16 27365097.95 16392610.68 19728268.75 27109947.26 24188203.49 23815284.59 23808907.29

1447NEG PS(18:0/22:4)-H PS(40:4)-H PS (18:0/22:4) (18:0) (22:4) C46 H81 O10 N1 P1 838.5603605 11.37791155 0.082743458 1.111940512 0.478232417 0.186849942 9039362.83 15594343.51 15209862.71 12302660.9 10211712.6 10866798.64 8138295.583 13183630.5 17365636.25 12113153.71 18013425.51 12607414.63 15815361.76 13886107.94 13628745.77

1448NEG PC(18:0p/20:4)+HCOO PC(38:4p)+HCOO PC (18:0p/20:4) (18:0p) (20:4) C47 H85 O9 N1 P1 838.5967455 10.7773761 0.023856769 1.44557918 0.161132327 0.900323894 13274930.95 19403369.43 12845356.54 14393922.64 36487883.03 20598638.76 6583250.771 31933659.22 29650489.35 34746060.77 35907064.75 30318168.04 27153854.75 27145013.94 28286921.47

1449NEG PC(16:0e/22:5)+HCOO PC(38:5e)+HCOO PC (16:0e/22:5) (16:0e) (22:5) C47 H85 O9 N1 P1 838.5967455 10.215 0.004765477 1.071380277 0.784306679 0.461890962 35909645.49 25270682.9 33551907.79 30499585.64 42892275.6 47602653.27 14013855.43 38043441.03 47113977.43 26896323.65 30578962.38 74478826.05 47558094.91 47167397.49 47559647.7

144POS Cer(d18:0/22:0)+H Cer(d40:0)+H Cer (d18:0/22:0) (d18:0) (22:0) C40 H82 O3 N1 624.6289205 12.77387665 0.007776058 1.191833764 0.468868779 0.177114232 17011047.04 15230548.23 15876171.68 9176877.244 16029686.75 22678073.31 11741530.7 21795400.8 24187067.9 11692315.55 11534519.92 33468071.97 25734698.5 26084310.91 26084583.76

1450NEG PG(20:5/22:6)-H PG(42:11)-H PG (20:5/22:6) (20:5) (22:6) C48 H72 O10 N0 P1 839.4868615 6.218849472 0.013798301 2.026341702 0.248237585 0.408510784 511169.6146 603088.6342 581929.3885 669146.2164 3156923.447 708389.7053 126210.2719 1744622.08 932652.022 3214075.218 5270262.194 1337598.074 2291672.93 2237542.182 2237985.247

1451NEG CerG2(d33:4)-H CerG2(d33:4)-H CerG2 (d33:4) (d33:4) C45 H78 O13 N1 840.5478675 9.803489355 0.016491208 1.050535537 0.873054813 0.365320757 10740243.55 11272773.5 12779292.99 8772297.982 9458517.487 7968562.732 2566040.791 14677304.49 23004135.87 5743106.617 6256053.501 11827294.67 14855195.55 14858529.51 15285297.66

1452NEG PS(40:3)-H PS(40:3)-H PS (40:3) (40:3) C46 H83 O10 N1 P1 840.5760105 11.9544332 0.002194078 1.156862821 0.159529102 0.269228082 6105058.202 6609963.396 6873675.19 8212298.343 6301937.358 5804018.798 5952903.736 9716246.523 7032727.182 8007351.303 8923177.321 6534462.197 7998660.386 8029178.857 7998826.371

1453NEG PC(17:0/20:4)+HCOO PC(37:4)+HCOO PC (17:0/20:4) (17:0) (20:4) C46 H83 O10 N1 P1 840.5760105 9.943715878 0.002558052 1.205028183 0.245807965 0.128819141 21362999.57 19429174.48 14004301.89 8897015.708 9806715.618 11375202.56 13008131.16 19057978.79 20194236.16 16524480.51 17528747.46 15963686.81 17248027.61 17324444.55 17247796.14

1454NEG PC(18:0e/20:4)+HCOO PC(38:4e)+HCOO PC (18:0e/20:4) (18:0e) (20:4) C47 H87 O9 N1 P1 840.6123955 10.8132085 0.018695774 1.199496424 0.473480929 0.457343683 21956916.02 18479559.6 20949939.29 19269546.66 25544456.72 24609569.51 8848984.11 31780288.09 33825017.23 16199787.59 19047097.22 47204938.31 29916440.92 28959370.92 29919203.59

1455NEG PE(25:1/18:1)-H PE(43:2)-H PE (25:1/18:1) (25:1) (18:1) C48 H91 O8 N1 P1 840.6487805 12.71662269 0.010830581 1.313964731 0.241380374 0.367898724 3667990.05 4403314.794 4831127.654 6398695.952 6657254.362 3893147.958 1799186.337 10138225.15 6115438.158 5831890.004 7191603.335 8147515.609 7867872.447 7867345.741 7720937.548

1456NEG PG(20:4/22:6)-H PG(42:10)-H PG (20:4/22:6) (20:4) (22:6) C48 H74 O10 N0 P1 841.5025115 7.025146045 0.009863623 1.803020653 0.217371649 0.535964301 1231473.704 1504304.193 1377688.527 1965029.168 5963878.269 1952180.078 991467.0093 3178491.379 1882216.04 6763171.99 8767352.541 3649770.821 4479648.618 4479587.468 4556587.47

1457NEG PG(20:4/22:6)-H PG(42:10)-H PG (20:4/22:6) (20:4) (22:6) C48 H74 O10 N0 P1 841.5025115 7.682049737 0.017057697 1.397699295 0.353312961 0.128725401 710551.3778 1379245.753 559198.4033 1834165.985 1708365.348 752750.6795 209100.6845 2483740.774 2987349.397 1100482.01 1108839.086 1816499.883 2070667.514 2010088.318 2070670.892

1458NEG PC(19:1/18:2)+HCOO PC(37:3)+HCOO PC (19:1/18:2) (19:1) (18:2) C46 H85 O10 N1 P1 842.5916605 10.24261015 0.005520316 1.195728889 0.465577867 0.455151553 32832350.12 24835486.25 30378238.5 44213344.76 47951958.85 31764286.22 13923632.05 64875048.15 63398070.11 28931419.86 32239715.43 50097540.42 41558828.8 41803033.29 42019992.26

1459NEG PS(18:1/22:1)-H PS(40:2)-H PS (18:1/22:1) (18:1) (22:1) C46 H85 O10 N1 P1 842.5916605 11.21064773 0.010424219 1.274154505 0.396368733 0.65222221 16327495.15 7289973.405 28474875.16 20573260.55 17427591.63 47649474.35 25790451.91 31391786.33 11107579.55 42110919.18 29838821.44 35265885.45 20282858.83 20282835.93 20651277.16

145POS DG(17:0/18:1)+NH4 DG(35:1)+NH4 DG (17:0/18:1) (17:0) (18:1) C38 H76 O5 N1 626.5718005 12.06368001 0.007250311 1.044038574 0.8819663 0.102259971 26214792.48 32075368.2 19865080.41 33310816.44 31709923.54 17340934.36 5642334.071 51475406.35 46700762.61 17601320.37 21424786.03 24741242.09 35885028.58 35887443.45 36338781.52

1460NEG PC(17:0/20:3)+HCOO PC(37:3)+HCOO PC (17:0/20:3) (17:0) (20:3) C46 H85 O10 N1 P1 842.5916605 10.56881485 0.029047275 0.945187034 0.726141487 0.203516854 5318897.018 3577552.986 4991016.807 3398568.568 5819301.686 2883936.588 2278561.748 3640037.354 5258884.066 5028775.052 3929623.738 4428842.519 6344845.07 6373859.191 6683768.75

1461NEG PC(20:1p/18:1)+HCOO PC(38:2p)+HCOO PC (20:1p/18:1) (20:1p) (18:1) C47 H89 O9 N1 P1 842.6280455 11.49565799 0.120637556 1.027881929 0.881032517 0.143322757 5273079.768 5362726.438 6511039.387 6826965.539 6749045.799 6549244.039 3526427.181 6419238.393 6526777.942 8187190.589 3370571.692 10281113.26 8504258.466 8445005.915 10377234.4

1462NEG PE(25:0/18:1)-H PE(43:1)-H PE (25:0/18:1) (25:0) (18:1) C48 H93 O8 N1 P1 842.6644305 13.30262198 0.00863704 1.267399062 0.284349312 0.394938378 5170866.948 5148486.323 6711761.569 7950769.683 7203104.247 5692793.026 2447581.139 13275036.15 7555587.909 7502893.078 8243246.616 8981920.23 10411121.47 10411515.46 10256340.5

1463NEG PG(20:3/22:6)-H PG(42:9)-H PG (20:3/22:6) (20:3) (22:6) C48 H76 O10 N0 P1 843.5181615 7.524666535 0.009845738 2.049422512 0.132746617 0.518417624 203559.3882 1015772.467 854291.4114 1962429.619 5097321.916 1498044.554 603117.7607 5480012.367 1956253.123 4520457.826 6282386.484 2946042.596 3536753.425 3536989.996 3476897.581

1464NEG PG(20:3/22:6)-H PG(42:9)-H PG (20:3/22:6) (20:3) (22:6) C48 H76 O10 N0 P1 843.5181615 7.217442043 0.014219836 1.06327248 0.794725996 0.056292764 4883275.983 4594029.049 4140704.865 7151443.743 7554825.209 3937505.916 1406201.688 8110126.288 7957250.412 4007394.238 5173639.535 7648455.722 7932910.354 7741443.54 7739931.66

1465NEG SM(d41:2)+HCOO SM(d41:2)+HCOO SM (d41:2) (d41:2) C47 H92 O8 N2 P1 843.6596795 11.45324123 0.021877491 1.163129723 0.360530946 0.668418737 108467335.9 68007179.76 90997722.79 65301545.3 111057362.5 83777832.6 43277786.39 97191958.96 120797528.7 105602371.6 117198453.6 129609586.1 131192489.2 131207843.7 126290638.3

1466NEG PC(19:1/18:1)+HCOO PC(37:2)+HCOO PC (19:1/18:1) (19:1) (18:1) C46 H87 O10 N1 P1 844.6073105 10.82451299 0.003652377 1.1160267 0.534531488 0.199538512 135805868.9 134866350.2 125850831.2 142267783.5 183777237.3 131377007.9 63262510.1 212523743.2 219840706.4 127696239.7 145063356.6 184638952.7 186285541 187466218.1 186284995.6

1467NEG PS(18:1/22:0)-H PS(40:1)-H PS (18:1/22:0) (18:1) (22:0) C46 H87 O10 N1 P1 844.6073105 11.7338467 0.004386202 1.244355679 0.254242523 1.053616528 20490531.02 21765512.16 43329759.94 49467730.11 36295379.98 50810372.84 32121460.04 53355694.83 30365147.94 54288754.43 61053866.79 45260245.1 35692007.13 35964719.65 35964368.15

1468NEG PC(20:0p/18:1)+HCOO PC(38:1p)+HCOO PC (20:0p/18:1) (20:0p) (18:1) C47 H91 O9 N1 P1 844.6436955 12.07530883 0.012294325 0.940255159 0.744617858 0.63430972 17094823.6 24219197.44 16085035.71 15874993.22 19638241.44 17457706.62 8792464.419 21849846.76 24083666.48 11062938.58 12300032.31 25687011.45 21652878.51 21195467.04 21653743.01

1469NEG PG(20:2/22:6)-H PG(42:8)-H PG (20:2/22:6) (20:2) (22:6) C48 H78 O10 N0 P1 845.5338115 8.214294022 0.008731678 1.580673069 0.219224413 0.52971239 2014713.945 1817693.299 3295245.52 6105285.881 11772911.93 4347447.663 1201655.095 10065255.99 5207967.673 9594787.603 9230076.559 11098225.08 9336633.991 9310353.092 9185043.983

146POS PC(26:3p)+H PC(26:3p)+H PC (26:3p) (26:3p) C34 H63 O7 N1 P1 628.4336685 4.778 0.015191082 1.200157147 0.669823377 0.038702969 338902.4279 240053.5544 357383.2216 118452.9069 193574.1604 185006.9019 133384.6633 435904.4771 689311.2868 70829.53862 52439.41655 338403.6756 397177.0941 404736.4001 409323.1048

1470NEG SM(d41:1)+HCOO SM(d41:1)+HCOO SM (d41:1) (d41:1) C47 H94 O8 N2 P1 845.6753295 12.09706297 0.003081739 1.167911792 0.367092048 0.795665713 159992278.6 125250891.4 156223104.3 114766770 168470205.7 135404217.5 65619243.62 205702814.8 199068793.2 143172628.4 162300772.1 228665401.3 199074234.3 197910850.3 198166637.7

1471NEG CerG2(d32:2+hO)-H CerG2(d32:2+hO)-H CerG2 (d32:2+hO) (d32:2+hO) C44 H80 O14 N1 846.5584325 11.48865686 0.009294635 1.3238156 0.143116909 0.622369879 9059292.906 8990995.627 10378401.56 10130302.64 10892723.35 9957046.277 7824101.938 12815450.09 11785897.67 12746417.02 22353877.28 11120502.38 12224811.01 12029246.44 12225195.6

1472NEG PC(19:0/18:1)+HCOO PC(37:1)+HCOO PC (19:0/18:1) (19:0) (18:1) C46 H89 O10 N1 P1 846.6229605 11.47503602 0.01081721 1.077502545 0.690685157 0.472076644 65886329.24 73958313.37 73019228.14 63965657.66 75702874.51 62523938.29 31326753.53 101918164.2 115060224 53725835.73 60850833.98 84342452.61 92070771.17 90346659.45 92050652.1

1473NEG Cer(d52:3+pO)+HCOO Cer(d52:3+pO)+HCOO Cer (d52:3+pO) (d52:3+pO) C53 H100 O6 N1 846.7556125 14.37394445 0.037627597 1.164225587 0.649290485 0.102869496 687173.5565 624539.2868 522760.7015 866776.4194 812728.0905 569507.297 216259.6473 1298565.663 1366572.978 309499.4039 301127.6094 1262072.831 1082874.495 1082837.392 1154996.001

1474NEG PI(17:1/18:1)-H PI(35:2)-H PI (17:1/18:1) (17:1) (18:1) C44 H80 O13 N0 P1 847.5342065 9.093276353 0.00630724 0.982387068 0.942491667 0.289520156 26970284.65 20447602.65 46988590.08 48584582.02 30244657.58 57135717.4 20807016.54 47913526.16 28324440.65 29545927.23 32262130.41 67460877.07 43750296.98 43275460.43 43753070.48

1475NEG PC(16:1/22:6)+HCOO PC(38:7)+HCOO PC (16:1/22:6) (16:1) (22:6) C47 H79 O10 N1 P1 848.5447105 8.48242432 0.00452564 1.034965687 0.90475204 1.267995615 75411692.97 77561053.56 69055761.78 64511232.58 59784314.92 68623570.46 23115157.78 94556215.6 129465126.8 29033089.66 35791454.99 117495510.2 97980761.12 97216230.66 97983783.32

1476NEG PE(24:1/20:4)-H PE(44:5)-H PE (24:1/20:4) (24:1) (20:4) C49 H87 O8 N1 P1 848.6174805 11.91689564 0.002918376 1.311239464 0.107224994 0.623682112 8844061.649 8729483.353 10002487.94 10675971.73 11332621.51 9903369.859 5302875.768 17474217.03 12493297.29 13136158.91 14609061.68 14987397.34 14530221.88 14527745.41 14602517.03

1477NEG Cer(d52:2+pO)+HCOO Cer(d52:2+pO)+HCOO Cer (d52:2+pO) (d52:2+pO) C53 H102 O6 N1 848.7712625 14.93437516 0.017886915 1.216543863 0.565070863 0.123532836 1142579.147 1079381.033 995858.2319 1123541.599 1378505.393 855382.153 527804.2064 1560302.054 1718865.998 525865.7191 688426.4986 2977812.59 1402647.978 1359471.495 1402305.116

1478NEG PI(17:1/18:0)-H PI(35:1)-H PI (17:1/18:0) (17:1) (18:0) C44 H82 O13 N0 P1 849.5498565 9.858773153 0.029430101 1.105860055 0.652615958 0.400921574 62551948.15 31228749.85 114029789.9 68654889.47 47418798.61 114575531.1 57985170.02 92448384.07 57654791.2 80023740.88 81197397.91 115565591.8 82292308.82 78228234.61 78242546.02

1479NEG PC(18:1/20:5)+HCOO PC(38:6)+HCOO PC (18:1/20:5) (18:1) (20:5) C47 H81 O10 N1 P1 850.5603605 8.827157358 0.00764054 0.985042901 0.947950869 0.853527687 44624932.97 40218705.09 39037463.23 38535003.79 48502822.96 46373346.91 12985628.73 55440790.99 63667077.07 22350244.49 31107209.15 67892978.48 60770062.28 59967580.36 59978279.32

147POS LPC(24:0)+Na LPC(24:0)+Na LPC (24:0) (24:0) C32 H66 O7 N1 P1 Na1 630.4469135 8.273526105 0.007608385 1.051176397 0.824946798 0.073225666 6185071.513 3000311.195 6678395.168 8012868.821 6427586.816 8323186.31 3489168.83 9527757.783 8091390.662 4505286.93 4422015.182 10568612.62 9793401.177 9923165.889 9793671.605

1480NEG PC(18:1/20:5)+HCOO PC(38:6)+HCOO PC (18:1/20:5) (18:1) (20:5) C47 H81 O10 N1 P1 850.5603605 9.100259153 0.006076667 1.202499952 0.296680575 0.946471369 121197827.2 80843537.28 120232349.7 79175970.77 103626789.9 99086538.1 48782814.88 130173567.9 160577614.8 105335876.5 121817024.2 159819096 144967381.4 143451693.5 143452741.4

1481NEG PC(16:0/22:6)+HCOO PC(38:6)+HCOO PC (16:0/22:6) (16:0) (22:6) C47 H81 O10 N1 P1 850.5603605 9.378045347 0.007641717 1.115490663 0.686119625 1.975469896 327722049.1 349138272.5 305994445.2 253640461.9 252964814.7 236174950.5 125627520.5 488114830.4 572464045.1 166097879.5 175195053 397430395.8 394172636.9 388997132.4 394210500.8

1482NEG PE(24:0/20:4)-H PE(44:4)-H PE (24:0/20:4) (24:0) (20:4) C49 H89 O8 N1 P1 850.6331305 12.4955527 0.008177353 1.309615724 0.104822154 0.755001009 14536203.87 11702241.78 15107581.05 17282928.48 14673349.48 15186161.29 8403666.417 25811590.61 17899478.13 20287692.16 20229275.71 23254183.33 23178909.35 22812604.64 22923468.92

1483NEG PC(18:0/20:5)+HCOO PC(38:5)+HCOO PC (18:0/20:5) (18:0) (20:5) C47 H83 O10 N1 P1 852.5760105 9.669216763 0.001366955 1.137667296 0.422510152 0.681349595 366268980.6 358876428.7 368699219 300835011.5 391879015.3 307777412.3 176897727.7 489758793.1 565999880.2 332644626 374193511.1 443163112.6 453475650.8 452399454.6 452407950.8

1484NEG PC(18:0/20:5)+HCOO PC(38:5)+HCOO PC (18:0/20:5) (18:0) (20:5) C47 H83 O10 N1 P1 852.5760105 9.9366248 0.013306346 1.227828463 0.127410365 1.046824617 119457284.1 109037830 107084715.2 81972245.15 107323209.6 90846183.53 64860756.33 135512192.8 156609429.8 125991655.6 139301888 133724420.5 137124482.1 133987666.2 137123324.5

1485NEG PI(16:1/20:5)-H PI(36:6)-H PI (16:1/20:5) (16:1) (20:5) C45 H74 O13 N0 P1 853.4872565 6.940392177 0.039020231 0.801902937 0.498185302 0.139872071 1001933.995 741813.3651 1572801.137 1739163.11 766686.8765 1633983.547 169609.4979 1194868.78 726747.9125 777149.8396 791369.638 2319548.981 1373564.355 1470628.4 1377600.963

1486NEG SM(d22:0/20:4)+HCOO SM(d42:4)+HCOO SM (d22:0/20:4) (d22:0) (20:4) C48 H90 O8 N2 P1 853.6440295 10.64052552 0.012779557 1.109761351 0.523738145 0.152127186 8891762.368 10526184.76 10635457.3 6389924.835 13733473.77 7902329.257 5435329.015 14397711.61 12568503.13 10727416.58 10516843.91 10808172.05 11705560.1 11444690.24 11697887.31

1487NEG PS(41:3)-H PS(41:3)-H PS (41:3) (41:3) C47 H85 O10 N1 P1 854.5916605 10.121 0.004162521 0.95012638 0.813114161 1.11167222 145710416 122199241.2 129926907.5 123532953.8 245546370.4 137155097.1 53923462.16 150941948.6 177619008.7 87747497.09 189922579.1 198827197.5 164663677.3 164326039 163348355

1489NEG PE(26:1/18:1)-H PE(44:2)-H PE (26:1/18:1) (26:1) (18:1) C49 H93 O8 N1 P1 854.6644305 12.97770735 0.001364845 1.108754774 0.677243956 0.333154139 22981626.72 23936811.93 25557016.64 36389465.78 25194349.28 26298775.65 8756020.314 49385623.26 32167667.9 19206965.39 22343347.68 45938124.58 39823427.53 39730299.58 39728591.4

148POS PC(26:2e)+H PC(26:2e)+H PC (26:2e) (26:2e) C34 H67 O7 N1 P1 632.4649685 7.186407825 0.010259805 0.935984382 0.779001833 0.100277471 1920282.366 1774146.234 2136796.603 1753620.472 2142117.686 2289936.772 1029137.931 2338039.876 2893806.101 770113.885 1014059.68 3202473.375 2872113.082 2924810.232 2874884.675

1490NEG PI(16:0/20:5)-H PI(36:5)-H PI (16:0/20:5) (16:0) (20:5) C45 H76 O13 N0 P1 855.5029065 7.687098138 0.009895038 0.792208329 0.433686376 0.526264323 7699633.751 6769613.692 11652017.22 13992902.31 6477860.835 14781672.5 3824912.013 8348441.638 7535766.604 4823802.768 5967127.173 18120706.35 11237850.48 11045698.64 11236533.95

1491NEG PI(16:0/20:5)-H PI(36:5)-H PI (16:0/20:5) (16:0) (20:5) C45 H76 O13 N0 P1 855.5029065 7.94922221 0.001797653 0.975993334 0.92749041 0.327664936 13073966.36 9199436.29 20008850.68 14198639 9746705.592 23307585.74 8787653.323 16099654.07 11862256.74 10601379.09 10956376.42 29078422.76 17373339.19 17319365.13 17319349.17

1492NEG SM(d42:3)+HCOO SM(d42:3)+HCOO SM (d42:3) (d42:3) C48 H92 O8 N2 P1 855.6596795 11.19777626 0.006680626 1.145311959 0.413812834 0.586807206 138606214.2 108763820.4 131212994.1 101240977.9 181995965.4 129792296 66687667.61 187154193 173080805.6 128506184.9 159598594.1 191615552.4 174529276.4 174534984.6 172520352.9

1493NEG PC(18:0/20:3)+HCOO PC(38:3)+HCOO PC (18:0/20:3) (18:0) (20:3) C47 H87 O10 N1 P1 856.6073105 10.59726672 0.009072236 1.072291998 0.686249423 0.843124946 147353931.6 140337011.9 128740563.7 133515667 147560454.8 128589466.2 73441905.46 186952069.2 225793167.9 117777753 104446872.3 177405537 178012430.4 178011697.5 175229433.5

1494NEG PE(26:0/18:1)-H PE(44:1)-H PE (26:0/18:1) (26:0) (18:1) C49 H95 O8 N1 P1 856.6800805 13.55578939 0.007275737 1.082325396 0.778859966 0.549907345 28715207.24 29356362.38 42209860.32 50842960.35 30886832.33 37963895.08 13463526.71 67245526.12 43410438.83 22007354.51 23093672.8 68864137.38 54220061.88 54222791.77 53540993.65

1495NEG PI(16:0/20:4)-H PI(36:4)-H PI (16:0/20:4) (16:0) (20:4) C45 H78 O13 N0 P1 857.5185565 8.61493066 0.003468214 1.036862737 0.863307685 0.678820034 132066636.3 116928186.7 214311657.3 182214071.1 121596782.4 263831867.1 131510670.8 223413430.8 161594008.5 127175873.7 136476984 288781842.8 205108028.1 203880294.5 203881373.2

1496NEG PI(18:2/18:2)-H PI(36:4)-H PI (18:2/18:2) (18:2) (18:2) C45 H78 O13 N0 P1 857.5185565 8.223871877 0.012282505 0.906779184 0.719528795 0.445920771 13080639.36 10055382.75 21718370.51 18601786.68 10296239.04 20633642.32 7535958.342 15488432.39 11748665.77 10573720.21 10105885.68 30134652.65 18866042.06 18469082.54 18869228.64

1497NEG SM(d42:2)+HCOO SM(d42:2)+HCOO SM (d42:2) (d42:2) C48 H94 O8 N2 P1 857.6753295 11.75005466 0.024494411 1.074767558 0.669471519 0.31293324 1015348743 814735554.7 987760697.9 857070793.4 1100618751 915294587.8 421739554.9 1330259507 1015218202 818678761.3 1032100708 1498321793 1046657593 1091806587 1046863406

1498NEG PC(20:1/18:1)+HCOO PC(38:2)+HCOO PC (20:1/18:1) (20:1) (18:1) C47 H89 O10 N1 P1 858.6229605 11.13150636 0.004344374 0.991128745 0.955876992 1.649105417 280454580.4 334555652.1 267674728.3 291890956.1 441866410 311916956.2 156935153.4 435067315.1 389092391.5 238617189.4 306894083.4 384646182.6 397888778.1 397909580.7 394912662

1499NEG PI(18:1/18:2)-H PI(36:3)-H PI (18:1/18:2) (18:1) (18:2) C45 H80 O13 N0 P1 859.5342065 8.832451648 0.003531967 0.998457627 0.992986545 0.416557094 57364700.81 51897983.65 90353135.53 102899415.3 77744667.32 108821614.8 44489791.74 99110218.4 66691657.04 78817374.27 83477764.69 115740364.9 93401042.57 93975295.38 93974238.58

149POS LPC(26:1)+H LPC(26:1)+H LPC (26:1) (26:1) C34 H69 O7 N1 P1 634.4806185 8.247669475 0.00344024 0.952825694 0.886352595 0.427649604 33966376.39 21377897.36 39880159 37623703.38 24275091.8 41617573.76 14738159.68 40461700.36 43233488.35 9507799.173 9886380.074 71537814.72 48362120.03 48652532.78 48365459.69

14POS AcCa(12:0)+H AcCa(12:0)+H AcCa (12:0) (12:0) C19 H38 O4 N1 344.2795355 1.670586982 0.016814543 0.991881202 0.981171263 0.079571141 708001.3173 3098006.525 1310964.969 5533249.6 3899663.835 5019329.987 3029553.819 3634122.049 4887165.798 1207577.034 1105757.698 5546161.318 4550060.261 4684791.53 4685601.565

1500NEG PI(18:0/18:3)-H PI(36:3)-H PI (18:0/18:3) (18:0) (18:3) C45 H80 O13 N0 P1 859.5342065 9.303285813 0.009486962 1.100422063 0.685297696 0.110004417 21258122.12 18166734.59 36733554.11 32485658.28 21090456.94 50525662.79 23296725.92 38681351.47 20308702.05 30325557.39 30424108.68 55325843.43 34414857.65 33855754.04 34421542.36

1501NEG SM(d16:1/26:0)+HCOO SM(d42:1)+HCOO SM (d16:1/26:0) (d16:1) (26:0) C48 H96 O8 N2 P1 859.6909795 12.3721419 0.001902697 1.104009532 0.565583511 1.867888178 1260916999 1072589824 1271989657 1061038182 1234094829 1120616339 522381642.5 1654908106 1651365614 949224940 1199057912 1774584111 1582524804 1587736402 1582506005

1502NEG PC(16:0/22:1)+HCOO PC(38:1)+HCOO PC (16:0/22:1) (16:0) (22:1) C47 H91 O10 N1 P1 860.6386105 11.81326681 0.011825139 1.036576341 0.88375773 1.443266073 133099660.4 132416288.9 126561838.8 143678866.5 101661597.5 111688810.7 58809505.09 193334580.8 215990025.9 64200637.93 68746382.65 175425525.6 158496831 158499027.4 161766558.1

1503NEG PI(18:1/18:1)-H PI(36:2)-H PI (18:1/18:1) (18:1) (18:1) C45 H82 O13 N0 P1 861.5498565 9.522605817 0.028046035 0.914502434 0.6739579 2.042657653 293573531.1 245434204.9 436830054.3 485310881.4 326276930.8 567365795.6 249243097.4 413670634 278831688.4 302323874.9 281608674.8 627784495.3 448144346.7 426952622 427164232.6

1504NEG PI(18:0/18:2)-H PI(36:2)-H PI (18:0/18:2) (18:0) (18:2) C45 H82 O13 N0 P1 861.5498565 9.826815432 0.020271277 1.112716133 0.666752509 0.54448048 48176265.35 28768596.69 108734620.3 127416815 79651327.9 121993431.8 70726352.74 133777945.6 53103481.43 79758080.57 96841081.83 138553736.3 77402175.84 80197715.87 80172046.35

1505NEG PC(18:0p/22:6)+HCOO PC(40:6p)+HCOO PC (18:0p/22:6) (18:0p) (22:6) C49 H85 O9 N1 P1 862.5967455 10.56533303 0.005864337 1.267518657 0.348340818 0.215339021 6427652.831 10761975.47 5426572.841 7253051.781 16867318.49 9184146.149 3471647.987 14080300.73 15323430.27 10236793.8 11926733.82 15841646.23 13536989.69 13675008.4 13537092.54

1506NEG PC(18:1p/22:5)+HCOO PC(40:6p)+HCOO PC (18:1p/22:5) (18:1p) (22:5) C49 H85 O9 N1 P1 862.5967455 10.07796478 0.125634836 1.185311068 0.510283329 0.054625962 4641132.437 5305718.379 4240616.132 4098899.431 10289954.19 7005283.516 2284362.531 6366209.295 8864839.018 6142893.042 6782945.05 11734020.21 9275966.015 11461822.59 9291407.396

1507NEG PI(18:0/18:1)-H PI(36:1)-H PI (18:0/18:1) (18:0) (18:1) C45 H84 O13 N0 P1 863.5655065 10.2640913 0.011051513 1.169581909 0.396893191 1.706285505 349127035.1 181921511.1 559498424.1 455999462.8 297384940.2 620675728.5 410517559.9 509899466.6 329574479.5 490963651.8 528308854.4 613295866.2 429578824.8 437963929.9 437850944.4

1508NEG PC(17:1/22:5)+HCOO PC(39:6)+HCOO PC (17:1/22:5) (17:1) (22:5) C48 H83 O10 N1 P1 864.5760105 9.28828355 0.035288602 1.071089092 0.788194542 0.31226117 10336581.07 12546069.86 9429698.543 8195247.828 12205112.95 8039499.383 3287022.566 14416757.22 18476094.72 5714547.027 7730224.26 15446383.26 13024629.44 12255365.99 13047360.37

1509NEG PC(18:0p/22:5)+HCOO PC(40:5p)+HCOO PC (18:0p/22:5) (18:0p) (22:5) C49 H87 O9 N1 P1 864.6123955 10.79291331 0.060720504 0.99807797 0.993102758 0.256056997 9216508.063 12251359.93 8613958.616 17089109.34 18329986.74 13807638.02 3943708.061 13355245.78 18628687.27 11621357.47 11580519.5 20026609.23 18672896.92 18571795.45 20650073.51

150POS DG(16:0/20:4)+NH4 DG(36:4)+NH4 DG (16:0/20:4) (16:0) (20:4) C39 H72 O5 N1 634.5405005 11.17349063 0.007085981 0.811304061 0.404235429 0.208486766 28734614.66 18525185.7 32046725.02 25149439.18 13113041.37 12706645.81 7036456.102 29772410.95 17632861.42 15035769.63 12451899.45 23763767.72 25557646.41 25457925.42 25208144.58

1510NEG PG(22:6/22:6)-H PG(44:12)-H PG (22:6/22:6) (22:6) (22:6) C50 H74 O10 N0 P1 865.5025115 6.752966758 0.003497569 1.542656507 0.275371262 1.518700378 21796582.88 47253318.32 23082079.38 48269711.16 127587557.6 28045518.47 15496510.74 131536239.9 87129826.81 75786818.61 98256140.99 48474423.91 86650346.77 86128110.28 86126958.09

1511NEG PI(18:0/18:0)-H PI(36:0)-H PI (18:0/18:0) (18:0) (18:0) C45 H86 O13 N0 P1 865.5811565 10.87666246 0.012343566 1.546008603 0.119098843 0.594427516 5676476.737 1717078.4 10530666.06 5095782.327 2798602.727 6836862.213 6782429.048 9676529.397 3831791.681 11467651.67 11084857.27 7642376.099 6751033.954 6607416.229 6750410.518

1512NEG PS(42:4)-H PS(42:4)-H PS (42:4) (42:4) C48 H85 O10 N1 P1 866.5916605 11.988 0.008525014 1.237912612 0.283267272 0.555885941 6495526.901 7432538.602 7466565.179 10824541.66 10069567.02 7785419.041 5567185.864 13332394.61 7494844.611 12597935.52 15364898.6 7630173.038 10312020.01 10161153.2 10161325.93

1513NEG PC(17:0/22:5)+HCOO PC(39:5)+HCOO PC (17:0/22:5) (17:0) (22:5) C48 H85 O10 N1 P1 866.5916605 9.910735272 0.006053847 1.242060674 0.225810997 0.257682778 11882832.54 12380734.18 10356269.5 7680672.227 12428788.61 9539184.104 6411992.107 16155875.1 19557916.43 10457476.52 13305104.12 13936988.75 14492387.95 14339898.46 14343116.41

1514NEG PC(18:0e/22:5)+HCOO PC(40:5e)+HCOO PC (18:0e/22:5) (18:0e) (22:5) C49 H89 O9 N1 P1 866.6280455 10.94021667 0.008176827 1.015144206 0.957604554 0.501381192 13143733.34 14679788.66 11583621.08 10718303.82 13435932.7 15782427.68 4464062.169 13060954.57 18455799.47 8288063.122 7873489.594 28403037.33 15618939.58 15400448.11 15399162.67

1515NEG PC(20:0p/20:4)+HCOO PC(40:4p)+HCOO PC (20:0p/20:4) (20:0p) (20:4) C49 H89 O9 N1 P1 866.6280455 11.34708054 0.010709343 1.645571367 0.256087767 0.42725405 2203164.707 4177280.058 2240363.086 2005299.913 6302669.229 3384675.072 1253634.351 4991118.718 5431181.301 2645318.447 13140541.08 5965441.18 5341613.055 5342661.194 5243658.23

1516NEG PG(22:5/22:6)-H PG(44:11)-H PG (22:5/22:6) (22:5) (22:6) C50 H76 O10 N0 P1 867.5181615 7.111465473 0.011001756 1.416443338 0.300129024 0.572419866 4533046.661 8430196.509 4355201.279 10327709.71 23374885.47 6649444.202 3452445.525 17123916.62 15980866.9 14418080.15 18604112.99 12107550.46 16194599.47 15894375.38 15885559.18

1517NEG PS(42:3)-H PS(42:3)-H PS (42:3) (42:3) C48 H87 O10 N1 P1 868.6073105 12.49959288 0.002948434 1.363445793 0.155794982 0.57857707 5031442.752 4492840.009 6074591.292 7965768.021 6027753.021 5604504.276 4407554.203 9763435.356 5225688.596 11248008.46 11463299.62 5881078.146 7858727.651 7818692.068 7818769.188

1518NEG PC(20:0e/20:4)+HCOO PC(40:4e)+HCOO PC (20:0e/20:4) (20:0e) (20:4) C49 H91 O9 N1 P1 868.6436955 11.59119435 0.016878938 1.165672918 0.488975425 0.329874066 14588985.34 13329739.56 13981181.19 11977660.03 15680122.68 13723119.03 5624176.623 20463875.46 25602908.31 10963352.05 12122720.28 22301149.58 19500533.63 19506732.67 18938969.15

1519NEG PE(27:1/18:1)-H PE(45:2)-H PE (27:1/18:1) (27:1) (18:1) C50 H95 O8 N1 P1 868.6800805 13.286 0.004142595 0.930514157 0.802001926 0.177270721 1125951.764 1285489.803 1535934.16 1500905.846 985346.1415 1518347.071 409586.0809 2120040.05 1639896.913 454021.4633 587299.1319 2188581.473 1917069.58 1930856.044 1917065.92

151POS LPC(26:0)+H LPC(26:0)+H LPC (26:0) (26:0) C34 H71 O7 N1 P1 636.4962685 9.403957897 0.009720091 0.792737497 0.298713797 0.800609794 91991386.74 63025208.45 94137980.99 97108005.35 62407050.95 101962877.8 58954250.33 84871469.19 77365611.51 29473025.28 31748436.06 122384745.8 95070759.27 95083288.99 96686708.33

1520NEG PI(17:1/20:4)-H PI(37:5)-H PI (17:1/20:4) (17:1) (20:4) C46 H78 O13 N0 P1 869.5185565 8.223331908 0.000389845 1.021281709 0.945289159 0.271248176 11146636.41 9032716.934 22056398.82 23336466.9 11737887.89 23852707.45 7120517.26 23105723.17 14245337.69 12719819.22 10650723.59 35473611.03 20470667.48 20482063.85 20466685.92

1521NEG PG(22:4/22:6)-H PG(44:10)-H PG (22:4/22:6) (22:4) (22:6) C50 H78 O10 N0 P1 869.5338115 7.807634618 0.025476651 1.517671075 0.273363981 0.319115608 852038.9705 1599448.44 987418.3381 1823458.187 5398987.112 1417477.571 506437.1107 3867224.285 3074819.522 3451701.234 4621669.986 2809836.675 3957260.19 3787411.5 3961852.335

1522NEG SM(d43:3)+HCOO SM(d43:3)+HCOO SM (d43:3) (d43:3) C49 H94 O8 N2 P1 869.6753295 11.5136419 0.005694757 1.119468515 0.526949243 0.061913699 10960947.99 8886222.425 10201552.6 7951327.846 13099033.2 9766269.642 4011600.394 12977318.81 14437643 9669251.104 11478942.41 15562091.39 13523166.05 13657146.49 13657724.02

1523NEG PS(18:1/24:1)-H PS(42:2)-H PS (18:1/24:1) (18:1) (24:1) C48 H89 O10 N1 P1 870.6229605 11.70026781 0.041721101 1.361398867 0.211212341 0.974966731 9989430.116 14700453.09 16372032.67 26743731.97 29308136.62 27824793.75 13854440.16 44463397.19 19321771.3 31699911.07 35409344.91 25342374.18 23668732.35 23615966.85 25392375.86

1524NEG PC(16:1p/24:1)+HCOO PC(40:2p)+HCOO PC (16:1p/24:1) (16:1p) (24:1) C49 H93 O9 N1 P1 870.6593455 11.806 0.102455451 1.537433327 0.187325266 0.605129362 7768963.084 9256920.737 7279412.314 9056107.475 11399585.21 10534136.46 4014628.838 23333946.42 13526922.5 7974666.014 11064640.09 25097764.54 13873977.88 16500294.8 13889985.89

1525NEG PI(17:0/20:4)-H PI(37:4)-H PI (17:0/20:4) (17:0) (20:4) C46 H80 O13 N0 P1 871.5342065 8.921846808 0.030218209 1.202549478 0.230547511 0.803374765 31630293.3 39262448.03 57175273.01 53029203.65 40877730.97 59882535.37 38134207.56 82004386.21 50686652.24 54483006.45 57522733.18 56116584.88 63877488.62 63856836.2 67269243.37

1526NEG SM(d43:2)+HCOO SM(d43:2)+HCOO SM (d43:2) (d43:2) C49 H96 O8 N2 P1 871.6909795 12.03966151 0.009675535 1.137064977 0.509638882 0.258195247 61231193.19 55499780.54 61885939.21 48265176.57 83178497.29 57315908.7 24490024.92 86276185.08 93681103.92 56330646.75 61242253.98 95710731.73 89528018.67 88027557.57 88061496.21

1527NEG PC(20:5/20:4)+HCOO PC(40:9)+HCOO PC (20:5/20:4) (20:5) (20:4) C49 H79 O10 N1 P1 872.5447105 8.092940587 0.029947608 1.15219838 0.647667674 0.211680475 4804518.987 2959850.258 4318580.335 1445238.528 1766699.441 2527088.28 1174483.002 3954012.906 4740807.106 1755186.431 2654232.301 6255729.93 4576684.666 4568865.975 4814045.36

1528NEG PS(18:1/24:0)-H PS(42:1)-H PS (18:1/24:0) (18:1) (24:0) C48 H91 O10 N1 P1 872.6386105 12.28177247 0.015000683 1.189495942 0.304005267 0.862291631 22798829.68 31650922.53 36049526.45 52481790.06 38183024.3 59434930.72 34930256.95 57610289.15 37825417.55 56792019.34 56914177.96 42119401.55 39643001.77 38616229.28 39631375.71

1529NEG PC(18:1/21:1)+HCOO PC(39:2)+HCOO PC (18:1/21:1) (18:1) (21:1) C48 H91 O10 N1 P1 872.6386105 11.46648673 0.010988644 1.074703786 0.721085378 0.28267764 25761457.19 23463851.95 24510057.3 27388244.62 31027663.99 22015135.05 9735117.823 37010328.22 42703423.64 19452228.41 22697619.8 34084506.75 35317957.32 35317740.84 34649886.11

152POS LPC(26:0)+H LPC(26:0)+H LPC (26:0) (26:0) C34 H71 O7 N1 P1 636.4962685 8.052375027 0.011018583 1.503979778 0.169542586 0.397756864 12087500.49 12005470.21 12818546.05 17398818.93 13138730.71 15282293.09 4881384.349 20609199.33 30134242.07 15011210.23 17151129.2 36639126.48 26255803.41 26759910.78 26766712.52

1530NEG PC(16:1p/24:0)+HCOO PC(40:1p)+HCOO PC (16:1p/24:0) (16:1p) (24:0) C49 H95 O9 N1 P1 872.6749955 12.65658116 0.009499631 1.197767061 0.368020878 0.174442417 4593443.333 4246929.62 4992280.383 5368512.529 6438476.269 5194828.602 3230912.624 7989213.7 8394900.245 4283661.59 4138843.135 8894982.097 7222559.177 7342560.72 7342870.79

1531NEG PI(17:0/20:3)-H PI(37:3)-H PI (17:0/20:3) (17:0) (20:3) C46 H82 O13 N0 P1 873.5498565 9.269966997 0.024026421 1.059378581 0.820385488 0.224499499 5647560.022 4814977.639 7844412.759 7135968.718 5233231.45 11143007.2 7424023.405 11709386.29 7981663.508 3497023.337 2832934.498 10857288.99 8016610.975 7698246.445 7686096.496

1532NEG SM(d25:0/18:1)+HCOO SM(d43:1)+HCOO SM (d25:0/18:1) (d25:0) (18:1) C49 H98 O8 N2 P1 873.7066295 12.65790086 0.050407282 1.11553956 0.560713686 0.449085764 88841186.07 66412523.91 73054617.84 64969943.06 79007948.43 63574179.98 29834199.52 114173941.9 106719004.5 64063666.45 67804381.98 103624323.7 110837930.8 110872891.4 101450603.4

1533NEG PS(43:7)-H PS(43:7)-H PS (43:7) (43:7) C49 H81 O10 N1 P1 874.5603605 8.710478762 0.006516529 1.09230706 0.737703298 0.595960847 27394839.33 26235960.6 24107611.59 19421258.28 21743203.23 18720052.86 8877211.455 30185902.09 44114080.54 12706075.32 15882054.77 38561169.44 31259228.38 30908827.12 30909256.77

1534NEG PI(19:1/18:1)-H PI(37:2)-H PI (19:1/18:1) (19:1) (18:1) C46 H84 O13 N0 P1 875.5655065 10.177 0.028601917 1.016671532 0.935523739 0.145028405 3187591.982 3286944.958 5888980.255 6652053.561 3138889.38 6562088.975 4731767.875 5751231.287 6514437.89 3407850.99 2620486.396 6169523.535 5936298.835 5944081.075 6239330.707

1535NEG PI(19:1/18:1)-H PI(37:2)-H PI (19:1/18:1) (19:1) (18:1) C46 H84 O13 N0 P1 875.5655065 9.872144388 0.050869531 0.85442882 0.559512871 0.471650662 23661073.8 12214074.25 30450603.2 29390385.07 17937188.73 51992717.68 12206742.99 32116929.07 19108478.15 19759665.41 22909314.47 35431622.78 34195762.44 34291639.54 31313763.2

1536NEG PC(18:1/22:6)+HCOO PC(40:7)+HCOO PC (18:1/22:6) (18:1) (22:6) C49 H83 O10 N1 P1 876.5760105 9.41995541 0.003813938 1.034413013 0.898902347 2.177705842 262669588.2 261130947.7 245311303.1 236390944.2 215441105.7 214635700.1 100825966.5 350771292.9 451622962.8 115930361.5 119025444.2 346806180.8 320831558.1 318708240.1 318734929.4

1537NEG PC(22:5/18:2)+HCOO PC(40:7)+HCOO PC (22:5/18:2) (22:5) (18:2) C49 H83 O10 N1 P1 876.5760105 9.094718458 0.001074263 0.908824433 0.792523422 0.614116054 29622335.3 16452840.9 30026365.66 25701523.34 26307608.87 22505528.82 8572503.403 42284412.51 54167179.77 11777411.07 7599383.641 12482794.74 37851414.35 37851704.08 37781173.75

1538NEG PE(26:1/20:4)-H PE(46:5)-H PE (26:1/20:4) (26:1) (20:4) C51 H91 O8 N1 P1 876.6487805 12.44874047 0.011297633 0.933060418 0.754063684 0.099160089 6015378.011 5009529.703 7669061.162 6954161.541 4577543.19 6461105.842 3150419.799 10183349.46 7792585.601 4719884.842 5796014.365 2588727.711 9052013.401 8876254.219 9052468.179

1539NEG PC(18:1/22:5)+HCOO PC(40:6)+HCOO PC (18:1/22:5) (18:1) (22:5) C49 H85 O10 N1 P1 878.5916605 9.71799784 0.034412443 1.364012304 0.443646364 1.436712875 101649274.1 112794790.9 92751851.91 96413186.47 108758311.1 84561010.05 44807384.34 181866326.5 336007448.2 57699194.46 67769704.9 126067657.3 151060255.3 160256799.6 151079551.9

153POS DG(18:1/18:2)+NH4 DG(36:3)+NH4 DG (18:1/18:2) (18:1) (18:2) C39 H74 O5 N1 636.5561505 11.3669022 0.007659861 2.062405604 0.116216617 0.908871172 27976646.17 33854060.65 27576989.66 32377080.25 31553734.81 20232739.66 7247595.579 124002739.5 40055643.46 47865961.06 38909426.14 99892955.37 39969879.23 40505646.32 40504155.6

1540NEG PC(18:0/22:6)+HCOO PC(40:6)+HCOO PC (18:0/22:6) (18:0) (22:6) C49 H85 O10 N1 P1 878.5916605 10.23110735 0.001224856 1.202300833 0.322018436 0.928350291 136994217.1 115925761 125356822 103171041.2 92201920.37 93504707.22 65193742.92 169311330.6 204267388.6 103707465.8 114203298.4 145437147.3 153768966 153442746.6 153443663.3

1541NEG PE(26:0/20:4)-H PE(46:4)-H PE (26:0/20:4) (26:0) (20:4) C51 H93 O8 N1 P1 878.6644305 13.03945892 0.006044494 0.99144867 0.974541557 0.327494125 8912088.386 4958485.814 7608207.558 7012630.023 3043997.852 6089005.3 2637466.327 8515454.162 7344828.255 3471408.505 3926378.279 11407140.61 8514627.72 8516739.875 8426858.76

1542NEG PI(16:1/22:6)-H PI(38:7)-H PI (16:1/22:6) (16:1) (22:6) C47 H76 O13 N0 P1 879.5029065 7.425429768 0.007403337 0.919871486 0.788030342 0.256136498 3244561.425 2275896.053 3209165.922 2890167.26 2223165.809 2796325.918 961733.1705 2951273.386 2338010.067 1554940.693 1316344.937 6183699.162 2804803.523 2804807.659 2768992.769

1543NEG PC(18:0/22:5)+HCOO PC(40:5)+HCOO PC (18:0/22:5) (18:0) (22:5) C49 H87 O10 N1 P1 880.6073105 10.27449584 0.043944198 1.027590545 0.93140336 1.035111016 94420181.28 182093431 86754904.21 92598300.91 92033165.21 67734152.05 33499342.58 158960060.2 208218393.3 55336886.88 64950975.18 111654157.7 152086235.9 141031956.2 141107264.5

1544NEG PC(18:0/22:5)+HCOO PC(40:5)+HCOO PC (18:0/22:5) (18:0) (22:5) C49 H87 O10 N1 P1 880.6073105 10.753 0.044856344 1.087511709 0.723004446 0.300521085 15627759.03 12590974.35 14160186.38 8911371.859 10284966.97 8654957.802 5641984.314 16619196.86 22709413.76 8785894.86 8972446.811 13647246.02 13725803.31 13703089.63 14808116.14

1545NEG PI(18:1/20:5)-H PI(38:6)-H PI (18:1/20:5) (18:1) (20:5) C47 H78 O13 N0 P1 881.5185565 8.032629148 0.001818326 1.014369409 0.946975037 0.219316317 36933616.72 27152108.01 54037726.84 50186520.29 39517381.36 65150777.57 24196773.6 53564615.77 36420448.14 40132216.45 42533868.86 80052742.38 54316886.3 54316776.01 54488078.09

1546NEG PI(16:0/22:6)-H PI(38:6)-H PI (16:0/22:6) (16:0) (22:6) C47 H78 O13 N0 P1 881.5185565 8.34169161 0.015698633 1.104977891 0.692345439 0.491344064 31321161.57 23286832.16 56127300.55 45132239.63 25829605.51 53510924.74 26062181.57 59134571.07 43316755.69 27050205.44 29013373.5 75322623.46 49667709.67 48331657.24 49672471.99

1547NEG PI(16:1/22:5)-H PI(38:6)-H PI (16:1/22:5) (16:1) (22:5) C47 H78 O13 N0 P1 881.5185565 7.7076325 0.026791508 0.812807555 0.427637126 0.388582775 4074388.083 4153548.387 6447695.41 6271826.686 2938155.779 5526499.177 1902564.149 5362119.323 4409424.181 2225977.182 2208992.006 7797311.227 5483975.121 5746023.823 5490931.348

1548NEG SM(d44:4)+HCOO SM(d44:4)+HCOO SM (d44:4) (d44:4) C50 H94 O8 N2 P1 881.6753295 11.317 0.018242683 1.0243967 0.914615332 0.259759513 7268959.83 5736659.107 6585072.38 4151130.071 6600580.063 5956435.854 2087677.642 7583876.62 9636626.342 3301611.278 5435435.145 9139182.112 8069017.969 7820013.813 7818629.867

1549NEG PC(18:0/22:4)+HCOO PC(40:4)+HCOO PC (18:0/22:4) (18:0) (22:4) C49 H89 O10 N1 P1 882.6229605 10.82049787 0.017065793 1.187321704 0.408686992 0.420872122 41927202.68 46551574.06 39924019.78 37778200.66 51372598.25 40034100.66 18961548.91 67286981.76 81064993.81 39237753.61 41221850.96 58066333.24 61196807.8 62285113.61 60195984.71

154POS Cer(d18:1/23:0)+H Cer(d41:1)+H Cer (d18:1/23:0) (d18:1) (23:0) C41 H82 O3 N1 636.6289205 12.85774023 0.037093578 1.210311648 0.255753716 0.467821036 40342283.14 32954562.99 38043869.47 38933395.39 46447030.22 38242199.89 19822184.71 61007953.6 56380634.88 37006119.25 49948341.43 60213634.62 59608063.29 59626316.46 63531264.66

1550NEG PE(28:1/18:1)-H PE(46:2)-H PE (28:1/18:1) (28:1) (18:1) C51 H97 O8 N1 P1 882.6957305 13.4923729 0.008522835 0.919655383 0.802587651 0.261313895 2285133.017 2081019.024 3023430.203 3250227.087 1866642.051 2741049.52 653829.2446 3943680.729 3038714.248 700050.0659 797650.7289 4888521.261 3620999.773 3621233.651 3567923.974

1551NEG PI(18:1/20:4)-H PI(38:5)-H PI (18:1/20:4) (18:1) (20:4) C47 H80 O13 N0 P1 883.5342065 8.672439213 0.002756706 1.040831269 0.829658198 0.347113709 505300550.3 527643649.5 802697924.4 932140822.2 649882035.6 998469056.9 424189690.8 1034144556 681092286.3 648268094.1 690388784.8 1118366983 872143869 876314982 872144270.6

1552NEG PI(18:1/20:4)-H PI(38:5)-H PI (18:1/20:4) (18:1) (20:4) C47 H80 O13 N0 P1 883.5342065 8.942506752 0.005979706 1.154239866 0.404574544 1.164231059 131958409.3 132349578.6 221489227.6 213205078.4 192035412.8 280428449.8 139239142.8 290425075 176818369.2 217584351.6 227807462.8 300278538.3 238707666.9 236328122.5 238869915.8

1553NEG SM(d44:3)+HCOO SM(d44:3)+HCOO SM (d44:3) (d44:3) C50 H96 O8 N2 P1 883.6909795 11.80541762 0.004672434 1.061339742 0.78055081 0.573151616 38417655.07 30248541.15 36463832.49 29544560.02 41739564.14 34955905.36 14302129.56 48822466.5 51976098.02 23537732.52 27507219.69 58189796.73 44732642.35 45097033.27 45096210.53

1554NEG PC(18:1/22:2)+HCOO PC(40:3)+HCOO PC (18:1/22:2) (18:1) (22:2) C49 H91 O10 N1 P1 884.6386105 11.29238178 0.021329187 1.230181121 0.395229783 0.495353871 48143718.28 35986668.91 28497149.11 29103420.54 43973857.72 29136964.91 17956817.26 68845200.46 60181324.35 21260881.27 40476879.34 55573198.4 55197283.14 55226351.85 53197081.88

1555NEG PE(46:1)-H PE(46:1)-H PE (46:1) (46:1) C51 H99 O8 N1 P1 884.7113805 13.1006187 0.005624303 0.815457151 0.55344707 2.80714782 189789097.3 132241854.1 189093590.6 143270882.1 86815254.37 186368875.4 69240088.65 143853311 189026240.5 28194647.73 28023703.23 298063389 191499809.8 190199951.6 189376261.5

1556NEG PI(18:0/20:4)-H PI(38:4)-H PI (18:0/20:4) (18:0) (20:4) C47 H82 O13 N0 P1 885.5498565 9.831992282 0.022183604 1.31184101 0.103215394 2.760421209 143470138.1 150794548.8 183027170.1 227577962.7 175884580 205991491.7 149893216.2 280747037.7 169116000.1 290546288.2 321370791.4 213964494.6 232159075.1 241028566.4 231846434.5

1557NEG PI(18:0/20:4)-H PI(38:4)-H PI (18:0/20:4) (18:0) (20:4) C47 H82 O13 N0 P1 885.5498565 9.51451878 0.007554756 1.264037878 0.098906502 7.787600555 1542845245 1839688576 2158988631 2730103612 2366470765 2530603742 1838120063 3574966610 2346494750 3330884567 2939943724 2615326608 2744652227 2708983510 2709113711

1558NEG PI(18:1/20:3)-H PI(38:4)-H PI (18:1/20:3) (18:1) (20:3) C47 H82 O13 N0 P1 885.5498565 9.078346737 0.007554459 1.049560504 0.868581809 1.035572729 48407644.78 88203297.46 84427458.38 96667304.58 58058115.87 117465659.1 40495631.54 151522467 124665549 39528678.47 36087120.59 125374735.4 83518113.83 82430478.16 83518982.02

1559NEG SM(d44:2)+HCOO SM(d44:2)+HCOO SM (d44:2) (d44:2) C50 H98 O8 N2 P1 885.7066295 12.31840745 0.007810714 0.994203473 0.974069645 1.05513498 120086391.5 97697979.44 111446591.6 100836517.7 119680514.1 102689158.6 39057684.31 125332693.3 140775078.5 83300757.04 94455906.67 165733163.3 144534320.3 142620618 144599295.7

155POS Cer(d17:1/24:0)+H Cer(d41:1)+H Cer (d17:1/24:0) (d17:1) (24:0) C41 H82 O3 N1 636.6289205 12.55341945 0.004738804 1.151924814 0.481285102 0.189406533 16882648.58 13371065.48 15594259.16 13246259 14491397.18 14247707.01 7477127.097 20163581.59 21318242.28 12001638.45 12892673.85 27324136.43 23316777.86 23288977.72 23113654.45

1560NEG PC(18:1/22:1)+HCOO PC(40:2)+HCOO PC (18:1/22:1) (18:1) (22:1) C49 H93 O10 N1 P1 886.6542605 11.77360055 0.007540907 1.059768048 0.816412628 0.992702123 78157687.77 73849646.01 70772750.29 90393130.62 74957885.39 68562911.6 29415888.93 124459294.6 127902288.4 40975162.59 46880247.6 114356839.1 94615085.16 95867094.05 95856295.49

1561NEG DGDG(29:4)+HCOO DGDG(29:4)+HCOO DGDG (29:4) (29:4) C45 H75 O17 887.5009785 7.990825678 0.018102436 1.229219575 0.513928863 0.297291832 2496837.204 2041328.495 2781583.356 4431819.116 9252297.669 5859850.916 1869029.279 4820219.927 3742726.907 6293935.307 7859833.454 8435661.613 7028164.908 6809288.649 7026520.499

1562NEG PI(18:0/20:3)-H PI(38:3)-H PI (18:0/20:3) (18:0) (20:3) C47 H84 O13 N0 P1 887.5655065 10.11294367 0.005574789 1.252580057 0.252344667 1.879943379 102045290.3 122872639.5 184653678.6 232511606.3 180742463 243806436.2 137694950.5 343044764.8 172646647.6 214896410.6 221651846.4 246107493.7 201699360.6 202118924.4 199999958.3

1563NEG PI(18:0/20:3)-H PI(38:3)-H PI (18:0/20:3) (18:0) (20:3) C47 H84 O13 N0 P1 887.5655065 9.838550103 0.005427857 1.12945514 0.535245252 0.767290204 257030805.4 235566717.3 435670289.3 445741872 292593369 592362964 290008302.6 566707239.8 425877676 326220469.5 360769172.6 581817919.6 449593793.8 445420599.4 445366604.3

1564NEG SM(d44:1)+HCOO SM(d44:1)+HCOO SM (d44:1) (d44:1) C50 H100 O8 N2 P1 887.7222795 12.92153246 0.003275405 1.065002231 0.773859146 0.878108141 103473244.9 77278313.01 98543479.35 84280938 80012041.21 84304173.74 26669423.89 128716805.9 128870361 60663327.49 75426423.75 141860018.3 128709634.8 128190126.1 127878008.4

1565NEG PC(16:0/24:1)+HCOO PC(40:1)+HCOO PC (16:0/24:1) (16:0) (24:1) C49 H95 O10 N1 P1 888.6699105 12.32404168 0.005478622 0.971309761 0.916890017 1.341318521 76622042.09 81365242.92 70440961.47 93782040.17 60700338.09 68005800.52 27411185.56 110071008.9 127780537.7 29364057.64 32013576 111339159.3 93852734.28 92963063.44 93848912.24

1566NEG PI(18:0/20:2)-H PI(38:2)-H PI (18:0/20:2) (18:0) (20:2) C47 H86 O13 N0 P1 889.5811565 10.34781828 0.030559722 0.952037944 0.819984381 0.457704044 29991886.55 20972956.18 46966259.74 49192576.59 22451337.82 59001737.8 42212499.84 48571079.83 39315770.29 22341500.84 23222352.92 41950539.8 42931472.83 40731002.68 40742080.6

1567NEG PC(16:0/24:0)+HCOO PC(40:0)+HCOO PC (16:0/24:0) (16:0) (24:0) C49 H97 O10 N1 P1 890.6855605 12.86128083 0.002752913 0.977350657 0.940286632 0.861580541 30312245.64 30798653.89 29961949.68 39855239.92 21976200.27 26802698.33 9663977.234 45890702.89 51042216.09 9555637.204 11803689.11 47680520.01 41520098.92 41322510.52 41322991.82

1568NEG PI(18:0/20:1)-H PI(38:1)-H PI (18:0/20:1) (18:0) (20:1) C47 H88 O13 N0 P1 891.5968065 10.90884883 0.070114496 0.999569718 0.998831167 0.10181457 5627132.581 3321958.37 15258469.9 8811108.233 3429019.315 10831006.38 12894701.29 7527542.162 6100775.436 5505656.021 5495173.249 9734503.439 6883678.337 6935072.33 7782681.293

1569NEG PC(20:0e/22:6)+HCOO PC(42:6e)+HCOO PC (20:0e/22:6) (20:0e) (22:6) C51 H91 O9 N1 P1 892.6436955 11.41569133 0.020634625 1.15318215 0.538473916 0.313760818 11621306.55 10892189.73 10380135.27 8213756.244 12721582.08 12373619.09 6283708.806 13798742.87 17502577.75 7707987.662 8760047.579 22290579.2 15058326.39 15058715.43 14526662.42

156POS DG(18:1/18:1)+NH4 DG(36:2)+NH4 DG (18:1/18:1) (18:1) (18:1) C39 H76 O5 N1 638.5718005 11.87400621 0.00470083 0.93068651 0.757769719 0.35125109 271121716.7 303476988.7 240467151.7 394565277.4 359403221.8 215991132.2 67752888.46 490170183 325043531.5 244729276.7 195072036.6 338531225.8 398821125.4 402074366.8 402097667.5

1570NEG PI(17:1/22:6)-H PI(39:7)-H PI (17:1/22:6) (17:1) (22:6) C48 H78 O13 N0 P1 893.5185565 7.952484777 0.011412504 1.044054882 0.864954826 0.107628479 1032998.142 788748.8281 1626380.305 1509352.383 842269.4912 1526770.107 633678.0239 1742447.758 1444249.333 791761.7149 765624.2256 2271527.141 1604417.32 1636033.439 1603820.022

1571NEG PS(24:0/20:4)-H PS(44:4)-H PS (24:0/20:4) (24:0) (20:4) C50 H89 O10 N1 P1 894.6229605 12.46768183 0.007109087 1.276582762 0.240863133 0.571010647 5066264.257 7204818.681 6300813.996 9644866.51 9351106.486 8366478.264 5258593.721 10589933.07 7056254.415 14188986.73 13975023.75 7570205.397 9162747.553 9050555.729 9051131.484

1572NEG PC(20:0e/22:5)+HCOO PC(42:5e)+HCOO PC (20:0e/22:5) (20:0e) (22:5) C51 H93 O9 N1 P1 894.6593455 11.57796806 0.008789763 1.050097432 0.847922278 0.414870558 11134452.87 8829596.488 9898704.941 7491988.935 11323645.77 11139769.65 4878204.736 10649446.87 15153205.44 5327617.065 6909805.459 19896615.2 13864558.94 13863713.6 13654131.27

1573NEG PI(17:0/22:6)-H PI(39:6)-H PI (17:0/22:6) (17:0) (22:6) C48 H80 O13 N0 P1 895.5342065 8.782765863 0.017717347 1.364219812 0.073898512 0.281845328 3363164.856 3736705.099 5673768.818 4678221.002 2821567.521 4703317.209 4782448.266 8459122.908 6081690.394 4364492.962 4501383.858 5884631.298 5784696.396 5614463.714 5607233.789

1574NEG PI(17:1/22:5)-H PI(39:6)-H PI (17:1/22:5) (17:1) (22:5) C48 H80 O13 N0 P1 895.5342065 8.253934195 0.024387755 0.971928129 0.922337153 0.111660551 1263495.94 1068268.955 2438536.4 2767470.282 1153541.733 2490061.724 738929.8324 2460900.902 1793383.7 999840.3892 1397384.846 3477053.249 2224617.034 2133615.858 2132831.296

1575NEG PC(20:5/22:6)+HCOO PC(42:11)+HCOO PC (20:5/22:6) (20:5) (22:6) C51 H79 O10 N1 P1 896.5447105 7.802724485 0.010874108 1.055554461 0.855855503 0.24720978 3860903.051 2822065.389 3895175.392 1885087.538 1786867.151 2535398.01 1086288.059 3583051.51 5106847.612 1362221.595 1659101.737 4920495.23 3938275.338 3864080.183 3865996.312

1576NEG PI(19:1/20:4)-H PI(39:5)-H PI (19:1/20:4) (19:1) (20:4) C48 H82 O13 N0 P1 897.5498565 9.107154778 0.017024302 1.073777454 0.708940307 0.120963488 16440042.2 16476732.7 27969412.17 31409586.09 20622599.88 29973947.9 15853442.26 39076238.62 27084717.9 18881080.57 20286471.09 32252602.18 29287142.75 29290999.52 28433838.2

1577NEG PC(20:4/22:6)+HCOO PC(42:10)+HCOO PC (20:4/22:6) (20:4) (22:6) C51 H81 O10 N1 P1 898.5603605 8.502522568 0.018038122 1.104040217 0.726270981 0.410385891 11513398.84 12449996.13 11275652.21 8182077.255 7539494.221 7395649.815 4146437.204 15372557.78 18775372.4 4996324.791 5631589.37 15505385.76 14327421.47 13885432.3 14329535.23

1578NEG PC(20:5/22:5)+HCOO PC(42:10)+HCOO PC (20:5/22:5) (20:5) (22:5) C51 H81 O10 N1 P1 898.5603605 8.170600357 0.012003509 1.058390537 0.858320709 0.202232727 2340864.841 2395020.565 2572887 1382409.087 1760166.01 1368077.375 595867.5888 2673558.751 3985723.699 829826.8706 1069593.235 3354997.302 2791185.023 2733562.151 2734341.095

157POS Cer(d17:0/24:0)+H Cer(d41:0)+H Cer (d17:0/24:0) (d17:0) (24:0) C41 H84 O3 N1 638.6445705 13.05156837 0.055294438 0.934044065 0.78648315 0.083502985 5776559.062 11071590.74 15616719.28 14383061.48 14096288.61 13967406.52 2556468.849 13964646.47 13485346.71 10500267.35 8182195.327 21281834.68 13121361.88 13557169.86 12158271.68

1580NEG PC(22:5/20:4)+HCOO PC(42:9)+HCOO PC (22:5/20:4) (22:5) (20:4) C51 H83 O10 N1 P1 900.5760105 8.848341852 0.020240822 0.994168103 0.98389991 0.385120911 15869106.48 12955222.7 7131663.022 8696717.43 10425742 9265259.295 4398018.689 16928469.67 20703459.99 5162532.913 7064000.742 9711983.013 15679449.11 15137594.46 15682518.67

1581NEG PC(18:1/23:1)+HCOO PC(41:2)+HCOO PC (18:1/23:1) (18:1) (23:1) C50 H95 O10 N1 P1 900.6699105 12.03833087 0.00627744 1.018638782 0.95143817 0.49797063 12878011.88 13291180.9 13045663 14577889.1 10834054.7 10586518.26 3095825.48 20323016.28 23414433.6 5722255.353 5193777.635 18865894.09 16718527.39 16536663.43 16717016.71

1582NEG PS(26:0/18:1)-H PS(44:1)-H PS (26:0/18:1) (26:0) (18:1) C50 H95 O10 N1 P1 900.6699105 12.82350901 0.04214058 0.847400528 0.396773966 0.39873794 6958512.312 8718902.137 12453551.53 12546722.73 5791131.248 14896652.21 7290817.574 11264642.42 9864032.951 4706046.903 8570760.197 10304833.44 8935468.655 8929275.693 9600578.532

1583NEG PI(39:3)-H PI(39:3)-H PI (39:3) (39:3) C48 H86 O13 N0 P1 901.5811565 10.27510124 0.012868783 1.263940849 0.1877576 0.47764913 9571398.746 8800337.822 16333330.62 20706700.28 17559460.17 20559965.65 15003343.78 28792267.96 19421664.5 16510782.13 18532633.13 19957204.34 16955400.17 17335952.23 17342026.86

1584NEG PC(22:4/20:4)+HCOO PC(42:8)+HCOO PC (22:4/20:4) (22:4) (20:4) C51 H85 O10 N1 P1 902.5916605 9.282918683 0.007867042 1.031810421 0.925883855 0.351146094 5754238.714 7015003.167 5862564.243 4672427.847 4338033.313 3827547.345 1895559.766 9068562.338 10637445.25 1520889.659 1884895.829 7463529.844 7053069.603 6955742.327 6960060.942

1585NEG PC(25:1/16:0)+HCOO PC(41:1)+HCOO PC (25:1/16:0) (25:1) (16:0) C50 H97 O10 N1 P1 902.6855605 12.60636068 0.005464821 0.995663449 0.989305157 0.496356114 11923962.85 12586892.38 9877226.504 16948635.93 9170878.439 10141906.83 3483027.599 19157259.89 22446752.87 4062105.968 4503808.739 16690172.69 16002185.3 16152416.41 15998843.24

1586NEG PI(20:5/20:4)-H PI(40:9)-H PI (20:5/20:4) (20:5) (20:4) C49 H76 O13 N0 P1 903.5029065 6.953549065 0.011768669 1.022828979 0.947447849 0.083495209 660221.6834 905198.0437 1080928.808 954654.5972 484368.8455 704993.902 184320.5222 889098.2984 700342.2862 543672.9811 585335.9441 1996955.011 966515.8379 985990.3595 965824.8987

1587NEG PS(45:6)-H PS(45:6)-H PS (45:6) (45:6) C51 H87 O10 N1 P1 904.6073105 10.22691168 0.020771497 1.179157555 0.517344453 0.449168956 16344500.04 12364531.38 16244113.48 13485739.02 11175491.79 11370072.45 6420099.161 20008879.2 28293557.08 9067097.919 10579777.74 21124012.77 16961472.01 16963430.41 17580119.21

1588NEG PC(25:0/16:0)+HCOO PC(41:0)+HCOO PC (25:0/16:0) (25:0) (16:0) C50 H99 O10 N1 P1 904.7012105 13.11040937 0.011922568 0.978183691 0.947855725 0.335976198 4176752.579 4925413.869 4558555.046 5299258.828 2584971.485 3442251.98 1454849.753 6349717.065 8327842.318 1127894.883 1272732.51 5909038.698 5666988.066 5614043.831 5534236.838

1589NEG PI(20:4/20:4)-H PI(40:8)-H PI (20:4/20:4) (20:4) (20:4) C49 H78 O13 N0 P1 905.5185565 7.701014687 0.009297533 0.901116345 0.727218676 0.21078151 1935845.099 2492675.203 3559401.928 4821775.86 1851499.917 3866337.727 1194477.271 3417193.042 2781129.055 1569118.163 1874757.493 5858790.266 3790442.718 3790951.513 3852069.601

158POS DG(18:0/18:1)+NH4 DG(36:1)+NH4 DG (18:0/18:1) (18:0) (18:1) C39 H78 O5 N1 640.5874505 12.43613164 0.004550063 1.101560245 0.748755086 0.528417319 218826391.2 288673623.9 191725893 280887366.6 221280112.4 178565553.7 52501291.6 519363128.4 368464597.5 170199518.2 156536998.4 253042374.6 358341817.3 361189915.3 361171602.4

1590NEG PC(20:1/22:5)+HCOO PC(42:6)+HCOO PC (20:1/22:5) (20:1) (22:5) C51 H89 O10 N1 P1 906.6229605 10.34210175 0.00145284 0.983067241 0.960487835 0.997745991 65313113.55 64494845.56 49722075.73 42142769.83 41377211.13 32377112.61 18766854.88 80839491.31 107730691.1 18845514.13 19085928.44 45156252.28 59237699.73 59186712.5 59070023.01

1591NEG PI(18:1/22:6)-H PI(40:7)-H PI (18:1/22:6) (18:1) (22:6) C49 H80 O13 N0 P1 907.5342065 8.410452587 0.007334746 1.05206453 0.786303489 0.197656146 37175372.03 40457619.89 58854133.24 75298310.02 41861189.44 62312159.39 32640117.47 79422415.32 59331651.56 43011653.31 43701092.82 74302098.98 65617302.4 64786036.71 65614929.99

1592NEG PC(22:1/20:4)+HCOO PC(42:5)+HCOO PC (22:1/20:4) (22:1) (20:4) C51 H91 O10 N1 P1 908.6386105 10.88739678 0.053236032 1.080568651 0.832020732 0.689345121 34285327.09 49962352.62 29454313.16 32654288.46 31467208.99 20984530.11 8754272.591 56396592.95 81414948.78 14794550.07 16257597.11 37207752.93 51365171.69 56120063.52 51132755.27

1593NEG PI(18:0/22:6)-H PI(40:6)-H PI (18:0/22:6) (18:0) (22:6) C49 H82 O13 N0 P1 909.5498565 9.271998427 0.001305063 1.36074937 0.070779814 1.876118923 94012469.93 96063985.27 160846983.9 152378440.6 96839060.36 156558432.2 126690857.4 250350941 164565818.2 145478636.6 153562531.8 189029408.9 177739928.9 177277975.9 177540066.2

1594NEG PI(18:1/22:5)-H PI(40:6)-H PI (18:1/22:5) (18:1) (22:5) C49 H82 O13 N0 P1 909.5498565 8.722254477 0.007072593 0.999274793 0.996493636 0.380170898 37476282.17 42362439.18 56917974.24 76436815.69 49081848.02 66708050.35 33115183.7 68345728.87 55814300.42 45926087.03 49096594.66 76446933.97 63366511.51 63439746.24 64180413.68

1595NEG PC(20:3/22:1)+HCOO PC(42:4)+HCOO PC (20:3/22:1) (20:3) (22:1) C51 H93 O10 N1 P1 910.6542605 11.4047078 0.001943117 1.125448226 0.685985637 0.636010899 37752607.31 44525144.1 34581305.13 35135215.24 34543587.55 25708128.04 11540939.14 55248529.57 78559749.09 22866917.06 21940514.68 48715220.39 48483393.13 48491888.04 48324800.5

1596NEG PI(18:0/22:5)-H PI(40:5)-H PI (18:0/22:5) (18:0) (22:5) C49 H84 O13 N0 P1 911.5655065 9.531105205 0.004917894 1.159614732 0.198178782 0.187917602 89512032.58 96791422.94 128192969.1 131302448.8 92619917 142278528.6 132648486.5 157057934.6 136283525.4 105920379.7 104429424.6 153006888.2 134763944.2 134508360.9 133514125.6

1597NEG PI(18:0/22:5)-H PI(40:5)-H PI (18:0/22:5) (18:0) (22:5) C49 H84 O13 N0 P1 911.5655065 9.83422642 0.004078826 1.326834434 0.16086267 0.787104639 22175674.21 23935500.56 33295550.25 38692842 27375443.46 40431273.05 27730307.63 66717424.29 45643957.91 33577307.68 29697687.51 43300173.39 42681078.19 42380958.82 42682488.56

1598NEG PC(18:1/24:2)+HCOO PC(42:3)+HCOO PC (18:1/24:2) (18:1) (24:2) C51 H95 O10 N1 P1 912.6699105 11.84381364 0.007205642 1.02790805 0.926322944 0.801364784 32783832.82 35261180.14 29154422.63 37015664.38 28863156.96 30546520.82 10496523.87 53709523.45 60216363.74 10611482.05 16655454.95 47339119.76 42146762.82 41622274.46 42145437.21

1599NEG PI(18:0/22:4)-H PI(40:4)-H PI (18:0/22:4) (18:0) (22:4) C49 H86 O13 N0 P1 913.5811565 9.967933298 0.018024856 1.284340284 0.149707365 0.624975401 36634667.65 45935392.11 60154407.09 61970688.8 43274217.4 70814338.19 61260842.2 101560363.2 78863334.11 51110321.53 48613659.43 68018241.61 66223605.1 66229639.21 64180330.98

159POS PC(24:0)+Na PC(24:0)+Na PC (24:0) (24:0) C32 H64 O8 N1 P1 Na1 644.4261785 6.017142253 0.036611354 0.750105495 0.512898716 0.094557842 1229795.892 544661.285 1514521.037 2183105.143 820136.7455 1153192.468 124443.429 1313272.076 1502468.125 85836.45532 149096.9109 2409727.889 2194678.407 2195071.819 2058573.608

15POS WE(2:0/20:2)+NH4 WE(22:2)+NH4 WE (2:0/20:2) (2:0) (20:2) H44 C22 O2 N1 354.3366555 3.785 0.006043327 0.732244899 0.366630465 0.126629358 7136375.721 1922822.173 2975255.779 1343397.363 3310760.758 2435610.877 1996230.061 1313142.107 3623201.573 1843244.126 2594087.363 2633709.27 7377471.466 7454945.929 7455524.23

1600NEG PI(20:0/20:4)-H PI(40:4)-H PI (20:0/20:4) (20:0) (20:4) C49 H86 O13 N0 P1 913.5811565 10.30336888 0.019621903 1.218003703 0.219407857 0.246948434 6859434.399 6633983.173 11316147.06 11404058.75 7185701.706 13702599.46 9825716.264 16143476.44 11698765.66 9757905.355 9984660.072 12139831.77 11734615.45 12144159.08 12141174.63

1601NEG PC(18:1/24:1)+HCOO PC(42:2)+HCOO PC (18:1/24:1) (18:1) (24:1) C51 H97 O10 N1 P1 914.6855605 12.31398887 0.007191342 0.996471506 0.991022427 1.679100472 103954487.3 107516028.3 100252801.5 129019150.4 78495568.5 103304635.7 33629298.39 150813910.8 185378336.6 31230519.16 39692168.47 179601800.1 133785741.9 132126701.4 133786700.7

1602NEG PI(18:0/22:3)-H PI(40:3)-H PI (18:0/22:3) (18:0) (22:3) C49 H88 O13 N0 P1 915.5968065 10.61071596 0.012747177 1.299582907 0.267947647 0.193182706 3199064.12 3327688.694 7034178.194 8367561.744 3973139.276 7036248.726 7180628.611 11004589.49 6856929.901 4514077.541 4253966.681 8995314.606 6350836.072 6213281.089 6212030.211

1603NEG PC(26:1/16:0)+HCOO PC(42:1)+HCOO PC (26:1/16:0) (26:1) (16:0) C51 H99 O10 N1 P1 916.7012105 12.88110978 0.011082709 1.015941809 0.963832535 2.103376862 155233149.2 144983939.9 150715228.9 192858882.1 96807003.19 147830221.6 44174761.02 215966063.8 262275752.4 37928914.29 46578404.04 295667685.4 210093937.8 206086087.5 210092781.7

1604NEG PC(26:0/16:0)+HCOO PC(42:0)+HCOO PC (26:0/16:0) (26:0) (16:0) C51 H101 O10 N1 P1 918.7168605 13.38545484 0.005648694 0.88133022 0.710446219 1.525830071 65617994.88 58974778.17 62151303.86 76677943.75 36031328.61 56526576.94 17927908.57 80794534.4 94764613.77 10479243.06 11685380.45 98084186.33 76398782.48 75704274.04 75615892.24

1605NEG PC(22:6/22:6)+HCOO PC(44:12)+HCOO PC (22:6/22:6) (22:6) (22:6) C53 H81 O10 N1 P1 922.5603605 8.211497697 0.006609777 1.198722342 0.615680203 0.302370693 4837520.385 5190377.937 4540469.509 3474332.356 2880167.467 3174937.541 1478938.766 7634223.494 10118102.48 1420291.95 2262018.636 5973002.149 6031061.331 5962624.118 5962452.004

1606NEG SQDG(43:12)-H SQDG(43:12)-H SQDG (43:12) (43:12) C52 H75 O12 S1 923.4984755 8.443563522 0.004282883 0.937489576 0.503636987 0.379634515 8387464.097 8288280.289 7624302.146 8243872.122 9797124.429 7984320.216 6937598.268 9074253.967 7068678.328 9549682.439 9259747.375 5289543.121 7699454.914 7691040.371 7752006.765

1607NEG PC(22:5/22:6)+HCOO PC(44:11)+HCOO PC (22:5/22:6) (22:5) (22:6) C53 H83 O10 N1 P1 924.5760105 8.559595832 0.014165637 1.097601858 0.779158209 0.345665503 6154741.791 6919612.112 6024021.091 4425497.704 5005152.88 4599767.981 2189183.403 7976778.443 12989408.74 2187750.075 2528463.301 8490641.392 8281095.533 8277457.813 8484064.354

1608NEG PC(22:5/22:5)+HCOO PC(44:10)+HCOO PC (22:5/22:5) (22:5) (22:5) C53 H85 O10 N1 P1 926.5916605 8.961911432 0.019876785 1.187012606 0.630188998 0.212318367 2639114.416 3619126.492 2758440.912 2111586.098 2206380.841 1767152.515 813891.9645 4063026.95 6526578.498 1034213.084 1626344.242 3861973.747 3622996.288 3751044.638 3750266.534

1609NEG PC(26:1/17:1)+HCOO PC(43:2)+HCOO PC (26:1/17:1) (26:1) (17:1) C52 H99 O10 N1 P1 928.7012105 12.59842296 0.005958229 1.140818499 0.688945397 0.54007705 15558091.86 15795963.18 12649874.63 20646509.79 10843923.75 14992331.56 3562543.357 24400314.4 31170578.04 7622245.886 7888660.382 28584553.23 20344352.39 20135759.41 20135909.03

160POS LPC(25:0)+Na LPC(25:0)+Na LPC (25:0) (25:0) C33 H68 O7 N1 P1 Na1 644.4625635 7.437489918 0.00978229 1.672549004 0.1016247 0.412511893 5235839.73 8595771.342 5741433.955 13116574.13 11357941.11 9657844.004 1614768.419 22607916.75 21407425.58 12651842.99 14792295.31 16750671.36 19574424.45 19911022.36 19908708.13

1610NEG PI(20:4/22:6)-H PI(42:10)-H PI (20:4/22:6) (20:4) (22:6) C51 H78 O13 N0 P1 929.5185565 7.415552632 0.016063136 1.160935672 0.696006934 0.120082528 909001.7014 1617106.986 1264062.752 1670605.845 546037.551 666859.5513 278388.4432 2382578.729 1385586.652 544350.5772 618115.5071 2538686.752 1701124.186 1654300.577 1655021.866

1611NEG PC(27:1/16:0)+HCOO PC(43:1)+HCOO PC (27:1/16:0) (27:1) (16:0) C52 H101 O10 N1 P1 930.7168605 13.16741042 0.006468758 0.961195895 0.912374277 0.839856876 22113454.59 21684338.96 21328201.7 24889407.17 10729436.39 19744484.22 5695888.853 29227465.08 35985338.4 3883994.052 4563756.851 36457399.5 27055646.04 26759387.11 26750328.37

1612NEG PI(22:5/20:4)-H PI(42:9)-H PI (22:5/20:4) (22:5) (20:4) C51 H80 O13 N0 P1 931.5342065 7.773465613 0.003567658 1.023011023 0.934314937 0.158787265 1262995.204 2359735.437 1887413.777 2447782.342 1584879.906 1747774.709 714818.1157 3039392.093 2719251.513 919609.4486 973679.1295 3183638.901 2609902.305 2593931.506 2593751.859

1613NEG PC(26:0/17:0)+HCOO PC(43:0)+HCOO PC (26:0/17:0) (26:0) (17:0) C52 H103 O10 N1 P1 932.7325105 13.65704092 0.021344417 0.90229184 0.775620828 0.319019473 2819017.58 3365465.155 3612377.129 4831610.489 2000518.957 3137417.473 974059.829 5146503.924 5990526.055 539126.9515 616165.0662 4568685.712 4113090.38 3972565.347 4131462.271

1614NEG DGDG(33:9)+HCOO DGDG(33:9)+HCOO DGDG (33:9) (33:9) C49 H73 O17 933.4853285 6.746830187 0.011258219 1.571180565 0.252995 0.557350339 2854544.373 5613483.573 3030547.316 5622537.541 15591858.05 3367816.613 2064363.622 16384777.09 10648652.76 10069325.26 11692987.74 5829325.571 10746734.52 10745721.32 10957146.43

1615NEG PC(22:1/22:5)+HCOO PC(44:6)+HCOO PC (22:1/22:5) (22:1) (22:5) C53 H93 O10 N1 P1 934.6542605 10.995 0.002333268 1.029090887 0.946809618 0.886712535 34992715.15 45254853.26 30892840.29 29858330.27 21767461.94 20811742.15 7056892.867 52819624.99 80727702.07 7009010.318 7239673.979 34065483.98 39643296.52 39483607.25 39483422.92

1616NEG DGDG(33:8)+HCOO DGDG(33:8)+HCOO DGDG (33:8) (33:8) C49 H75 O17 935.5009785 7.112765737 0.012690459 1.368687119 0.408640658 0.197144445 497991.7295 1138169.685 417615.3851 1190800.109 3317281.723 899294.1131 350324.6215 2245844.728 1880332.953 2000386.448 2371661.355 1363433.548 1983589.517 1943121.075 1938486.867

1617NEG PI(20:3/22:4)-H PI(42:7)-H PI (20:3/22:4) (20:3) (22:4) C51 H84 O13 N0 P1 935.5655065 9.035 0.012779807 0.790099086 0.48170095 0.305252002 2093119.938 1997611.08 3267289.672 3884718.964 1058353.223 3489519.709 1334957.008 3282522.214 2634715.37 533238.5743 553133.9386 4137581.47 2795615.165 2751875.093 2822521.084

1618NEG DGDG(16:0/16:1)+HCOO DGDG(32:1)+HCOO DGDG (16:0/16:1) (16:0) (16:1) C48 H87 O17 935.5948785 9.920909793 0.035682145 0.784616165 0.533707441 0.730307823 10331890.26 9797622.963 11276440.11 9900811.539 7119287.391 18385704.63 3089936.768 10588022.86 11088064.83 2347333.681 1923244.398 23384981.94 12822550.06 12059868.61 12063008.65

1619NEG PC(24:1/20:4)+HCOO PC(44:5)+HCOO PC (24:1/20:4) (24:1) (20:4) C53 H95 O10 N1 P1 936.6699105 11.41843919 0.001250079 1.032472751 0.929766504 0.733637307 24762594.59 28343445.63 21691187.18 20602188.17 18100950.71 15681402.37 6159285.166 29327800.06 51750861.61 6886919.15 8175848.774 31075941.32 30155561.86 30104394.91 30082146.59

161POS Cer(d42:4)+H Cer(d42:4)+H Cer (d42:4) (d42:4) C42 H78 O3 N1 644.5976205 12.58412774 0.004636461 1.035937943 0.585525616 0.462047442 117233399 103270961.1 111668635 94875102.74 124871204.2 117030344.8 112273484.2 114886888.7 100142203.9 118076782.6 140226574.5 107384387.2 115806245.7 115806726 114878974.8

1620NEG PS(47:4)-H PS(47:4)-H PS (47:4) (47:4) C53 H95 O10 N1 P1 936.6699105 11.782 0.085220944 0.936892067 0.799725155 0.445829926 11276882.39 10273645.97 12645170.28 10836711.15 17369197.16 15681996.55 5072259.898 17856929.77 23156478.99 7254734.519 6844489.845 12971015.65 9213733.571 10694547.4 10748230.35

1621NEG PC(24:0/20:4)+HCOO PC(44:4)+HCOO PC (24:0/20:4) (24:0) (20:4) C53 H97 O10 N1 P1 938.6855605 12.25904742 0.024078898 1.254231676 0.489546089 0.27061297 5685049.55 9213942.33 6477768.551 5706536.309 4359042.225 5093929.473 5913436.598 9874198.748 16423604.21 3007053.369 3305186.499 7301465.769 7313954.533 7309793.698 7621100.076

1622NEG PC(24:1/20:3)+HCOO PC(44:4)+HCOO PC (24:1/20:3) (24:1) (20:3) C53 H97 O10 N1 P1 938.6855605 11.91150088 0.002591634 1.056039933 0.87564688 0.671030238 20103450.98 22486304.87 21285794.73 19484175.18 16422450.16 15609869.25 4951887.196 31669741.02 42903760.47 6053354.565 7480685.675 28799178.77 25798485.21 25667789.98 25756378.01

1623NEG PC(26:1/18:2)+HCOO PC(44:3)+HCOO PC (26:1/18:2) (26:1) (18:2) C53 H99 O10 N1 P1 940.7012105 12.32365202 0.002971893 1.016246796 0.960860199 0.998955559 36527805.76 37749519.32 34338723.57 39035321.25 29433135.02 33366104.16 11488973.8 52982593.36 67656580.16 10164870.39 10994619.54 60582119.9 47902448.93 47777582.94 47619196.05

1624NEG PS(47:1)-H PS(47:1)-H PS (47:1) (47:1) C53 H101 O10 N1 P1 942.7168605 12.8345418 0.004062157 1.004130314 0.990976587 2.116468338 146092547.6 140530393.7 140135651.9 178187215.8 91013853.25 134098652 39177043.62 210780295.5 256829495.6 27481206.96 32827996.42 266390677.5 191245579.1 189910357.5 189902246.6

1625NEG PC(26:0/18:1)+HCOO PC(44:1)+HCOO PC (26:0/18:1) (26:0) (18:1) C53 H103 O10 N1 P1 944.7325105 13.41084912 0.004810145 0.944150725 0.877868435 2.570967528 206113614.1 167431374.3 194106145.3 223080808.6 87398373.59 169452103.3 50191071.84 241841290.2 321354771.9 27740014.59 31092969.36 316855582.9 233619401.4 231690650 231676966.1

1626NEG PC(26:0/18:0)+HCOO PC(44:0)+HCOO PC (26:0/18:0) (26:0) (18:0) C53 H105 O10 N1 P1 946.7481605 13.93749507 0.003997964 0.842073944 0.640245362 0.884840121 22349998.11 15509635.46 22729017.53 22389975.6 8173789.289 16185279.69 5319132.55 21912807.06 26907910.1 2162082.4 2756796.869 31327547.72 22813916.94 22655917.38 22657421.12

1627NEG PS(48:1)-H PS(48:1)-H PS (48:1) (48:1) C54 H103 O10 N1 P1 956.7325105 13.09228388 0.021001424 0.868263003 0.724749346 0.768750652 15711043.53 12674799.73 13813890.83 16238484.65 5333034.566 12465637.69 3015249.383 14394775.95 22809611.3 1268568.037 1161030.051 23544437.23 16677966.25 16087871.01 16083472.86

1628NEG PC(27:0/18:1)+HCOO PC(45:1)+HCOO PC (27:0/18:1) (27:0) (18:1) C54 H105 O10 N1 P1 958.7481605 13.69188482 0.008870067 0.968255156 0.934276668 0.51604388 8051588.963 6769785.676 8561648.721 8638411.579 3299243.448 6855992.279 1922015.16 10594292.25 13277390.49 926895.9094 970495.8265 13146689.21 9787051.439 9638947.572 9637489.89

1629NEG DGDG(16:0/18:1)+HCOO DGDG(34:1)+HCOO DGDG (16:0/18:1) (16:0) (18:1) C50 H91 O17 963.6261785 10.6702307 0.00342618 0.894289997 0.734149112 0.694301904 12452878.95 13013899 10503399.96 12870638.2 10617030.61 20944104 4160648.538 16198454.59 18095454.67 4082309.39 4056271.129 25309521.98 15641662.2 15549239.83 15642097.17

162POS Cer(d18:1/24:2)+H Cer(d42:3)+H Cer (d18:1/24:2) (d18:1) (24:2) C42 H80 O3 N1 646.6132705 12.78313615 0.029348008 1.0463561 0.677306908 0.120830959 9134767.711 7851695.858 9051869.975 8878156.215 10847901.63 11732323.95 13843064.05 8013116.657 8762233.204 10623683.55 10098141.15 8821800.241 9547837.239 9090316.239 9067316.342

1630NEG PC(26:0/20:5)+HCOO PC(46:5)+HCOO PC (26:0/20:5) (26:0) (20:5) C55 H99 O10 N1 P1 964.7012105 12.2973783 0.013112679 1.78705302 0.290389691 0.545332274 7271011.181 5506049.538 5872913.936 5094966.972 2861794.071 5704250.654 2994685.684 7637527.13 27774403.43 4309850.023 5561536.612 9463442.876 7448962.24 7619228.51 7448564.393

1631NEG PC(26:0/20:4)+HCOO PC(46:4)+HCOO PC (26:0/20:4) (26:0) (20:4) C55 H101 O10 N1 P1 966.7168605 12.88181994 0.002166237 1.097246165 0.788571677 0.665728525 18657072.78 13250333.45 17660785.13 14493213.08 7918664.508 14021026.59 6298735.991 22062347.89 29208543 4217128.545 5062259.158 27515357.72 20536211.75 20459495.76 20459207.99

1632NEG PC(26:0/20:4)+HCOO PC(46:4)+HCOO PC (26:0/20:4) (26:0) (20:4) C55 H101 O10 N1 P1 966.7168605 12.55934723 0.011836252 0.954709161 0.898965183 0.489141231 7601601.028 6435926.487 5856648.751 6787566.742 3412667.725 6144064.933 2244595.555 8743077.756 11446126.17 933701.7395 854066.6742 10375636.8 8594016.722 8421996.603 8599928.161

1633NEG PC(26:0/20:3)+HCOO PC(46:3)+HCOO PC (26:0/20:3) (26:0) (20:3) C55 H103 O10 N1 P1 968.7325105 13.10827386 0.016737292 0.995228228 0.99101333 0.413610391 6939303.918 2040800.662 7649628.221 5081076.04 1444285.608 5900274.676 1841365.044 7036850.382 8798671.399 589406.4981 895498.1157 9754932.086 7311756.132 7521781.879 7304164.117

1634NEG PS(49:1)-H PS(49:1)-H PS (49:1) (49:1) C55 H105 O10 N1 P1 970.7481605 13.34318085 0.006658213 0.902319548 0.795438144 1.233807999 41965034.72 32362232 38996557.23 43652702.07 15425298.94 36074967.38 9157876.11 42932643.01 60542415.27 3480056.095 4023182.221 67976512.3 45080389.1 45098298.03 44571579.47

1635NEG PC(28:0/18:1)+HCOO PC(46:1)+HCOO PC (28:0/18:1) (28:0) (18:1) C55 H107 O10 N1 P1 972.7638105 13.9611069 0.005004343 0.939964162 0.878706005 1.094199036 35750504.56 25825761.51 34372132.4 36337299.62 12169241.29 29536981.02 7570300.662 37680500.89 50068218.9 3289416.435 3812641.566 61125091.17 38518990.25 38187313.57 38186756.13

1636NEG PC(28:0/18:0)+HCOO PC(46:0)+HCOO PC (28:0/18:0) (28:0) (18:0) C55 H109 O10 N1 P1 974.7794605 14.48783522 0.050434417 0.836581431 0.639198049 0.413021995 4973748.908 3070184.811 5247558.623 5039632.251 1559216.696 3631305.343 1190670.147 4502878.792 5900749.356 515832.9875 520239.3589 7047402.151 4884501.661 5320233.293 4877693.965

1637NEG PC(29:1/18:1)+HCOO PC(47:2)+HCOO PC (29:1/18:1) (29:1) (18:1) C56 H107 O10 N1 P1 984.7638105 13.60282208 0.016586985 0.909064268 0.841543997 0.30641378 2744287.883 1616033.753 2715387.904 2198720.374 640871.2267 2194765.301 421832.3739 2037245.573 3080630.102 103827.0563 148380.7467 5216912.832 2588994.586 2588881 2515264.5

1638NEG PC(26:1/22:6)+HCOO PC(48:7)+HCOO PC (26:1/22:6) (26:1) (22:6) C57 H99 O10 N1 P1 988.7012105 12.10264965 0.004420072 1.214767648 0.576533644 0.434875614 8242592.192 6887553.948 7826718.455 6344225.793 4369316.827 5765728.148 2878468.519 9826683.194 15052438.63 2091172.678 3878437.275 14178541.12 8924317.984 8993000.734 8992981.627

1639NEG PC(26:0/22:6)+HCOO PC(48:6)+HCOO PC (26:0/22:6) (26:0) (22:6) C57 H101 O10 N1 P1 990.7168605 12.70590136 0.003059016 1.128193143 0.747909008 0.540867763 10687216.92 9074872.835 10191778.39 8851170.426 4793939.204 8108807.496 3907845.876 12653367.98 19234371.64 2231144.178 2276988.296 18032650.81 12309359.4 12244780.29 12243966.16

163POS Cer(d18:2/24:1)+H Cer(d42:3)+H Cer (d18:2/24:1) (d18:2) (24:1) C42 H80 O3 N1 646.6132705 12.01472645 0.007174697 1.091050359 0.633830145 0.163548002 37812706.67 40709897.01 34451314.82 39881497.7 61771108.87 43363219.32 19797258.83 67436013.37 56316592.75 35548141.91 47513890.91 54867905.53 62657321.92 62666067.71 63443590.09

1640NEG PC(28:1/20:4)+HCOO PC(48:5)+HCOO PC (28:1/20:4) (28:1) (20:4) C57 H103 O10 N1 P1 992.7325105 12.8626009 0.007514067 0.908376873 0.810027684 0.657794499 18722331.42 8680429.355 11205033.42 9511658.355 4432833.987 9118165.018 3435280.337 12079069.39 18598778.44 1677647.326 1812294.282 18416942.17 12955083.9 12787902.8 12787951.45

1641NEG PC(28:0/20:4)+HCOO PC(48:4)+HCOO PC (28:0/20:4) (28:0) (20:4) C57 H105 O10 N1 P1 994.7481605 13.30149243 0.00730037 1.298329698 0.612146005 0.319175998 3485259.461 2489667.819 2978781.995 2612417.571 1270575.435 2434418.135 870744.5075 3693072.37 4872254.16 507522.607 541507.1856 9341848.334 3555498.748 3555533.604 3510746.764

1642NEG PS(51:2)-H PS(51:2)-H PS (51:2) (51:2) C57 H107 O10 N1 P1 996.7638105 13.418 0.004215043 0.993735871 0.987548449 0.280827587 2989398.034 1743218.12 2868507.865 2368733.143 789538.2911 2279844.571 681077.1763 2433792.446 3691589.774 203845.1789 893026.1601 5054229.811 2824066.795 2823973.061 2844687.299

1643NEG PC(30:1/18:1)+HCOO PC(48:2)+HCOO PC (30:1/18:1) (30:1) (18:1) C57 H109 O10 N1 P1 998.7794605 13.8842468 0.023258626 0.829806614 0.664310639 0.460023814 5590913.27 3554453.631 5462875.066 5201496.052 1340169.398 4874902.345 1198640.47 4135198.402 5805062.207 341737.9632 446392.9655 9668527.265 5490202.005 5273329.747 5493028.374

1644NEG PC(30:0/18:1)+HCOO PC(48:1)+HCOO PC (30:0/18:1) (30:0) (18:1) C57 H111 O10 N1 P1 1000.795111 14.50445825 0.007775422 0.9149418 0.81951099 0.358020584 3553399.116 2794562.399 3884005.073 4257141.593 1457524.753 3256678.33 892381.8869 3782538.433 5625530.602 435912.8959 521801.4175 6311746.94 4028940.819 4028960.908 3974933.756

1645NEG SQDG(46:16)+HCOO SQDG(46:16)+HCOO SQDG (46:16) (46:16) C56 H75 O14 S1 1003.488306 7.088790368 0.00502712 1.552167539 0.248002922 0.173344755 238374.7874 610282.1331 227308.403 598958.6293 1897482.647 594602.2101 198492.8625 1428724.678 1257373.659 1181251.944 1377157.719 1024894.949 1099495.385 1099431.04 1089917.804

1646NEG PS(53:5)-H PS(53:5)-H PS (53:5) (53:5) C59 H105 O10 N1 P1 1018.748161 12.27038191 0.012095372 1.182508694 0.376678016 1.925096315 428097183.7 376739058 458823548.7 339211723.8 361492658.5 484444585.3 172776107.6 644715957.1 565272152.4 347174306.3 511442256.5 654356865.9 551500798.4 540011160.5 551468188.7

1647NEG PIP2(37:2p)-H PIP2(37:2p)-H PIP2 (37:2p) (37:2p) C46 H86 O18 N0 P3 1019.503258 8.944 0.022300639 1.140256158 0.525094142 0.263084706 2739312.363 3516829.119 4492835.858 5799966.551 3820866.553 4241384.459 6588385.411 4281826.373 2859044.505 5635786.653 6507388.741 2190634.855 3121744.477 3243806.44 3121569.919

1648NEG PIP2(37:2p)-H PIP2(37:2p)-H PIP2 (37:2p) (37:2p) C46 H86 O18 N0 P3 1019.503258 8.685958762 0.013210266 1.129111946 0.436355266 0.401295345 8212488.049 12473684.5 10241912.24 12417355.4 12522355.87 9056744.733 15509280.26 10703697.02 10332657.25 15411301.04 15091416.56 6258722.489 8538539.448 8735852.507 8539407.295

1649NEG PS(53:1)-H PS(53:1)-H PS (53:1) (53:1) C59 H113 O10 N1 P1 1026.810761 14.48 0.008685684 0.988072781 0.980742309 0.173300221 962466.7146 439920.2011 865138.244 928501.09 283352.2306 778051.1123 67033.95759 808010.0013 1331520.058 62397.23543 33200.92755 1904488.119 946084.1554 960359.7084 946026.9655

164POS SM(d30:1)+H SM(d30:1)+H SM (d30:1) (d30:1) C35 H72 O6 N2 P1 647.5122525 7.21783974 0.011813291 1.040900239 0.849839317 0.088500953 7801826.15 4984688.078 7493549.079 6514602.659 8381881.998 7760079.01 2942769.794 8465111.803 10353454.44 5267203.789 5500747.078 12163458.37 10518507.24 10520826.09 10736380.66

1650NEG PIP2(42:9)-H PIP2(42:9)-H PIP2 (42:9) (42:9) C51 H82 O19 N0 P3 1091.466873 7.969874683 0.020192517 1.329694326 0.402475239 0.216502479 603778.8823 574330.5094 802781.0748 1039478.154 2506726.394 1342207.51 590743.1334 1009727.617 1018660.859 1979000.101 2619522.657 1916418.221 1590896.806 1652614.249 1641413.351

1651NEG GM3(d32:1)-H GM3(d32:1)-H GM3 (d32:1) (d32:1) C55 H99 O21 N2 1123.674587 7.124484475 0.011443719 0.887364715 0.675926779 0.232526752 4199689.585 2070645.799 7285737.673 6884471.271 3814407.214 5068638.856 2758391.937 4028346.132 3889245.007 3900680.113 2338686.419 9105369.83 4979124.858 4979248.28 5078535.882

1652NEG GM3(d34:2)-H GM3(d34:2)-H GM3 (d34:2) (d34:2) C57 H101 O21 N2 1149.690237 7.2963755 0.010841256 0.871363011 0.652209043 0.241792294 3081696.492 1140902.299 5108621.692 3793521.85 3191356.513 4051613.191 2067034.641 2570978.89 2860188.029 2172205.312 1406588.398 6670675.621 3275109.198 3294000.98 3344410.065

1653NEG GM3(d34:1)-H GM3(d34:1)-H GM3 (d34:1) (d34:1) C57 H103 O21 N2 1151.705887 8.23123457 0.003367049 1.070142098 0.733764827 0.27745772 131726705.7 81775283.86 214985042.6 221295674.1 159761483.2 172382081.1 95705592.72 203790551.4 152865040.2 185037044.2 140282468.6 273119941.8 190900497.6 190886380.5 192008811.8

1654NEG GM3(d34:0)-H GM3(d34:0)-H GM3 (d34:0) (d34:0) C57 H105 O21 N2 1153.721537 8.562176482 0.008778553 0.963560476 0.861399287 0.146933035 7365667.77 5587712.553 15612630.66 17562449.34 11194712.11 8851523.254 6188419.338 13159839.17 10050978.24 12028224.01 8877412.697 13458447.84 10733101.03 10571816.59 10735288.78

1655NEG GM3(d35:1)-H GM3(d35:1)-H GM3 (d35:1) (d35:1) C58 H105 O21 N2 1165.721537 8.723243942 0.023034284 1.216514481 0.233019081 0.266517463 4110588.287 3354213.808 6815282.338 7537829.257 4432868.964 4130568.784 4342671.852 6924197.494 5760408.032 7908448.904 5326753.905 6696873.787 5566499.361 5564916.881 5346574.088

1656NEG GM3(d34:1+O)-H GM3(d34:1+O)-H GM3 (d34:1+O) (d34:1+O) C57 H103 O22 N2 1167.700802 7.990250328 0.00293337 1.39114294 0.204852033 0.569980047 4126325.013 2750345.814 7402301.762 9166221.891 9170824.856 7521628.688 3533807.421 9513783.251 5879667.336 13735425.6 10895286.36 12279235.71 8980357.089 8934895.451 8934872.968

1657NEG GM3(d40:1)-H GM3(d40:1)-H GM3 (d40:1) (d40:1) C63 H115 O21 N2 1235.799787 10.64477512 0.001366578 1.050926249 0.728480665 0.238239786 52575010.63 38290681.28 81737520.57 78810495.67 53542977.12 74917148.89 57298423.47 74930381.29 54862515.8 64790099.71 57882024.79 89455938.43 63459826.29 63459458.99 63309553.5

1658NEG GM3(d41:1)-H GM3(d41:1)-H GM3 (d41:1) (d41:1) C64 H117 O21 N2 1249.815437 10.95449585 0.006706712 1.181995105 0.296244435 0.571674233 20231607.28 15264830.08 35245849.06 38348299.93 27481775.31 32637282.61 23966446.38 37014915.62 26705354.58 38852660.91 31822437.01 41643156.79 31307802.28 30917398.7 31241718.56

1659NEG GM3(d42:3)-H GM3(d42:3)-H GM3 (d42:3) (d42:3) C65 H115 O21 N2 1259.799787 10.04196797 0.002886241 1.063617507 0.646133989 0.268468245 8621433.703 8079726.044 14629554.66 14561491.9 12819593.68 13629802.97 10745131.78 13771698.41 8921658.763 14168945.87 12914496.37 16421864.17 12911391.68 12910972.39 12846745.93

165POS PC(26:1)+H PC(26:1)+H PC (26:1) (26:1) C34 H67 O8 N1 P1 648.4598835 6.220603415 0.019057124 0.635316219 0.389530224 0.14002387 2643864.07 806245.4448 2964122.799 1887314.563 562696.3122 1831744.034 290483.5361 851557.4394 1495650.619 185045.2566 11125.61672 3961471.689 2130801.484 2203199.456 2133350.093

1660NEG GM3(d42:2)-H GM3(d42:2)-H GM3 (d42:2) (d42:2) C65 H117 O21 N2 1261.815437 10.61076291 0.004077318 1.076295208 0.614474185 0.55142461 74071775.25 59183342.51 121601968.5 130073877.7 107914120.7 121631856.7 83172416.54 124386971.5 93667844.28 117627430.2 98146476.75 144357448 106500393.7 106499357.8 105749527.5

1661NEG GM3(d42:1)-H GM3(d42:1)-H GM3 (d42:1) (d42:1) C65 H119 O21 N2 1263.831087 11.23877947 0.009046694 1.117265987 0.488008351 0.738517712 208613825.9 154029746.1 328328884.8 388674043.2 255121758.7 339316145.5 244919413.2 372921543.6 268555055.5 270869338.4 297631654.9 415500558.7 309109422 309096702.2 313971919.2

1662NEG GM3(d43:2)-H GM3(d43:2)-H GM3 (d43:2) (d43:2) C66 H119 O21 N2 1275.831087 10.90962808 0.016591756 1.086096804 0.585501043 0.188029812 8517858.736 6251748.409 13612940.1 13981614.76 10485105.26 12763012.95 8545235.584 15188739.65 9522383.65 11666534.03 11591840.58 14746554.33 11730542.8 11732733.35 11397701.28

1663NEG GM3(d43:1)-H GM3(d43:1)-H GM3 (d43:1) (d43:1) C66 H121 O21 N2 1277.846737 11.50034974 0.009997416 1.126269864 0.45305443 0.300974185 16417598.14 11903211.48 28230204.64 31383955.45 22998222.71 25161118.7 19465802.93 28816591.55 21788947.98 25342652.91 23891947.54 33972978.3 27432373.52 27425955.47 26956958.49

1664NEG GM3(d44:2)-H GM3(d44:2)-H GM3 (d44:2) (d44:2) C67 H121 O21 N2 1289.846737 11.19575563 0.005474653 1.04605963 0.808798263 0.083544847 18705095.76 15373287.96 35498752.38 39802126.58 27453471.29 30853908.46 22311059.49 37360210.8 25555438.67 23806424.35 24045371.82 42331722.04 30653193.77 30360301.14 30646738.54

1665NEG GM3(d44:1)-H GM3(d44:1)-H GM3 (d44:1) (d44:1) C67 H123 O21 N2 1291.862387 11.75216573 0.00774444 0.990550545 0.962346488 0.239579289 32331146.23 23443873.7 53607153.76 61851916.55 32003011.98 49637843.52 26303939.57 55780615.69 39834390.41 35760435.41 32480065.69 60325968.46 43723932.07 43136876.89 43717465.42

1666NEG CL(16:1/16:1/16:1/16:1)-H CL(64:4)-H CL (16:1/16:1/16:1/16:1) (16:1) (16:1) (16:1) (16:1) C73 H133 O17 P2 1343.902355 12.99556369 0.005238824 0.953254266 0.880074881 0.131920479 3825617.164 3399095.52 5983800.507 8290205.859 6205198.399 10637014.2 1613145.038 6148470.546 4185848.094 5865799.309 5549206.59 13186187.08 7533454.278 7465648.618 7465367.111

1667NEG CL(14:0/16:1/16:1/18:1)-H CL(64:3)-H CL (14:0/16:1/16:1/18:1) (14:0) (16:1) (16:1) (18:1) C73 H135 O17 P2 1345.918005 13.37886999 0.01287755 0.977047449 0.934617108 0.132690957 3031855.164 1813133.724 4961317.006 4415638.392 3228136.756 5018087.19 1538696.207 4442624.631 2834056.796 2704827.09 2796632.998 7635628.723 4606874.045 4608742.803 4505804.461

1668NEG CL(17:1/16:1/16:1/16:1)-H CL(65:4)-H CL (17:1/16:1/16:1/16:1) (17:1) (16:1) (16:1) (16:1) C74 H135 O17 P2 1357.918005 13.18916161 0.011049915 1.083954299 0.750704403 0.027672577 1843893.443 1798385.951 2902070.53 3681296.114 3387441.105 4163428.951 888740.7939 3934349.874 2823915.313 2969075.175 2905437.311 5747412.57 4056135.003 4058068.257 3979962.99

1669NEG CL(17:1/14:0/18:1/16:1)-H CL(65:3)-H CL (17:1/14:0/18:1/16:1) (17:1) (14:0) (18:1) (16:1) C74 H137 O17 P2 1359.933655 13.651 0.01275624 1.076092619 0.795972249 0.078172414 1019398.447 798078.2856 2110196.401 1758413.284 1107215.995 1742211.722 588693.3793 2145849.192 1365012.557 1146377.795 1015908.93 2923161.906 1856187.333 1855845.235 1815309.491

166POS PC(27:0p)+H PC(27:0p)+H PC (27:0p) (27:0p) C35 H71 O7 N1 P1 648.4962685 7.402628383 0.010200293 1.411383665 0.160479541 0.154703363 1550704.047 1497119.789 1542010.162 2731982.716 2901541.493 1933796.631 643544.3098 4092979.67 3474282.346 2616631.452 3176361.851 3154610.124 3992451.563 4073656.61 4022003.162

1670NEG CL(16:1/16:1/16:1/18:2)-H CL(66:5)-H CL (16:1/16:1/16:1/18:2) (16:1) (16:1) (16:1) (18:2) C75 H135 O17 P2 1369.918005 13.05017147 0.004682149 1.054533126 0.839467602 0.062935512 7150230.376 6963370.148 11647780.73 16550931.43 11645531.72 17252760.96 3512344.147 13266106.89 10132500.66 12503954.03 12870682.57 22808353.97 15075706.85 14954884.42 14953339.27

1671NEG CL(16:1/16:1/16:1/18:1)-H CL(66:4)-H CL (16:1/16:1/16:1/18:1) (16:1) (16:1) (16:1) (18:1) C75 H137 O17 P2 1371.933655 13.40821461 0.005082357 1.056076681 0.835522176 0.055449961 16865165.66 14149624.03 25252214.06 35524469.35 28588518.99 36443536.98 8727863.91 33624550.59 20608225.61 25254157.12 26558393.98 50844480.82 33010791.97 32727221.24 32716849.8

1672NEG CL(18:1/16:1/18:1/14:0)-H CL(66:3)-H CL (18:1/16:1/18:1/14:0) (18:1) (16:1) (18:1) (14:0) C75 H139 O17 P2 1373.949305 13.81610956 0.013494935 1.0943174 0.710839175 0.076668304 5110028.753 2565515.374 7437097.511 5907374.502 4031808.049 7086764.238 3355880.57 6372131.705 4473887.549 4878629.952 5178814.437 10910472.33 7418512.506 7247786.316 7247760.479

1673NEG CL(18:1/16:0/16:1/16:0)-H CL(66:2)-H CL (18:1/16:0/16:1/16:0) (18:1) (16:0) (16:1) (16:0) C75 H141 O17 P2 1375.964955 14.314 0.003600242 1.380166068 0.329949403 0.082124674 826801.9936 46657.9991 1234584.874 738078.347 243586.2413 984026.3857 1050103.482 961671.6987 598353.5043 653845.1949 648255.0617 1710203.036 944350.4879 939565.1911 937807.3356

1674NEG CL(17:1/16:1/18:2/16:1)-H CL(67:5)-H CL (17:1/16:1/18:2/16:1) (17:1) (16:1) (18:2) (16:1) C76 H137 O17 P2 1383.933655 13.2550074 0.009664962 1.211931108 0.321830504 0.274810942 3514056.074 3748501.981 5166046.923 5966581.059 5470159.552 5953513.569 2096659.932 7978470.357 5804843.395 5804157.451 6100239.629 8354032.245 7089133.64 7089268.17 6971184.82

1675NEG CL(17:1/16:1/16:1/18:1)-H CL(67:4)-H CL (17:1/16:1/16:1/18:1) (17:1) (16:1) (16:1) (18:1) C76 H139 O17 P2 1385.949305 13.60680039 0.007094219 1.134220899 0.597877216 0.208123833 5854711.029 6377477.184 10257895.98 13959352.67 11081563.27 12176424.94 3685299.114 15875853.12 8728513.611 10895151.18 10725296.79 17811295.53 13548361.94 13382624.72 13548483.06

1676NEG CL(17:1/16:0/16:1/18:1)-H CL(67:3)-H CL (17:1/16:0/16:1/18:1) (17:1) (16:0) (16:1) (18:1) C76 H141 O17 P2 1387.964955 14.03114578 0.02094945 1.167937844 0.49184425 0.088566584 1256903.167 860600.869 2392383.713 1894472.747 1604165.692 2274017.205 1108215.729 2498677.08 1794935.31 1747995.276 1533546.89 3326001.275 2305375.8 2226732.515 2220965.585

1677NEG CL(18:4/16:1/16:1/18:2)-H CL(68:8)-H CL (18:4/16:1/16:1/18:2) (18:4) (16:1) (16:1) (18:2) C77 H133 O17 P2 1391.902355 13.084 0.01092214 1.003059066 0.989425539 0.097869491 1663760.933 1718837.325 2422227.866 3681337.473 2910541.985 3238820.563 734675.388 2845000.946 1878949.916 2775560.759 3307416.303 4141752.935 2876604.243 2931043.167 2875971.497

1678NEG CL(20:4/16:1/16:1/16:1)-H CL(68:7)-H CL (20:4/16:1/16:1/16:1) (20:4) (16:1) (16:1) (16:1) C77 H135 O17 P2 1393.918005 13.36146962 0.018068247 0.982793525 0.932675208 0.147679192 4198391.78 3603318.065 5799475.039 7607475.738 6832091.628 7887329.412 2317730.457 6734504.418 4539273.538 6398076.594 6120000.782 9200300.24 7308449.503 7085504.099 7083321.56

1679NEG CL(18:2/16:1/16:1/18:2)-H CL(68:6)-H CL (18:2/16:1/16:1/18:2) (18:2) (16:1) (16:1) (18:2) C77 H137 O17 P2 1395.933655 13.73442449 0.020197167 1.045471938 0.808260745 0.037983585 1321816.615 839992.8146 2002917.515 1630260.167 1568777.675 1952689.125 777530.9896 1867535.997 1369790.131 1629938.682 1674196.444 2421098.883 2004537.524 1936606.943 1935431.598

167POS Cer(d18:1/24:1)+H Cer(d42:2)+H Cer (d18:1/24:1) (d18:1) (24:1) C42 H82 O3 N1 648.6289205 12.53584423 0.016999807 1.157852291 0.345081578 0.658253121 193857020.1 171074623.7 157832007.9 175108175.8 240979539 185133981.4 108133537.7 263488174.8 243232595.8 164410222.1 237062136.1 285082343.9 275269406.4 283415855.5 275194035.7

1680NEG CL(18:2/16:1/16:1/18:2)-H CL(68:6)-H CL (18:2/16:1/16:1/18:2) (18:2) (16:1) (16:1) (18:2) C77 H137 O17 P2 1395.933655 13.12094628 0.003654336 1.127861648 0.548444119 0.208519657 9447307.854 10740584.57 14401439.24 15587836.95 15806216.99 17945925.17 5252442.826 19211663.83 14864586.26 15443042.94 15603673.17 24285241.77 19337765.44 19216778.84 19340055.79

1681NEG CL(18:2/16:1/16:1/18:1)-H CL(68:5)-H CL (18:2/16:1/16:1/18:1) (18:2) (16:1) (16:1) (18:1) C77 H139 O17 P2 1397.949305 13.44521711 0.02041999 1.150868822 0.534107916 0.495025264 26582959.1 30100592.67 45427701.68 59296072.27 48075896.86 56323041.32 16933496.34 69977049.45 43163892.15 48420159.95 49218169.05 78195374.94 62348219.94 60213995.03 60174256.26

1682NEG CL(18:1/16:1/16:1/18:1)-H CL(68:4)-H CL (18:1/16:1/16:1/18:1) (18:1) (16:1) (16:1) (18:1) C77 H141 O17 P2 1399.964955 13.82631389 0.002502681 1.153145679 0.559881277 0.474921308 29198163.73 25506409.92 48880199.21 59250491.59 46668002.29 59269501.01 18197234.85 66484513.26 38221360.29 49995306.07 49326194.45 87709546.84 59342811.88 59087573.12 59085076.29

1683NEG CL(18:1/16:0/16:1/18:1)-H CL(68:3)-H CL (18:1/16:0/16:1/18:1) (18:1) (16:0) (16:1) (18:1) C77 H143 O17 P2 1401.980605 14.25571567 0.054660934 1.160177264 0.483260567 0.252173046 4039522.656 2965271.609 6438143.342 6731739.143 5427005.184 7957601.818 3634240.282 8548132.943 3548420.323 7027639.302 7079697.956 9096587.183 7261859.958 7275719.949 7979287.229

1684NEG CL(17:1/16:1/20:4/16:1)-H CL(69:7)-H CL (17:1/16:1/20:4/16:1) (17:1) (16:1) (20:4) (16:1) C78 H137 O17 P2 1407.933655 13.61216988 0.01337968 1.113717171 0.618430046 0.096262492 1532210.833 1606322.573 2617861.298 3321819.511 2646833.908 3063374.58 988993.8669 3391525.934 2268364.802 2617764.659 2911757.799 4291713.236 3217719.77 3226024.409 3296770.739

1685NEG CL(17:1/18:2/18:2/16:1)-H CL(69:6)-H CL (17:1/18:2/18:2/16:1) (17:1) (18:2) (18:2) (16:1) C78 H139 O17 P2 1409.949305 13.31450172 0.024313079 1.224758806 0.293903714 0.27638271 2760964.762 3387934.063 4300083.067 4854435.456 4769246.317 4410512.433 1697935.08 7319222.488 5149097.563 4876662.105 5054316.301 5888751.997 6354360.201 6092532.955 6095646.089

1686NEG CL(17:1/16:1/18:2/18:1)-H CL(69:5)-H CL (17:1/16:1/18:2/18:1) (17:1) (16:1) (18:2) (18:1) C78 H141 O17 P2 1411.964955 13.6603494 0.004850092 1.228052809 0.332557135 0.45221604 8948985.622 10520694.42 14276504.31 16033618.48 13544668.97 14531581.77 5429012.055 24219630.56 15171835.72 14568959.32 14476857.02 21745050.58 18894546.92 18736905.67 18736505.58

1687NEG CL(17:1/18:1/16:1/18:1)-H CL(69:4)-H CL (17:1/18:1/16:1/18:1) (17:1) (18:1) (16:1) (18:1) C78 H143 O17 P2 1413.980605 14.04430811 0.009044076 1.216773198 0.359651476 0.41420908 7923672.203 7731373.823 13518248.55 15314741.6 11713552.38 14305300.62 5650775.115 19557639.61 11779226.1 13819120.67 13709225.76 21274905.79 16321630.61 16321917.79 16067425.12

1688NEG CL(20:5/16:1/18:2/16:1)-H CL(70:9)-H CL (20:5/16:1/18:2/16:1) (20:5) (16:1) (18:2) (16:1) C79 H135 O17 P2 1417.918005 13.12941464 0.020978044 1.015395649 0.933514915 0.119748075 1831317.612 2735392.357 3542939.149 3822501.019 3441907.079 3639172.179 1315045.32 2756354.936 2986324.058 3745640.491 3696629.738 4805955.849 3643048.865 3646553.732 3778824.165

1689NEG CL(18:2/20:4/16:1/16:1)-H CL(70:8)-H CL (18:2/20:4/16:1/16:1) (18:2) (20:4) (16:1) (16:1) C79 H137 O17 P2 1419.933655 13.0346574 0.007774133 1.119437909 0.600186854 0.141747939 4696159.087 4649483.907 7083765.292 7811233.712 3530908.047 7492482.704 2268499.572 7859683.393 6463770.219 6159841.397 6427793.06 10296307.44 8358296.65 8443715.847 8315930.834

168POS PC(26:0)+H PC(26:0)+H PC (26:0) (26:0) C34 H69 O8 N1 P1 650.4755335 7.380614542 0.012164649 0.916646807 0.842918474 0.43093534 33783751.04 11299340.5 34495740.76 24059292.7 6555098.7 26729426.04 9633081.787 20610153.05 38110456.86 3067224.49 3202242.677 50886550.87 34840307.98 35583929.5 34848903.96

1690NEG CL(18:4/18:2/18:1/16:1)-H CL(70:8)-H CL (18:4/18:2/18:1/16:1) (18:4) (18:2) (18:1) (16:1) C79 H137 O17 P2 1419.933655 13.47301902 0.003723884 1.108972815 0.581876024 0.273397538 6517965.263 7517668.414 10665707.73 13245341.64 11705244.12 13104454 4901211.251 14833552.94 9233833.765 12163088.11 12695884.99 15767549.64 12936214.42 12935638.29 13019540.79

1691NEG CL(20:4/16:1/16:1/18:1)-H CL(70:7)-H CL (20:4/16:1/16:1/18:1) (20:4) (16:1) (16:1) (18:1) C79 H139 O17 P2 1421.949305 13.82434482 0.002645491 1.138871278 0.520844129 0.272285884 7726809.548 6939256.73 11594786.48 14142301.45 11703670.45 14541806.89 5213723.04 15564456.06 10011492.26 13056133.42 13107178.35 18951229.08 14401358.99 14335365.13 14335779.07

1692NEG CL(18:2/18:2/18:2/16:1)-H CL(70:7)-H CL (18:2/18:2/18:2/16:1) (18:2) (18:2) (18:2) (16:1) C79 H139 O17 P2 1421.949305 13.21795762 0.061740569 1.224924592 0.244615265 0.339482975 4401448.039 5509454.797 6755968.863 6437860.317 6626503.81 6646236.261 2740867.716 9842880.728 7674699.676 7162551.919 7668957.222 9469702.875 13799931.88 12420357.35 12424459.27

1693NEG CL(18:2/18:1/18:2/16:1)-H CL(70:6)-H CL (18:2/18:1/18:2/16:1) (18:2) (18:1) (18:2) (16:1) C79 H141 O17 P2 1423.964955 14.24872995 0.009889919 1.117046392 0.622367007 0.13163702 2296131.124 1759232.238 2938483.757 3265743.446 3546274.942 2412491.514 1379346.142 4855850.204 1645997.338 2924506.623 3090304.571 4220652.31 3104230.569 3101635.273 3156343.374

1694NEG CL(18:2/16:1/18:2/18:1)-H CL(70:6)-H CL (18:2/16:1/18:2/18:1) (18:2) (16:1) (18:2) (18:1) C79 H141 O17 P2 1423.964955 13.52827984 0.008447587 1.246860575 0.257286583 0.891560371 27200842.79 29606952.32 41846875.7 43286304.84 40010221.32 42505666.65 17451387.05 65528021.25 45491984.33 43386191.71 44218696.76 63790133 53551770.88 53551521.89 52771900.45

1695NEG CL(18:2/18:1/16:1/18:1)-H CL(70:5)-H CL (18:2/18:1/16:1/18:1) (18:2) (18:1) (16:1) (18:1) C79 H143 O17 P2 1425.980605 13.87576778 0.008993922 1.217640002 0.310278202 0.97878011 43937430.68 43536460.83 72657866.52 74957960.78 61012334.94 72871883.17 34040747.47 103565275.3 66850289.44 67790088.92 70340525.04 106690499.3 89921129.9 89997704.1 88566844.66

1696NEG CL(18:1/16:1/18:1/18:1)-H CL(70:4)-H CL (18:1/16:1/18:1/18:1) (18:1) (16:1) (18:1) (18:1) C79 H145 O17 P2 1427.996255 14.25886627 0.001588547 1.397336271 0.154352851 1.072840862 20054254.79 16508434.32 32842707.31 34330062.67 26799508.99 34945360.53 16578394.69 46880707.5 26682302.78 40194738.21 39698938.64 61196583.51 41275467.52 41162158.09 41162058.58

1697NEG CL(17:1/16:1/20:4/18:2)-H CL(71:8)-H CL (17:1/16:1/20:4/18:2) (17:1) (16:1) (20:4) (18:2) C80 H139 O17 P2 1433.949305 13.67836238 0.010389863 1.207831138 0.290128368 0.232854375 2421167.76 2973365.554 3779948.735 3916437.352 3527061.072 3806295.548 1611269.114 5643107.692 3931288.367 4022692.23 4059667.214 5401051.939 4383611.867 4462700.81 4383077.084

1698NEG CL(17:1/16:1/20:4/18:1)-H CL(71:7)-H CL (17:1/16:1/20:4/18:1) (17:1) (16:1) (20:4) (18:1) C80 H141 O17 P2 1435.964955 14.04121087 0.006730656 1.214706027 0.302837831 0.2358669 2286617.686 2077085.069 3787283.211 3889475.086 3103218.526 3824588.79 1768130.126 4955934.178 3116185.006 3954665.101 3879419.425 5366536.068 4380075.628 4380437.489 4329390.863

1699NEG CL(17:1/18:2/18:2/18:1)-H CL(71:6)-H CL (17:1/18:2/18:2/18:1) (17:1) (18:2) (18:2) (18:1) C80 H143 O17 P2 1437.980605 13.74201335 0.009829285 1.27339858 0.202622168 0.432850323 5394996.202 6415919.413 8224815.363 7900892.234 7067642.244 7089229.173 3580141.771 13457842.18 9468380.258 7979414.493 8329601.265 10786416.31 10441393.07 10269008.31 10449977.46

169POS PC(27:0e)+H PC(27:0e)+H PC (27:0e) (27:0e) C35 H73 O7 N1 P1 650.5119185 8.618169763 0.030942079 1.364217609 0.156711359 0.288673177 8874987.181 9903212.257 8228990.395 11568483.82 10889583.03 12363868.84 4176621.278 13562876.46 18775370.06 13282107.69 13663677.13 20887729.15 16672273.76 16712163.91 15813996.64

16POS WE(22:2)+NH4 WE(22:2)+NH4 WE (22:2) (22:2) H44 C22 O2 N1 354.3366555 2.56809922 0.034515946 0.981393957 0.958350149 0.027673498 642687.2358 3039898.979 1529071.984 4332949.304 1970832.116 754100.4548 210404.7274 3111165.509 1793895.835 2198921.215 2021584.861 2705280.332 3247660.512 3064446.108 3057625.687

1700NEG CL(17:1/18:1/18:2/18:1)-H CL(71:5)-H CL (17:1/18:1/18:2/18:1) (17:1) (18:1) (18:2) (18:1) C80 H145 O17 P2 1439.996255 14.09153455 0.009647772 1.281320916 0.210306549 0.508303525 7047459.489 7330146.741 11513294.61 11955518.05 8077005.422 9719534.978 5446942.619 17784644.61 10689645.9 10776922.29 11242779.14 15355552.99 13457545.52 13458800.01 13234529.27

1701NEG CL(17:1/18:1/18:1/18:1)-H CL(71:4)-H CL (17:1/18:1/18:1/18:1) (17:1) (18:1) (18:1) (18:1) C80 H147 O17 P2 1442.011905 14.48373301 0.007202789 1.419016113 0.076210055 0.498225965 3147945.934 2825765.253 5321213.981 4999299.575 4080233.254 4933541.638 2721675.696 8359150.478 5112905.219 6686188.326 6055907.375 6976632.167 6362268.638 6283608.384 6283492.229

1702NEG CL(22:6/16:1/16:1/18:2)-H CL(72:10)-H CL (22:6/16:1/16:1/18:2) (22:6) (16:1) (16:1) (18:2) C81 H137 O17 P2 1443.933655 13.22298083 0.014047027 1.162781591 0.35578756 0.105149476 1631173.567 2110628.941 2736961.07 2446987.541 2343509.084 2247292.892 1201784.339 2391288.337 3794828.009 2839285.602 2528397.993 2961214.826 2985656.374 2914126.484 2914222.236

1703NEG CL(20:5/16:1/18:1/18:2)-H CL(72:9)-H CL (20:5/16:1/18:1/18:2) (20:5) (16:1) (18:1) (18:2) C81 H139 O17 P2 1445.949305 13.53544712 0.003822703 1.205825709 0.265762395 0.384204744 5332728.841 7923452.465 10279869.49 10824067.01 9757586.211 9727475.628 4905757.091 13972634.07 10443766.46 10884220.21 11100633.3 13620890.79 11959697.12 11939165.22 12026700.79

1704NEG CL(18:2/16:1/18:2/20:4)-H CL(72:9)-H CL (18:2/16:1/18:2/20:4) (18:2) (16:1) (18:2) (20:4) C81 H139 O17 P2 1445.949305 13.09943832 0.037803806 1.044153468 0.785246177 0.042930387 8264293.967 6691386.041 8865579.326 8799043.35 7179145.255 8625364.878 3507655.476 11507266.18 8326687.387 7630548.912 8015061.135 11575717.13 9051263.212 8474588.821 9058303.93

1705NEG CL(20:4/16:1/18:1/18:2)-H CL(72:8)-H CL (20:4/16:1/18:1/18:2) (20:4) (16:1) (18:1) (18:2) C81 H141 O17 P2 1447.964955 13.88012959 0.005039591 1.225375123 0.215402384 0.567360954 11748221.6 11502507.93 17562785.93 18458707.43 14943286.45 18069622.1 8779131.594 23613861.03 19862256.6 18043537.1 18438505.43 24346612.51 20921137.07 20737954.09 20918905.66

1706NEG CL(18:2/20:4/16:1/18:1)-H CL(72:8)-H CL (18:2/20:4/16:1/18:1) (18:2) (20:4) (16:1) (18:1) C81 H141 O17 P2 1447.964955 13.4335156 0.00818094 1.07669566 0.861056088 0.221724959 3540151.566 12619389.11 5728391.341 19569778.63 18161917.59 16778063.68 7788250.923 26683855.98 7111079.275 5437688.414 5069676.142 30166512.55 21634170.93 21637008.58 21330468.76

1707NEG CL(18:2/18:2/18:2/18:1)-H CL(72:7)-H CL (18:2/18:2/18:2/18:1) (18:2) (18:2) (18:2) (18:1) C81 H143 O17 P2 1449.980605 13.61269187 0.010318742 1.304684761 0.183382398 0.646599952 11164884.57 15573944.69 18339074.24 17767867.01 14457296.07 16923619.88 9272852.211 31902076.29 21081831.92 17550769.28 17443738.61 25684853.59 23696448.2 23281374.16 23274544.87

1708NEG CL(20:4/18:1/16:1/18:1)-H CL(72:7)-H CL (20:4/18:1/16:1/18:1) (20:4) (18:1) (16:1) (18:1) C81 H143 O17 P2 1449.980605 14.27000836 0.004514571 1.313362383 0.18494108 0.528246051 5812545.847 5120420.899 9699134.914 9901324.517 7784548.851 10268344.63 4620857.197 13992039.91 8035684.336 11547744.95 11060846.85 14554271.31 12024623.9 11931620.05 12026172.55

1709NEG CL(18:2/18:1/18:1/18:2)-H CL(72:6)-H CL (18:2/18:1/18:1/18:2) (18:2) (18:1) (18:1) (18:2) C81 H145 O17 P2 1451.996255 13.93800771 0.006244413 1.275896477 0.202057935 0.904245343 23602583.06 25771565.54 38927907.14 34235580.04 27260112.59 33080231.76 17908436.68 57550195.39 40006817.17 33812551.26 33942558.27 50112811.79 44105503 43629685.94 44104487.56

170POS Cer(d18:1/24:0)+H Cer(d42:1)+H Cer (d18:1/24:0) (d18:1) (24:0) C42 H84 O3 N1 650.6445705 13.11968876 0.004200513 1.176434897 0.449500346 1.304432893 505308012.5 438704015.2 486811607.6 556002065.6 519222292.3 530009020.3 195844521.5 841117709.5 802100020.6 395733755.3 488783512.9 848143900.7 850068035.2 850158371.4 856312745.3

1710NEG CL(18:2/18:1/18:1/18:1)-H CL(72:5)-H CL (18:2/18:1/18:1/18:1) (18:2) (18:1) (18:1) (18:1) C81 H147 O17 P2 1454.011905 14.30904351 0.006135222 1.424662581 0.093298085 1.070886224 16996619.42 15486287.73 27317515.62 24445734.26 20097576.05 24163409.67 14259868.51 43026437.31 25817989.56 28990750.73 27789687.77 43194583.73 31511669.61 31847190.39 31510625.49

1711NEG CL(18:1/18:1/18:1/18:1)-H CL(72:4)-H CL (18:1/18:1/18:1/18:1) (18:1) (18:1) (18:1) (18:1) C81 H149 O17 P2 1456.027555 14.71371161 0.009224529 1.539908632 0.041166868 0.720099148 4846929.303 4068235.393 7839503.336 7491434.554 5885491.28 7350025.493 4503696.115 12364219.69 6867013.023 11292312.85 11439777.1 11251250.4 9478149.177 9327552.155 9329078.32

1712NEG CL(17:1/18:2/20:4/18:2)-H CL(73:9)-H CL (17:1/18:2/20:4/18:2) (17:1) (18:2) (20:4) (18:2) C82 H141 O17 P2 1459.964955 13.73984884 0.006468828 1.192734672 0.334138284 0.15224871 1457584.334 1659864.066 2219621.392 2023801 1937630.883 1901917.524 851989.9511 3289782.678 2460505.784 2047174.419 1990309.449 2719366.039 2691100.902 2691662.976 2661342.917

1713NEG CL(18:1/18:0/18:0/18:1)-H CL(72:2)-H CL (18:1/18:0/18:0/18:1) (18:1) (18:0) (18:0) (18:1) C81 H153 O17 P2 1460.058855 10.669 0.00879135 1.127499741 0.500083829 0.413469468 39896226.64 35780697.81 51815614.26 48616452.46 49238399.07 46444069.23 19122539.46 68551977.65 50621146.65 45899299.85 48904148.99 73345687.64 66929664.26 65921774.57 66941951.16

1714NEG CL(17:1/20:4/18:1/18:2)-H CL(73:8)-H CL (17:1/20:4/18:1/18:2) (17:1) (20:4) (18:1) (18:2) C82 H143 O17 P2 1461.980605 14.10124946 0.005016866 1.250759607 0.194160864 0.245977533 1980059.231 2120389.262 3321460.909 3014994.093 2432264.304 2762666.105 1731341.441 4724261.204 2990394.443 2993201.589 3082842.38 4029625.379 3679993.232 3712159.652 3712153.814

1715NEG CL(17:1/18:1/18:2/20:4)-H CL(73:8)-H CL (17:1/18:1/18:2/20:4) (17:1) (18:1) (18:2) (20:4) C82 H143 O17 P2 1461.980605 13.67425611 0.026331854 1.46559239 0.078673852 0.299966743 1645693.932 2027391.557 1544292.591 2536755.362 1710234.326 2303250.468 1321513.051 4444624.677 3530633.927 2591640.131 2311034.672 3047085.282 3304823.783 3303858.714 3457370.177

1716NEG CL(17:1/18:1/20:3/18:2)-H CL(73:7)-H CL (17:1/18:1/20:3/18:2) (17:1) (18:1) (20:3) (18:2) C82 H145 O17 P2 1463.996255 13.81 0.047811653 1.054606846 0.857569482 0.097646332 1131527.827 1100445.47 2200731.769 1563020.866 1377377.23 1428177.554 512256.2529 2881636.371 1714230.935 1253123.551 488120.442 2432523.347 2143197.074 1975363.165 1974639.108

1717NEG CL(17:1/18:1/20:4/18:1)-H CL(73:7)-H CL (17:1/18:1/20:4/18:1) (17:1) (18:1) (20:4) (18:1) C82 H145 O17 P2 1463.996255 14.49854945 0.002710846 1.390286575 0.145027022 0.258757041 1002717.876 769819.2474 1704437.822 1591611.77 1202822.397 1565763.722 687742.1123 2783438.502 1628271.442 1762250.982 1799717.991 2234495.15 1787293.298 1787266.693 1778901.284

1718NEG CL(17:1/18:1/20:3/18:1)-H CL(73:6)-H CL (17:1/18:1/20:3/18:1) (17:1) (18:1) (20:3) (18:1) C82 H147 O17 P2 1466.011905 14.20013424 0.010097654 1.237044202 0.375740918 0.150142641 1143508.709 1161100.183 1890796.136 1780234.331 1074970.588 1550233.377 854082.7736 2897342.142 959706.2334 1687661.387 1683326.624 2557504.207 2132076.153 2132164.189 2095046.349

1719NEG CL(18:2/18:1/22:6/16:1)-H CL(74:10)-H CL (18:2/18:1/22:6/16:1) (18:2) (18:1) (22:6) (16:1) C83 H141 O17 P2 1471.964955 13.60414792 0.002342225 1.161832811 0.315967713 0.176458663 5061009.056 4042637.682 4904642.86 4463399.66 3891330.809 4609332.006 2566063.334 7031723.628 5662784.017 4597030.022 4810797.445 6668965.177 5710425.482 5687292.161 5710428.985

171POS Cer(d18:1/24:0)+H Cer(d42:1)+H Cer (d18:1/24:0) (d18:1) (24:0) C42 H84 O3 N1 650.6445705 12.50645442 0.022513633 1.140432941 0.608909347 0.231514154 20727811.86 31298245.52 27007008.51 34281864.14 28223476.31 34546417.89 17383240.8 56047742.28 39059322.91 15862826.12 19415139.75 53044662.13 54275326.84 55029811.21 52651387.15

1720NEG CL(18:2/16:1/22:6/18:1)-H CL(74:10)-H CL (18:2/16:1/22:6/18:1) (18:2) (16:1) (22:6) (18:1) C83 H141 O17 P2 1471.964955 13.22941008 0.019430604 1.030393964 0.885318928 0.054899224 3528707.051 3901060.239 6400437.389 2716038.481 1804741.735 4254557.522 2295429.68 5285413.789 3771125.568 3692584.065 3377011.705 4871049.654 5657224.599 5475833.86 5466527.26

1721NEG CL(18:2/20:4/18:2/18:1)-H CL(74:9)-H CL (18:2/20:4/18:2/18:1) (18:2) (20:4) (18:2) (18:1) C83 H143 O17 P2 1473.980605 13.50041929 0.041301875 1.200795218 0.468384428 0.318749527 6522242.564 5402348.102 13970142.06 14425951.46 9100050.384 12374602.26 6173166.777 20617035.33 14458773.45 10393841.27 7139893.542 15420834.59 12493647.21 13426019.98 13438183.17

1722NEG CL(18:2/18:1/20:4/18:2)-H CL(74:9)-H CL (18:2/18:1/20:4/18:2) (18:2) (18:1) (20:4) (18:2) C83 H143 O17 P2 1473.980605 13.94828913 0.007057497 1.256376894 0.166653846 0.432990594 6130621.81 6726978.239 9950210.171 8509973.903 7129786.213 8518589.743 5222867.239 13938545.68 10146557.41 8889629.815 8900512.825 11909085.36 10982889.72 10851255.72 10986996.32

1723NEG CL(18:2/18:1/20:4/18:1)-H CL(74:8)-H CL (18:2/18:1/20:4/18:1) (18:2) (18:1) (20:4) (18:1) C83 H145 O17 P2 1475.996255 14.32528059 0.001989628 1.399432178 0.097288482 0.567065209 5171047.853 4539999.901 7890733.896 7033427.948 5829493.302 7122811.273 4126622.038 12535190.78 7577518.058 8238450.067 8187716.721 11935679.16 9064008.77 9063994.874 9032801.879

1724NEG CL(18:2/18:1/18:1/20:4)-H CL(74:8)-H CL (18:2/18:1/18:1/20:4) (18:2) (18:1) (18:1) (20:4) C83 H145 O17 P2 1475.996255 13.86342122 0.02843881 1.391749821 0.360992342 0.395993408 11107156.43 3257892.883 4855542.623 9044012.012 9455597.036 13940596.15 1921976.299 15344976.73 17908874.23 12143696.33 3736872.055 20842509.54 13442069.9 14115601.78 13442749.65

1725NEG CL(20:4/18:1/18:1/18:1)-H CL(74:7)-H CL (20:4/18:1/18:1/18:1) (20:4) (18:1) (18:1) (18:1) C83 H147 O17 P2 1478.011905 14.73530438 0.013973407 1.532232788 0.043993776 0.390972044 1584580.039 1309476.999 2634882.663 2580347.911 1816536.571 2198391.402 1473587.067 4015521.661 2330899.842 3893713.68 3153113.202 3710285.198 2967553.433 2967125.098 3039739.888

1726NEG CL(18:2/18:1/18:1/20:3)-H CL(74:7)-H CL (18:2/18:1/18:1/20:3) (18:2) (18:1) (18:1) (20:3) C83 H147 O17 P2 1478.011905 14.06257187 0.008289352 1.750516533 0.278593306 0.600577734 3862989.67 4132582.298 6689493.618 5743517.09 4425055.855 6641253.751 2996703.163 25664797.27 7171183.385 4981620.922 5316208.356 9001816.543 7244794.653 7244775.629 7141263.017

1727NEG CL(18:1/18:1/18:1/20:0)-H CL(74:3)-H CL (18:1/18:1/18:1/20:0) (18:1) (18:1) (18:1) (20:0) C83 H155 O17 P2 1486.074505 10.72128248 0.003215863 1.236479279 0.272813824 1.148963748 51002082.21 45207056.37 61131000.45 73966252.07 83410653.2 61981824.06 24647091.61 98099647.75 73414282 81243826.08 86992043.17 101383454.4 97236684.07 96633914.07 96798351.19

1728NEG CL(74:2)-H CL(74:2)-H CL (74:2) (74:2) C83 H157 O17 P2 1488.090155 9.841181917 0.015558655 0.9832401 0.953572838 0.413855739 8868120.613 8997250.395 11844276.2 11029598.05 6987011.331 11760781.52 2213788.11 14173209.21 12134508.25 4236271.394 6475419.89 19256844.45 13991524.45 14374441.25 14376492.8

1729NEG CL(18:1/18:0/18:0/20:0)-H CL(74:1)-H CL (18:1/18:0/18:0/20:0) (18:1) (18:0) (18:0) (20:0) C83 H159 O17 P2 1490.105805 11.318 0.005435221 1.303760403 0.183850691 1.173769113 46898923.98 34741422.56 58464632.6 39639578.1 39991047.11 38047296.81 21057762.18 81228171.27 58972118.43 48415108.11 55735291.38 70678687.62 74239158.77 73547612.97 74249463.87

172POS Cer(d18:0/24:0)+H Cer(d42:0)+H Cer (d18:0/24:0) (d18:0) (24:0) C42 H86 O3 N1 652.6602205 13.32451344 0.039651632 1.232197914 0.485149379 0.39790106 40482472.75 47444736.98 40892535.08 79606738.88 51602279.12 73132728.66 23871474.14 87552000.96 83348286.2 41272784.1 42173359.09 132302990.2 84800911.88 84970820.14 90850443.56

1730NEG CL(21:0/16:0/22:6/16:0)-H CL(75:6)-H CL (21:0/16:0/22:6/16:0) (21:0) (16:0) (22:6) (16:0) C84 H151 O17 P2 1494.043205 9.604 0.005818588 0.894844095 0.593136654 0.363949723 9407726.869 5194979.302 9984220.397 7489272.073 6024900.089 10242619.57 3045046.184 9679197.018 8296707.388 4609917.098 6446963.223 11182259.94 10567947.61 10675063.16 10568447.98

1731NEG CL(18:2/18:1/20:4/20:4)-H CL(76:11)-H CL (18:2/18:1/20:4/20:4) (18:2) (18:1) (20:4) (20:4) C85 H143 O17 P2 1497.980605 13.74540177 0.125318362 1.391679086 0.046519854 0.22361567 1101716.076 1079398.281 1734374.536 1371483.868 1093896.271 1516300.475 1283583.003 2224152.806 1768604.421 2584544.135 1632491.843 1496949.43 2105997.58 2596842.931 2102880.061

1732NEG CL(18:2/18:1/20:3/20:4)-H CL(76:10)-H CL (18:2/18:1/20:3/20:4) (18:2) (18:1) (20:3) (20:4) C85 H145 O17 P2 1499.996255 14.05064935 0.023701058 1.123954949 0.497264442 0.075483026 1147910.822 1197097.057 1819938.154 1636285.852 1095350.753 1413114.359 730508.2454 2223095.579 1822283.199 1465451.949 1277395.964 1820990.128 1742780.272 1815441.552 1743021.011

1733NEG CL(22:3/18:1/18:1/18:1)-H CL(76:6)-H CL (22:3/18:1/18:1/18:1) (22:3) (18:1) (18:1) (18:1) C85 H153 O17 P2 1508.058855 10.72145364 0.01000307 1.069243365 0.583756856 0.301648092 5924189.345 5409040.864 5689157.438 7049230.157 6693721.605 7695300.45 4987890.393 7314316.908 4709418.81 8560063.886 8680100.361 6871993.609 7011119.218 7009339.921 7132383.333

1734NEG CL(20:4/18:0/18:1/20:0)-H CL(76:5)-H CL (20:4/18:0/18:1/20:0) (20:4) (18:0) (18:1) (20:0) C85 H155 O17 P2 1510.074505 10.70803633 0.006893573 1.216256916 0.178074481 0.898534415 36905175.18 34380565.69 44430174.34 41777832.16 39464902.48 38608760.87 23376153.96 61580062.28 51269090.46 45651453.71 44446477.26 60187254.74 60065945.83 59353593.86 60069963.71

1735NEG CL(76:2)-H CL(76:2)-H CL (76:2) (76:2) C85 H161 O17 P2 1516.121455 10.80738039 0.006697111 1.244504264 0.350554211 0.37991362 12787841.08 12563930.16 13852835.47 12855407.81 16740731.22 11895905.08 3330795.045 23318941.68 21450147.8 13701658.37 14041089.47 24584693.66 20932339.69 20664444.17 20865501.24

1736NEG CL(23:0/16:1/18:1/20:4)-H CL(77:6)-H CL (23:0/16:1/18:1/20:4) (23:0) (16:1) (18:1) (20:4) C86 H155 O17 P2 1522.074505 9.887 0.026047829 1.252126612 0.242430639 0.325553718 9719856.885 6801108.545 8394695.2 4902075.181 8305563.216 8173637.45 3532913.573 12789642.39 11311409.34 8222573.347 9689063.178 12424024.41 13184653.71 12741017.95 12530563.82

1737NEG CL(23:0/18:1/18:1/18:1)-H CL(77:3)-H CL (23:0/18:1/18:1/18:1) (23:0) (18:1) (18:1) (18:1) C86 H161 O17 P2 1528.121455 10.7052685 0.004133053 1.161153391 0.409854264 0.238241538 11419392.12 11438179.76 12556500.51 12354320.27 15164912.53 13236406.31 6011883.102 20489958.57 19157617.2 10728663.12 13598336.63 18458260.17 18500639.97 18369217.37 18502042.59

1738NEG CL(23:0/18:0/18:0/18:1)-H CL(77:1)-H CL (23:0/18:0/18:0/18:1) (23:0) (18:0) (18:0) (18:1) C86 H165 O17 P2 1532.152755 11.35401974 0.005862584 1.175373505 0.440773104 0.241614649 8920529.143 6459993.517 7505484.123 7096301.437 7685847.895 6312026.148 3451749.948 12799480.59 12098077.33 5434929.772 7259019.526 10649883.82 11504487.35 11622125.34 11622081.65

1739NEG CL(23:0/18:1/18:2/20:4)-H CL(79:7)-H CL (23:0/18:1/18:2/20:4) (23:0) (18:1) (18:2) (20:4) C88 H157 O17 P2 1548.090155 9.937355278 0.051258978 1.312141602 0.28063829 0.333879072 6775275.365 4970951.088 6569244.804 6804067.041 9341762.43 4996021.424 2026461.291 11151581.57 11864185.64 5825288.798 8282519.256 12623557.36 10135762.56 10114388.71 11051235.97

173POS LPC(26:1)+Na LPC(26:1)+Na LPC (26:1) (26:1) C34 H68 O7 N1 P1 Na1 656.4625635 8.247669475 0.007576918 0.872310596 0.633933883 0.298272906 16180897.94 9077954.872 18204806.45 19033954.31 13480343 21230276.8 7618783.91 19321321.52 19028483.7 5581975.151 5265429.223 27979778.53 22065779.57 22063955.86 21776562.6

1740NEG CL(18:1/20:3/24:1/18:1)-H CL(80:6)-H CL (18:1/20:3/24:1/18:1) (18:1) (20:3) (24:1) (18:1) C89 H161 O17 P2 1564.121455 9.515157837 0.02093719 0.86473203 0.63285617 0.255934148 4149301.637 2020622.37 5878609.611 4524780.593 4895588.951 7811593.189 1259401.581 4889437.954 3874476.562 3953746.223 2369437.83 8973282.91 6842134.969 7125996.543 7048149.319

1741NEG CL(20:4/20:4/24:1/18:1)-H CL(82:10)-H CL (20:4/20:4/24:1/18:1) (20:4) (20:4) (24:1) (18:1) C91 H157 O17 P2 1584.090155 8.657107455 0.018862814 1.078164633 0.76294068 0.078833347 1352971.368 1302092.303 2144424.488 1130210.229 1441540.661 1856423.936 341334.0616 2738110.155 1834018.173 1206579.503 1409113.525 2419784.457 1906207.514 1846967.927 1843676.046

1742NEG GM1(d42:1)-H GM1(d42:1)-H GM1 (d42:1) (d42:1) C79 H142 O31 N3 1628.963286 11.23130005 0.043261805 0.922122774 0.811462306 0.273598495 4189658.631 925699.3274 7798031.573 12566197.47 7474649.045 4670461.009 9756929.114 5532250.587 2498118.067 8654421.823 4074308.601 4178561.822 4794881.8 4787771.816 5159493.089

174POS DG(16:1/22:6)+NH4 DG(38:7)+NH4 DG (16:1/22:6) (16:1) (22:6) C41 H70 O5 N1 656.5248505 10.207 0.009107604 0.796715732 0.42847025 0.142383908 13455482.42 12905522.8 14785598.34 20365010.01 9516572.083 5850338.11 1076811.234 18342072.65 11496691.85 7196937.077 8905671.483 14232145.06 14701652.88 14469802 14474572.25

175POS LPC(26:0)+Na LPC(26:0)+Na LPC (26:0) (26:0) C34 H70 O7 N1 P1 Na1 658.4782135 8.052375027 0.043156197 1.488967498 0.260788638 0.187916911 2939364.181 2520537.316 3163228.273 5005315.215 2771337.509 3371134.434 769293.3376 6157984.004 7450989.568 2661541.063 3255887.638 9142557.097 6749954.174 7267339.045 6749845.814

176POS DG(16:0/22:6)+NH4 DG(38:6)+NH4 DG (16:0/22:6) (16:0) (22:6) C41 H72 O5 N1 658.5405005 10.91666906 0.005194836 1.103640988 0.78772108 0.277193549 69531346.29 87683754.58 68336580.28 78854278.37 75242082.06 35594398.19 15561541.02 189855667.8 94335533.19 52942901.75 55239555.84 50343377.04 101668298 101812712.2 100836393.4

177POS Cer(d43:4)+H Cer(d43:4)+H Cer (d43:4) (d43:4) C43 H80 O3 N1 658.6132705 12.86324693 0.006000722 1.21037014 0.297465493 0.733054236 60636800.2 62622215.87 58970541.44 52183059.99 89262232.03 66213909.21 63971913.42 70379349.75 57806901.86 99484287.85 124839220.9 55428037.55 66605152.47 66603034.71 65914232.76

178POS DG(18:1/20:4)+NH4 DG(38:5)+NH4 DG (18:1/20:4) (18:1) (20:4) C41 H74 O5 N1 660.5561505 11.19875514 0.023427317 0.899144947 0.60016322 0.223529791 81340375.5 66645812.79 95166528.67 75848995 68333708.26 49108894.76 20053123.78 105999381.4 79190516.51 59229054.68 50331773.11 77622850.82 92196199.41 88503890.44 92193778.45

179POS SM(d31:1)+H SM(d31:1)+H SM (d31:1) (d31:1) C36 H74 O6 N2 P1 661.5279025 7.852496852 0.008053765 1.084673645 0.731723087 0.159705986 12383739.3 8235519.058 13826093.11 12355165.16 11541025.44 12506338.98 4611094.609 15745355.72 17518009.49 8497202.426 8351722.575 22123444.56 18642473.89 18907540.05 18902318.87

17POS AcCa(13:0)+H AcCa(13:0)+H AcCa (13:0) (13:0) C20 H40 O4 N1 358.2951855 1.815830042 0.039304147 0.970171297 0.927344501 0.046000215 332278.8006 979554.9037 357849.5436 1309796.995 839314.2745 1310561.359 540213.1235 931245.4353 1086756.691 385383.8292 363272.0239 1669482.74 1192806.141 1193067.182 1276033.29

180POS PE(30:1)+H PE(30:1)+H PE (30:1) (30:1) C35 H69 O8 N1 P1 662.4755335 8.914449997 0.045548395 0.854269901 0.581379356 0.343493613 19136574.97 17009684.04 23574546.68 22709169.51 17137431.56 26917011.61 8263939.802 22966393.37 19603338.71 8082944.458 8970392.068 40164823.21 28079208.43 28091765.38 30361025.61

181POS LPC(28:1)+H LPC(28:1)+H LPC (28:1) (28:1) C36 H73 O7 N1 P1 662.5119185 8.196082552 0.021558788 1.927747286 0.097393767 0.562491699 13585495.26 9213333.282 15160009.82 15473589.98 9377883.42 12240914.05 6250512.517 28682416.66 29500123.03 15209494.25 15475354.87 49561895.57 25062364.09 26017075.88 26027059.3

182POS LPC(28:1)+H LPC(28:1)+H LPC (28:1) (28:1) C36 H73 O7 N1 P1 662.5119185 8.992231708 0.016447961 1.030111649 0.913057636 0.246370452 19447475.61 15746172.62 16286330.74 22998695.28 13637569.64 19597066.32 6503890.203 27898483.5 30193571.45 8162117.006 9991189.304 28207484.17 26792188.85 27574116.58 26814709.87

183POS LPC(28:1)+H LPC(28:1)+H LPC (28:1) (28:1) C36 H73 O7 N1 P1 662.5119185 9.357004463 0.009675367 0.682937668 0.20957936 0.432924322 16918246.38 10878484.02 19078485.75 17960752.8 10593327.96 22571277.78 8022667.065 11984666.86 14161623.4 3512064.701 3824460.977 25422800.91 18223583.11 18530439.69 18534379.23

184POS DG(18:1/20:3)+NH4 DG(38:4)+NH4 DG (18:1/20:3) (18:1) (20:3) C41 H76 O5 N1 662.5718005 11.79676476 0.01748933 1.333563808 0.341684803 0.347909592 19228010.34 20331703.86 19306911.58 21526593.91 18332695.51 14949288.81 4993039.19 42814203.65 26482560.36 17298536.9 16804539.26 43200258.63 28291178.37 29163234.85 28303547.12

185POS Cer(d18:1/25:1)+H Cer(d43:2)+H Cer (d18:1/25:1) (d18:1) (25:1) C43 H84 O3 N1 662.6445705 12.7905912 0.003697036 1.28603858 0.271163426 0.267820985 14995207.07 13245666.11 12335785.6 13697850.62 15263338.77 13114499.85 7201994.095 22109816.56 21493693.84 10998462.69 14945166.11 29544974.95 22170862.03 22313320.49 22313560.79

186POS PC(27:0)+H PC(27:0)+H PC (27:0) (27:0) C35 H71 O8 N1 P1 664.4911835 7.813002045 0.012339306 0.845099159 0.693119203 0.123250225 2322197.812 1179965.88 2227675.523 1707450.072 452856.7164 1659191.586 565277.3139 1216954.538 2714185.721 184024.1417 189180.2398 3200515.216 2242143.296 2291453.074 2244260.205

188POS DG(18:0/20:3)+NH4 DG(38:3)+NH4 DG (18:0/20:3) (18:0) (20:3) C41 H78 O5 N1 664.5874505 12.09592187 0.005218848 1.174591629 0.636847471 0.149633664 14752145.38 30251028.66 15396281.35 28699567.24 11098359.28 10850442.03 7630809.256 26577861.98 46228795.76 19990181.79 11369814.29 18638381.35 20183653.8 20366046.19 20182459.9

189POS Cer(d18:1/25:0)+H Cer(d43:1)+H Cer (d18:1/25:0) (d18:1) (25:0) C43 H86 O3 N1 664.6602205 13.30759231 0.009943914 1.213912923 0.37195999 0.426320874 36072453.03 38563898.78 41250629.17 44177910.9 42015686.6 43788153.61 14700243.89 68986095.43 63109887.59 34239637.61 44378475.03 73048891.7 66893689.85 68058607.75 68059805.4

18POS WE(23:3)+NH4 WE(23:3)+NH4 WE (23:3) (23:3) H44 C23 O2 N1 366.3366555 2.446559613 0.011864284 0.982797956 0.9679468 0.042128759 562042.2072 5434070.496 618337.5724 6319569.379 3268249.909 1249357.222 64234.13786 4856813.336 2570397.833 3508862.838 2763910.321 3387204.673 4396239.039 4395916.353 4306355.326

190POS DG(20:1/18:1)+NH4 DG(38:2)+NH4 DG (20:1/18:1) (20:1) (18:1) C41 H80 O5 N1 666.6031005 12.42727582 0.029779965 1.071830747 0.832923171 0.191728544 29380239.38 38178899.51 27496090.1 42364774.76 35477734.92 25302598.71 7704308.119 78183133.78 50660928.22 21009989.47 17073629.82 37805226.35 49513365.28 49515537.01 52113106.21

191POS Cer(d18:0/25:0)+H Cer(d43:0)+H Cer (d18:0/25:0) (d18:0) (25:0) C43 H88 O3 N1 666.6758705 13.58981592 0.032621804 1.210202668 0.634459448 0.172977646 4187769.434 5872953.988 5570777.621 9899227.551 5585410.62 7816751.542 2181791.34 11120022.2 12094152.83 3753127.619 884573.8171 17083020.45 11444328.24 11467623.63 12115379.4

192POS Cer(d18:1/26:5)+H Cer(d44:6)+H Cer (d18:1/26:5) (d18:1) (26:5) C44 H78 O3 N1 668.5976205 12.005 0.025965961 0.97841262 0.85637015 0.412266589 55252665.17 70140864.93 50434715.03 62138803.94 88212333.48 58043076.97 46371070.97 75365090.29 57736669.65 60463688.33 78621069.5 57370514.54 68587397.14 65605028.08 65580157.79

193POS DG(16:0/22:1)+NH4 DG(38:1)+NH4 DG (16:0/22:1) (16:0) (22:1) C41 H82 O5 N1 668.6187505 12.98644068 0.00281351 1.084200645 0.844219189 0.205538493 11922363.24 11753640.88 12330178.08 14690057.2 9781021.933 9220900.107 279286.1323 30002357.62 20519995.34 1939995.164 6279669.682 16545487.63 19529361.88 19551239.71 19633774.41

194POS ChE(18:1)+NH4 ChE(18:1)+NH4 ChE (18:1) (18:1) C45 H82 O2 N1 668.6340055 16.57979528 0.009441126 1.212481854 0.742692861 0.173576088 13347413.79 1089347.402 17308006.57 1960864.54 5980585.985 25018380.96 13846084.76 7992063.126 9227642.625 3889008.556 3585306.076 39913047.34 20001698.1 20328587.22 19997833

195POS Cer(d18:0+pO/24:0)+H Cer(d42:0+pO)+H Cer (d18:0+pO/24:0) (d18:0+pO) (24:0) C42 H86 O4 N1 668.6551355 12.8058727 0.006406561 1.413835698 0.157173988 0.216630042 4447342.958 4336011.383 3536038.187 4673156.241 5252823.007 4009416.06 2043972.5 10040623.43 8366403.755 4597928.01 5320871.84 6750156.748 8493152.335 8588243.443 8587953.683

196POS SM(d30:1)+Na SM(d30:1)+Na SM (d30:1) (d30:1) C35 H71 O6 N2 P1 Na1 669.4941975 7.21783974 0.072739836 1.230868662 0.455856597 0.075932693 1155920.05 975780.0088 1314280.123 1749379.29 1418143.87 1465888.159 489686.1439 2538942.561 2395292.795 957193.8088 989565.5231 2573988.972 2017684.199 2295270.985 2295128.316

197POS PC(29:4e)+H PC(29:4e)+H PC (29:4e) (29:4e) C37 H69 O7 N1 P1 670.4806185 6.333952042 0.01349731 2.233647101 0.0550422 0.214217723 708865.327 1143084.322 668637.8118 1182983.892 1197062.959 902543.8057 337048.6338 2066109.358 1769136.467 3574835.72 3807799.284 1407322.516 2397729.874 2454496.029 2454845.382

198POS PC(26:0)+Na PC(26:0)+Na PC (26:0) (26:0) C34 H68 O8 N1 P1 Na1 672.4574785 7.380614542 0.009943388 1.07634823 0.877659128 0.23718852 8115426.438 3797364.409 10535036.02 8095775.615 1939599.93 7910706.803 1736578.707 8743032.742 13257558.55 917726.7855 796733.3278 18026282.56 11566590.07 11766078.61 11770078.91

199POS PC(28:3)+H PC(28:3)+H PC (28:3) (28:3) C36 H67 O8 N1 P1 672.4598835 6.884234263 0.016530058 0.920708031 0.800242891 0.088389179 1777647.799 1103922.305 1873025.317 1106589.411 486847.1018 1172392.659 571422.3415 1383756.892 2063928.021 410430.2841 440801.1714 2053776.608 1810985.307 1810515.723 1759398.789

19POS WE(23:2)+NH4 WE(23:2)+NH4 WE (23:2) (23:2) H46 C23 O2 N1 368.3523055 2.97535831 0.002375065 1.091880785 0.830162773 0.115601705 4140012.311 34474098.36 4859630.141 38241512.78 23132356.93 10173287.68 695599.82 34816001.1 14973527 24879009.95 17821782.96 32403187.75 29279550.24 29279501.05 29159242.41

1POS So(d16:1)+H So(d16:1)+H So (d16:1) (d16:1) C16 H34 O2 N1 272.2584055 1.940826175 0.021798851 1.009389767 0.927515583 0.019778773 1380365.14 977267.7076 1405172.576 940311.6201 1294836.422 1392468.335 973384.5476 1322994.147 1231723.515 1122270.795 1200604.302 1608838.831 1826921.235 1759655.751 1759616.913

200POS SM(d32:2)+H SM(d32:2)+H SM (d32:2) (d32:2) C37 H74 O6 N2 P1 673.5279025 7.40110252 0.006651551 1.247738329 0.441198461 0.277634309 18803736.66 14701965.85 19861671.29 17457835.69 27110383.86 22774173.14 5381586.853 38492012.57 37939859 15618010.9 15703121.38 37479611.62 36711613.21 37203253.58 36956126.76

201POS PE(16:0p/16:1)+H PE(32:1p)+H PE (16:0p/16:1) (16:0p) (16:1) C37 H73 O7 N1 P1 674.5119185 10.29028538 0.006424011 0.943138702 0.679720196 0.36347693 131951754.1 108942905.3 143769952.5 171959885.9 152827465.3 169123382.6 75471734.24 169656799.3 133760602.1 119929538.2 133499510.6 196300227.1 173754435.2 175718782.2 175685178

202POS SM(d32:1)+H SM(d32:1)+H SM (d32:1) (d32:1) C37 H76 O6 N2 P1 675.5435525 8.441988097 0.003309724 0.997258269 0.991070058 1.939037844 1423673046 938011273.2 1470332451 1192382067 1161606736 1313723253 411764450.7 1574965963 1875872520 737101966.1 750692966.7 2128768719 1872699497 1883492548 1883459656

203POS PC(28:1)+H PC(28:1)+H PC (14:0/14:1) (14:0) (14:1) C36 H71 O8 N1 P1 676.4911835 7.77650342 0.062951992 1.059975922 0.886695492 0.161764354 18966482.22 4473558.432 18273595.98 6662838.002 3450998.481 9895419.425 9631667.455 22709151.83 8039780.814 3228737.437 4070009.388 17745433.02 16785036.79 15111735.06 15047713.25

204POS PC(28:1)+H PC(28:1)+H PC (14:0/14:1) (14:0) (14:1) C36 H71 O8 N1 P1 676.4911835 7.491820157 0.012052668 0.832829136 0.66921658 0.662202898 75505885.05 25488726.17 69148917.19 48995271.89 17747869.67 80244429.58 18837844.69 40136902.42 72280921.65 9190631.118 10497461.16 113172258.5 74598502.91 74681507.43 76207437.16

205POS PC(28:1)+H PC(28:1)+H PC (28:1) (28:1) C36 H71 O8 N1 P1 676.4911835 9.402256535 0.011005006 0.877987576 0.57366314 0.179487669 11287955.04 12220863.61 9423287.805 10948476.72 8186980.438 8375359.171 3663840.471 15800810.93 9630109.067 4899966.172 5803546.984 13269861.62 12700954.22 12462555.58 12461242.25

206POS PE(16:0p/16:0)+H PE(32:0p)+H PE (16:0p/16:0) (16:0p) (16:0) C37 H75 O7 N1 P1 676.5275685 10.9882051 0.008103365 1.059077757 0.762513585 0.246577229 30812070.84 25938176.06 35637462.91 36567745.22 58768939.39 41873917.83 17096974.95 49397926.75 34797608.23 40301857.65 44950746.7 56617351.3 51264577.69 51992266.32 51989515.26

207POS Cer(d18:1/26:1)+H Cer(d44:2)+H Cer (d18:1/26:1) (d18:1) (26:1) C44 H86 O3 N1 676.6602205 13.06122636 0.002068535 0.980117624 0.937539688 0.438462317 56794925.85 42845405.24 56041234.3 53348564.85 45121220.01 49741496.41 17126137.37 62574521.05 74402665.46 23856006.08 29694739.11 90196665.86 75577940.17 75853932.91 75587957.77

208POS SM(d32:0)+H SM(d32:0)+H SM (d32:0) (d32:0) C37 H78 O6 N2 P1 677.5592025 8.852414245 0.006236713 1.007806811 0.974160286 0.52522132 99767288.92 84718683.33 108554921.3 102150283.6 102416941.9 98852414.95 35643857.8 132504079.1 145502366.4 56163085.04 59145560.07 172158040.5 144036678.9 145607159.4 145600606.2

209POS PC(28:0)+H PC(28:0)+H PC (28:0) (28:0) C36 H73 O8 N1 P1 678.5068335 8.49627926 0.007787176 0.942803268 0.85861274 2.927005002 1648404495 933988281.2 1662716392 1468473151 804618551.4 1664341629 582765073 1388636598 2107632389 419544869.7 477594072.8 2738354810 1903516605 1929498197 1903898530

20POS AcCa(14:1)+H AcCa(14:1)+H AcCa (14:1) (14:1) C21 H40 O4 N1 370.2951855 1.773635515 0.007194852 0.813263536 0.542541125 0.151847664 1310072.628 5794140.552 1591416.042 10507446.07 8353600.81 6867721.79 3356231.652 5575960.538 5717800.568 2823051.839 2893560.75 7629502.211 7876561.638 7990636.951 7930132.821

210POS PC(29:0e)+H PC(29:0e)+H PC (29:0e) (29:0e) C37 H77 O7 N1 P1 678.5432185 9.602159003 0.004761496 0.965689023 0.878559166 0.210703862 17638714.27 20377207.3 15242329.3 15439810.63 18220921.61 14402790.93 10137213.92 25043571.89 27609207.38 5737546.285 11115860.01 18201925.47 20479245.67 20561963.74 20674398.68

211POS Cer(d18:1/26:0)+H Cer(d44:1)+H Cer (d18:1/26:0) (d18:1) (26:0) C44 H88 O3 N1 678.6758705 13.68348002 0.019552557 1.077917023 0.802794677 0.553379958 89413745.98 55256711.4 95905554.03 85917872.92 53617674.64 88249189.7 26697063.15 117786196 112492859.5 39644687.11 44410858.85 163822359.5 134399963.2 134472959.2 139040832.6

212POS Cer(d18:0/26:1)+H Cer(d44:1)+H Cer (d18:0/26:1) (d18:0) (26:1) C44 H88 O3 N1 678.6758705 13.26747369 0.00868235 0.955794225 0.917176763 0.348055155 12829848.13 24852855.19 13884449.3 25865332.58 12140947.56 27916605.74 6001891.827 21356862.9 26871755.47 3626489.264 3780847.738 50658453.14 28197368.66 28206860.91 28628282.83

213POS Cer(d18:0/26:0)+H Cer(d44:0)+H Cer (d18:0/26:0) (d18:0) (26:0) C44 H90 O3 N1 680.6915205 13.87350218 0.005452263 1.00702963 0.989798455 0.481904533 24570132.27 41503326.66 29479906.07 46075515.79 16423428.26 58594873.87 3306999.385 41890844.76 44734809.17 4327855.474 4863930.126 119045693.5 57304183.77 57789819.5 57892894.29

214POS SM(d33:4)+H SM(d33:4)+H SM (d33:4) (d33:4) C38 H72 O6 N2 P1 683.5122525 7.845 0.037737186 1.187364644 0.593334166 0.135714523 4497774.274 2623624.793 5033096.044 5071506.301 3743326.947 5223953.639 1031061.806 6588374.544 7332246.457 2918614.026 2888163.168 10342516.95 7444563.729 7954891.404 7951753.961

215POS LPC(28:1)+Na LPC(28:1)+Na LPC (28:1) (28:1) C36 H72 O7 N1 P1 Na1 684.4938635 8.196082552 0.01082625 1.682072488 0.046457524 0.324643888 4437818.022 2689650.34 5510037.806 5541432.525 3459841.333 4102037.988 2486548.224 8663824.344 8562477.214 6266879.875 6201121.176 11117070.97 8272013.522 8430385.037 8427763.254

216POS PC(30:4e)+H PC(30:4e)+H PC (30:4e) (30:4e) C38 H71 O7 N1 P1 684.4962685 7.48995089 0.015618107 1.198876679 0.594173768 0.06363823 1012230.579 1054213.998 999790.7866 862689.1497 1541158.358 1382150.374 333088.9402 2179530.96 2289224.269 582025.2206 529688.179 2301425.07 2057438.784 2058153.273 2113965.275

217POS PC(30:4e)+H PC(30:4e)+H PC (30:4e) (30:4e) C38 H71 O7 N1 P1 684.4962685 7.01013518 0.019561604 1.742042893 0.084182031 0.133628405 550207.4238 734442.9246 534732.2206 789134.1332 900333.3184 742063.0785 90059.25609 1106210.644 1317993.081 1736902.432 1986287.853 1167819.686 1384700.752 1432524.045 1385427.039

218POS DG(18:1/22:6)+NH4 DG(40:7)+NH4 DG (18:1/22:6) (18:1) (22:6) C43 H74 O5 N1 684.5561505 10.96040019 0.011452973 1.151089042 0.632906876 0.358572675 48482291.7 57657600.78 49352979.69 74707047.87 48465541.34 28111699.03 12283846.32 120165214.9 68589730.31 51192104.44 52541133.67 48355797.99 75773810.35 77293174.58 75786087.87

219POS LPC(28:0)+Na LPC(28:0)+Na LPC (28:0) (28:0) C36 H74 O7 N1 P1 Na1 686.5095135 9.124719663 0.016901267 2.013455532 2.83111E-05 0.640631253 11981517.55 14866286.58 11028962.45 14111040.46 12126607.03 11491262.88 17451572.24 24968260.59 27700726.14 26777693.1 26919042.36 28411374.1 20801308.82 20802280.65 20198731.3

21POS WE(23:1)+NH4 WE(23:1)+NH4 WE (23:1) (23:1) H48 C23 O2 N1 370.3679555 3.950136315 0.007474929 0.939847808 0.836224818 0.019597244 720220.6556 1539641.727 811229.089 1771733.55 1317135.99 522939.552 169058.3774 1723527.176 1056510.216 935562.4133 722080.8949 1674170.369 1610690.779 1589918.238 1610673.327

220POS DG(18:1/22:5)+NH4 DG(40:6)+NH4 DG (18:1/22:5) (18:1) (22:5) C43 H76 O5 N1 686.5718005 11.20425233 0.056731056 0.885777774 0.639774535 0.084084249 12720993.09 20441948.23 13842225.44 15885715.2 11309179.93 8967804.87 2211446.03 23320371.84 15904020.56 10406336.5 10700417.27 11125655.65 24197357.05 21962956.77 21968808.48

221POS DG(18:0/22:6)+NH4 DG(40:6)+NH4 DG (18:0/22:6) (18:0) (22:6) C43 H76 O5 N1 686.5718005 11.61321382 0.00271698 1.099700688 0.712190943 0.259937121 46663234.1 35887582.16 51472309.47 34333649.76 25787309.1 23663444.42 12557266.67 70674982.24 56571136.1 33046789.55 31125817.01 35547097.9 53656117.06 53909432.74 53909396.39

222POS SM(d33:2)+H SM(d33:2)+H SM (d33:2) (d33:2) C38 H76 O6 N2 P1 687.5435525 8.054682282 0.003258741 1.130143973 0.667368667 0.133164971 5726604.11 3610047.289 5087294.465 3878592.451 4278944.794 4843340.095 1540982.58 6695631.188 8103512.543 2865498.276 2690457.875 9097916.206 7348338.447 7390054.948 7389887.288

223POS PC(29:2)+H PC(29:2)+H PC (29:2) (29:2) C37 H71 O8 N1 P1 688.4911835 9.02507817 0.016459096 0.856538353 0.537653205 0.6085728 84549661.32 63489201.98 104492704.2 97609135.95 90351493.53 121464071.2 37083826.86 77435834.73 82506405.54 55220436.03 57403033.56 171687559.7 116169796.2 119581206.6 119509063.1

224POS PC(29:2)+H PC(29:2)+H PC (29:2) (29:2) C37 H71 O8 N1 P1 688.4911835 9.347 0.058903515 1.160358634 0.534902227 0.152674299 14241428.03 10626613.44 10518465.19 9672542.127 5546023.819 14176903.86 3916416.146 15620489.01 8838578.404 12234598.49 13894182.71 20666060.95 20801368.27 20857582.65 23028836.41

225POS PE(16:0p/17:1)+H PE(33:1p)+H PE (16:0p/17:1) (16:0p) (17:1) C38 H75 O7 N1 P1 688.5275685 10.68237101 0.003308111 1.067447298 0.604708992 0.436311882 71232893.67 71874716.2 79934420.6 86607098.2 96274389.72 78753722.49 42617170.24 111266102.7 91190767.03 84842029.42 93154842.2 94296499.82 106189756.1 106797240.9 106185547.3

226POS PC(30:2e)+H PC(30:2e)+H PC (30:2e) (30:2e) C38 H75 O7 N1 P1 688.5275685 9.101819673 0.085777943 0.97504301 0.924141459 0.226515669 39315772.29 22551271.49 104029388.6 39154451.48 43647220.84 45276425.28 39286798.8 34850405.92 37682096.2 53563580.63 65014106.53 56240822.53 50522740.09 43409819.96 50612968.74

227POS DG(18:1/22:4)+NH4 DG(40:5)+NH4 DG (18:1/22:4) (18:1) (22:4) C43 H78 O5 N1 688.5874505 11.66738605 0.009839816 1.025711285 0.922710614 0.211698461 40354497.89 31654165.88 40170120.79 33300372.26 25167399.36 21835196.25 11033981.9 60781546.15 48814462.96 20702945.11 22183936.38 33913833.07 47089882.68 46293845.24 46298864.54

228POS SM(d33:1)+H SM(d33:1)+H SM (d33:1) (d33:1) C38 H78 O6 N2 P1 689.5592025 8.994412942 0.008967398 1.140467847 0.529545166 1.568063188 1085540590 819317949 1078934971 994575193.8 1107517859 1111221013 411891066.3 1556385129 1549140678 834264566.5 925372097.6 1790548395 1536449188 1560737228 1537050630

229POS PC(29:1)+H PC(29:1)+H PC (29:1) (29:1) C37 H73 O8 N1 P1 690.5068335 9.840497237 0.007313695 0.875774586 0.325805307 1.466650594 511341667 445909684.8 564068848.7 502931711 473459655.4 612921171.1 330570384 531984154 440145742.8 351981723.8 371982686.7 697548406.7 568114442.5 575374856.2 575370012.4

22POS AcCa(14:0)+H AcCa(14:0)+H AcCa (14:0) (14:0) C21 H42 O4 N1 372.3108355 2.032617555 0.002862817 1.179798106 0.752851719 0.464444459 21482104.01 77187733.61 32480373.82 101726202.4 59151898.66 157185580 34791022.99 73109683.77 104600508.1 29991767.16 28451555.97 259037161.5 114091898.7 114658456.5 114091682.6

230POS PC(30:1e)+H PC(30:1e)+H PC (30:1e) (30:1e) C38 H77 O7 N1 P1 690.5432185 9.923885033 0.010372619 0.999456722 0.997221422 0.56037305 272910689.9 306518091.5 282606063.5 310784618.5 377760092 332459133.3 164565683.2 369548814.4 432302633.8 241420077.6 248995226.2 425183240.2 380889368.6 381002918.9 387830712

231POS PC(30:1e)+H PC(30:1e)+H PC (30:1e) (30:1e) C38 H77 O7 N1 P1 690.5432185 9.23977971 0.010810605 1.121659762 0.651637705 0.643662982 124523945.6 102464502.8 134348892.8 102511717.4 97577901.95 133546144.4 49343216.29 159317348 203115604 80927618.23 70582328.05 216237253 163392177.9 160345079.7 163378609.1

232POS DG(18:0/22:4)+NH4 DG(40:4)+NH4 DG (18:0/22:4) (18:0) (22:4) C43 H80 O5 N1 690.6031005 12.230278 0.033747556 1.20134362 0.534258578 0.19253278 13026429.77 11970234.27 12142804.82 9310590.178 9238168.885 7606546.876 2955628.023 24386274.24 19083349.93 8752080.575 8715188.238 12146252.87 18242050.7 18223716.19 19319700.45

233POS ChE(20:4)+NH4 ChE(20:4)+NH4 ChE (20:4) (20:4) C47 H80 O2 N1 690.6183555 15.4790873 0.002395589 1.176635361 0.599714183 0.064478471 6665407.273 721159.603 5518142.272 1213561.478 3208880.672 4278197.618 2565952.774 3682588.419 4558501.237 3647484.149 3614020.983 7353069.958 6877302.096 6905901.091 6877349.179

234POS PC(29:0)+H PC(29:0)+H PC (29:0) (29:0) C37 H75 O8 N1 P1 692.5224835 8.860087815 0.200886909 1.15296551 0.664445191 0.652481778 179706935.3 38468779.65 209171156 33080792.18 118836790.3 47991118.03 63810022.44 151640119.4 93265386.81 161104727.4 160486097 92897686.97 83911686.36 84665309.93 117456891.8

235POS PE(32:0)+H PE(32:0)+H PE (32:0) (32:0) C37 H75 O8 N1 P1 692.5224835 10.58361621 0.005518202 0.942134725 0.715315325 0.28871223 30782977.36 35617952.72 35379004.07 36212943.01 53163679.41 34186367.38 14958338.28 43538927.63 27410056.34 42016933.81 39820793.56 44558344.15 45058871.66 45491819.9 45492773.23

236POS PC(30:0e)+H PC(30:0e)+H PC (30:0e) (30:0e) C38 H79 O7 N1 P1 692.5588685 10.04757889 0.002064054 0.842770693 0.467071458 1.802205104 444284173 390552272 432921690.2 473673990.7 476414166.3 578352306.8 180056120.9 472572978.1 538820216.2 211764995.7 210238720.3 743101201 525392415.5 527278873.6 525404259.3

237POS ChE(20:3)+NH4 ChE(20:3)+NH4 ChE (20:3) (20:3) C47 H82 O2 N1 692.6340055 15.79988468 0.007680158 1.22614364 0.681367574 0.175926351 10749792.35 1807671.092 8839308.433 3724878.909 5679174.537 12224657.47 4015785.166 8464308.082 8654175.385 3394599.785 2964889.859 25261663.79 14481160.7 14481845.36 14289715.27

238POS PE(14:0p/20:5)+H PE(34:5p)+H PE (14:0p/20:5) (14:0p) (20:5) C39 H69 O7 N1 P1 694.4806185 8.683 0.035465771 1.04933479 0.899944034 0.060696965 14134191.32 8995944.421 17085142.86 2182683.668 3391454.708 4007653.371 13860406.13 6380840.421 15465051.84 4465914.36 6723132.887 5358452.741 8004334.004 7501327.313 7569822.03

239POS DG(18:1/22:1)+NH4 DG(40:2)+NH4 DG (18:1/22:1) (18:1) (22:1) C43 H84 O5 N1 694.6344005 12.955 0.016949753 1.134586082 0.708308493 0.138406795 7220885.994 7230439.993 7916289.936 10863667.47 7099000.942 6137376.147 1426898.102 19131816.42 12084601.03 5146722.695 4482344.704 10449177.88 12678078.23 13057355.15 13058062.29

23POS ZyE(0:0)+H ZyE()+H ZyE (0:0) (0:0) C27 H45 O1 385.3464915 6.084237488 0.016607907 0.58729854 0.182849726 0.18548316 6106148.031 5320870.079 6069430.998 10895027.36 3661433.918 2074388.637 347963.5478 6559386.066 6802583.337 2291245.735 1827872.553 2213861.664 6373911.096 6196903.397 6191249.032

240POS SM(d32:2)+Na SM(d32:2)+Na SM (d32:2) (d32:2) C37 H73 O6 N2 P1 Na1 695.5098475 7.40110252 0.017305102 1.765516999 0.176506999 0.428116309 11527433.92 12760138.1 558328.0328 14862448.88 27254900.66 17533839.02 3130231.658 45271038.02 34866625.32 15070946.02 15781077.27 35061128.05 33953375.85 34998143.2 33986789.94

241POS DG(16:0/24:1)+NH4 DG(40:1)+NH4 DG (16:0/24:1) (16:0) (24:1) C43 H86 O5 N1 696.6500505 13.47695335 0.011137575 1.25332983 0.562387398 0.302971782 15261344.26 16347792.67 17961458.62 25114729.35 13283573.9 14811798.22 2948350.59 46986914.01 33400456.42 9580170.339 8403282.804 27498939.31 29769762.36 29769789.66 30347776.86

242POS SM(d32:1)+Na SM(d32:1)+Na SM (d32:1) (d32:1) C37 H75 O6 N2 P1 Na1 697.5254975 8.441988097 0.002868482 0.90276125 0.369175364 1.121003351 426206473.3 352252741.7 433072307.4 400448394.4 440633554.8 425233579.4 197261567.5 468501404.2 429676693.2 347317492.3 350537315.1 443609829.9 478215110.4 478219452.9 475845258.8

243POS PC(14:0/14:1)+Na PC(28:1)+Na PC (14:0/14:1) (14:0) (14:1) C36 H70 O8 N1 P1 Na1 698.4731285 7.491820157 0.017507383 1.076903485 0.872497557 0.290186651 11667794 6943668.311 14040805.64 15306481.35 4929620.622 16984754.66 3009309.566 14496639.15 21508375.83 1788131.837 2420973.813 32023181.18 20332292.67 20960112.91 20963004.4

244POS PC(14:0/14:1)+Na PC(28:1)+Na PC (14:0/14:1) (14:0) (14:1) C36 H70 O8 N1 P1 Na1 698.4731285 7.77650342 0.094859925 1.287081616 0.529712031 0.134776548 3014710.855 1152242.544 3951273.406 2384102.925 1572395.673 3508308.228 795200.865 6940210.351 3543805.158 1451120.125 1418874.538 5907425.07 8487154.81 7152562.432 8456862.746

245POS PC(30:4)+H PC(30:4)+H PC (30:4) (30:4) C38 H69 O8 N1 P1 698.4755335 7.117001978 0.037955132 0.824786129 0.604078909 0.143478551 3995218.642 1540557.817 3755898.342 2236429.103 1174859.227 3070945.52 732630.134 2489884.683 4010221.196 670272.4502 639967.6786 4467124.918 3367367.114 3150198.259 3366198.533

246POS PE(16:0p/18:3)+H PE(34:3p)+H PE (16:0p/18:3) (16:0p) (18:3) C39 H73 O7 N1 P1 698.5119185 10.093 0.019296232 1.195187947 0.289755518 0.20018719 17264232.81 11457283.79 34290858.81 20430446.92 24076977.37 23434594.53 26772246.47 27207636.67 26966423.8 15741599.7 31252001.76 28575205.24 25196668.57 26059031.08 26056933.6

247POS CerG1(d18:2/16:0)+H CerG1(d34:2)+H CerG1 (d18:2/16:0) (d18:2) (16:0) C40 H76 O8 N1 698.5565455 8.988607912 0.020950273 0.924094918 0.795818554 0.334338738 19695952.22 13287407.39 21080248.8 15102419.87 11854195.72 24032606.63 8990000.614 18168411.25 25351593.54 6078729.43 5571583.399 32918468.71 22598968.23 23439448.92 23439241.83

248POS DG(16:0/24:0)+NH4 DG(40:0)+NH4 DG (16:0/24:0) (16:0) (24:0) C43 H88 O5 N1 698.6657005 14.08882408 0.011503599 1.130876086 0.743140416 0.156461233 5614818.44 3345421.681 5181417.669 5306837.391 1923344.749 3493948.45 1116898.088 8685801.337 8100730.85 1425917.21 1569626.049 7221151.913 7283014.895 7286594.328 7430890.376

249POS SM(d32:0)+Na SM(d32:0)+Na SM (d32:0) (d32:0) C37 H77 O6 N2 P1 Na1 699.5411475 8.852414245 0.005294102 0.867888268 0.158626456 0.303821851 25936483.54 28435759.54 27209339.17 27607705.66 30426252.04 22735507.4 17568538.17 31871333.18 26524101.73 21611506.19 23528306.44 19798783.52 27596222.16 27348352.37 27341222.06

24POS AcCa(15:0)+H AcCa(15:0)+H AcCa (15:0) (15:0) C22 H44 O4 N1 386.3264855 2.281262043 0.003986275 1.257387235 0.553519734 0.131229184 3148026.417 14599185.33 4310290.894 12766337.23 8205521.59 15592826.53 4056104.547 13154725.34 15108503.91 7413125.604 6548060.758 27430270.7 15497153.81 15604335.68 15604956.89

250POS PC(28:0)+Na PC(28:0)+Na PC (28:0) (28:0) C36 H72 O8 N1 P1 Na1 700.4887785 8.49627926 0.00438069 0.805249208 0.233009851 1.222812804 220865732.1 172451251.1 234074769 236407133.1 145394700.4 235366575.2 139308818.2 189577349.8 224893721.3 94525422.65 97200739.4 256675032.8 221511505.4 219842650.6 219835827.6

251POS PC(30:3)+H PC(30:3)+H PC (30:3) (30:3) C38 H71 O8 N1 P1 700.4911835 8.173270835 0.012489207 0.941520753 0.870906094 0.423679358 32728450.04 19576003.99 37774552.23 20797403.7 13342236.98 25179157.3 8951735.012 30673786.62 39965560.68 5282866.396 5784734.345 50002450.11 36854169.35 37661891.48 37664227.04

252POS PC(30:3)+H PC(30:3)+H PC (30:3) (30:3) C38 H71 O8 N1 P1 700.4911835 7.421392045 0.015092536 1.120754652 0.689036277 0.156272429 9620252.21 4596674.972 8782443.877 5068434.168 4007061.761 8292272.219 5829233.318 8025652.027 11278548.17 3131659.274 3561314.053 13415252.21 11210975.53 11507645.76 11212987.91

253POS PE(16:0p/18:2)+H PE(34:2p)+H PE (16:0p/18:2) (16:0p) (18:2) C39 H75 O7 N1 P1 700.5275685 10.44715058 0.026485863 1.073883441 0.562578683 0.645851709 112538035.9 93885131.5 156720931.8 120962762.9 134140631.8 121845704.1 76963607.7 147881207.9 117072085.7 146414081.1 160738486.4 145704361.4 156067040.9 155835130.3 163213599

254POS CerG1(d18:1/16:0)+H CerG1(d34:1)+H CerG1 (d18:1/16:0) (d18:1) (16:0) C40 H78 O8 N1 700.5721955 9.824401123 0.000133125 0.884794376 0.593574514 1.888927587 581674829.8 526409674 570545279 502850678.1 394502656.8 636828486.1 367485176.2 605110626.3 699251710.3 204511896.3 175752523.4 790565706.8 630995387.2 630849913.1 630995404.8

255POS CerG1(d18:1/16:0)+H CerG1(d34:1)+H CerG1 (d18:1/16:0) (d18:1) (16:0) C40 H78 O8 N1 700.5721955 8.20735035 0.006870129 1.082300265 0.713528396 0.120436461 16790629.07 9798411.367 27910075.45 28912737.8 19624565.9 22609305.88 12409140.87 25859662.77 19530650.82 22899357.77 17853833.18 37433756.57 24706645.9 25002621.41 25003359.96

256POS SM(d34:2)+H SM(d34:2)+H SM (d34:2) (d34:2) C39 H78 O6 N2 P1 701.5592025 8.603998178 0.004831996 1.077679745 0.76630604 1.925823702 1515593850 1085618129 1763463922 1397837769 1368857560 1891695077 575797662 2239677885 2089087401 944949547.6 1038622578 2835840726 2202949556 2221474032 2203020836

257POS PC(30:2)+H PC(30:2)+H PC (30:2) (30:2) C38 H73 O8 N1 P1 702.5068335 8.292158323 0.00380004 1.235954359 0.579535744 0.664921748 89047319.42 49557177.03 129989625.4 58147911.09 43204984.89 89736329.41 39320498.89 107014750.7 122218516.8 41152459.38 41739983.11 216701427.7 120940999.5 121747981.8 120959633.8

258POS PC(30:2)+H PC(30:2)+H PC (30:2) (30:2) C38 H73 O8 N1 P1 702.5068335 9.508992803 0.03327869 0.859110957 0.195452208 0.388151423 39380994.08 39871246.98 42193101.68 43578243.54 38766292.81 37823296.35 18327129.86 38697421.26 36826235.19 28453543.72 39142557.73 46125638.61 40796434.54 43230866.97 43252838.01

259POS PC(30:2)+H PC(30:2)+H PC (30:2) (30:2) C38 H73 O8 N1 P1 702.5068335 7.750130955 0.037818358 1.067167202 0.905472483 0.675696811 147473115.6 36501951.66 71470315.38 99505892.45 24324456.01 100997808.3 15828217.51 84488438.67 90255920.46 37038116.06 11496919.75 273424556.7 131796743.5 135115752.3 125387269.4

25POS AcCa(16:1)+H AcCa(16:1)+H AcCa (16:1) (16:1) C23 H44 O4 N1 398.3264855 2.130767748 0.003472204 0.789830846 0.554017768 0.417858034 30731157.01 84411427.58 40476485.92 197542895.2 82892396.89 66883411.43 13737415.48 86519877.46 80480802.76 54308970.97 42599068.99 119589632 106981823.9 107625931.8 107629664.3

260POS PC(31:1p)+H PC(31:1p)+H PC (31:1p) (31:1p) C39 H77 O7 N1 P1 702.5432185 11.007 0.001187486 1.068138347 0.668115439 2.362823269 1819034036 1808809227 2073146677 2525140514 2897347433 2230503030 958880704 2973286408 2374903687 2494853495 2696818007 2765156798 2987044796 2980881217 2986996253

261POS SM(d34:1)+H SM(d34:1)+H SM (d34:1) (d34:1) C39 H80 O6 N2 P1 703.5748525 9.48662391 0.003836371 1.075329128 0.345924164 5.175981185 16074634296 14602638482 16940748235 15803951430 16949418551 15940880326 11631202055 19278963075 18855581698 17434992348 17468794896 18897856675 18354249870 18477416208 18476078107

262POS PC(30:1)+H PC(30:1)+H PC (30:1) (30:1) C38 H75 O8 N1 P1 704.5224835 10.26443872 0.003109275 0.961691989 0.768633137 0.443270902 175166711.9 140618063.6 186528346.6 154998751.8 133313238.2 167280751.4 95324243.41 200588116.7 184829389 122191000.7 123397719.2 194879926.4 200108103.3 201184115.1 200100991.4

263POS PC(30:1)+H PC(30:1)+H PC (30:1) (30:1) C38 H75 O8 N1 P1 704.5224835 8.633686167 0.005070261 0.991844106 0.976204802 3.982297397 5021167072 3187581886 4568988892 4389471277 3179864341 6071817281 1838649835 4664412548 6126114953 2406332463 2516528517 8651382766 5894702332 5946482367 5947061131

264POS PC(31:0p)+H PC(31:0p)+H PC (31:0p) (31:0p) C39 H79 O7 N1 P1 704.5588685 10.32584034 0.011451073 1.069264714 0.564115737 0.039733478 43943460.62 44368164.78 41612299.43 36718440.41 53910422.13 45267681.53 36573462.87 55614942.28 62466897.58 36620360.65 39188386.4 53768397.91 51824448.68 50812416.8 50807569.6

265POS PE(18:0p/16:0)+H PE(34:0p)+H PE (18:0p/16:0) (18:0p) (16:0) C39 H79 O7 N1 P1 704.5588685 11.64639477 0.009766585 1.002075778 0.991230506 0.014683835 33004986.48 25683281.96 40024042.04 38245698.41 44988810.17 43938983.54 14945505.78 50767170.59 36748566.27 33437068.84 31856020.93 58600359.03 56006355.96 56958278.94 56004656.14

266POS SM(d34:0)+H SM(d34:0)+H SM (d34:0) (d34:0) C39 H82 O6 N2 P1 705.5905025 9.83142037 0.00172238 0.994682163 0.965154365 1.116310265 2143996516 1862791370 2327464042 2238777759 2161594614 2063295352 1353781063 2728778356 2530929875 1668120049 1753211226 2695041828 2504622896 2497152151 2504610338

267POS PC(30:0)+H PC(30:0)+H PC (30:0) (30:0) C38 H77 O8 N1 P1 706.5381335 9.48992238 0.003474277 0.88510026 0.245315525 6.122916768 7273434137 6863926156 7212576220 7627934113 6681854751 7361507917 4521646321 6786588017 7921018309 5165763502 5278264916 8404823715 7601666555 7602748771 7648037232

268POS PC(31:0e)+H PC(31:0e)+H PC (31:0e) (31:0e) C39 H81 O7 N1 P1 706.5745185 10.46746551 0.043917989 0.71418231 0.175309987 0.720892427 38746289.22 46012563.92 38191646.99 46523011.28 53091016.06 53599293.27 24226619.41 4889619.559 52796157.56 24625507.58 28259669.41 62433741.91 38148005.28 35396156.93 38309736.31

269POS PC(12:0p/20:5)+H PC(32:5p)+H PC (12:0p/20:5) (12:0p) (20:5) C40 H71 O7 N1 P1 708.4962685 9.244 0.018267712 1.325278193 0.012369345 0.239533842 7703399.747 6086941.932 7829667.694 11670170.16 8187142.404 7961244.609 9242960.588 12069606.45 10428823.71 10240819.22 11902404.23 11635239.96 13548604.37 13132333.29 13125301.46

26POS AcCa(16:0)+H AcCa(16:0)+H AcCa (16:0) (16:0) C23 H46 O4 N1 400.3421355 2.622819605 0.011661291 1.358615122 0.492836839 0.961270464 95371571.55 438268626.5 126444125.5 393663958.1 239799252.6 673277664.5 125967195.2 469822867 483163179.9 259238969.5 252826994.8 1081139250 552734821.8 552803251 564009357.9

270POS SM(d33:2)+Na SM(d33:2)+Na SM (d33:2) (d33:2) C38 H75 O6 N2 P1 Na1 709.5254975 8.054682282 0.011394989 1.078991322 0.731685799 0.05873034 2641258.683 1379550.294 3648867.913 2509359.027 2430735.373 2997716.553 996760.3607 4218786.07 3194723.93 2531554.411 1984130.678 3914388.488 3620804.783 3694108.345 3692297.952

271POS PE(32:2)+Na PE(32:2)+Na PE (16:1/16:1) (16:1) (16:1) C37 H70 O8 N1 P1 Na1 710.4731285 9.025906462 0.027735012 0.903335014 0.617823845 0.150328342 8314318.14 6854675.004 9274432.742 10233124.94 11603482.46 10197853.9 4827242.711 6402556.103 8423823.859 6763617.432 8425379.847 16175833.04 8968303.779 8544734.991 8969277.465

272POS PC(32:4p)+H PC(32:4p)+H PC (32:4p) (32:4p) C40 H73 O7 N1 P1 710.5119185 7.419433623 0.01052486 2.884669026 0.002087196 0.428962244 3219538.003 2339003.631 2675160.932 2340588.435 2305210.318 1859859.227 1910152.49 8179146.479 9624484.948 6649378.863 7678545.417 8476468.632 7796805.652 7939842.846 7941516.064

273POS SM(d33:1)+Na SM(d33:1)+Na SM (d33:1) (d33:1) C38 H77 O6 N2 P1 Na1 711.5411475 8.994412942 0.002048905 0.991735398 0.909528966 0.635558013 171317922.2 180430604 170618516.2 187886425.4 214161345 161362017.6 158230375.7 210512538.4 171555448.2 202418235.6 188173875.7 145912843.1 172822049.5 172210138.8 172210231.2

274POS PC(29:1)+Na PC(29:1)+Na PC (29:1) (29:1) C37 H72 O8 N1 P1 Na1 712.4887785 8.192800023 0.074655007 1.269856766 0.543725066 0.244976824 11455844.48 4600947.218 12758039.23 9109602.846 4711456.596 10893996.15 3419588.091 25271336.11 13774451.02 5051769.701 4149569.913 16308573.77 18787110.03 16557484.97 16543801.39

275POS PC(32:4e)+H PC(32:4e)+H PC (32:4e) (32:4e) C40 H75 O7 N1 P1 712.5275685 8.217763048 0.008616723 2.663520752 0.004720589 0.788080269 10236326.91 8149099.303 12775287.82 11053692.14 7765804.889 7643010.655 7337137.142 29598076.67 29809574.32 24049886.82 23339756.14 39346215.72 26261418 26566888.11 26708401.75

276POS PC(30:1e)+Na PC(30:1e)+Na PC (30:1e) (30:1e) C38 H76 O7 N1 P1 Na1 712.5251635 9.23977971 0.002059171 0.9994488 0.994511475 0.087568514 11621465.62 10496694.5 10656847.58 11474005 10679520.79 11813370.38 9641734.665 11813865.13 12362998.34 9752211.182 8871188.713 14263117.67 10464112.68 10426846.9 10426913.23

277POS PC(31:3)+H PC(31:3)+H PC (31:3) (31:3) C39 H73 O8 N1 P1 714.5068335 9.230325937 0.015410952 0.818369787 0.402040589 0.625247687 67033981.84 44833950.08 74573797.19 67844922.56 141222562.8 86649846.45 47266178.44 80400227.66 67757736.65 46415333.29 46273247.32 106471684.4 93262924.24 93268668.56 90798254.34

278POS PE(18:1p/17:1)+H PE(35:2p)+H PE (18:1p/17:1) (18:1p) (17:1) C40 H77 O7 N1 P1 714.5432185 10.73450815 0.005268553 1.413024955 0.103065103 0.650696505 27523302.13 21398958.75 28798735.97 37918526.76 37508627.73 35216128.8 16363360.55 55042597.74 34220767.76 53017039.38 46733660.1 60786002.86 42492879.56 42059123.36 42189291.16

27POS WE(27:6)+NH4 WE(27:6)+NH4 WE (27:6) (27:6) H46 C27 O2 N1 416.3523055 2.392825197 0.006120079 1.084897507 0.874767789 0.017015398 251335.0011 2253162.234 302132.5233 1998108.73 1228584.602 305863.4317 13483.10352 2912228.338 1482976.535 651980.3008 569118.2639 1247581.112 1738447.65 1738462.5 1720091.842

280POS SM(d35:2)+H SM(d35:2)+H SM (d35:2) (d35:2) C40 H80 O6 N2 P1 715.5748525 9.13738 0.01086008 1.277060668 0.25756898 0.525456505 42893000.87 35675394 46260343.77 35491595.02 45809772.41 39252441.16 17420049.18 71284635.27 78332060.28 40627657.34 43782981.08 61921016.52 68659847.96 69968218.26 68677124.7

281POS PC(31:2)+H PC(31:2)+H PC (31:2) (31:2) C39 H75 O8 N1 P1 716.5224835 9.928875177 0.002127043 0.929910614 0.609186977 1.724988751 1552477164 1203481190 1541274607 1602542631 1640223685 1828478464 738373236.6 1601360778 1621421218 1248591891 1362631624 2139468143 1868630602 1861755377 1868631888

282POS PC(31:2)+H PC(31:2)+H PC (31:2) (31:2) C39 H75 O8 N1 P1 716.5224835 8.358971243 0.050638147 0.93002239 0.872325246 0.575526045 117967589.4 58332367.11 44361580.67 38052336.41 27168040.88 48639221 14238290.98 52835677.23 61273256.5 20216219.87 20556771.51 141991929.7 104029792.7 104188181.5 113514129.9

283POS PC(31:2)+H PC(31:2)+H PC (31:2) (31:2) C39 H75 O8 N1 P1 716.5224835 8.822704887 0.036642262 1.005026164 0.985383561 0.478183589 83735669.14 51392399.95 78718155.31 41930823.55 37713432.83 64884467.5 32902836.38 65948405.99 80536934.97 32948107.24 31977411.76 115862503.3 79930914 85232469.62 85223422.83

284POS PC(32:1p)+H PC(32:1p)+H PC (32:1p) (32:1p) C40 H79 O7 N1 P1 716.5588685 10.04515487 0.103868163 1.643030762 0.124497746 3.004338288 317878627.8 345130525.9 334418137.4 307899764.7 950118229.4 444464219.8 168950797.6 939346788.8 930307109.7 917852820.7 1011283387 468293466.3 884526118.5 883925639.9 734149978.9

285POS PE(18:0p/17:1)+H PE(35:1p)+H PE (18:0p/17:1) (18:0p) (17:1) C40 H79 O7 N1 P1 716.5588685 11.37131015 0.107281247 1.239149479 0.298750051 0.927821367 145586565.2 135963012.9 150918778.9 188968474.5 211475374.8 250587437.2 71219218.56 273305523.7 292334697.3 291085708.8 184358122.7 230314747.6 352732374.4 290831614.3 352292992.2

286POS ChE(22:5)+NH4 ChE(22:5)+NH4 ChE (22:5) (22:5) C49 H82 O2 N1 716.6340055 15.45658372 0.005995773 1.897133397 0.030593739 0.516780627 16161856.46 4879220.547 14748684.03 7192095.14 22472486.46 27304108.23 21036017.34 33622326.78 39217027.19 20199577.85 19390601.34 42509604.54 38510814.36 38913302.4 38913770.6

287POS SM(d34:2+hO)+H SM(d34:2+hO)+H SM (d34:2+hO) (d34:2+hO) C39 H78 O7 N2 P1 717.5541175 8.236591273 0.002381398 1.201422063 0.607493471 0.264723379 11715680.07 9477960.308 14921511.37 13161441.01 9577863.783 13200212.03 3350481.46 20856095.57 22150018.13 6025819.169 5006714.662 29178939.6 19510651.18 19547557.41 19603154.57

289POS PC(31:1)+H PC(31:1)+H PC (31:1) (31:1) C39 H77 O8 N1 P1 718.5381335 6.991546528 0.005315216 0.993155312 0.97274582 0.048961925 2537501.863 1938164.268 2580200.889 2410725.543 2324126.009 1865051.906 741845.8547 2823653.935 3566658.159 1687674.951 1744029.822 2998438.263 3150187.043 3179426.608 3179307.964

28POS AcCa(18:1)+H AcCa(18:1)+H AcCa (18:1) (18:1) C25 H48 O4 N1 426.3577855 2.743 0.020936308 1.003506118 0.991784163 0.07687431 63448818.29 298529869.3 74536407.94 428816484.5 210857739.1 199916705.6 33412693.64 315560928.8 247169319.8 190243719.6 186756538.8 307437002.4 318563322.8 330291851.1 318631526.5

290POS PC(31:1)+H PC(31:1)+H PC (31:1) (31:1) C39 H77 O8 N1 P1 718.5381335 10.64364244 0.005108405 0.993866518 0.953617606 1.001661161 2862858316 2444285754 3185669152 2861093644 2708425003 2882604023 1856800161 3280506002 3038745955 2488560701 2457442219 3718949390 3457532392 3422432989 3438509000

291POS PC(31:1)+H PC(31:1)+H PC (31:1) (31:1) C39 H77 O8 N1 P1 718.5381335 9.125447373 0.004960488 0.955654572 0.836205401 1.86009024 1485457345 1005171671 1474009820 1267183499 1652220998 1615141279 505944896.2 1578672416 2007048657 850429447.9 968451578.6 2211737640 1971427871 1988394267 1988532087

292POS PC(32:1e)+H PC(32:1e)+H PC (32:1e) (32:1e) C40 H81 O7 N1 P1 718.5745185 10.14075531 0.00254999 0.862550544 0.579939723 1.994917158 759783170.2 900429215.6 799415037.9 766133267.9 833845756.8 1245053459 295254125 762522485.8 855675165.9 367892730.2 644217949.2 1649974835 1002159318 997771374 997721180

293POS PC(32:1e)+H PC(32:1e)+H PC (32:1e) (32:1e) C40 H81 O7 N1 P1 718.5745185 10.73446061 0.005245069 1.117946884 0.522024933 1.645027289 646434189.2 753996141.4 626699294 798090498.2 1346328405 936939767.1 434051372.6 1066437736 978288053.4 1052707445 1088857840 1090676122 1065021476 1074688074 1074822252

294POS ChE(22:4)+NH4 ChE(22:4)+NH4 ChE (22:4) (22:4) C49 H84 O2 N1 718.6496555 16.04925319 0.014751463 1.982000351 0.054170685 0.148693631 1808901.279 393148.3603 1094763.845 605539.5584 1972968.506 3169601.533 2398827.639 3394360.266 3455073.161 1871041.379 1642660.653 5165077.629 4165363.175 4059744.758 4165176.272

295POS SM(d34:1+pO)+H SM(d34:1+pO)+H SM (d34:1+pO) (d34:1+pO) C39 H80 O7 N2 P1 719.5697675 8.020636937 0.007704826 1.143479502 0.605606916 0.177526765 10188420.07 7898170.434 10019667.87 10155396.69 8932609.802 8551470.862 3288776.104 15359902.29 15788586.1 6058228.434 6279562.997 16969050.19 14228638.69 14418567.38 14421843.26

296POS PE(34:0)+H PE(34:0)+H PE (34:0) (34:0) C39 H79 O8 N1 P1 720.5537835 11.27917793 0.012623695 1.039624908 0.79405885 0.282788684 31282249.18 41921159.77 40392925.44 45691368.82 48568109.62 36324131.17 17619216.24 57325615.62 44208481.9 42753542.45 49382847.95 42565847.74 57001197.5 55770647.25 55774922.15

297POS PC(32:0e)+H PC(32:0e)+H PC (32:0e) (32:0e) C40 H83 O7 N1 P1 720.5901685 10.8450696 0.002292097 0.821877899 0.552461652 2.632224839 1140868492 850313418 1227894599 1264181375 1614545480 2049180801 434703254.2 1031793619 1112751378 643436082.5 663056606.9 2810085285 1620135560 1626909925 1620862068

298POS PC(30:3)+Na PC(30:3)+Na PC (30:3) (30:3) C38 H70 O8 N1 P1 Na1 722.4731285 8.173270835 0.007515429 0.882678784 0.699861225 0.19655942 8050984.652 4660896.044 9508530.996 5055970.824 3178002.669 6573043.226 2548215.503 8081183.708 9259123.874 1190170.257 2315025.79 9289606.34 8595715.65 8709628.412 8707514.601

299POS PE(16:1p/20:4)+H PE(36:5p)+H PE (16:1p/20:4) (16:1p) (20:4) C41 H73 O7 N1 P1 722.5119185 9.703289233 0.004542422 1.174422097 0.216781832 1.774128433 512297794.9 426214699.7 582298945.5 464143180.4 583901632.1 571399019.4 325809098.8 703358655.7 558566298.8 651198909.7 751024779.7 698027439.4 753238002.9 753216799.6 759169126.9

29POS AcCa(18:0)+H AcCa(18:0)+H AcCa (18:0) (18:0) C25 H50 O4 N1 428.3734355 3.64452248 0.007254465 0.980878115 0.955019244 0.346600982 25476323.66 173186578.9 28747213.35 112059456 103888831.9 119997188.6 34324350.03 107588516.6 115601846.7 61223202.27 60926106.98 172919148.9 128771022.4 130398923.5 130406473.6

2POS So(d16:0)+H So(d16:0)+H So (d16:0) (d16:0) C16 H36 O2 N1 274.2740555 1.688 0.009053572 0.086450696 0.325820809 0.734875747 3457691.356 184013912.5 4440035.247 3443450.337 4235587.222 4106721.179 1017179.475 3612991.175 3089570.951 2828784.12 3672302.028 3388954.059 27164058.52 27165819.96 26741176.9

300POS DG(18:1/24:1)+NH4 DG(42:2)+NH4 DG (18:1/24:1) (18:1) (24:1) C45 H88 O5 N1 722.6657005 13.49116742 0.004156919 1.04911249 0.896172177 0.233197799 17999895.87 16651817.32 20112995.35 29398727.89 15262221.54 16448812.83 2904213.492 44396281.69 28806635.2 8193867.944 7169762.994 30094593.25 32245982.84 32507588.08 32432243.7

301POS SM(d34:2)+Na SM(d34:2)+Na SM (d34:2) (d34:2) C39 H77 O6 N2 P1 Na1 723.5411475 8.603998178 0.004298759 1.020641524 0.860486928 0.824334635 306670517.7 320417892.5 270655236.2 226666426.1 335042713.4 201079094.6 270733271.5 265063687.1 294543897.2 344506231 331340128.4 188620573.7 259838763.8 259827506.7 257903318

302POS PC(32:5)+H PC(32:5)+H PC (32:5) (32:5) C40 H71 O8 N1 P1 724.4911835 8.264875043 0.00638331 1.012316798 0.964331799 0.277335163 29535389.13 18852432.13 34456146.38 19479316.53 17752281.99 34053894.84 8698361.358 28143579.06 38763724.55 16652613 16150698.5 47618865.92 38491796.61 38492645.94 38919373.24

303POS PC(32:5)+H PC(32:5)+H PC (32:5) (32:5) C40 H71 O8 N1 P1 724.4911835 7.7284595 0.004589416 0.837011493 0.714713606 0.33321673 22807946.6 5989350.336 25438057.02 11996141.41 4064270.516 17711752.45 1858964.835 17613733.22 17175983.71 1676079.658 1947527.722 33391015.2 17146376.84 17283695.98 17283104.64

304POS PE(34:2e)+Na PE(34:2e)+Na PE (34:2e) (34:2e) C39 H76 O7 N1 P1 Na1 724.5251635 11.03517542 0.008144555 0.996155356 0.965996566 0.611738857 96926145.86 105446330.2 104486840 120733200.7 142501408.7 113679194.3 94296252.44 114374160 96245990.33 132813842.9 137932228.8 105481781.4 111922496.7 112543364.1 113729557.6

305POS PE(16:0p/20:4)+H PE(36:4p)+H PE (16:0p/20:4) (16:0p) (20:4) C41 H75 O7 N1 P1 724.5275685 10.26991074 0.004490282 1.178177847 0.232675705 3.42784543 1758817069 1531190194 1819232614 1956569868 2399002718 2003645600 1086489267 2693848942 2261272001 2457833877 2559915828 2452523319 2649747545 2670477916 2649884493

306POS DG(26:1/16:0)+NH4 DG(42:1)+NH4 DG (26:1/16:0) (26:1) (16:0) C45 H90 O5 N1 724.6813505 14.01705832 0.023682596 1.180884359 0.681424405 0.279489578 28165802.51 26117003.14 16638217.38 21691870.46 15626705.96 13360041.52 5403252.225 53080470.8 44556049.27 9180158.507 8511686.464 22863496.85 41036521.47 42750072.29 41049884.98

307POS SM(d34:1)+Na SM(d34:1)+Na SM (d34:1) (d34:1) C39 H79 O6 N2 P1 Na1 725.5567975 9.48662391 0.001460305 0.954031418 0.603119768 0.655090304 214050254.6 237170474.7 173951530.2 187582292.9 220033000.8 154426192.7 239429592.4 164442014.9 174978201.1 199298285.9 180746757 173744362.4 170319995.7 169890152.1 169889701.6

308POS PC(32:4)+H PC(32:4)+H PC (32:4) (32:4) C40 H73 O8 N1 P1 726.5068335 8.220947468 0.107502121 0.958461699 0.892475588 0.33692129 22613840.21 17911121.1 25546194.38 16084971.2 9260356.788 19374425.34 7994908.574 23303839.38 29132992.65 5469451.104 7080280.926 33207370.21 25960548.93 31125070.44 25978643.65

309POS PE(16:0p/20:3)+H PE(36:3p)+H PE (16:0p/20:3) (16:0p) (20:3) C41 H77 O7 N1 P1 726.5432185 10.61421881 0.005567521 0.989427443 0.912661652 0.375815558 159457396.3 136559484.7 132784314.1 146620108.2 155654290.7 178468689 106771409.5 162321752.9 179632595.7 139342580.2 127626589.1 184233146.4 175500243.6 173812018.2 175497698.6

30POS AcCa(20:4)+H AcCa(20:4)+H AcCa (20:4) (20:4) C27 H46 O4 N1 448.3421355 2.188 0.003032491 1.258686451 0.647207867 0.406441282 19096306.82 41895264.09 27231174.3 64090492.81 36390402.06 18384706.72 670951.1474 66800239.95 67928829.87 11535317.93 7322930.193 106401027.2 55604520.93 55897199.71 55898010.54

310POS PE(16:0p/20:3)+H PE(36:3p)+H PE (16:0p/20:3) (16:0p) (20:3) C41 H77 O7 N1 P1 726.5432185 10.865 0.003801129 1.02811596 0.867037268 0.055916033 96740976.55 80307507.59 112463685.7 121063086.1 139369744.3 120780425.8 57683160.79 155814881 120550994.3 93971810.82 99352923.06 162209745.3 154600736.5 153588749.1 153585921.1

311POS DG(26:0/16:0)+NH4 DG(42:0)+NH4 DG (26:0/16:0) (26:0) (16:0) C45 H92 O5 N1 726.6970005 14.65553046 0.005748623 0.850821629 0.681822938 0.242481568 11018869.69 6696850.153 10538166.97 9172432.62 2418484.815 5740672.724 1694680.573 10396507.06 12392549.17 1175565.686 1042483.666 12083323.61 11288044.72 11288977.42 11401281.11

312POS SM(d34:0)+Na SM(d34:0)+Na SM (d34:0) (d34:0) C39 H81 O6 N2 P1 Na1 727.5724475 9.83142037 0.013931999 0.827879404 0.166148974 0.7422078 139703326.5 151531560.8 115812718.4 117918022.1 131494027.6 83774098.65 124722855.4 90456326.63 91005348.68 128384285.5 116382352.7 61873109.93 88100057.9 86032912.56 85983509.76

313POS PC(30:0)+Na PC(30:0)+Na PC (30:0) (30:0) C38 H76 O8 N1 P1 Na1 728.5200785 9.48992238 0.079069916 0.832540835 0.04016542 0.566057121 77346214.37 84050896.07 61421861.25 70721659.23 66325261.81 69907989.79 76293103.27 58716488.92 63051404.6 50087255.16 51184202.23 58471852.76 65831910.56 57551517.57 57589251.69

314POS PC(32:3)+H PC(32:3)+H PC (32:3) (32:3) C40 H75 O8 N1 P1 728.5224835 8.389638843 0.0023398 0.917339607 0.763891291 0.933578956 212112224.7 156044269.4 237421628.2 147620536.8 115237172.1 231171069 59725502.21 162587503.6 252936248.1 94417970.1 97333168.13 341712569.1 268690517.6 269784766.3 269779971.4

315POS PE(35:3)+H PE(35:3)+H PE (35:3) (35:3) C40 H75 O8 N1 P1 728.5224835 9.809 0.054336517 0.84234843 0.233923335 0.310094039 41205498.26 34070968.85 38478194.49 55887443.47 27433431.37 27414942.06 38686190.18 30326737.27 33931563.77 28736586.85 29298873.5 28119250.52 32509417.87 29582403.59 29680918.49

316POS PE(18:1p/18:1)+H PE(36:2p)+H PE (18:1p/18:1) (18:1p) (18:1) C41 H79 O7 N1 P1 728.5588685 11.05103633 0.289338265 1.038470215 0.732185044 0.930670611 475260131.9 528028004 507393912.3 524542394.7 566402532.8 526911299.9 273153988.1 585697589.7 550945176 574976678.3 605170382.9 658950001.5 583306191.1 584741048.7 333237753.3

317POS SM(d36:2)+H SM(d36:2)+H SM (d36:2) (d36:2) C41 H82 O6 N2 P1 729.5905025 9.613249577 0.01003175 1.198834494 0.157790581 0.695693903 80882133.92 86318377.29 87551020.48 70911000.54 92672793.22 77159291.32 53641418.37 124332636.2 114836404.8 98141585.85 111463407.9 91600585 110310983.6 112235433.1 112264544.9

318POS PC(32:2)+H PC(32:2)+H PC (32:2) (32:2) C40 H77 O8 N1 P1 730.5381335 9.317364222 0.002257863 1.036409853 0.859710395 1.212009003 1193543961 703806529.4 1201576622 761406435.4 710387518.1 1114251923 577489472.2 988122486.3 1304631518 700708127.5 729352165.5 1591658251 1314714527 1320525280 1318734939

319POS PC(32:2)+H PC(32:2)+H PC (16:1/16:1) (16:1) (16:1) C40 H77 O8 N1 P1 730.5381335 8.796657103 0.007502563 1.015623487 0.949166615 2.831799244 5613103798 3724088215 5990802313 4989902726 5685173519 7606832583 1986811454 6250972467 7336459691 3830943395 4211786060 10518033966 8499905402 8611225230 8611425310

31POS AcCa(20:3)+H AcCa(20:3)+H AcCa (20:3) (20:3) C27 H48 O4 N1 450.3577855 2.626 0.169006173 1.128998685 0.822806618 0.147482929 3516189.71 8953196.207 4738120.959 16106684.25 5962712.68 2906545.867 95955.22548 11645903.78 10185707.65 2007877.337 1868168.263 21821446.94 10288749.99 13204428.5 9715769.13

320POS PE(35:2)+H PE(35:2)+H PE (35:2) (35:2) C40 H77 O8 N1 P1 730.5381335 10.32042151 0.003288461 1.013182022 0.893214418 0.655987585 290181188.9 296140397.2 340514310.2 333426978.9 365004745.2 316078836.4 195625320.5 401930982.7 354709148.4 317908485.7 340479078.3 356284312.4 392341474.1 394579535.3 394589796

322POS PE(18:0p/18:1)+H PE(36:1p)+H PE (18:0p/18:1) (18:0p) (18:1) C41 H81 O7 N1 P1 730.5745185 11.67232002 0.005267052 1.102617887 0.607638519 1.028260875 537751153 578974177.8 678173494.3 859959409 914026698.1 699781494 284283811.1 1159329884 825997703.7 703043077 718086747.9 1015966733 1069826814 1079637819 1069869398

323POS SM(d36:1)+H SM(d36:1)+H SM (d36:1) (d36:1) C41 H84 O6 N2 P1 731.6061525 10.36345319 0.009174299 1.027516974 0.731760027 0.186811952 261619467.4 226625978.6 267506506.8 189601341.5 216034595.4 207176235 201816460.8 260488445 261109255.2 213501463.2 204051305.3 265255938.1 250647801.3 254659489.6 250662799.6

324POS PC(32:4p)+Na PC(32:4p)+Na PC (32:4p) (32:4p) C40 H72 O7 N1 P1 Na1 732.4938635 7.419433623 0.016156021 3.519617851 0.002454137 0.295120113 1241559.199 826845.7008 823733.7042 812128.652 1000624.862 536551.1792 490790.5645 3836726.855 4123943.224 2984991.218 3514921.771 3496503.763 3037531.017 3134856.852 3068166.502

325POS PC(16:0/16:1)+H PC(32:1)+H PC (16:0/16:1) (16:0) (16:1) C40 H79 O8 N1 P1 732.5537835 10.97786614 0.030583391 1.088122221 0.647509787 0.609489714 353975392.7 305706552.6 560746340.6 343673260.9 328739399.8 326189198.7 217154815.3 483201134.1 440862849.1 319466069.1 339060759.7 614830383.4 536047421.9 536830436.3 565357846.3

326POS PC(16:0/16:1)+H PC(32:1)+H PC (16:0/16:1) (16:0) (16:1) C40 H79 O8 N1 P1 732.5537835 11.93507646 0.061938989 1.069111671 0.831195149 0.107744683 22765883.73 13372447.78 33397469.43 15861952 16473253.56 20836007.42 7458458.926 17195934.99 46411315.18 28654464.34 11348833.5 20118493.75 33240375.53 29716064.98 30147914.38

327POS PC(32:1)+H PC(32:1)+H PC (32:1) (32:1) C40 H79 O8 N1 P1 732.5537835 9.585976402 0.00700304 0.909584771 0.415562165 6.619710609 20550916304 17334117589 26585201302 19352321890 17645147673 22525006604 14155698091 19638095689 22715618933 16617578396 15341298213 24313592661 21365974126 21627867267 21626624376

328POS PC(32:1)+H PC(32:1)+H PC (32:1) (32:1) C40 H79 O8 N1 P1 732.5537835 13.745 0.026221134 0.927646542 0.725985204 0.041250004 5892339.178 4152351.288 10619854.95 8584755.682 8280488.102 8927259.733 2253184.452 9595994.313 8308846.728 6450732.692 5868233.431 10618729.19 15096407.67 14404257.34 14461154.68

329POS PC(33:0p)+H PC(33:0p)+H PC (33:0p) (33:0p) C41 H83 O7 N1 P1 732.5901685 10.54086478 0.033259834 0.8555891 0.487501916 0.961117519 132114244.3 117270904.2 132986444.3 137759485.3 161711578.7 170488255.7 64407294.27 125292454.2 156207283.2 74246338.13 72718488.77 236373179.4 168824837.8 168694638.6 159221695.5

32POS LPE(16:1)+H LPE(16:1)+H LPE (16:1) (16:1) C21 H43 O7 N1 P1 452.2771685 2.159020278 0.00431632 0.985257855 0.940479884 0.031071873 5435044.719 3959733.991 6631835.497 4600813.959 8043042.507 7220449.258 2904787.75 7793724.732 5195621.502 4415940.134 5688219.441 9363517.241 8274802.87 8336751.964 8274666.935

330POS PC(33:0p)+H PC(33:0p)+H PC (33:0p) (33:0p) C41 H83 O7 N1 P1 732.5901685 12.21710289 0.020782976 1.01735534 0.92281149 0.076419822 7407736.611 6318619.715 8943299.842 9962666.646 10514922.5 9914355.607 3330075.571 13616634.57 8512879.98 8399897.08 9160136.455 10962879.4 13354482.75 13841626.95 13355608.37

331POS SM(d36:0)+H SM(d36:0)+H SM (d36:0) (d36:0) C41 H86 O6 N2 P1 733.6218025 10.67354498 0.003647081 0.897660075 0.369372861 0.458971904 81358888.27 66745727.24 82406386.79 67550004.11 67829360.47 59248443.65 53475453.71 80878707.28 79412328.69 43949235.3 49165265.95 74749145.48 79052067.14 79554964.53 79552114.08

332POS PS(32:1)+H PS(32:1)+H PS (32:1) (32:1) C38 H73 O10 N1 P1 734.4966635 8.727 0.039499942 0.820820566 0.634965694 0.266582723 13195092.93 10773208.79 15937617.98 21770236.32 9869503.169 43833467.94 10481323.51 16083763.2 12855211.24 8579382.811 8117210.063 38588669.57 15737456.63 14698030.79 15765298.87

333POS PC(32:4e)+Na PC(32:4e)+Na PC (32:4e) (32:4e) C40 H74 O7 N1 P1 Na1 734.5095135 8.217763048 0.001981125 2.958350555 0.000529198 0.489956115 3101680.432 2357387.602 3415147.342 3435817.372 2999183.366 2211948.033 3179438.571 9954430.173 9061964.853 10004099.77 10641579.56 8992232.758 8119820.772 8138979.056 8107007.067

334POS PC(16:0/16:0)+H PC(32:0)+H PC (16:0/16:0) (16:0) (16:0) C40 H81 O8 N1 P1 734.5694335 10.33210726 0.004250686 0.945269888 0.579642191 4.616644227 8578420518 8420252984 8505198378 8219750376 10345533232 8918855608 4711845562 8182955975 8250040250 9510846259 9808581037 9623702251 9617638383 9617456108 9688529541

335POS PC(33:0e)+H PC(33:0e)+H PC (33:0e) (33:0e) C41 H85 O7 N1 P1 734.6058185 11.20770069 0.035023799 1.181895222 0.661784145 0.300765417 44591667.95 36912324.28 45970771.33 47080222.16 65216626.88 70097666.16 32361520.3 75935224.9 49622069.68 30034160.16 24062524.29 154217520.8 79382652.45 84085315.09 79003899.01

336POS SM(d37:6)+H SM(d37:6)+H SM (d37:6) (d37:6) C42 H76 O6 N2 P1 735.5435525 15.11885896 0.001278007 1.433482268 0.028837997 0.29947505 27015918.41 11922856.14 26395883.2 11184359.97 28592468.68 30566341.97 36427568.03 29801323.65 33251779.87 28344582.41 29895718.95 36770788.26 29914306.8 29980613.66 29914387.59

337POS PE(16:1/20:5)+H PE(36:6)+H PE (16:1/20:5) (16:1) (20:5) C41 H71 O8 N1 P1 736.4911835 8.370459772 0.022028174 0.984792534 0.94225667 0.175454572 26342077.74 18585615.97 31919832.9 20936439.25 21154953.38 30636468.92 11995545.36 22376372.82 29799341.92 19426711.39 19503325.22 44199428.86 36487071.22 36494697.56 37901073.86

338POS PE(14:0/22:6)+H PE(36:6)+H PE (14:0/22:6) (14:0) (22:6) C41 H71 O8 N1 P1 736.4911835 8.65510958 0.010233295 0.949578681 0.78218156 0.159098659 11975367.79 10143402.6 11999452.1 12029149.58 8830448.242 10013105.53 4598196.631 14201303.91 15099944.43 6680271.564 7596014.499 13538266.6 16456299.49 16753888.74 16464679.83

339POS PC(34:6e)+H PC(34:6e)+H PC (34:6e) (34:6e) C42 H75 O7 N1 P1 736.5275685 7.902981543 0.025649749 3.253863423 0.002313759 0.411284627 2467782.85 2184308.963 2424596.28 1701667.416 1575617.56 1654001.168 2215412.201 7084417.297 9850201.095 5336331.168 5912842.309 8673104.09 6115213.748 6397920.537 6392363.052

33POS LPC(14:0p)+H LPC(14:0p)+H LPC (14:0p) (14:0p) C22 H47 O6 N1 P1 452.3135535 2.179074555 0.007898725 1.099263918 0.657504802 0.082908295 3640297.078 3404922.249 4894656.467 3856223.49 4333138.409 3393484.282 1949596.737 5914609.998 6353151.959 2896174.734 2685263.614 6058882.467 5970432.023 6053464.291 6052252.856

340POS SM(d35:2)+Na SM(d35:2)+Na SM (d35:2) (d35:2) C40 H79 O6 N2 P1 Na1 737.5567975 9.13738 0.003879758 1.157182012 0.309834027 0.22953902 9942596.674 11123652.09 11644391.11 9843400.048 13898422.41 8080964.943 7546846.042 16034361.78 13346296.53 15133015.38 13303880.94 9312520.572 12395039.39 12327747.56 12420775.56

341POS SM(d37:5)+H SM(d37:5)+H SM (d37:5) (d37:5) C42 H78 O6 N2 P1 737.5592025 15.40835098 0.009392684 1.638059813 0.064623407 0.163043509 6547373.991 3020477.649 6722070.831 2571798.097 8462030.551 12682088.88 13559378.16 8705519.248 12400284.94 7512276.265 7825477.081 15529023.1 9751795.338 9595291.972 9594410.531

342POS PC(33:5)+H PC(33:5)+H PC (33:5) (33:5) C41 H73 O8 N1 P1 738.5068335 9.053571762 0.022204892 1.060916084 0.75734862 0.285614456 151287543.6 78797199.81 168325537.7 91940081.73 90352109.77 126467645.5 90651705.1 131273218.4 142964961 88492434.87 100116070.2 196749762.9 187775005.7 180740598.6 180726855.9

343POS PE(34:2)+Na PE(34:2)+Na PE (34:2) (34:2) C39 H74 O8 N1 P1 Na1 738.5044285 9.929002653 0.012384597 0.919137063 0.212983916 0.418027289 50417479 57855972.25 53921002.25 59717863.53 68366014.96 59670045.31 49799284.68 54241736.32 44005903.51 53482645.44 59993146.84 60127806.96 58424073.59 57171361.12 58406787.37

344POS PC(33:5)+H PC(33:5)+H PC (33:5) (33:5) C41 H73 O8 N1 P1 738.5068335 8.303974523 0.022209412 1.02851976 0.930790051 0.19904505 11784394.56 21215856.49 11475642.03 18232423.06 12483202.89 26588609.82 5952885.027 17259675.95 22895283.72 10389971 10023334.65 38161723.31 25538434.32 26535130.68 25541381.18

345POS PE(16:0/20:5)+H PE(36:5)+H PE (16:0/20:5) (16:0) (20:5) C41 H73 O8 N1 P1 738.5068335 9.051640723 0.022309122 1.09459602 0.601099964 0.387784887 120583861 93278654.56 133547353.3 123494236.3 121576550.3 152156715.3 53666421.47 155387979.4 141477161.7 122352419.7 135694787.1 206498333.3 184238040.3 191530711.3 191559869

346POS PE(35:1p)+Na PE(35:1p)+Na PE (35:1p) (35:1p) C40 H78 O7 N1 P1 Na1 738.5408135 10.684 0.058096283 1.279209422 0.243541423 0.88850304 138751153.2 82131663.13 97418246.26 143477391.1 205189304.1 93708884.07 105297773.1 220790453.3 111129880.2 222389160.5 134419480.8 179037979.5 164687471.4 148904725.2 149422379.5

347POS SM(d34:2+hO)+Na SM(d34:2+hO)+Na SM (d34:2+hO) (d34:2+hO) C39 H77 O7 N2 P1 Na1 739.5360625 8.236591273 0.02247777 1.229744722 0.45352127 0.202725298 8594315.363 6132898.966 9280101.551 8968634.459 7868774.872 10434698.91 3584664.49 15615722.86 16079747.1 7119465.205 4718588.518 15942412.99 14329736.73 14323536.13 13776034.31

348POS PC(31:1)+Na PC(31:1)+Na PC (31:1) (31:1) C39 H76 O8 N1 P1 Na1 740.5200785 9.125447373 0.021438577 0.912599803 0.28827255 0.71364261 239959359.2 207047527.6 209599935.2 233073053.2 203475432.6 218456222.2 122502383.9 219295477.3 229658398 205523024.8 202559819 217437321.5 237502674.2 227886042.1 230496544.4

349POS PE(34:1)+Na PE(34:1)+Na PE (34:1) (34:1) C39 H76 O8 N1 P1 Na1 740.5200785 10.64382354 0.015056503 0.91916303 0.231643952 0.343040918 72287644.05 72890776.21 92898268.92 84551157.75 67128905.7 77187004.96 82445567.47 68495686.92 76933125.03 62034821.24 65460472.2 73827766.2 72123106.54 74034880.04 74039625.21

34POS AcCa(20:2)+H AcCa(20:2)+H AcCa (20:2) (20:2) C27 H50 O4 N1 452.3734355 2.93 0.002204293 1.167349256 0.664037075 0.043973369 648308.5003 4670766.714 788961.5003 4439108.491 2589733.283 1844918.215 523055.0261 4952711.236 3312525.499 2721564.086 2353314.773 3625818.614 3891533.208 3906378.566 3906478.508

350POS PE(18:1/18:3)+H PE(36:4)+H PE (18:1/18:3) (18:1) (18:3) C41 H75 O8 N1 P1 740.5224835 9.448598467 0.005693716 0.871270529 0.289970625 0.510143041 67130422.05 53233541.41 64444392.03 51680999.87 45538350.47 54663429.78 39466649.73 54101600.59 61431782.6 37250701.35 34627429.97 66470899.44 66221912 66222027.04 66877191.6

351POS PE(16:0/18:1)+Na PE(34:1)+Na PE (16:0/18:1) (16:0) (18:1) C39 H76 O8 N1 P1 Na1 740.5200785 9.861011205 0.017578646 1.0640381 0.525871477 0.26261102 622846251.1 435694022.4 664997234.8 437365660.4 438959415.7 521270566.4 505402461.8 631766351.7 533994354.1 478424532.9 520771917.5 650644968.1 644958281.4 665052976.2 664951126.4

352POS PC(16:0p/16:0)+Na PC(32:0p)+Na PC (16:0p/16:0) (16:0p) (16:0) C40 H80 O7 N1 P1 Na1 740.5564635 10.17411632 0.021984914 0.744004644 0.114711065 0.516423763 31107966.01 27556113.95 17733622.19 32460414.3 31446417.13 38673425.56 28260519.08 12345767.53 23488797.04 19139430.88 15346215.5 34579702.68 26928432.62 26950395.74 25926622.52

353POS SM(d34:1+pO)+Na SM(d34:1+pO)+Na SM (d34:1+pO) (d34:1+pO) C39 H79 O7 N2 P1 Na1 741.5517125 8.020636937 0.010742691 1.262239222 0.468506344 0.176041864 5308139.162 3860311.887 6684272.565 7005757.764 5566924.865 5945076.783 1214693.415 10661563.39 9799923.539 4071264.467 4161915.858 13474411.08 10296917.38 10488298.09 10493433.54

354POS PC(33:3)+H PC(33:3)+H PC (33:3) (33:3) C41 H77 O8 N1 P1 742.5381335 8.643550992 0.007260264 0.937464499 0.776989969 0.410876547 73531926.17 40540735.97 65495086.67 50737381.12 44311925.26 71806756.59 20177232.94 63475583.73 76612582.46 36120686.75 38560790.07 89813149.27 76404066.23 77352998.15 76373379.67

355POS PE(18:1/18:2)+H PE(36:3)+H PE (18:1/18:2) (18:1) (18:2) C41 H77 O8 N1 P1 742.5381335 10.49005192 0.021196559 0.952500024 0.870074424 0.29512168 49696846.07 29294160.13 38990111.87 4603696.271 92978913.5 44814679.36 44466715.64 48985363.4 38119861.44 35879571.87 30469460.14 50089466.5 49427952.67 47672658.41 47641021.11

356POS PC(31:0)+Na PC(31:0)+Na PC (31:0) (31:0) C39 H78 O8 N1 P1 Na1 742.5357285 10.083 0.048554657 1.080900536 0.558427298 0.965409264 555901993.2 536890490.9 651296336.1 601089296 644511655.3 660756525.5 345208947.9 848650036.5 739488667.9 557564710 617958247.4 836898747.9 921682053.5 922402978.8 1001819439

357POS PC(33:3)+H PC(33:3)+H PC (33:3) (33:3) C41 H77 O8 N1 P1 742.5381335 8.923522018 0.036548849 0.890633736 0.589182884 0.535754518 95470087.56 59175952.21 87207827.56 63359483.4 57865727.92 92233257.33 28010912.94 77700505.8 93320382.67 40767585.37 54729344.86 110987795.3 95752357.01 89952318.43 89920332.11

358POS LPC(32:0)+Na LPC(32:0)+Na LPC (32:0) (32:0) C40 H82 O7 N1 P1 Na1 742.5721135 10.87442061 0.00518869 0.706251259 0.041378564 0.791243306 55676634.67 47584287.16 51591258.87 55160426.55 69090543.68 79817850.65 46035791.48 33165238.07 33764214.86 39089190.84 32994861.09 68439112.75 49339543.73 48899822.53 48897717.82

359POS ChE(24:6)+NH4 ChE(24:6)+NH4 ChE (24:6) (24:6) C51 H84 O2 N1 742.6496555 15.57322402 0.007048176 1.934334762 0.088211661 0.410361651 9779656.239 2794551.909 9209235.259 5829851.802 9325833.198 13082370.46 9741153.841 25048394.75 26934121.86 6971225.284 6382462.743 21680965.65 19745953.28 19987364.84 19990574.8

35POS LPE(16:0)+H LPE(16:0)+H LPE (16:0) (16:0) C21 H45 O7 N1 P1 454.2928185 2.811134052 0.005545193 1.262669962 0.101568958 0.198177391 14904941.85 10513375.38 20191661.2 9902969.301 14204010.92 14899934.38 17119779.31 20366920.74 14384482.31 14266574.48 17751900.01 22953552.29 22021169.42 22233508.5 22021479.21

360POS PC(33:2)+H PC(33:2)+H PC (33:2) (33:2) C41 H79 O8 N1 P1 744.5537835 9.233767878 0.004251945 1.09331192 0.640942654 1.263879013 2187515331 1567461258 2164037854 2062353705 2317963774 2026754050 877890701.8 2471866813 2794663250 1791190873 1919006195 3621638890 3046765765 3072164405 3064405274

361POS PE(36:2)+H PE(36:2)+H PE (18:1/18:1) (18:1) (18:1) C41 H79 O8 N1 P1 744.5537835 10.70024403 0.004087863 1.083764286 0.509688389 4.125294614 4317461172 4560130902 4910003261 5411151612 5952910762 5056590325 2741765884 6636601205 5421033399 5804295176 6213011646 5921913052 6557134648 6557703875 6603955545

362POS PC(33:2)+H PC(33:2)+H PC (33:2) (33:2) C41 H79 O8 N1 P1 744.5537835 9.811 0.028891062 0.878173445 0.09333744 0.527276903 95199832.23 78513352.34 92979390.5 91796085.08 76726711.18 85381134.27 61661404.13 67655567.2 91337816.89 81619271.63 69618284.96 85281682.1 102348849.3 97330576.18 102389229.2

363POS PC(34:2e)+H PC(34:2e)+H PC (34:2e) (34:2e) C42 H83 O7 N1 P1 744.5901685 10.77883035 0.008155018 1.131284014 0.499567632 2.252364348 1488718228 1722485892 1471742530 1842424231 2844245058 1747658350 719732303.3 2533047044 2413745279 2241692423 2272987078 2395590550 2431327838 2466143596 2431949505

364POS ChE(24:5)+NH4 ChE(24:5)+NH4 ChE (24:5) (24:5) C51 H86 O2 N1 744.6653055 16.11752724 0.019613429 1.698801076 0.18810207 0.278313849 11524848.09 2640997.322 10310759.31 4302743.161 6349972.758 16744205.09 13525625.69 17258617.75 22432804.84 4504821.976 4253372.257 26147558.79 18707872.55 19351611.49 18709667.09

365POS PC(33:1)+H PC(33:1)+H PC (33:1) (33:1) C41 H81 O8 N1 P1 746.5694335 10.01285898 0.006605694 1.041714151 0.706048536 0.784686359 8248345686 7209927471 8087964382 7353700423 7826217013 7936115267 4853121676 9802369924 9712255748 7213224950 7301079210 9726695739 9596221636 9706211731 9707496459

366POS PC(33:1)+H PC(33:1)+H PC (33:1) (33:1) C41 H81 O8 N1 P1 746.5694335 11.33172472 0.001568676 1.093148058 0.518773713 2.912939071 3604236323 3173153925 4142472470 3993628513 3442779758 3354669240 1954924340 5468673484 4354958623 3616600293 3816888235 4521227172 5219614688 5212586970 5228912583

367POS PC(34:1e)+H PC(34:1e)+H PC (34:1e) (34:1e) C42 H85 O7 N1 P1 746.6058185 11.20837919 0.006693747 1.183094095 0.510137949 0.288234108 76294784.3 97689001.36 67880794.86 93574131.58 176019242.2 130033799.3 34550629.68 144124358.5 161121608.2 107516941.1 108021505.1 203610063.2 164944151.4 166288611.9 167150679.2

368POS PC(34:1e)+H PC(34:1e)+H PC (34:1e) (34:1e) C42 H85 O7 N1 P1 746.6058185 10.89332569 0.004855333 0.970522699 0.909993295 2.597887813 2708940262 2140221098 2656928448 2849256702 3533562146 4109744738 1096015569 3731742653 3497985341 1665989197 1771173535 5705195381 3966544099 4000660264 3999487924

369POS ChE(24:4)+NH4 ChE(24:4)+NH4 ChE (24:4) (24:4) C51 H88 O2 N1 746.6809555 16.71210755 0.010061867 1.707465742 0.25602102 0.113198731 2555609.548 373728.5134 2350190.946 507986.3529 1197475.258 4325591.736 3452817.796 3142214.231 4069966.382 847381.5879 887753.1771 6912298.719 4340774.702 4416513.714 4340086.297

36POS AcCa(20:0)+H AcCa(20:0)+H AcCa (20:0) (20:0) C27 H54 O4 N1 456.4047355 5.149 0.003956605 0.896258472 0.727748875 0.074604098 511826.3536 2521523.79 519749.533 1955357.593 3379801.065 1896684.382 1114791.632 2164413.091 2176783.261 1010223.916 1073084.856 2126799.528 2354921.526 2371124.243 2371143.679

370POS PC(32:4)+Na PC(32:4)+Na PC (32:4) (32:4) C40 H72 O8 N1 P1 Na1 748.4887785 8.220947468 0.024973698 0.942363222 0.815801933 0.13229446 7389733.938 3757448.131 7400798.458 4943466.068 3650771.989 5804462.195 1986898.52 7026110.546 8033221.994 2256517.838 3970081.927 7774909.441 7513213.89 7844919.845 7517004.848

371POS PE(18:1p/20:5)+H PE(38:6p)+H PE (18:1p/20:5) (18:1p) (20:5) C43 H75 O7 N1 P1 748.5275685 9.761266222 0.010554472 1.280483947 0.093394191 1.560668927 221039329.1 196395433.4 255820561.6 207245167.1 306950762.6 257558268.4 164271219.6 347725232.5 281511086.2 355017387.1 387068478.7 314718091.9 340583679.2 340591893.7 346852200.3

372POS PC(33:0)+H PC(33:0)+H PC (33:0) (33:0) C41 H83 O8 N1 P1 748.5850835 10.59554164 0.002111835 1.038364182 0.788215051 0.291167646 826777085.2 973825745.9 800005962.5 835985173.3 977511360.7 736132038 412615386.4 999020240.9 1062926380 868185686.5 797084905.6 1207989410 1041914212 1046237450 1044833614

373POS PC(34:0e)+H PC(34:0e)+H PC (16:0e/18:0) (16:0e) (18:0) C42 H87 O7 N1 P1 748.6214685 11.51963678 0.011668041 0.87909394 0.729660554 1.532390358 427889202.3 314300892.9 426306799.5 470555261.6 647868096.6 755729227.4 121482218.5 475790822.8 529407972.5 188937386.5 194044786.4 1165111533 686286849.3 686372869 700294211.7

374POS PC(32:3)+Na PC(32:3)+Na PC (32:3) (32:3) C40 H74 O8 N1 P1 Na1 750.5044285 8.389638843 0.00941856 0.822033466 0.409412725 0.379943105 27698861.25 52540694.43 28415925.78 21677429.35 20185641.08 37501351.49 16224244.15 23945777.17 26257171.54 20551699.73 21934987.87 45644772.33 33088390.01 33086880.13 33630357.35

375POS PC(32:3)+Na PC(32:3)+Na PC (32:3) (32:3) C40 H74 O8 N1 P1 Na1 750.5044285 8.872 0.01181544 1.139689582 0.431184595 0.226582748 14942970.45 12269413.92 18406970.96 9588214.663 13105150.26 12467580.98 8155228.889 14720779.89 16092196.12 14456927.08 23094023.02 15545312.73 18262658.15 18640570.36 18642565.74

376POS PE(18:0p/20:5)+H PE(38:5p)+H PE (18:0p/20:5) (18:0p) (20:5) C43 H77 O7 N1 P1 750.5432185 10.54371572 0.004522108 1.071516207 0.524373951 0.93339132 478490919.8 418861083.5 511540723.8 425185361.1 548133691.1 515237865.5 295387202 601507117.4 540232834.3 495264458.2 555877887.9 616394752.2 622498740.4 622564675.7 627420129.5

377POS PE(18:1p/20:4)+H PE(38:5p)+H PE (18:1p/20:4) (18:1p) (20:4) C43 H77 O7 N1 P1 750.5432185 10.28707255 0.005241266 1.057194361 0.682774582 0.645638393 1198199247 882125184.5 1244843545 1080829212 1227070351 1459278789 765473196 1270943702 1199771266 1190877610 1229885667 1841037101 1494047833 1507709478 1507677650

378POS DG(26:1/18:1)+NH4 DG(44:2)+NH4 DG (26:1/18:1) (26:1) (18:1) C47 H92 O5 N1 750.6970005 14.02290895 0.006281766 0.907817761 0.790400101 0.269898655 16669487.45 16916129.57 16447456.67 22968466.76 9208121.985 11855282.35 3314695.26 29723203.27 22630758.47 3693520.311 3419317.886 22612332.39 24633467.14 24902992.54 24634517.48

379POS PC(32:2)+Na PC(32:2)+Na PC (32:2) (32:2) C40 H76 O8 N1 P1 Na1 752.5200785 9.317364222 0.043568928 1.192832032 0.483297381 0.5462894 77877572.7 58515972 77127349.86 55826068.42 56055956.8 72096064.36 90435123.85 46640832.74 49749340.52 157811066.6 68436089.55 61077067.62 59111849.99 57644732.68 62710209.1

37POS LPE(16:0p)+Na LPE(16:0p)+Na LPE (16:0p) (16:0p) C21 H44 O6 N1 P1 Na1 460.2798485 3.198789512 0.022617253 1.469196419 0.081963593 0.221450294 2597355.942 2306379.146 3033711.223 2674498.021 5162666.343 4026188.475 2401900.49 6926813.1 4151847.244 4135721.32 6196582.956 5278398.097 5077328.775 5281979.126 5281144.327

380POS PE(17:1/20:4)+H PE(37:5)+H PE (17:1/20:4) (17:1) (20:4) C42 H75 O8 N1 P1 752.5224835 9.5148436 0.003027027 1.094508849 0.479953285 0.412465101 40849019.86 41315419.76 43830166.74 33248523.14 49466574.34 30682352.12 28593131.33 48869894.07 36762016.35 53625978.31 55105980.29 39059723.36 49883028.83 49885570.76 49623223.74

381POS PC(34:5)+H PC(34:5)+H PC (14:0/20:5) (14:0) (20:5) C42 H75 O8 N1 P1 752.5224835 7.9504531 0.008622234 1.106868024 0.695002093 0.347047601 60900891.62 33854233.22 60846225.04 32216267.86 34594995.62 49484288.01 19953228.52 48790082.04 76108167.27 33001644.71 36465776.54 86635086.89 70828442.79 71894477.62 71899191.27

382POS PE(36:2e)+Na PE(36:2e)+Na PE (36:2e) (36:2e) C41 H80 O7 N1 P1 Na1 752.5564635 11.67245268 0.00524034 0.992722372 0.94405693 0.437776944 71087701.5 77935068.35 80620894.44 97865304.62 97093304.26 80188795.3 50817866.44 98802739.76 75650035.85 92525115.76 92224555.17 91097073.63 95999471.42 96876613.98 96875622.54

383POS PE(18:0p/20:4)+H PE(38:4p)+H PE (18:0p/20:4) (18:0p) (20:4) C43 H79 O7 N1 P1 752.5588685 10.82446204 0.0034495 1.057376883 0.746541007 0.205152644 272592543.4 224031400.2 293788101.3 256869963.1 333280820.5 318173489.4 142196486.3 357270369.4 333207090.3 236736220.7 257965256.1 468829089.3 394110150.8 394108141.4 396468532.1

384POS DG(28:1/16:0)+NH4 DG(44:1)+NH4 DG (28:1/16:0) (28:1) (16:0) C47 H94 O5 N1 752.7126505 14.66631308 0.012334363 0.823754516 0.599395219 0.347498915 21706181.87 21242121.64 21157317.53 26462496.81 8625517.548 12455449.91 3919092.111 29870939.74 27300614.41 3401730.954 2736866.029 24742194.95 26885052.36 26888294.48 27465186.33

385POS WE(53:11)+H WE(53:11)+H WE (53:11) (53:11) H85 C53 O2 753.6544065 17.464 0.009620053 1.687348397 0.267363417 0.084812684 2182538.389 184162.9306 1836943.091 409124.1681 822742.937 2987447.854 3065055.27 2365926.683 2662786.613 617258.171 492673.7501 5008766.507 2990340.434 3048356.52 3022224.083

386POS PE(17:0/20:4)+H PE(37:4)+H PE (17:0/20:4) (17:0) (20:4) C42 H77 O8 N1 P1 754.5381335 10.27860987 0.061993857 1.279881695 0.109443898 0.225256057 74228938.59 58851051.11 76887757.81 54376878.59 51393860.14 63883857.44 99252417.42 81877528.79 113538980.6 57928934.14 57569660.82 75704167.02 102325615.7 92089619.64 92054922.64

387POS PC(32:1)+Na PC(32:1)+Na PC (32:1) (32:1) C40 H78 O8 N1 P1 Na1 754.5357285 9.585976402 0.011527663 0.920471136 0.317367486 0.72097613 249468620.1 269865765.7 240763179.8 233290704.1 226331955.8 228161395 300426144.3 196116078.3 212197862.3 227469920.2 214970405.9 181552828.3 208010980.6 203864982.1 207969431.2

388POS PC(34:4)+H PC(34:4)+H PC (14:0/20:4) (14:0) (20:4) C42 H77 O8 N1 P1 754.5381335 8.668240177 0.005028707 1.083820369 0.707259158 0.902493629 473057401.1 293379087.6 525837588.5 339649094.9 340884589.9 462644925.6 204718005 478995980.1 618989735.4 301441681.8 332051374.6 703396454.1 596927831 602203041.1 597050621.5

389POS PC(34:4)+H PC(34:4)+H PC (34:4) (34:4) C42 H77 O8 N1 P1 754.5381335 8.230062368 0.237597058 1.021280083 0.954183559 0.639230451 82574607.03 88276069.11 89461695.04 55065985.33 41642986.99 86678939.41 21288470.88 129185070 97577555.13 21360004.36 22768002.94 160963158.4 160552020.6 108819400.8 108590297.6

38POS LSM(d18:1)+H LSM(d18:1)+H LSM (d18:1) (d18:1) C23 H50 O5 N2 P1 465.3451875 2.414 0.013969934 1.134493936 0.561027921 0.065048728 2066204.503 1581998.451 2680246.154 2679872.804 2514743.025 2074411.111 1159277.709 3134726.373 3535657.226 1854926.087 1682265.574 4059401.155 3397883.293 3481387.117 3481510.015

390POS PE(18:0p/20:3)+H PE(38:3p)+H PE (18:0p/20:3) (18:0p) (20:3) C43 H81 O7 N1 P1 754.5745185 11.32535612 0.00225568 1.007385055 0.96815272 0.335271387 129291202.6 120375475.7 154086794.9 144164894.4 163019538.2 163583383.8 72438471.7 211630102.9 170009330.9 101370886.8 106665509.9 218865374.9 206791976.2 207606790.1 206803726.2

391POS PC(34:3)+H PC(34:3)+H PC (34:3) (34:3) C42 H79 O8 N1 P1 756.5537835 8.951167948 0.002884106 1.100338482 0.64879215 0.890161851 1659401172 1188725781 1646958667 1572896472 1894999355 2292723384 602938108.5 1889374428 2262145192 1971277188 1495889075 3063122695 2341184265 2352921686 2341229612

392POS PC(32:0)+Na PC(32:0)+Na PC (16:0/16:0) (16:0) (16:0) C40 H80 O8 N1 P1 Na1 756.5513785 10.33210726 0.00602243 0.92034382 0.47000948 0.5136675 115625044.2 122533096.4 113146618.4 101218133.3 107499451.3 100547547.3 150398265.1 79427301.07 76720329.73 104840408.4 108197941 88367171.44 88721892.51 87709129.28 88497086.53

393POS PC(34:3)+H PC(34:3)+H PC (34:3) (34:3) C42 H79 O8 N1 P1 756.5537835 9.377334902 0.004009618 1.01289811 0.944077552 1.150741666 1694217682 1035363526 1658854786 1151427490 1298002013 1661137045 768246253 1486353482 1754849577 1121010168 1157153414 2321010719 1876538581 1889570472 1889691685

394POS PE(20:1p/18:1)+H PE(38:2p)+H PE (20:1p/18:1) (20:1p) (18:1) C43 H83 O7 N1 P1 756.5901685 11.71723095 0.038419378 1.099624062 0.690987357 0.192463445 25534662.45 20611616.91 31584366.93 29875861.98 36801872.13 60500166.94 15477907.02 47465967.56 34543084.91 53237511.7 26141319.38 48456578.5 46500993.65 49795177.56 49679622.23

395POS PC(34:2)+H PC(34:2)+H PC (34:2) (34:2) C42 H81 O8 N1 P1 758.5694335 9.779482123 0.007083474 1.199686186 0.452131505 6.972114103 15699121371 14287715731 15602864424 28974969017 15219749565 16236867772 9872831849 18051698463 18423742424 16244082316 27948926945 36650992469 18466349282 18694245448 18695311105

396POS PC(34:2)+H PC(34:2)+H PC (34:2) (34:2) C42 H81 O8 N1 P1 758.5694335 11.00529239 0.011054959 2.279270809 0.309217152 2.223544056 137686161 144688621.4 148497542.5 161962355.3 187411105 153711874.8 79388801.5 224661404.8 191531936.3 1278204326 178571911.8 176384051.1 217573241.4 217669724.9 221814374.1

397POS PC(35:1p)+H PC(35:1p)+H PC (35:1p) (35:1p) C43 H85 O7 N1 P1 758.6058185 11.0077825 0.055718094 1.107280893 0.574646553 0.292547383 74738613.33 81720681.52 74431160.6 82239849.9 124826823.9 92656784.89 32612722.06 118900695.1 130226980.2 95835135.21 91557111.86 118406004.2 151405422.5 137603221.9 137752027.3

398POS PE(20:0p/18:1)+H PE(38:1p)+H PE (20:0p/18:1) (20:0p) (18:1) C43 H85 O7 N1 P1 758.6058185 12.24192871 0.000506783 1.074822935 0.708376108 0.214290976 66534598.64 57002985.95 76253391.62 85440687.01 88373534.77 93106827.59 33897632.61 122961822.2 85560610 64951687.14 73088908.76 121172128.3 119240613.1 119234973.4 119133274

399POS PC(35:1p)+H PC(35:1p)+H PC (35:1p) (35:1p) C43 H85 O7 N1 P1 758.6058185 10.57892244 0.032240408 1.239860078 0.423393 0.890511449 77337852.76 47142754.23 59745737.57 84975352.39 156328682.9 87267327.36 40103803.99 150339270.7 67324033.88 142272534.8 145018848.1 90738913.79 77732543.68 82231763.04 82250440.91

39POS LPC(14:1)+H LPC(14:1)+H LPC (14:1) (14:1) C22 H45 O7 N1 P1 466.2928185 1.683 0.031265165 1.225239532 0.665031606 0.054019257 725921.6822 270248.8377 895446.7087 620654.1875 503979.1623 1050103.561 364835.5066 566306.6099 1043508.787 385165.2565 278634.1407 2343807.544 1121194.592 1175453.081 1108693.912

3POS So(d17:1)+H So(d17:1)+H So (d17:1) (d17:1) C17 H36 O2 N1 286.2740555 2.178678593 0.012152218 1.125766868 0.427668942 0.024423508 1550264.67 1242433.983 1761499.079 1278428.703 1867101.774 1931186.536 1284781.85 1813405.685 2687447.482 1413217.377 1552758.338 2090553.998 2473449.526 2422937.159 2421316.967

400POS SM(d38:1)+H SM(d38:1)+H SM (d38:1) (d38:1) C43 H88 O6 N2 P1 759.6374525 11.13524433 0.007342015 1.045854433 0.663499088 0.221341911 206010540.9 155643586.6 227507755 158802755.5 200388499.9 200032072.4 141341878.9 207429685.5 212568939.4 183118444.2 197107437.4 259477377.3 233134757.2 233172860.7 236131203.9

401POS PS(16:1/18:1)+H PS(34:2)+H PS (16:1/18:1) (16:1) (18:1) C40 H75 O10 N1 P1 760.5123135 8.866389347 0.010306005 0.957371268 0.919669578 0.256214295 17411953.44 16528923.31 33242731.93 33282032.96 11671565.79 67641896.87 16746462.57 35210139.83 23006105.14 8976615.557 17808762.57 70367263.36 14569570.34 14807939.01 14528640.76

402POS PC(16:0/18:1)+H PC(34:1)+H PC (16:0/18:1) (16:0) (18:1) C42 H83 O8 N1 P1 760.5850835 10.39762054 0.002429053 0.964662104 0.575858783 5.595759392 33678595014 31497427098 36668033105 31785060147 31380023911 37063874445 25591506531 36824092940 35427845644 30316330547 30966948577 35805454379 35053804618 35053732611 35201455451

403POS PC(34:1)+H PC(34:1)+H PC (34:1) (34:1) C42 H83 O8 N1 P1 760.5850835 13.55962051 0.128000136 1.307902243 0.442386302 0.353615275 26951058.94 49971045.89 58623405.03 35745532.27 66343956.31 34002160.46 17806420.18 41339870.53 108138733.3 29756595.31 48896632.64 109336597.5 79529860.7 79464329.74 63085315.13

404POS PC(34:1)+H PC(34:1)+H PC (34:1) (34:1) C42 H83 O8 N1 P1 760.5850835 13.00683168 0.015352139 1.046221934 0.83987205 0.14805644 22085034.69 30632282.98 51177913.51 32837597.24 30799410.48 42111358.73 12299001.72 45482405.66 43166519.31 31816969.41 27887321.85 58681512.14 50336219.64 49009523.38 50336258.33

405POS PC(34:1)+H PC(34:1)+H PC (34:1) (34:1) C42 H83 O8 N1 P1 760.5850835 31.89429563 0.178369958 0.919820825 0.740279737 0.179035202 32444551.39 48512185.71 50937902.92 31069196.62 24833971.11 31896923.22 27154656.07 28906116.73 11993169.48 22589903.87 60185259.09 51250683.49 34793019.8 36643442.46 47903127.39

406POS PC(34:1)+H PC(34:1)+H PC (34:1) (34:1) C42 H83 O8 N1 P1 760.5850835 12.42681676 0.043720353 2.020964841 0.194623765 1.726284621 92267589.66 56836919.23 74510438.79 135324166.8 103636605.8 66242336.25 19194697.26 415732192.1 122023301.1 90733881.8 326612948.1 94425679.01 91609622.99 98358440.71 90968095.52

408POS PC(34:1)+H PC(34:1)+H PC (34:1) (34:1) C42 H83 O8 N1 P1 760.5850835 16.63455126 0.004368775 1.134936152 0.5074335 0.113517128 10585039.54 4255173.475 7534583.253 7348007.36 7678660.99 9035451.296 3251651.471 10923685.67 10461004.13 9314399.134 7358741.405 11393452.86 10230569.97 10275233.81 10185843.88

409POS PC(34:1)+H PC(34:1)+H PC (34:1) (34:1) C42 H83 O8 N1 P1 760.5850835 13.87617961 0.060146912 1.110294717 0.727752582 0.143319958 37944739.37 22237879.02 18850149.5 32752653.18 29636936.91 35848073.02 12691216.44 69581044.61 27365190.43 29645150.73 15215056.87 42324764.02 63372229.14 63510516.91 57055100.36

40POS LPC(14:0)+H LPC(14:0)+H LPC (14:0) (14:0) C22 H47 O7 N1 P1 468.3084685 1.975669118 0.008275116 1.124777514 0.671412961 0.150080669 14243835.13 9033990.49 18716559.67 11103221.84 17866713.74 15099066.4 11205949.97 14921244.14 21199631.11 9310577.637 7767400.746 32397359.18 21850482.71 22167295.93 21854721.74

410POS PC(34:1)+H PC(34:1)+H PC (34:1) (34:1) C42 H83 O8 N1 P1 760.5850835 15.87566879 0.07335907 1.184547893 0.68786797 0.13103323 25907275.03 5975254.971 7224580.763 8205457.571 6589632.35 10071677.11 3180446.719 7968310.618 27780909.75 14287057.08 6657962.6 15905435.37 19275874.2 16977029.96 17060960.87

411POS PC(34:1)+H PC(34:1)+H PC (34:1) (34:1) C42 H83 O8 N1 P1 760.5850835 31.25901597 0.073433262 1.023756852 0.926608902 0.137761988 12930930 18380548.58 14920886.49 16724023.47 21583823.35 15832245.35 2947543.448 25582615.85 17533362.59 30003033.98 10253257.24 16437177.76 13817464.07 15681193.9 15789287.63

412POS PC(34:1)+H PC(34:1)+H PC (34:1) (34:1) C42 H83 O8 N1 P1 760.5850835 17.35783012 0.231231565 1.631879659 0.273349387 0.197465634 3224834.396 4693376.137 2971399.848 3159083.697 3689051.409 3454283.002 1854525.286 10089712.76 13120595.77 2680443.213 2838466.679 3999096.517 6205059.236 9234864.433 6437254.182

413POS PE(18:2/18:2)+Na PE(36:4)+Na PE (18:2/18:2) (18:2) (18:2) C41 H74 O8 N1 P1 Na1 762.5044285 8.766950473 0.015382978 1.005703919 0.972095105 0.209600492 77050863.23 61813736.55 81539409.25 70069540.37 64110035.7 78944449.51 33191920.41 84379622.97 85476915.79 56204228.77 65977828.84 110770326.6 104665880 107482672.3 104671877.4

414POS PC(34:0)+H PC(34:0)+H PC (34:0) (34:0) C42 H85 O8 N1 P1 762.6007335 11.06356928 0.001566734 0.887231768 0.32821265 3.446393813 3316672271 3590094879 3113627332 3308558391 3671538411 2965900653 1461724230 3694246540 3558195852 2681458703 2750119252 3569072632 3829030631 3818670482 3829072940

415POS SM(d39:6)+H SM(d39:6)+H SM (d39:6) (d39:6) C44 H80 O6 N2 P1 763.5748525 15.55373389 0.004082461 1.564215957 0.166608405 0.136614444 5806956.015 1470210.032 5218917.166 2069059.255 3864988.674 7519682.378 7488221.787 6646533.055 9272650.315 3132431.438 2935307.442 11115968.34 6616485.775 6569910.422 6616686.556

416POS PC(35:6)+H PC(35:6)+H PC (35:6) (35:6) C43 H75 O8 N1 P1 764.5224835 9.620261653 0.003214669 1.038653743 0.709258544 0.457909018 488713665.6 356036940.8 501551644 370890469.2 326378357.1 352571463 299637615.9 486846177.3 417091420 403610689.1 440111310 441465206 524283751.8 527211085.4 527216556

417POS PE(18:1/18:2)+Na PE(36:3)+Na PE (18:1/18:2) (18:1) (18:2) C41 H76 O8 N1 P1 Na1 764.5200785 9.338158202 0.004972504 1.206851608 0.189719586 1.570688213 386908051.5 316447959.9 422367485.6 322537734.4 394242831.9 395333131.6 200650427.3 509655638.7 466791606.4 454690367.8 517799462.6 551149913.3 587671518.9 587730801.5 592777125.5

418POS PC(36:5p)+H PC(36:5p)+H PC (36:5p) (36:5p) C44 H79 O7 N1 P1 764.5588685 9.404020337 0.0059697 1.495874569 0.119904622 1.578085426 117171051.7 149101294.5 111610044.6 119073509.6 281369012.8 159939966.1 71880939.21 240147772.1 231358389.6 307863080.7 332876335.3 219400055.1 248157071.3 250758067.3 250723140

419POS PC(35:5)+H PC(35:5)+H PC (35:5) (35:5) C43 H77 O8 N1 P1 766.5381335 9.927768602 0.005247542 1.198327664 0.149397163 3.446787046 1435679331 1450342285 1648949208 1530947058 1935402942 1552876033 1106895601 2136959723 1755202407 2238000564 2380657871 1831342239 2188330594 2208389874 2208292984

41POS LPE(16:0)+Na LPE(16:0)+Na LPE (16:0) (16:0) C21 H44 O7 N1 P1 Na1 476.2747635 2.811134052 0.021530639 1.300931921 0.061985571 0.076554514 1967772.542 1109509.072 2052086.74 1078889.776 1287821.514 1521732.948 1907228.46 2359993.496 1534026.446 1691635.566 2008233.895 2230442.394 2031545.945 2108605.024 2109855.932

420POS PE(18:1/18:1)+Na PE(36:2)+Na PE (18:1/18:1) (18:1) (18:1) C41 H78 O8 N1 P1 Na1 766.5357285 10.70024403 0.031105325 0.987991739 0.862911466 0.601325811 127642125.1 136740396.3 129309624.4 127706066.7 152629046.7 151536267.4 124345398.1 123941486.9 119518962.2 162889026.3 159251719.5 125703351.7 151093592.9 151060944.9 159365505.7

421POS PE(18:0/20:5)+H PE(38:5)+H PE (18:0/20:5) (18:0) (20:5) C43 H77 O8 N1 P1 766.5381335 10.14601427 0.006272722 1.105113812 0.416164971 0.906453945 786666898 555949157.6 851967003.8 593728708.1 575946943.1 634694888.1 475342309.8 856169842.7 821318019.4 637361012.8 721386592.4 907721077 922144091.3 917024000.1 910666986.6

422POS PC(33:2)+Na PC(33:2)+Na PC (33:2) (33:2) C41 H78 O8 N1 P1 Na1 766.5357285 9.233767878 0.017192194 0.871864321 0.388492438 0.912623296 165459718.6 149825676.2 156596001.6 172712606.2 271517877.7 266430360 163588757.4 149062758.6 134629334.5 243515710.2 152656197.9 187563628.4 158095243.3 158102467.3 153437300.9

423POS PC(35:5)+H PC(35:5)+H PC (35:5) (35:5) C43 H77 O8 N1 P1 766.5381335 8.47706788 0.016339312 1.167145582 0.438634185 0.225954487 20839933.57 17138776.92 25970900.74 15289763.9 19199854.75 17708490.06 7298929.129 23966597.35 32814251.24 21843916.39 19260045.28 30377558.81 28485089.18 29298981.44 28485229.79

424POS PC(36:4p)+H PC(36:4p)+H PC (36:4p) (36:4p) C44 H81 O7 N1 P1 766.5745185 9.98693542 0.00927916 1.458940248 0.165108941 2.670634924 326830477.9 477749002.9 312597077.2 389229226.3 867797688.3 430036829.9 214039368.7 724212745.6 666019260.4 945054772.7 991945161.5 549947733.2 672697817.1 683599559.1 683653237

425POS PE(18:0/20:4)+H PE(38:4)+H PE (18:0/20:4) (18:0) (20:4) C43 H79 O8 N1 P1 768.5537835 10.66656847 0.004119077 1.072072312 0.399495343 0.912918638 2119070882 1862636691 2329601345 1849154937 1727491138 1931525042 1606385065 2475133339 2412156302 1956991915 1966760167 2253910501 2508563422 2490728474 2508603261

426POS PC(35:4)+H PC(35:4)+H PC (35:4) (35:4) C43 H79 O8 N1 P1 768.5537835 9.170806295 0.004569467 1.036334404 0.839919511 0.103809409 91946211.65 72384361.22 89250850.2 141431044 78597820.55 72215020.45 48846266.53 110191182.9 133487531.5 81170810.49 86161500.16 105800253.9 119640599.5 120595580.7 119651759.9

427POS PE(36:1)+Na PE(36:1)+Na PE (36:1) (36:1) C41 H80 O8 N1 P1 Na1 768.5513785 11.33283378 0.009066377 0.988614008 0.807293032 0.615443965 281551272.4 267636849.9 298289723.6 282067008.4 250559759.1 251704954.8 236472081.6 274449167.6 257581559.7 284539881.9 303603559.8 256583547.3 296051863.8 296064531.6 300731776.5

428POS PE(18:1/20:3)+H PE(38:4)+H PE (18:1/20:3) (18:1) (20:3) C43 H79 O8 N1 P1 768.5537835 10.26576108 0.029037703 1.140606287 0.36395572 0.776329659 185565441.1 169626194 184282821.4 174053955.4 181462671.3 172984354 121587455 216554287.7 193948958.4 154449857.6 307111610.8 224487328.4 268309624.7 254979575.2 255543812.2

429POS PC(35:4)+H PC(35:4)+H PC (35:4) (35:4) C43 H79 O8 N1 P1 768.5537835 8.764379813 0.069157419 0.804823558 0.667123766 0.706567094 182516416.1 39427166.94 62940343.1 35091731.19 62216437.34 51811039.17 36000626.65 46083234.71 61261120.62 20198574.86 19613919.6 166138470.1 97573023.08 110274199.8 110275922.7

42POS AcCa(22:4)+H AcCa(22:4)+H AcCa (22:4) (22:4) C29 H50 O4 N1 476.3734355 2.664 0.004473159 1.422848049 0.449964352 0.091978366 1140645.321 5080617.512 1600596.286 6513379.493 3192598.955 1216882.42 6278065.025 5530981.679 1253059.519 793997.3136 8369636.676 5176967.162 5220688.849 5185193.995

431POS PC(36:4e)+H PC(36:4e)+H PC (36:4e) (36:4e) C44 H83 O7 N1 P1 768.5901685 10.144267 0.005073162 1.0853225 0.715949476 0.605250144 473470134.3 335562488.7 464020493.4 401190549.5 497986259.4 613562831 198277140.8 529395044.6 611473435.6 381868613.8 398305689.3 904163633.4 604186641.6 609527248.3 609526545.9

432POS TG(16:0/14:0/14:0)+NH4 TG(44:0)+NH4 TG (16:0/14:0/14:0) (16:0) (14:0) (14:0) C47 H94 O6 N1 768.7075655 14.71960095 0.005127891 0.89393338 0.585927297 0.099364699 3832345.29 2878932.51 3833613.389 5407617.653 3280271.339 3820999.668 2266232.776 5344189.38 5124141.02 2337671.037 1749578.25 3786730.869 5515211.39 5564636.712 5564337.901

433POS PE(18:0/18:0)+Na PE(36:0)+Na PE (18:0/18:0) (18:0) (18:0) C41 H82 O8 N1 P1 Na1 770.5670285 10.9697249 0.008126345 1.097331498 0.471117805 0.657640282 170707582.9 144205999.7 193984867.2 175662303.7 177629474.1 150101505.7 106556269.2 264264040.2 185702944.3 172123799 187410872.1 194761679.1 235715632.6 239068971.3 239060641.7

434POS PE(38:3)+H PE(38:3)+H PE (38:3) (38:3) C43 H81 O8 N1 P1 770.5694335 10.96474769 0.001298972 1.033852673 0.816613765 0.269414538 341175500.9 290580661.9 395565632.4 364329623.9 332762804.8 305165309.9 196946261 490224505.9 424435403.9 266985190.9 303690103.9 416004760.4 478394723.4 478972431.2 479638105.9

435POS PC(35:3)+H PC(35:3)+H PC (35:3) (35:3) C43 H81 O8 N1 P1 770.5694335 9.414200323 0.00577645 1.036130176 0.803951783 0.089196779 434638330.1 380055333.7 427922980.1 405427918.1 519593421.1 462498425.2 227509889.2 512262880.5 579713525.4 379472930.3 410690902.5 615513572.4 582067110.4 587950600.6 587908890.6

436POS PC(35:3)+H PC(35:3)+H PC (35:3) (35:3) C43 H81 O8 N1 P1 770.5694335 9.81939049 0.051401191 0.983219325 0.91783433 0.247949296 167702978.8 101323500.4 132080344.6 138379642.8 133785543.6 108962833.6 78747235.38 123596161.9 120751904.1 109744498.3 123407457 212861158.1 136245703.9 136413228.7 148837149.9

437POS PC(36:2p)+H PC(36:2p)+H PC (36:2p) (36:2p) C44 H85 O7 N1 P1 770.6058185 10.81346042 0.047896768 1.177948215 0.42379842 1.196625299 363479381 495735144.8 361225512.8 593781613.2 801356647.2 520136445 187989709.6 693143660 794633600.8 638946181.8 635367353.8 743629078.1 847265394.1 778347338.3 846117372.2

438POS PC(16:0e/18:0)+Na PC(34:0e)+Na PC (16:0e/18:0) (16:0e) (18:0) C42 H86 O7 N1 P1 Na1 770.6034135 11.51963678 0.002703844 0.713278509 0.180283287 0.98452409 83236862.79 65183963.86 78397089.43 88923116.03 109424738.2 130459684.6 40650232.62 63522876.87 67338719.07 42182868.04 41702215.04 140918784.3 92341896.35 91935551.63 92389115.38

439POS PC(36:2p)+H PC(36:2p)+H PC (36:2p) (36:2p) C44 H85 O7 N1 P1 770.6058185 10.349568 0.185428777 0.771592054 0.366895467 0.420006604 38252241.38 95041555.2 134864114.3 79657755.34 68073465.15 46146279.85 77495424.73 73285859.39 31176827.03 25920334.52 99755929.94 48868476.58 57840062.69 65469121.18 82696066.45

43POS LPE(18:2)+H LPE(18:2)+H LPE (18:2) (18:2) C23 H45 O7 N1 P1 478.2928185 2.28566003 0.014973143 1.14622948 0.501498453 0.064990156 3472580.71 2732356.344 4441191.834 2222358.81 4763680.058 3608789.142 2272782.907 6057031.409 4189508.011 2686152.395 3517499.281 5624036.974 5475544.411 5621969.278 5618043.082

440POS ChE(26:6)+NH4 ChE(26:6)+NH4 ChE (26:6) (26:6) C53 H88 O2 N1 770.6809555 16.20756378 0.003833137 1.490432369 0.364986716 0.320478825 18807062.08 3157268.969 17536391.24 7040989.152 7042413.648 24478457.36 15486185.94 22079165.46 30238091.44 4895536.526 3933108.725 39714911.58 27741637.21 27926840.56 27742860.97

441POS PC(35:2)+H PC(35:2)+H PC (35:2) (35:2) C43 H83 O8 N1 P1 772.5850835 10.58185806 0.008566033 0.941801875 0.480765484 0.629992945 174008082 207088057.5 206877091.2 203913461.3 215628724.9 161910852.9 125252920.9 184788615.6 211221010 189378185 181394032.1 209333090.5 221427925.2 224743135.1 224748913.5

442POS PC(35:2)+H PC(35:2)+H PC (35:2) (35:2) C43 H83 O8 N1 P1 772.5850835 10.08368414 0.031723821 1.006484658 0.966364429 1.226090434 5121441845 4946699525 5001049669 5506526001 7534814423 6565739932 2620395256 7710822888 7015394132 5079554905 5568277476 6906690514 7298754512 7710983358 7305850839

443POS PC(35:2)+H PC(35:2)+H PC (35:2) (35:2) C43 H83 O8 N1 P1 772.5850835 11.32971892 0.025664591 1.034621122 0.840805877 0.540470111 195297455.2 200962741.9 245163685.9 284981885.1 253674915.7 193303955.3 92347590.71 362907736.3 249180197.3 229243548.8 248929347.4 238324336.1 358544268.1 358514767.6 374706691.2

444POS PC(36:2e)+H PC(36:2e)+H PC (36:2e) (36:2e) C44 H87 O7 N1 P1 772.6214685 10.92058433 0.008751701 0.979761488 0.922076918 0.625033761 622142331.7 527251543 608210704.7 695336629 998517931.1 695641705 241910890.4 924051355.4 889433009.5 487956249.3 530356097.1 989462091.6 883912067.1 897648530.9 884459993.3

445POS PC(36:2e)+H PC(36:2e)+H PC (36:2e) (36:2e) C44 H87 O7 N1 P1 772.6214685 11.45852596 0.019401309 1.23365314 0.388226209 1.287640192 424016043.4 541886201.1 432295250.3 541114864 985983620.8 617068559.8 155348523.8 930735856.9 960022592.4 661802875.4 692511896.5 969627391.6 924953593.2 956666358.3 956829133.1

446POS ChE(26:5)+NH4 ChE(26:5)+NH4 ChE (26:5) (26:5) C53 H90 O2 N1 772.6966055 16.82695643 0.004942889 1.526894018 0.33473052 0.211013237 9311887.016 1776704.976 8256178.02 2804096.789 3215658.696 11454465.21 8495172.584 10421751.08 13320214.17 2335657.003 2086135.967 19559765.87 12950370.52 13061945.63 13061813.1

447POS PC(36:8)+H PC(36:8)+H PC (36:8) (36:8) C44 H73 O8 N1 P1 774.5068335 7.957539383 0.010203372 1.184976158 0.591092114 0.20996706 13325928.2 7194373.956 15795287.33 8877054.232 9071178.79 13031497.11 3304937.581 13262966.6 19244060.09 8087051.299 9002718.213 26841615.51 20507727.58 20870327.84 20878487.73

448POS PC(35:1)+H PC(35:1)+H PC (35:1) (35:1) C43 H85 O8 N1 P1 774.6007335 10.77856462 0.00939261 0.93965277 0.675196831 2.217470706 5750448023 5365459368 7786916441 5392464946 5394410657 6834611041 3139947488 7435302113 8057243432 4737376387 4954002625 5996297450 6765175771 6765195706 6875845096

449POS PC(35:1)+H PC(35:1)+H PC (35:1) (35:1) C43 H85 O8 N1 P1 774.6007335 11.26969594 0.014065853 2.095297616 0.164467856 2.17201757 204264534 183112185.7 143015084.2 159256735.2 193216418 101755372.6 93620679.69 831160893.4 227712291.1 567979537.5 162915664.6 179683563.1 237949759.4 243749768.3 237863691.3

44POS LPC(16:1p)+H LPC(16:1p)+H LPC (16:1p) (16:1p) C24 H49 O6 N1 P1 478.3292035 2.366605892 0.006490755 1.057623059 0.753433988 0.029622429 1671613.687 1486483.564 1945848.727 1511544.177 2110503.177 1661333.922 890634.6507 2429113.591 2533145.165 1431691.235 1275343.472 2425948.709 2451681.299 2424282.773 2424364.933

450POS PE(38:1)+H PE(38:1)+H PE (38:1) (38:1) C43 H85 O8 N1 P1 774.6007335 11.91729953 0.005364731 1.125941667 0.478832557 0.527003466 77214398.36 77274752.99 97596602.81 99170972.13 90119039.76 78958131.29 39512779.09 142927751.9 111489878.1 80548648.6 100987471.2 110399087.1 130212061.9 131438103.5 130236920.8

451POS PC(36:1e)+H PC(36:1e)+H PC (36:1e) (36:1e) C44 H89 O7 N1 P1 774.6371185 11.56535245 0.00664077 1.016499485 0.956142114 1.58207149 690182874.3 601882877.5 688462881.9 833276980.2 831974850.7 852144560 206384495.8 1113184139 1173779614 322141903.9 345473737.6 1411174582 1098155010 1112747535 1104096815

452POS PC(36:1e)+H PC(36:1e)+H PC (36:1e) (36:1e) C44 H89 O7 N1 P1 774.6371185 12.0270372 0.005524961 1.005890083 0.980862553 0.20604285 27446883.12 24372712.95 27113509.7 28298563.82 38817973.92 35468375.18 8625840.521 40347086.67 42127729.11 19620420.62 19652123.57 52213974.32 42647459.33 43056798.54 43059577.48

453POS ChE(26:4)+NH4 ChE(26:4)+NH4 ChE (26:4) (26:4) C53 H92 O2 N1 774.7122555 17.46668797 0.013679069 1.654676426 0.316150417 0.126940506 3458437.224 559895.9642 3170796.219 705226.1495 1042125.182 5068441.112 4137397.526 3440104.007 4951866.198 865123.9753 798842.536 8980279.797 4983962.133 5102825.018 5105027.108

454POS PC(14:0/20:4)+Na PC(34:4)+Na PC (14:0/20:4) (14:0) (20:4) C42 H76 O8 N1 P1 Na1 776.5200785 8.668240177 0.017009489 1.10940957 0.498491024 0.302798159 52160971.67 40785020.71 35945606.08 27756591.16 41114500.96 27162643.76 47181529.94 34279359.63 42065682.39 48789311.91 53195965.59 24022469.03 36293849.83 36293613.75 35234869.78

455POS PC(34:4)+Na PC(34:4)+Na PC (34:4) (34:4) C42 H76 O8 N1 P1 Na1 776.5200785 8.230062368 0.011194344 0.755924311 0.422774207 0.415971253 29023757.56 15321779.81 53514542.34 20870315.24 19309586.43 36810867.24 9727194.204 21385926.21 32130173.49 10348244.01 11016729.63 47565739.75 34573317.73 35256224.65 34589694.6

456POS PC(36:7)+H PC(36:7)+H PC (36:7) (36:7) C44 H75 O8 N1 P1 776.5224835 9.241022162 0.028609004 1.16962413 0.311330738 0.328205751 27619224.69 23063492.83 26502742.33 24958155.87 25833479.4 19516070.94 11638423.34 36691466.07 36218462.88 27825851.39 29813015.26 30324347.13 39936896.56 41948557.77 39935872.34

457POS PE(18:1p/22:5)+H PE(40:6p)+H PE (18:1p/22:5) (18:1p) (22:5) C45 H79 O7 N1 P1 776.5588685 10.38779038 0.019835709 1.341184114 0.530759586 0.815500138 85559982.52 58982340.73 78076942.96 79053825.91 279287585.9 90010929.14 46662267.01 328897114.8 83240188.32 263855284.5 59215740.64 118025865.3 97505007.08 97503699.53 100893065.2

458POS PC(35:0)+H PC(35:0)+H PC (35:0) (35:0) C43 H87 O8 N1 P1 776.6163835 11.29261227 0.00231478 1.119355747 0.638409646 0.63199936 251478551.3 216234838.6 241376943.5 230993107.8 220263961.4 206494287.2 108467812.5 479947054.4 330608448.6 163417353.9 174157063.9 273384367.1 300499688.9 300500663.1 301706588.5

459POS PC(36:0e)+H PC(36:0e)+H PC (36:0e) (36:0e) C44 H91 O7 N1 P1 776.6527685 12.10822226 0.023093357 1.018036041 0.949575005 0.436115321 86051749.12 67270440.98 85078258.83 91172807.42 127708463 124141941.9 32183959.32 130404475.7 132899979.4 51643809.65 55313500.97 189464517.4 149009922.7 155051098 149010703

45POS LPE(18:1)+H LPE(18:1)+H LPE (18:1) (18:1) C23 H47 O7 N1 P1 480.3084685 2.783551503 0.012157026 1.055069893 0.788036022 0.034534158 43078003.59 40201189.73 74518697.47 48694996.62 76484334.7 77723479.84 43285488.45 78469545.98 61028650.46 43259117.59 51952973.17 102568675.2 78951217.65 80624894.71 78950205.37

460POS PE(17:0/22:6)+H PE(39:6)+H PE (17:0/22:6) (17:0) (22:6) C44 H77 O8 N1 P1 778.5381335 9.88952167 0.071001806 0.993724493 0.980115711 0.181350385 95339830.08 38543244.47 56915161.98 39962929.03 26704868.63 36826115.19 27968344.88 54410608.38 51380301.62 41035562.24 41899750.89 75750748.84 75429488.42 66885773.18 66826821.72

461POS PC(36:6)+H PC(36:6)+H PC (16:1/20:5) (16:1) (20:5) C44 H77 O8 N1 P1 778.5381335 8.285388327 0.05205556 1.218829162 0.629672276 1.257788826 174504596.8 310851594 316254201.5 277560387.5 196293025.1 269219498.5 72398523.16 442631828 725279754.5 92621152.88 86114099.03 463659699.2 451516780.8 411347582.6 450055545.6

462POS PC(36:6)+H PC(36:6)+H PC (36:6) (36:6) C44 H77 O8 N1 P1 778.5381335 8.953599525 0.005490748 0.749700377 0.095869427 1.300169215 211407629.8 404027822.6 218260622.1 214253257.6 235175377.1 210294632.6 136201398.3 219586849.1 216935688.1 169126937.7 176532359.9 201233810.6 221788410.3 223911465.1 221802512.3

463POS PE(18:0p/22:5)+H PE(40:5p)+H PE (18:0p/22:5) (18:0p) (22:5) C45 H81 O7 N1 P1 778.5745185 11.0185343 0.103135516 1.053438363 0.762294025 0.092147564 381891770 273153179.9 433803712.9 326879001.6 321884668.6 466111855.5 238720975.8 375777770.3 406763356.2 436707472.3 253352003.9 610166022.7 546595255.7 651320429.3 548125354.8

464POS DG(28:1/18:1)+NH4 DG(46:2)+NH4 DG (28:1/18:1) (28:1) (18:1) C49 H96 O5 N1 778.7283005 14.60331009 0.018794783 0.701427088 0.347550066 0.181542746 4344987.005 3476993.12 4223065.043 4040312.181 1353878.965 2241846.453 524792.986 3909356.06 4132758.28 351457.8544 292370.7283 4594108.672 4631443.062 4630377.607 4783313.726

465POS PC(36:5)+H PC(36:5)+H PC (16:0/20:5) (16:0) (20:5) C44 H79 O8 N1 P1 780.5537835 8.802442282 0.005026438 1.033615431 0.885659626 1.010529308 1059496363 654366656.9 1075593307 746808345.1 726589020.8 1049541091 334229480.8 918729699 1102259525 658805976 800989242.8 1675959299 1292763121 1304062589 1292786666

466POS PC(34:2)+Na PC(34:2)+Na PC (34:2) (34:2) C42 H80 O8 N1 P1 Na1 780.5513785 9.779482123 0.013004686 0.845504609 0.142003632 1.768681621 763236124.5 805422350 687918471.4 683220637.1 754589660.3 584890236.8 688450686.5 478743932.1 519365541.2 798078081.4 712013860.9 421496728.9 544672331.4 532647662.9 532523707.1

467POS PE(19:1/18:1)+Na PE(37:2)+Na PE (19:1/18:1) (19:1) (18:1) C42 H80 O8 N1 P1 Na1 780.5513785 10.294 0.122889615 1.829515745 0.053543009 0.697691633 42876205.37 37284913.88 41107120.71 32539596.97 31990656.27 31474957.76 75904676.8 44443181.79 96642879.04 34146496.96 111350483.2 35017481.71 67030291.19 54487419.76 54584677.43

468POS PE(18:0p/22:4)+H PE(40:4p)+H PE (18:0p/22:4) (18:0p) (22:4) C45 H83 O7 N1 P1 780.5901685 11.47438276 0.007389064 1.062891895 0.784406907 0.515875793 152196524.3 101618740.4 136333986.8 99730650.56 121002288.6 145655505.5 61131226.2 179035029.9 168600174.4 85968508.09 85057864.36 224324982.6 187553433.3 187662091.5 190017252.7

469POS DG(28:0/18:1)+NH4 DG(46:1)+NH4 DG (28:0/18:1) (28:0) (18:1) C49 H98 O5 N1 780.7439505 15.25248712 0.014342576 0.695936601 0.37539001 0.20189303 5115258.998 4998866.429 5540051.402 5988367.666 1426957.864 2567700.174 644165.3115 5454036.087 6237820.445 308838.2878 105919.241 5091088.21 6688845.32 6856401.783 6688853.615

46POS LPE(18:1)+H LPE(18:1)+H LPE (18:1) (18:1) C23 H47 O7 N1 P1 480.3084685 11.03164365 0.010191558 1.063676227 0.662580102 0.259765434 21995243.55 22909016.04 25198954.58 27958367.82 31872086.49 27823864.85 11329712.18 32817878.13 28337458.97 30027941.45 32914289.34 32375657.77 33081934.94 33084343.24 33670585.34

470POS MGDG(16:0/18:0)+Na MGDG(34:0)+Na MGDG (16:0/18:0) (16:0) (18:0) C43 H82 O10 Na1 781.5800215 11.57904285 0.009267174 0.808608765 0.363751348 0.621599942 92184394.98 58240398 105296277 63985978.55 34442260.77 79036023.92 62497433.66 65346168.52 69074757.28 31633143.09 27779337.5 93946617.2 72593748.87 72587308.32 73761949.12

471POS PC(36:4)+H PC(36:4)+H PC (16:0/20:4) (16:0) (20:4) C44 H81 O8 N1 P1 782.5694335 9.615077765 0.003583934 1.167994837 0.112430228 1.968213294 2444994048 1884895335 2357623273 1438666381 1807135446 1731963334 1898875016 2296172289 2625778752 2348410379 2364597062 2091150769 2396272225 2411189172 2411228815

472POS PC(36:4)+H PC(36:4)+H PC (36:4) (36:4) C44 H81 O8 N1 P1 782.5694335 10.37065979 0.074638998 0.860523466 0.429483398 0.966862354 280147362.9 346245468 519149463.3 495153043.5 280814574.4 264421276.6 381588521.2 225597949.3 250884329.3 479556163 298642236 244775884.6 339578458.3 387744083.6 387492995.9

473POS PC(36:4)+H PC(36:4)+H PC (36:4) (36:4) C44 H81 O8 N1 P1 782.5694335 5.752 0.03492995 1.276019348 0.443358343 0.075158301 418182.509 542228.864 507007.2929 989723.9373 1441720.812 743656.9605 106559.2558 1725352.316 969844.883 1025581.318 1240373.45 856234.5979 890157.8295 888921.1797 944454.0524

474POS PC(34:1)+Na PC(34:1)+Na PC (34:1) (34:1) C42 H82 O8 N1 P1 Na1 782.5670285 10.90912685 0.034106569 1.058522995 0.731639267 0.27137903 66152518.14 131690440 119473341.7 97634172.8 89992969.69 96085179.36 147062747.6 103890911 135851567.3 104110082.6 92987610.04 52299698.2 114987809 115064785.1 121957562.3

475POS PC(36:4)+H PC(36:4)+H PC (36:4) (36:4) C44 H81 O8 N1 P1 782.5694335 9.216143895 0.032323874 0.849357735 0.438773392 1.82171417 1044113668 722086713.5 1659079399 1245520724 817921061.2 1537438390 629237621.1 1146641169 1320730415 523136423 886126917.8 1461850758 1540715527 1546118840 1631344338

476POS PE(20:0p/20:3)+H PE(40:3p)+H PE (20:0p/20:3) (20:0p) (20:3) C45 H85 O7 N1 P1 782.6058185 11.91831555 0.030370432 1.000972244 0.997008276 0.172381543 34493464.89 28667861.06 41095909.39 27631738.91 30521287.77 67574191.42 18041625.29 59353096.63 37168876.75 25695850.64 28093057.49 61855547.64 42504344.72 42585139.3 44821576.66

477POS PC(34:0)+Na PC(34:0)+Na PC (34:0) (34:0) C42 H84 O8 N1 P1 Na1 784.5826785 9.837072502 0.005274947 1.106290176 0.516218139 2.481059428 8572764560 7922788877 8550050201 8594124262 10230515727 9534952229 5065864228 11152587996 11095122997 8331508030 8523184027 14913376255 10775773907 10778198542 10875728302

478POS PC(36:3)+H PC(36:3)+H PC (36:3) (36:3) C44 H83 O8 N1 P1 784.5850835 10.23209641 0.009290657 1.042328783 0.797569098 1.19849613 1610280172 1352312557 1739780818 1502157551 1535349064 1665005793 781236264.4 2157442253 2156647964 1229187257 1320819370 2157650220 2163473724 2163605028 2128909878

479POS PC(34:0)+Na PC(34:0)+Na PC (34:0) (34:0) C42 H84 O8 N1 P1 Na1 784.5826785 11.06356928 0.144347464 0.938491528 0.252186624 0.341613158 58997697.46 72915929.26 59842971.76 58182780.73 56862655.46 58264116.07 61643478.68 48768969.5 60692199.89 57934190.03 56101077.78 57471573.76 62452444.71 61890522.69 79120991.62

47POS LPC(16:0p)+H LPC(16:0p)+H LPC (16:0p) (16:0p) C24 H51 O6 N1 P1 480.3448535 2.982619237 0.004506771 1.172749302 0.391847005 0.323132048 35413690.34 31857718.74 39756047.29 31124102.3 42596322.86 33705249.26 19199696.17 55660646.79 57397763.05 30249716.39 33768265.41 55223671.67 56266128.29 56775515.69 56529132.05

480POS PC(34:0)+Na PC(34:0)+Na PC (34:0) (34:0) C42 H84 O8 N1 P1 Na1 784.5826785 11.32 0.012453379 0.986763239 0.921164011 0.317644277 61090746.66 48067692.62 90420065.34 106202241.1 91838323.44 73428723 53640386.12 78202627.57 90639386.22 78807496.64 85097357.25 78425391.47 84527329.81 82533728.65 83028092.55

481POS PC(37:2p)+H PC(37:2p)+H PC (37:2p) (37:2p) C45 H87 O7 N1 P1 784.6214685 12.21148392 0.006831633 1.055140621 0.778809991 0.055088389 20307990.29 15524491.55 20994031.68 27249654.64 25506956.3 23041908.05 11664478.44 31445268.48 23656024.3 17497986.1 18567548.07 37106753.78 39127361.28 39596541.26 39136107.1

482POS CerG1(d18:1/22:0)+H CerG1(d40:1)+H CerG1 (d18:1/22:0) (d18:1) (22:0) C46 H90 O8 N1 784.6660955 11.95427536 0.014963541 0.97711389 0.92758385 0.907506127 185840113.4 153193956.9 183090970.3 148665718.2 146026576.8 192414656.4 85200723.24 225903623.5 244063937.5 74309627.98 68656961.78 287999723.7 242956739.5 249331677.4 243002207.6

483POS SM(d40:2)+H SM(d40:2)+H SM (d40:2) (d40:2) C45 H90 O6 N2 P1 785.6531025 11.08325374 0.040749126 1.230452158 0.297221937 1.133051915 301954610.1 367051845.9 335629361.1 237101563.7 343895467.5 267341765.5 149077673.1 572004423.2 352364389.8 474906190.7 285099169.6 446544766 459865626.4 493350990.4 460329566.2

484POS PC(36:2)+H PC(36:2)+H PC (36:2) (36:2) C44 H85 O8 N1 P1 786.6007335 8.768621298 0.01437415 1.186760775 0.344048359 0.167617121 3591213.096 4811104.344 4355608.837 5569628.419 6727441.064 5273244.044 1955798.257 6911362.8 7257056.902 7172288.65 7026324.155 5669534.621 6970403.955 6971092.057 7145748.331

485POS PC(18:1/18:1)+H PC(36:2)+H PC (18:1/18:1) (18:1) (18:1) C44 H85 O8 N1 P1 786.6007335 10.47300028 0.045246586 0.927632835 0.550736852 7.593479776 16344715467 16845789654 15806984975 17650524980 27702157533 18822898689 11246853020 18943217009 18907058193 18198885040 18858184665 18828858998 23647197677 21893431975 21878651350

486POS PC(36:2)+H PC(36:2)+H PC (36:2) (36:2) C44 H85 O8 N1 P1 786.6007335 12.477 0.051343973 1.863301439 0.072290355 1.007022831 25616887.91 33124540.68 23635257.48 26913576.82 45828811.64 44299912.05 8164904.103 70298388.17 48707824.05 104285688 87182298.67 52938581.59 72960742.89 66869274.77 73396785.09

487POS PC(36:2)+H PC(36:2)+H PC (36:2) (36:2) C44 H85 O8 N1 P1 786.6007335 11.644 0.024719125 1.176208492 0.36228595 0.641823992 103548149.6 78458282.61 102611824.7 113533763.7 119049159.4 106182909.2 45999136.35 170358546.5 158430108.5 115667808.9 104428379.9 138345679.5 175987227.1 182027895.7 184719694.7

488POS PC(36:2)+H PC(36:2)+H PC (36:2) (36:2) C44 H85 O8 N1 P1 786.6007335 12.72718322 0.034599537 1.448598029 0.435473504 0.308987327 23497289.73 14825612.43 12924273.73 14290253.34 15260430.17 24141173.49 3839821.358 22722733.09 25544078.44 15538304.41 14418681.32 69950857.57 21733468.88 23083946.2 23096200.4

489POS PC(36:2)+H PC(36:2)+H PC (36:2) (36:2) C44 H85 O8 N1 P1 786.6007335 13.36534005 0.061807992 0.536698486 0.243950677 0.523846791 9402248.358 27302252.51 13727815.37 17485140 68877327.34 13148585.45 4535819.896 15848997.58 19547760.84 12040265.41 14200944.87 14300590.51 25904869.8 23840897.75 26945220.91

48POS LPC(15:0)+H LPC(15:0)+H LPC (15:0) (15:0) C23 H49 O7 N1 P1 482.3241185 2.19852975 0.098360952 1.460590325 0.097687018 0.094302787 2218271.765 1515249.865 2661327.721 2103222.338 2937830.325 1807801.206 2817160.994 2674561.904 5272583.924 2801944.99 1698059.512 4079313.471 3365572.493 3361678.502 3971165.544

490POS PC(36:2)+H PC(36:2)+H PC (36:2) (36:2) C44 H85 O8 N1 P1 786.6007335 16.66106773 0.060830459 1.16553651 0.628063082 0.085912972 3825385.831 1650993.956 2430648.929 3044088.645 2890718.946 3212905.006 1150486.841 2517237.141 6630369.385 2079814.946 2070496.373 5429518.987 3075152.692 3411961.262 3076985.753

491POS PC(37:1p)+H PC(37:1p)+H PC (37:1p) (37:1p) C45 H89 O7 N1 P1 786.6371185 12.76733355 0.000963011 1.110401472 0.595823401 0.180200485 18497404.8 17374564.13 21734529.71 29766157.46 23949163.39 25977360.91 10257181.15 37233822.61 25305332 20317196.59 22696316.85 36647362.82 36889512.24 36866649.42 36819876.71

492POS SM(d40:1)+H SM(d40:1)+H SM (d40:1) (d40:1) C45 H92 O6 N2 P1 787.6687525 11.773089 0.004304557 1.043306227 0.81631924 1.547749502 2765011712 1958460974 2776557683 1987762587 2400355105 2317642260 1065832212 3203653166 3218320457 1821758985 1964695251 3546729435 3308616440 3335903842 3314899026

493POS PE(20:4/20:4)+H PE(40:8)+H PE (20:4/20:4) (20:4) (20:4) C45 H75 O8 N1 P1 788.5224835 8.98643056 0.069335577 0.992913551 0.971344001 0.185353771 53498203.77 33838363.43 46728220.84 46939275.91 24058536.85 33453414.85 18098524.08 47494453.46 45906356.65 29846286.12 34458919.56 61021244.1 58213694.37 65823035.01 65803250.91

494POS PS(18:1/18:1)+H PS(36:2)+H PS (18:1/18:1) (18:1) (18:1) C42 H79 O10 N1 P1 788.5436135 9.763255263 0.082695262 0.87189024 0.605534061 0.579706602 81165594.25 97841426.21 130180905.1 301065218.3 239945466.9 166380557.5 115348770.3 136256737.1 245980778.9 108495686.7 124985187.9 155278294.4 159562601.8 158211477.7 182755737.5

495POS PC(36:1)+H PC(36:1)+H PC (36:1) (36:1) C44 H87 O8 N1 P1 788.6163835 31.63782965 0.057463242 0.961540876 0.890777004 0.155803109 11869078.28 18791589.13 22514726.97 9134015.661 19925933.66 25508390.21 7910232.114 13482649.72 34474777.39 7316935.003 18018153.61 22397256.46 18791764.64 17113302.08 16970510.79

496POS PC(36:1)+H PC(36:1)+H PC (36:1) (36:1) C44 H87 O8 N1 P1 788.6163835 11.12427558 0.002729756 1.032609213 0.76513719 2.479921629 24790688003 25057168042 25481575362 27400388664 25250486329 23860697785 15212671497 31316767108 33208735020 23569789815 24950110248 28534346148 30258187029 30401888705 30401515334

497POS SM(d40:0)+H SM(d40:0)+H SM (d40:0) (d40:0) C45 H94 O6 N2 P1 789.6844025 11.99092727 0.00933186 1.083096018 0.705589899 0.420317164 102912423.9 79655645.86 109960188.1 103000110.7 98139539.39 88615885.07 38996196.32 151354454.5 147424065.1 66488093.07 78753564.92 147652883.7 149627832.1 152104140.1 152039877.1

498POS PE(18:1/22:6)+H PE(40:7)+H PE (18:1/22:6) (18:1) (22:6) C45 H77 O8 N1 P1 790.5381335 9.682051897 0.010524304 1.084047126 0.545468816 1.672682505 644113446.5 659291454.7 745060401.5 688618069.7 682042886.6 546047192 362227821.5 841853106.2 662714209 844661941.1 938690745.9 648287060.6 877209599.4 861377856.8 861444870.8

499POS PS(36:1)+H PS(36:1)+H PS (36:1) (36:1) C42 H81 O10 N1 P1 790.5592635 10.40859585 0.039716008 1.085093887 0.558101575 1.180248333 309375706.1 441222829.3 415446345.2 578194711.1 408235951.9 620280033.5 528622657.1 544744513.5 356506645.8 550003562.4 642333688.9 386489058.4 360129221.1 360230695.1 335958681.3

49POS LPE(18:0)+H LPE(18:0)+H LPE (18:0) (18:0) C23 H49 O7 N1 P1 482.3241185 3.922158222 0.006763786 1.34207249 0.040370785 0.483213915 40779547.34 32155481.45 53558323.9 30289554.67 37806770.73 39503797.06 44600867.28 70612726.54 47130071.74 41524098.57 49185963.39 61116685.61 63305748.32 64052746 63310613.59

4POS WE(17:1)+NH4 WE(17:1)+NH4 WE (17:1) (17:1) H36 C17 O2 N1 286.2740555 1.753811998 0.011129636 0.984915303 0.968569205 0.018498615 462181.2425 1041235.839 210590.1273 1864437.826 953506.3443 426975.9345 449748.7125 1495255.946 1142474.38 344858.9644 496364.234 955421.161 1157728.645 1132629.946 1141461.095

500POS PS(36:1)+H PS(36:1)+H PS (36:1) (36:1) C42 H81 O10 N1 P1 790.5592635 12.285 0.056916305 1.091007577 0.607211017 0.232303804 22255823.3 23637824.21 29797807.43 28789870.34 25051776.46 43202100.61 15585571.61 38156848.8 40584500.78 29161819.36 37572682.69 27393991.37 31497898.06 34770786.43 31613844.36

501POS PS(36:1)+H PS(36:1)+H PS (36:1) (36:1) C42 H81 O10 N1 P1 790.5592635 13.143 0.032639381 1.386821575 0.081039033 0.389109747 11348435.29 9923500.032 12961261.69 16785705.89 13109298.72 16893157.22 8288139.747 19807201.57 22384209.8 25825834.81 16941176.54 19115606.02 23933770.46 22592635.1 22670450.59

502POS PS(36:1)+H PS(36:1)+H PS (36:1) (36:1) C42 H81 O10 N1 P1 790.5592635 15.1827566 0.022523693 1.412190015 0.082987189 0.263919387 4147105.099 4015259.712 4851874.327 8419620.715 5016635.475 5523089.957 3660767.955 8950059.465 10274978.46 7951110.631 7867573.575 6448287.807 6891593.51 6630135.868 6628973.116

503POS PS(36:1)+H PS(36:1)+H PS (36:1) (36:1) C42 H81 O10 N1 P1 790.5592635 13.78301553 0.065579776 2.377519791 0.305004584 0.742441164 17620736.35 16809198.82 23646469.15 21968304.64 20995800.67 14984153.84 17440435.44 168572019.5 24360196.74 19366109.61 16570372.7 29541799.63 23718953.37 26669144.43 26597134.89

504POS PS(36:1)+H PS(36:1)+H PS (36:1) (36:1) C42 H81 O10 N1 P1 790.5592635 16.64725158 0.026034455 1.169722804 0.586261945 0.127695497 5237199.017 3523147.588 8061468.705 6177065.827 4597970.611 9806657.33 4164036.392 14880739.63 5742221.379 4964419.791 5638510.336 8361809.993 7388257.777 7358353.225 7709853.622

505POS PS(36:1)+H PS(36:1)+H PS (36:1) (36:1) C42 H81 O10 N1 P1 790.5592635 13.42316943 0.121349512 0.951684871 0.831894106 0.180968783 9793087.968 15851919.3 16788211.43 14814364.7 36363739.23 18217045.67 13054536.02 24205828.54 17944590.31 14898897.64 14863311.09 21458202.69 16051045.57 13113941.15 16526086.4

506POS PS(36:1)+H PS(36:1)+H PS (36:1) (36:1) C42 H81 O10 N1 P1 790.5592635 14.179 0.087540667 1.103266037 0.711853261 0.153874284 8143518.029 11822799.16 11341556.61 27879079.65 10877117.34 28126107.71 8306865.87 25209831.85 19146302.37 14255850.3 21890028.48 19521010.2 23732249.89 20281443.82 23671692.91

507POS PS(36:1)+H PS(36:1)+H PS (36:1) (36:1) C42 H81 O10 N1 P1 790.5592635 11.28916809 0.033454612 1.04797024 0.963795056 1.077492863 37332714.6 1647605730 79367737.27 147030931.1 113989542.7 149906411.6 50109916.66 158397151.8 70634771.75 162145737.2 108092788.9 1730199153 77858219.99 75263802.73 80473691.19

508POS PC(38:6p)+H PC(38:6p)+H PC (38:6p) (38:6p) C46 H81 O7 N1 P1 790.5745185 9.737 0.00351555 1.227766013 0.358672347 1.089298109 173665072.2 277757635.1 168021014.9 210820789 457224131 232351547.7 118208365.7 349820828.1 393232306.1 353703436.4 358326665 292716529.4 336835605 338899859 338890094.7

509POS PC(37:6)+H PC(37:6)+H PC (37:6) (37:6) C45 H79 O8 N1 P1 792.5537835 10.42827369 0.009003071 0.935003449 0.629945517 0.502157087 646388925 424853594.1 720312955.2 499625679.8 296154023.5 454566431.5 507607200.2 537977323.2 465269711.7 402594589.6 430707346.4 500032325.2 559725655.4 568556934.3 568534187.7

50POS LPC(16:0e)+H LPC(16:0e)+H LPC (16:0e) (16:0e) C24 H53 O6 N1 P1 482.3605035 3.120871523 0.010129921 1.27318016 0.515606918 0.086516869 4472042.798 1549218.082 5231750.248 1977568.123 3540365.978 4787719.87 3822456.009 3509799.381 4515998.18 1897595.768 2806142.134 10896073.21 5659234.75 5759638.276 5759770.113

510POS PC(37:6)+H PC(37:6)+H PC (37:6) (37:6) C45 H79 O8 N1 P1 792.5537835 9.942475165 0.007384966 1.045698845 0.599824495 0.632643349 247603411.9 233765596.2 285702359.4 256781295.3 283003100.4 249560160.9 180332341.5 293388402.1 269567481.7 262974245.9 298754497.1 322525366.4 354851539.2 350345686.9 354879429.1

511POS PC(37:6)+H PC(37:6)+H PC (37:6) (37:6) C45 H79 O8 N1 P1 792.5537835 8.89134025 0.005347063 1.036581453 0.921116634 0.503630201 93100831.49 174181578.8 83602120.29 67547703.14 72534615.62 58512548.82 30324355.92 146741111 200340378.1 37434736.46 47108659.79 107630911.9 131222928 132578939.9 132241171.6

512POS PC(38:6e)+H PC(38:6e)+H PC (38:6e) (38:6e) C46 H83 O7 N1 P1 792.5901685 9.882301995 0.003378024 1.450709449 0.143818086 2.170856146 353695991.1 494769829.8 331683696.1 384028033.6 808407650.3 552547951.9 216245584.3 939889547 1050720498 677485216.2 673674893 685502565.1 666729503.9 670638856.3 670652614.4

513POS PC(38:6e)+H PC(38:6e)+H PC (38:6e) (38:6e) C46 H83 O7 N1 P1 792.5901685 10.25728211 0.005879083 1.331762827 0.222382011 1.097616104 91311445.67 129643563.3 94804580.72 105596138.4 227980953.8 144030874.8 50443452.15 184581277.7 180318251.2 230424381.9 220467632 190342425.4 187876494.7 189818923.4 187922434.4

514POS PC(38:6e)+H PC(38:6e)+H PC (38:6e) (38:6e) C46 H83 O7 N1 P1 792.5901685 9.586625448 0.009087403 1.655592917 0.319663225 0.809134994 50982393.18 38280367.19 36398264.22 32367903.79 48659086.67 48607194.59 27892298.45 41462570.48 53504767.97 201376923.5 48925141.45 49503239.03 51731854.24 50917692.48 51723333.48

515POS TG(16:1/14:0/16:1)+NH4 TG(46:2)+NH4 TG (16:1/14:0/16:1) (16:1) (14:0) (16:1) C49 H94 O6 N1 792.7075655 14.161 0.010153551 0.998676349 0.994954047 0.030222852 4134336.922 2669863.672 4647610.807 7582820.554 6816236.521 6554434.716 2601698.223 8140975.436 5856584.748 5460519.382 3913635.151 6388996.93 8661912.231 8815514.93 8662645.908

516POS PC(37:5)+H PC(37:5)+H PC (37:5) (37:5) C45 H81 O8 N1 P1 794.5694335 9.251333357 0.012264759 1.155196855 0.436272162 0.813210536 279405825.1 221747461.2 324188872.7 192312067.8 245661739.5 229973267 114868976.1 326076980.8 417227904.3 240999258 260127882.2 365742024.5 369248759.1 377185396 377225376.3

517POS PC(37:5)+H PC(37:5)+H PC (37:5) (37:5) C45 H81 O8 N1 P1 794.5694335 10.92088625 0.019337852 0.998086592 0.98643372 0.076681114 110597182.1 100506422.1 128469753.3 114785357 93411582.98 83397142.16 67442443.47 125148685.9 119363242.9 91733449.69 99553936.89 126718000.2 129731340 134113692.2 134235396.4

518POS PC(37:5)+H PC(37:5)+H PC (37:5) (37:5) C45 H81 O8 N1 P1 794.5694335 10.11351159 0.152549198 0.99648659 0.993256204 0.118493996 78639378.98 75197304.25 76903846.17 83326712.67 65766197.51 314333319.6 80113501.89 220749934.4 79101897.07 156422095.5 65436565.87 89903872.1 163481408.8 127574413.4 125980030.4

519POS PE(20:1/20:4)+H PE(40:5)+H PE (20:1/20:4) (20:1) (20:4) C45 H81 O8 N1 P1 794.5694335 10.66428581 0.001407653 0.995473133 0.96704766 0.30239304 388311305.7 297310349.9 419861405.8 317777831.2 243712407.1 329376270.4 283228315 371493659.3 381027884 267697749.3 282062056.3 401802697.4 405570218.2 405692088.1 406615289.8

51POS LPE(18:1p)+Na LPE(18:1p)+Na LPE (18:1p) (18:1p) C23 H46 O6 N1 P1 Na1 486.2954985 3.330248152 0.030751907 1.553989541 0.072396875 0.146452419 971594.032 1009651.133 940736.7958 893465.9929 1830713.964 1410962.557 989548.7455 2706845.197 1372716.918 1614554.517 2663640.627 1619391.619 1950071.601 2057704.235 2057823.718

520POS PC(38:4p)+H PC(38:4p)+H PC (38:4p) (38:4p) C46 H85 O7 N1 P1 794.6058185 10.59084832 0.007957206 1.410678709 0.197631149 1.612950303 153714933.7 253978874.5 146408910.1 163068971.7 445769712.3 233994052.2 93433993 327612301.8 348456649.5 432264393.5 443318899.7 325540865.4 335675230.4 340316973.6 340371551.8

521POS PC(38:4p)+H PC(38:4p)+H PC (38:4p) (38:4p) C46 H85 O7 N1 P1 794.6058185 10.16717898 0.004590653 1.017319581 0.938863891 0.47018067 524694559.4 389307234.8 534898127.4 492909764.8 688287431.1 784650767 245783791.2 564534592.8 667482524.2 464834123.9 476210454.8 1055044401 710470541.2 716153100.8 716146435.7

522POS PC(36:2e)+Na PC(36:2e)+Na PC (36:2e) (36:2e) C44 H86 O7 N1 P1 Na1 794.6034135 11.45852596 0.01102364 1.095108854 0.62144134 0.542313398 66922646.45 93267047.12 63737882.67 79695578.51 140539742.1 85173799.28 40207505.99 96418350.1 98849605.72 118453099.2 117643805.4 108108936.2 97477027.39 97481477.17 95629806.59

523POS TG(16:0/14:0/16:1)+NH4 TG(46:1)+NH4 TG (16:0/14:0/16:1) (16:0) (14:0) (16:1) C49 H96 O6 N1 794.7232155 14.71844472 0.006706267 0.924174057 0.673253597 0.089311767 13669825.44 9541576.722 14290635.97 22227717.41 14523121.59 15671059.33 7551980.088 20897945.1 17605962.93 11835585.73 10395323.27 14818572.08 22019286.04 22242991.67 21963162.7

524POS PC(35:1)+Na PC(35:1)+Na PC (35:1) (35:1) C43 H84 O8 N1 P1 Na1 796.5826785 10.77856462 0.027517563 0.828018516 0.540921045 0.818706744 295039726.7 305192511.8 127523268.8 210612163.6 83202894.76 100185508.5 259692288.2 83354888.29 213729084.7 191954914.1 88237523.38 91866100.66 79000507.39 79018895.4 82836206.99

525POS PC(37:4)+H PC(37:4)+H PC (37:4) (37:4) C45 H83 O8 N1 P1 796.5850835 9.86244445 0.004877274 1.266611534 0.069815572 0.971212616 165772035.1 154488725.4 167464541.6 109165266.8 147094170.5 121348657.5 116168771.7 203977299.3 222680648.3 198306386.7 201095495.2 153812660.2 194332554.3 195978479.1 194331817.9

526POS PC(37:4)+H PC(37:4)+H PC (37:4) (37:4) C45 H83 O8 N1 P1 796.5850835 11.15214916 0.015055634 1.064940409 0.602006158 0.442279883 166529168.7 171323097.7 211110832.2 171650724.8 152890010 165330009.4 106349304.9 249201947.8 203750457.4 167578057 182245138.3 197171231.6 220247039.6 225469847.7 226558467.5

527POS PC(38:4e)+H PC(38:4e)+H PC (38:4e) (38:4e) C46 H87 O7 N1 P1 796.6214685 10.71366015 0.020441805 1.177939261 0.524057638 0.588482522 79543861.85 77817877.34 74417350.99 215802081.1 115026400.5 121530617.7 113075831 103623859.3 120066437.3 81889476.48 228788528.8 158429100.8 113569606.6 117649331.2 113590202.7

528POS PC(36:1e)+Na PC(36:1e)+Na PC (36:1e) (36:1e) C44 H88 O7 N1 P1 Na1 796.6190635 11.56535245 0.002035067 0.798539221 0.237962605 1.063851798 121444809.7 113409858.2 110379152.6 132107909.9 124245158.3 128887372.2 52809537.57 126065127.3 138292873.7 58539710.28 53712249.72 153892848.5 130366787.1 129908407.3 129908277.5
[truncated: 170,263 more chars]
