# Supplementary material for: Untargeted lipidomic analysis and network pharmacology for parthenolide treated papillary thyroid carcinoma cells
Source: BMC Complement Med Ther. 2023 Apr 24;23:130. doi: 10.1186/s12906-023-03944-7 (PMC10123985; doi:10.1186/s12906-023-03944-7)
Supplement: Supplementary file 3 — Additional file 3. Lipid class analysis between two groups. [file 12906_2023_3944_MOESM3_ESM.docx]

Additional file 3. Lipid class analysis between two groups.

| class | groupname | T-1 | T-2 | T-3 | T-4 | T-5 | T-6 | C-1 | C-2 | C-3 | C-4 | C-5 | C-6 | Mean_group1 | Mean_group2 | FC | Pvalue |
| --- | --- | --- | --- | --- | --- | --- | --- | --- | --- | --- | --- | --- | --- | --- | --- | --- | --- |
| AcCa | T_vs_C | 2.56E+08 | 1.17E+09 | 1.15E+09 | 6.25E+08 | 5.96E+08 | 2.12E+09 | 2.67E+08 | 1.16E+09 | 3.45E+08 | 1.36E+09 | 7.69E+08 | 1.27E+09 | 9.86E+08 | 8.62E+08 | 1.144359 | 0.715578 |
| Cer | T_vs_C | 2.92E+09 | 7.12E+09 | 6.74E+09 | 3.99E+09 | 5.18E+09 | 8.11E+09 | 5.43E+09 | 4.78E+09 | 4.88E+09 | 4.8E+09 | 5.84E+09 | 5.46E+09 | 5.68E+09 | 5.2E+09 | 1.091696 | 0.579722 |
| CerG1 | T_vs_C | 1.26E+09 | 2.97E+09 | 3.29E+09 | 9.75E+08 | 8.74E+08 | 3.92E+09 | 2.56E+09 | 2.17E+09 | 2.53E+09 | 2.27E+09 | 1.93E+09 | 2.76E+09 | 2.21E+09 | 2.37E+09 | 0.934415 | 0.786151 |
| CerG2 | T_vs_C | 4.25E+08 | 1.84E+09 | 1.64E+09 | 8.57E+08 | 1.16E+09 | 1.81E+09 | 1.19E+09 | 1.1E+09 | 1.28E+09 | 9.68E+08 | 1.03E+09 | 1.39E+09 | 1.29E+09 | 1.16E+09 | 1.109811 | 0.611093 |
| CerG3 | T_vs_C | 3.54E+08 | 9.88E+08 | 7.58E+08 | 9.55E+08 | 1.02E+09 | 9.51E+08 | 3.99E+08 | 5.07E+08 | 5.91E+08 | 7.42E+08 | 7.52E+08 | 6.63E+08 | 8.38E+08 | 6.09E+08 | 1.37534 | 0.081972 |
| CerG3GNAc1 | T_vs_C | 1.25E+08 | 2.72E+08 | 2.17E+08 | 1.87E+08 | 2.04E+08 | 3.7E+08 | 1.5E+08 | 1.21E+08 | 2.2E+08 | 2.34E+08 | 1.87E+08 | 2.5E+08 | 2.29E+08 | 1.94E+08 | 1.183765 | 0.394611 |
| ChE | T_vs_C | 1.6E+08 | 3E+08 | 3.06E+08 | 1.45E+08 | 1.43E+08 | 3.51E+08 | 1.68E+08 | 41816590 | 1.6E+08 | 68542534 | 1.63E+08 | 2.23E+08 | 2.34E+08 | 1.37E+08 | 1.706474 | 0.069049 |
| CL | T_vs_C | 4.21E+08 | 1.45E+09 | 9.93E+08 | 9.81E+08 | 9.99E+08 | 1.52E+09 | 6.87E+08 | 6.48E+08 | 9.83E+08 | 1.03E+09 | 9.09E+08 | 1.01E+09 | 1.06E+09 | 8.78E+08 | 1.206116 | 0.326362 |
| Co | T_vs_C | 1.9E+08 | 4.75E+08 | 5.28E+08 | 4.23E+08 | 4.83E+08 | 3.28E+08 | 3.63E+08 | 3.18E+08 | 3.81E+08 | 2.77E+08 | 3.23E+08 | 2.63E+08 | 4.04E+08 | 3.21E+08 | 1.260563 | 0.155879 |
| DG | T_vs_C | 4.9E+08 | 3.69E+09 | 2.55E+09 | 1.43E+09 | 1.28E+09 | 2.3E+09 | 1.93E+09 | 2.15E+09 | 1.79E+09 | 2.32E+09 | 2E+09 | 1.48E+09 | 1.96E+09 | 1.95E+09 | 1.005519 | 0.982428 |
| DGDG | T_vs_C | 11534303 | 50237319 | 45455232 | 24793290 | 27903998 | 64322925 | 28634143 | 31604504 | 28009586 | 34016607 | 45897755 | 49456770 | 37374511 | 36269894 | 1.030455 | 0.901952 |
| FA | T_vs_C | 6685942 | 1.52E+08 | 69449796 | 94167911 | 57547344 | 49116789 | 9287094 | 33361929 | 8943843 | 19591432 | 63047277 | 15181491 | 71430650 | 24902178 | 2.86845 | 0.056555 |
| GM1 | T_vs_C | 9756929 | 5532251 | 2498118 | 8654422 | 4074309 | 4178562 | 4189659 | 925699.3 | 7798032 | 12566197 | 7474649 | 4670461 | 5782432 | 6270783 | 0.922123 | 0.811462 |
| GM3 | T_vs_C | 1.09E+09 | 1.82E+09 | 1.32E+09 | 1.53E+09 | 1.36E+09 | 2.24E+09 | 1.09E+09 | 7.81E+08 | 1.78E+09 | 1.95E+09 | 1.36E+09 | 1.67E+09 | 1.56E+09 | 1.44E+09 | 1.084971 | 0.632115 |
| LPC | T_vs_C | 1.2E+09 | 2.52E+09 | 2.77E+09 | 1.48E+09 | 1.59E+09 | 3.62E+09 | 1.76E+09 | 1.32E+09 | 2.03E+09 | 1.24E+09 | 2.03E+09 | 1.98E+09 | 2.2E+09 | 1.73E+09 | 1.272697 | 0.274576 |
| LPE | T_vs_C | 2.65E+08 | 5.39E+08 | 4.25E+08 | 3.29E+08 | 4.16E+08 | 5.33E+08 | 3.4E+08 | 2.96E+08 | 4E+08 | 2.54E+08 | 3.94E+08 | 3.67E+08 | 4.18E+08 | 3.42E+08 | 1.222043 | 0.162233 |
| LPG | T_vs_C | 10119375 | 31183767 | 36347741 | 25983042 | 22806834 | 39465160 | 9152077 | 16376627 | 13381405 | 9052695 | 31583420 | 22334799 | 27650987 | 16980170 | 1.628428 | 0.08582 |
| LPI | T_vs_C | 44537546 | 16957461 | 11302354 | 32766138 | 25872831 | 29943594 | 11313489 | 5701758 | 29906184 | 9834668 | 25334933 | 21218976 | 26896654 | 17218334 | 1.562094 | 0.151108 |
| LPS | T_vs_C | 5103148 | 6313321 | 4288339 | 6763967 | 5665517 | 5326345 | 2509197 | 2304226 | 6631946 | 6064541 | 6011478 | 5866850 | 5576773 | 4898040 | 1.138572 | 0.4551 |
| LSM | T_vs_C | 9628525 | 27898922 | 27448150 | 14277412 | 14515482 | 30255547 | 17194920 | 14082056 | 20865900 | 19486748 | 19312750 | 16800495 | 20670673 | 17957145 | 1.151111 | 0.4851 |
| MGDG | T_vs_C | 1.26E+08 | 1.78E+08 | 1.94E+08 | 74411717 | 73245188 | 2.88E+08 | 2.32E+08 | 1.46E+08 | 3.18E+08 | 2.61E+08 | 94898497 | 2.23E+08 | 1.56E+08 | 2.13E+08 | 0.732159 | 0.252799 |
| PA | T_vs_C | 31713033 | 1.1E+08 | 96120704 | 71730315 | 69738703 | 1.29E+08 | 1.39E+08 | 68996256 | 88510276 | 1.04E+08 | 94939430 | 85179009 | 84655690 | 96654344 | 0.87586 | 0.497092 |
| PC | T_vs_C | 1.76E+11 | 3.67E+11 | 3.95E+11 | 2.64E+11 | 2.82E+11 | 4.13E+11 | 3.06E+11 | 2.84E+11 | 3.18E+11 | 3.16E+11 | 3.18E+11 | 3.2E+11 | 3.16E+11 | 3.1E+11 | 1.018824 | 0.880139 |
| PE | T_vs_C | 2.71E+10 | 6.05E+10 | 5.2E+10 | 4.85E+10 | 5.28E+10 | 6.19E+10 | 4.47E+10 | 3.89E+10 | 4.83E+10 | 4.63E+10 | 5.05E+10 | 4.71E+10 | 5.05E+10 | 4.6E+10 | 1.097965 | 0.42156 |
| PG | T_vs_C | 3E+08 | 1.13E+09 | 9.34E+08 | 7.26E+08 | 8.27E+08 | 1.22E+09 | 6.3E+08 | 5.24E+08 | 7.46E+08 | 7.77E+08 | 8.81E+08 | 7.07E+08 | 8.56E+08 | 7.11E+08 | 1.204441 | 0.334203 |
| PI | T_vs_C | 7.43E+09 | 1.32E+10 | 8.76E+09 | 1.04E+10 | 1.04E+10 | 1.3E+10 | 6.8E+09 | 6.61E+09 | 1.05E+10 | 1.11E+10 | 8.29E+09 | 1.24E+10 | 1.05E+10 | 9.28E+09 | 1.133837 | 0.379778 |
| PIP2 | T_vs_C | 22688409 | 15995251 | 14210363 | 23026088 | 24218328 | 10365776 | 11555579 | 16564844 | 15537529 | 19256800 | 18849949 | 14640337 | 18417369 | 16067506 | 1.146249 | 0.386867 |
| PS | T_vs_C | 5.64E+09 | 1.04E+10 | 9.14E+09 | 7.69E+09 | 8.79E+09 | 1.2E+10 | 7E+09 | 8.13E+09 | 8.3E+09 | 8.52E+09 | 8.21E+09 | 9.73E+09 | 8.95E+09 | 8.31E+09 | 1.075918 | 0.526824 |
| SM | T_vs_C | 2.9E+10 | 6.82E+10 | 6.76E+10 | 4.99E+10 | 5.09E+10 | 7.09E+10 | 5.44E+10 | 4.57E+10 | 5.6E+10 | 4.89E+10 | 5.69E+10 | 5.15E+10 | 5.61E+10 | 5.22E+10 | 1.074278 | 0.581499 |
| So | T_vs_C | 61579383 | 88513695 | 71214612 | 72523800 | 79244640 | 1.18E+08 | 63083028 | 2.32E+08 | 75651753 | 55511474 | 92600589 | 96928755 | 81811742 | 1.03E+08 | 0.797116 | 0.472567 |
| SQDG | T_vs_C | 7136091 | 10502979 | 8326052 | 10730934 | 10636905 | 6314438 | 8625839 | 8898562 | 7851611 | 8842831 | 11694607 | 8578922 | 8941233 | 9082062 | 0.984494 | 0.88689 |
| TG | T_vs_C | 1.06E+09 | 6.6E+09 | 4.58E+09 | 3.71E+09 | 3.41E+09 | 2.65E+09 | 2.53E+09 | 2.53E+09 | 2.6E+09 | 5.22E+09 | 3.88E+09 | 2.57E+09 | 3.67E+09 | 3.22E+09 | 1.138337 | 0.625476 |
| WE | T_vs_C | 8899409 | 92826199 | 54566652 | 53440863 | 42383678 | 1.01E+08 | 24044401 | 85986725 | 23117385 | 1.08E+08 | 62179750 | 35781114 | 58865815 | 56435021 | 1.043072 | 0.905084 |
| ZyE | T_vs_C | 347963.5 | 6559386 | 6802583 | 2291246 | 1827873 | 2213862 | 6106148 | 5320870 | 6069431 | 10895027 | 3661434 | 2074389 | 3340485 | 5687883 | 0.587299 | 0.18285 |
