# Supplementary material for: Untargeted lipidomic analysis and network pharmacology for parthenolide treated papillary thyroid carcinoma cells
Source: BMC Complement Med Ther. 2023 Apr 24;23:130. doi: 10.1186/s12906-023-03944-7 (PMC10123985; doi:10.1186/s12906-023-03944-7)
Supplement: Supplementary file 4 — Additional file 4. The correlation analysis between the significant lipid species. [file 12906_2023_3944_MOESM4_ESM.docx]

Additional file 4. The correlation analysis between the significant lipid species.

| LipidIon1 | LipidIon2 | Coefficient | P value | ABS | LABLE |
| --- | --- | --- | --- | --- | --- |
| CerG3(d18:1/24:1) | CerG3(d18:1/24:1) | 1 | 0 | 1 | pos |
| LPE(18:0) | CerG3(d18:1/24:1) | 0.423907 | 0.169655 | 0.423907 | pos |
| PE(16:1/17:0) | CerG3(d18:1/24:1) | -0.17172 | 0.593591 | 0.171719 | neg |
| PC(12:0e/16:0) | CerG3(d18:1/24:1) | 0.64916 | 0.022361 | 0.64916 | pos |
| PC(18:0/20:4) | CerG3(d18:1/24:1) | 0.240984 | 0.450539 | 0.240984 | pos |
| PI(19:0/20:4) | CerG3(d18:1/24:1) | 0.711735 | 0.009426 | 0.711735 | pos |
| LPC(28:0) | CerG3(d18:1/24:1) | 0.688414 | 0.013312 | 0.688414 | pos |
| ChE(22:6) | CerG3(d18:1/24:1) | 0.529409 | 0.076714 | 0.529409 | pos |
| PC(34:1) | CerG3(d18:1/24:1) | -0.10424 | 0.74716 | 0.104237 | neg |
| PC(16:0p/18:0) | CerG3(d18:1/24:1) | -0.33445 | 0.287986 | 0.334445 | neg |
| LPE(18:0) | LPE(18:0) | 1 | 0 | 1 | pos |
| PE(16:1/17:0) | LPE(18:0) | -0.34482 | 0.272346 | 0.344822 | neg |
| PC(12:0e/16:0) | LPE(18:0) | 0.7685 | 0.003497 | 0.7685 | pos |
| PC(18:0/20:4) | LPE(18:0) | 0.418951 | 0.175234 | 0.418951 | pos |
| PI(19:0/20:4) | LPE(18:0) | 0.621981 | 0.030813 | 0.621981 | pos |
| LPC(28:0) | LPE(18:0) | 0.73372 | 0.006604 | 0.73372 | pos |
| ChE(22:6) | LPE(18:0) | 0.746983 | 0.005242 | 0.746983 | pos |
| PC(34:1) | LPE(18:0) | -0.1863 | 0.56209 | 0.186301 | neg |
| PC(16:0p/18:0) | LPE(18:0) | -0.22718 | 0.477658 | 0.227178 | neg |
| PE(16:1/17:0) | PE(16:1/17:0) | 1 | 0 | 1 | pos |
| PC(12:0e/16:0) | PE(16:1/17:0) | -0.33403 | 0.288618 | 0.334033 | neg |
| PC(18:0/20:4) | PE(16:1/17:0) | -0.83445 | 0.000736 | 0.834454 | neg |
| PI(19:0/20:4) | PE(16:1/17:0) | -0.3795 | 0.223719 | 0.379499 | neg |
| LPC(28:0) | PE(16:1/17:0) | -0.28828 | 0.363527 | 0.288276 | neg |
| ChE(22:6) | PE(16:1/17:0) | -0.30402 | 0.336688 | 0.304022 | neg |
| PC(34:1) | PE(16:1/17:0) | 0.595069 | 0.041237 | 0.595069 | pos |
| PC(16:0p/18:0) | PE(16:1/17:0) | 0.627551 | 0.02892 | 0.627551 | pos |
| PC(12:0e/16:0) | PC(12:0e/16:0) | 1 | 0 | 1 | pos |
| PC(18:0/20:4) | PC(12:0e/16:0) | 0.360455 | 0.249728 | 0.360455 | pos |
| PI(19:0/20:4) | PC(12:0e/16:0) | 0.490959 | 0.105065 | 0.490959 | pos |
| LPC(28:0) | PC(12:0e/16:0) | 0.991661 | 3.13E-10 | 0.991661 | pos |
| ChE(22:6) | PC(12:0e/16:0) | 0.627072 | 0.029079 | 0.627072 | pos |
| PC(34:1) | PC(12:0e/16:0) | -0.49491 | 0.10187 | 0.494912 | neg |
| PC(16:0p/18:0) | PC(12:0e/16:0) | -0.24053 | 0.451425 | 0.240527 | neg |
| PC(18:0/20:4) | PC(18:0/20:4) | 1 | 0 | 1 | pos |
| PI(19:0/20:4) | PC(18:0/20:4) | 0.316196 | 0.316695 | 0.316196 | pos |
| LPC(28:0) | PC(18:0/20:4) | 0.314495 | 0.319447 | 0.314495 | pos |
| ChE(22:6) | PC(18:0/20:4) | 0.541004 | 0.069316 | 0.541004 | pos |
| PC(34:1) | PC(18:0/20:4) | -0.43603 | 0.156485 | 0.436029 | neg |
| PC(16:0p/18:0) | PC(18:0/20:4) | -0.84434 | 0.000551 | 0.844344 | neg |
| PI(19:0/20:4) | PI(19:0/20:4) | 1 | 0 | 1 | pos |
| LPC(28:0) | PI(19:0/20:4) | 0.466018 | 0.126764 | 0.466018 | pos |
| ChE(22:6) | PI(19:0/20:4) | 0.567151 | 0.054467 | 0.567151 | pos |
| PC(34:1) | PI(19:0/20:4) | -0.09771 | 0.762583 | 0.097707 | neg |
| PC(16:0p/18:0) | PI(19:0/20:4) | -0.26759 | 0.40044 | 0.267586 | neg |
| LPC(28:0) | LPC(28:0) | 1 | 0 | 1 | pos |
| ChE(22:6) | LPC(28:0) | 0.622448 | 0.030651 | 0.622448 | pos |
| PC(34:1) | LPC(28:0) | -0.47833 | 0.115716 | 0.47833 | neg |
| PC(16:0p/18:0) | LPC(28:0) | -0.22299 | 0.486034 | 0.222987 | neg |
| ChE(22:6) | ChE(22:6) | 1 | 0 | 1 | pos |
| PC(34:1) | ChE(22:6) | -0.09325 | 0.773156 | 0.093251 | neg |
| PC(16:0p/18:0) | ChE(22:6) | -0.2911 | 0.358636 | 0.291099 | neg |
| PC(34:1) | PC(34:1) | 1 | 0 | 1 | pos |
| PC(16:0p/18:0) | PC(34:1) | 0.364061 | 0.244673 | 0.364061 | pos |
| PC(16:0p/18:0) | PC(16:0p/18:0) | 1 | 0 | 1 | pos |
